# The Perspectives of Patients on Health-Care for Co-Morbid Diabetes and Chronic Kidney Disease: A Qualitative Study

**DOI:** 10.1371/journal.pone.0146615

**Published:** 2016-01-05

**Authors:** Clement Lo, Dragan Ilic, Helena Teede, Alan Cass, Greg Fulcher, Martin Gallagher, Greg Johnson, Peter G. Kerr, Tim Mathew, Kerry Murphy, Kevan Polkinghorne, Rowan Walker, Sophia Zoungas

**Affiliations:** 1 Diabetes and Vascular Research Program, Monash Centre for Health Research and Implementation, School of Public Health and Preventive Medicine, Monash University, Clayton, Victoria, Australia; 2 Diabetes and Vascular Medicine Unit, Monash Health, Clayton, Victoria, Australia; 3 Department of Epidemiology & Preventive Medicine, School of Public Health and Preventive Medicine, Monash University, Prahran, Victoria, Australia; 4 Menzies School of Health Research, Casuarina, Northern Territory, Australia; 5 The George Institute for Global Health, Camperdown, New South Wales, Australia; 6 Department of Diabetes and Endocrinology, Royal North Shore Hospital, St Leonards, New South Wales, Australia; 7 Department of Nephrology, Concord Hospital, Concord, New South Wales, Australia; 8 Diabetes Australia, Canberra, Australian Capital Territory, Australia; 9 Department of Nephrology, Monash Health, Clayton, Victoria, Australia; 10 Kidney Health Australia, Adelaide, South Australia, Australia; 11 Department of Renal Medicine, Alfred Health, Prahran, Victoria, Australia; University of Stirling, UNITED KINGDOM

## Abstract

**Background:**

Multi-morbidity due to diabetes and chronic kidney disease (CKD) remains challenging for current health-systems, which focus on single diseases. As a first step toward health-care improvement, we explored the perspectives of patients and their carers on factors influencing the health-care of those with co-morbid diabetes and CKD.

**Methods:**

In this qualitative study participants with co-morbid diabetes and CKD were purposively recruited using maximal variation sampling from 4 major tertiary health-services from 2 of Australia’s largest cities. Separate focus groups were conducted for patients with CKD stages 3, 4 and 5. Findings were triangulated with semi-structured interviews of carers of patients. Discussions were transcribed verbatim and thematically analysed.

**Results:**

Twelve focus groups with 58 participants and 8 semi-structured interviews of carers were conducted. Factors influencing health-care of co-morbid diabetes and CKD grouped into patient and health service level factors. Key patient level factors identified were patient self-management, socio-economic situation, and adverse experiences related to co-morbid diabetes and CKD and its treatment. Key health service level factors were prevention and awareness of co-morbid diabetes and CKD, poor continuity and coordination of care, patient and carer empowerment, access and poor recognition of psychological co-morbidity. Health-service level factors varied according to CKD stage with poor continuity and coordination of care and patient and carer empowerment emphasized by participants with CKD stage 4 and 5, and access and poor recognition of psychological co-morbidity emphasised by participants with CKD stage 5 and carers.

**Conclusions:**

According to patients and their carers the health-care of co-morbid diabetes and CKD may be improved via a preventive, patient-centred health-care model which promotes self-management and that has good access, continuity and coordination of care and identifies and manages psychological morbidity.

## Introduction

Multi-morbidity can be defined as the co-existence of more than one chronic condition where one is not necessarily more central than the other [1]. Multi-morbidity is increasing in prevalence globally, due to the aging population and the rising prevalence of chronic non-communicable diseases [2], such as diabetes. Diabetes is the leading cause of end stage kidney disease (ESKD) globally [3].

Together, co-morbid diabetes and chronic kidney disease (CKD) pose an emerging public health problem. Firstly, co-morbid diabetes and CKD is associated with an increased risk of morbidity, mortality and cardiovascular disease [4]. Secondly, significant economic costs are associated with the health-care of patients with co-morbid diabetes and CKD, with 24.2 billion USD spent in 2012 alone in the United States of America [5]. Thirdly, there is growing evidence that the health-care of patients with co-morbid diabetes and CKD is suboptimal with studies reporting treatment target gaps and failure to meet other recommended health indicators of optimal clinical care such as regular HbA1c monitoring or treatment of anaemia [5–9]. Under-recognition of CKD and late referral to nephrologists have also been reported [10, 11]. Fourthly, given that most contemporary health systems are a conglomerate of services framed around a single disease, they are poorly equipped to deal with the multi-morbidity [1, 2] of diabetes and CKD.

With the challenge of multi-morbidity expected to grow, there have been recent calls to develop more person-centred health systems–“underpinned by technology-enabled primary, community and social care that sustain and improve health and do not merely react to disease” [2]. However, the perspectives and experiences of patients and their carers need to inform the development of person-centred health systems.

A handful of qualitative studies have explored the disease-related experiences of patients with co-morbid diabetes and CKD [12–16] but none have explored patients’ perspectives on the important factors affecting optimal health-care. Consequently, in this novel qualitative study, we explored the perspectives of patients and their carers on the factors influencing health-care of those with co-morbid diabetes and chronic kidney disease (CKD).

## Materials and Methods

This qualitative study, underpinned by a pragmatic worldview [17, 18] was a research collaboration between 4 large tertiary health-services, 2 research institutes, and 2 national consumer stake-holder groups (Diabetes Australia and Kidney Health Australia). Focus groups were conducted amongst patients with co-morbid diabetes and CKD to gain a broad range of ideas and perspectives concerning the management of diabetes and CKD and to allow emergence and discussion of key issues and perspectives, which is less likely to occur in a semi-structured interview dynamic [17, 19]. The experiences and perspectives of patients with different CKD stages were thought to be different enough to warrant organisation of separate focus groups for patients with CKD stages 3, 4 and 5 respectively. Patient focus group findings were cross-referenced or triangulated with separate semi-structured interviews with carers, which also allowed deeper exploration of themes raised during focus groups [17, 19]. The study was approved by the Human Research Ethics Committees of all participating institutions (Monash Health Human Research Ethics Committee, Alfred Health Research Ethics Committee, Monash University Human Research Ethics Committee, Northern Sydney Local Health District Human Research Ethics Committee, Sydney Local Health District Human Research Ethics Committee and the University of Sydney Human Research Ethics Committee).

### Participant selection and setting

Patients with diabetes and CKD (stages 3–5, eGFR < 60 mL/min/1.73 m^2^) and their carers from Monash Health and Alfred Health in Melbourne, and Royal North Shore Hospital and Concord Hospital in Sydney, were purposively sampled for information rich cases [17]. Potential participants were identified by senior clinicians of public specialist clinics or nurse unit managers of dialysis units such that the expressed views were likely to be representative of the patient cohort at the clinic/dialysis unit, and then approached by members of the research team who invited them to participate in the study. Within each focus group for a particular CKD stage, we sought to recruit an equal number of both genders. In addition for focus groups of patients with CKD stage 5 we sought to recruit equal numbers of patients who had commenced dialysis (haemodialysis or peritoneal dialysis) as patients who had not commenced dialysis (pre-dialysis). Prior to involvement, participants voluntarily gave written informed consent for involvement in and audio-recording of discussions, after receiving written information regarding the study, having an opportunity to ask questions, being ensured their involvement would not affect their normal medical treatment, and after the investigators were satisfied that they understood and were capable of participating in the study. Participants were assured of the de-identification and confidentiality of all data and reimbursed for their time with a gift voucher (50 AUD$). Potential participants who were incapable of giving informed consent or who had an unstable mental state were excluded. Focus groups and semi-structured interviews were conducted in a meeting room at the main hospital of each tertiary health-service.

### Data collection

The focus group and semi-structured interview questions (S1 Table) were developed by the research team, informed by a review of the literature and consensus with leading stakeholders. These open-ended questions were piloted in 5 semi-structured interviews of patients recruited from a diabetes clinic at Monash Health. An iterative approach was used with additional questions added according to themes raised in preceding focus groups. Focus group and semi-structured interviews were conducted by the same researcher (CL, a male Endocrinologist who had no prior therapeutic relationship with participants) from May 2013 to February 2014 and audio-taped. Focus groups and semi-structured interviews were conducted until a point of data saturation was reached across and within CKD stages, with no new ideas emerging [20]. De-identified audiotaped discussions were transcribed verbatim by an independent transcribing service.

### Data analysis

Data analysis was an ongoing process from the initiation of data collection to the study’s end. CL, kept a reflexive journal, recording entries after each focus group and interview to check for potential biases as a clinician/researcher and to identify recurring thoughts and ideas from participants. Transcripts were analysed independently by two researchers (CL and KM) using a generic inductive thematic approach as described by Patton [17] and Harding [21]. Both researchers immersed themselves in the data reading the transcript several times. Primary patterns within the data were identified, coded in a constant comparative manner, and also cross-referenced to notes from CL’s reflexive journal. Data was then categorised into themes [17, 21, 22], with variations noted primarily between CKD groups, but also between gender and dialysis modalities. Consensus concerning the emerging themes was then reached between the two researchers with any conflicts resolved through discussion with a third researcher (DI).

## Results

Fifty-eight participants, with type 2 diabetes of mean duration 19.2 years and CKD of median duration 6.0 years, participated in 12 focus groups. The majority of the participants were aged between 61 to 70 years, with 3 participants aged 41 to 50 years, 10 participants aged 51 to 60 years, 25 participants aged 61 to 70 years, 17 participants aged 71 to 90 years and 3 aged 81 to 90 years old. Focus groups ranged in size from 3 to 7 patients. Despite attempts for maximal variation sampling, there was a predominance of male participants, and a predominance of participants currently receiving haemodialysis in the CKD stage 5 focus groups (Table 1) with patients who were female and who were receiving peritoneal dialysis patients being more difficult to recruit. The majority of focus group participants were Caucasian (72.4%), followed by South Asian (13.8%), Asian (10.3%), Pacific Islander (1.7%) or Hispanic (1.7%).

**Table 1:** Focus group participants’ characteristics.

Focus Group	Participants (n)	Age (median range) years	Male (Female)	Duration of Diabetes (mean, SD) years	KDOQI CKD stage	Duration of CKD (median, IQR) years	Non-dialysis (n)	Dialysis (n)	Haemo-dialysis (n)	Peritoneal Dialysis (n)
All	58	67 (48–84)	41 (17)	18.8 (10.3)	3 to 5	6.0 (3.0–10.0)	39	19	13	6
FG 1	5	68 (58–80)	3 (2)	16.7 (12.0)	3	8.0 (4.5–10.0)	5	-	-	-
FG 2	7	68 (62–84)	5 (2)	15.0 (7.3)	3	1.5 (1.5–5)	7	-	-	-
FG 3	5	72 (55–74)	3 (2)	14.4 (9.5)	3	9.5 (2.0–28.5)	5	-	-	-
FG 4	5	70 (64–84)	5 (0)	19.4 (7.2)	3	6.0 (6.0–13.0)	5	-	-	-
FG 5	4	63.5 (58–66)	2 (2)	16.0 (8.2)	4	3.5 (2.5–9.5)	4	-	-	-
FG 6	4	72 (67–77)	3 (1)	19.5 (8.0)	4	5.5 (1.0–11.0)	4	-	-	-
FG 7	3	66 (49–76)	2 (1)	18.7 (7.1)	4	6.0 (1.5–11.0)	3	-	-	-
FG 8	4	58.5 (48–78)	4 (0)	21.2 (22.6)	4	10.0 (10.0–10.0)	4	-	-	-
FG 9	6	63 (51–82)	5 (1)	16.8 (11.8)	5	3.5 (2.0–8.0)	0	6	3	3
FG 10	4	64.5 (58–74)	4 (0)	23.3 (7.9)	5	2.5 (2.0–6.0)	0	4	3	1
FG 11	6	63 (50–78)	3 (3)	26.2 (12.9)	5	6.0 (4.0–10.0)	1	5	5	0
FG 12	5	72 (53–79)	2 (3)	21 (8.3)	5	5.0 (4.0–7.0)	1	4	2	2

Eight semi-structured interviews of carers of patients with CKD 5 were conducted before data saturation from both focus groups and interviews was reached. Table 2 describes the characteristics of patients being cared for by the carers. Most (3) were aged 71–80 years, 2 were aged 61–70 years, and 2 were aged 41–50 years and 51–60 years respectively, while one participant refused to disclose his age.

**Table 2:** Characteristics of patients whose carers participated in semi-structured interviews.

	Frequency unless otherwise stated
Number of participants	8
Age (median, range)	66 (48–77)
Males (females)	5 (3)
Ethnicity	
Caucasian	5
Asian	2
South Asian	1
Type 2 diabetes (%)	100
Duration of diabetes (mean, SD) years	18.63 (10.21)
Duration of chronic kidney disease (median, IQR) years	5.5 (4.25–7.13)
Pre-dialysis	3
Dialysis	
Haemodialysis	3
Peritoneal dialysis	2

**Fig 1:**
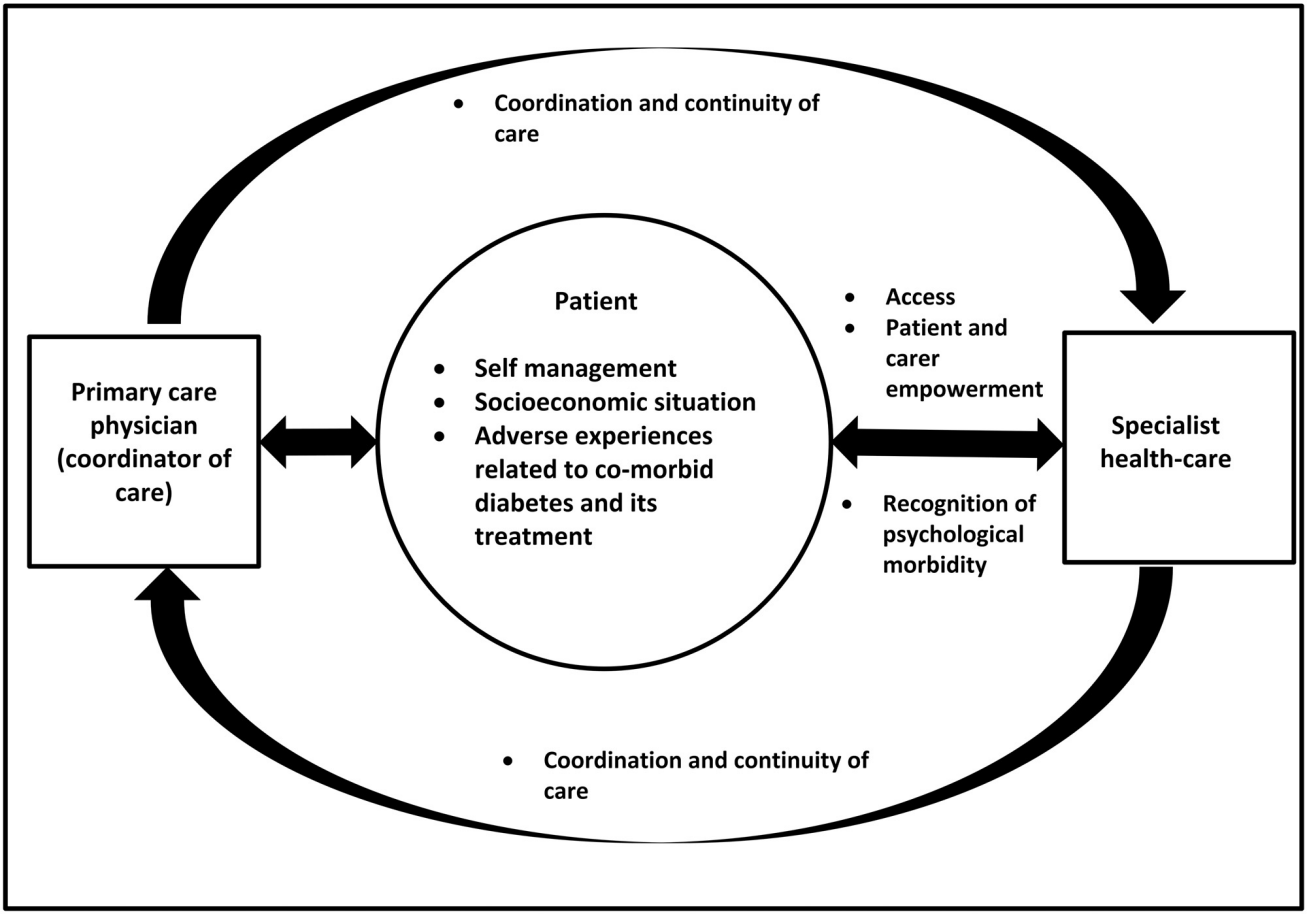
Thematic schema of patients’ and carers’ perspectives on health-care of co-morbid diabetes and CKD and major influencing factors.

Both patients and carers emphasised the central role patients played in their own health-care and identified 3 patient level (patient self-management, socio-economic situation and adverse experiences related to co-morbid diabetes and CKD and its treatment) and 5 health service level factors (patient and carer empowerment, access, poor coordination and continuity of care, poor recognition of psychological co- morbidity and prevention and awareness of co-morbid diabetes and CKD) that impacted on management of co-morbid diabetes and CKD (Table 3). While most themes were common across all stages of CKD, access and poor recognition of psychological co-morbidity were concerns that arose predominantly from focus groups with patients with CKD 5 and interviews of their carers, and poor coordination/continuity of care and patient empowerment were concerns that arose predominantly from focus groups with patients with CKD 4 and 5 (Table 3). Further variation in themes according to gender or mode of dialysis were not apparent. Fig 1 provides a thematic schema of patient’s perspectives on health-care of co-morbid diabetes and CKD and the role of each factor for optimal health-care.

**Table 3:** Summary of the main factors influencing health-care of co-morbid diabetes and CKD.

Sub-group of participants	Patient level factors	Health services level factors
**All**	1) Patient self-management. 2) Socio-economic situation. 3) Adverse experiences related to co-morbid diabetes and CKD and its treatment	1) Prevention and awareness of co-morbid diabetes and CKD.
**All (CKD 4 and 5 and carers > CKD 3)**		1) Poor coordination and continuity of care. 2) Patient and carer empowerment
**CKD 5 and carers**		1) Access. 2) Poor recognition of psychological co-morbidity

### Patient level factors influencing health-care of co-morbid diabetes and CKD

#### Patient self-management

Both patients and carers indicated that the responsibility was on patients to self-manage their co-morbid diabetes and CKD such as self-monitoring of blood glucose levels, adhering to diets or taking their glucose-lowering or anti-hypertensive medications. Patients reported the importance of motivation and ownership of their own health and recognised that doctors were resources to answer questions and provide management advice rather than being responsible for their health.

“(…) people are responsible for their own health and the one thing that seems to come across all the time is doctors are so busy trying to help people get better after they’ve got sick, but the people themselves so often do not make any effort for themselves”.

– Focus group (FG) 1

#### Socio-economic situation

The provision of emotional support was driven by family and friends, with their involvement central in facilitating adherence to treatment plans and dietary restrictions required for management of co-morbid diabetes and CKD. This was especially important for patients with advanced disabling complications of diabetes and CKD requiring a “carer” to be involved in day-to-day management.

“If it wasn’t for my wife keeping an eye on me, I think I’d probably slip off the straight and narrow more often than I do. She’s forever reminding me that you really don’t need to eat that, I think you’ve had enough to drink and etc. etc.”.

– FG 3

Conversely, patients and carers thought that being from a non-English speaking background or ethnic cultures characterised by apathy or which expressed hospitality through food, made education about the disease, judicious self-management and dietary restrictions difficult.

“[ethnic group] people have got an attitude ‘it will be okay. Nothing is a big problem.’”

– Semi-structured Interview (SSI) 6

Patients and carers acknowledged the influence of their financial status on disease management. Despite free access to medical advice and treatment, they reported additional expenses from transport to appointments, parking, medications, and maintaining a healthy lifestyle (healthy foods and gym membership). For patients with advanced disease, expenses from community services providing support for activities of daily living were noted.

#### Adverse experiences related to co-morbid diabetes and CKD, and its treatment

Optimal care was also influenced by the prior adverse experiences (morbidity) related to co-morbid diabetes and CKD and its treatment. Participants highlighted that morbidity due to diabetes and CKD, especially in advanced stages could hinder self-management. Tiredness, feeling unwell, increased disability and loss of independence all impacted upon treatment. These factors had the potential to negatively impact upon families, marriages, and social circles, leading to cases of divorces and lost friendships.

“You know, my wife says to me now, you know, we’ve lost a lot of friends because of my condition, because I’ve been moody or I get moody, you know. People don’t understand what you feel or what you’re going through”.

– FG 10

The demands of treatment, ranging from food restrictions to treatment side-effects (weight gain and hypoglycaemic episodes with insulin; lethargy and dizziness after haemodialysis or worsening glycaemic control due to the intraperitoneal carbohydrate load associated with peritoneal dialysis) and the time impositions of treatment (multiple appointments and dialysis episodes) were described as challenging for patients.

“It’s the impact of the haemodialysis, because I’ve just had my two days off in a row and I’m feeling pretty good today, but when I only have one day and the dialysis, I just feel terrible the whole time”.

– FG 11

The adverse experiences (morbidity) could have a negative psychological impact on patients. While some patients maintained a positive outlook others displayed poor coping strategies leading to frustration, denial and suboptimal management of their disease. Participants described how pre-existing psychiatric comorbidities such as depression made adjustment and management even more challenging.

### Health service level factors influencing health-care of co-morbid diabetes and CKD

Both patients and carers expressed general satisfaction and gratitude for health services. However, the following health service level factors were identified as potential pathways for improvement.

#### Prevention and awareness of co-morbid diabetes and CKD

Participants expressed the importance of increased prevention and awareness of co-morbid diabetes and CKD. Greater investment in public health campaigns promoting healthy eating and a healthy lifestyle were viewed as a primary method to prevent the onset and progression of diabetes and its complications.

“(…) educate people about the consequences of doing the incorrect thing… yes the general population”.

– FG 1

#### Patient and carer empowerment

Participant groups, particularly those with CKD 4 and 5, articulated the importance of empowering both patients and their carers to self-manage co-morbid diabetes and CKD. Greater access and uptake of education strategies to improve understanding of the nature, consequences and management of the conditions (including dietary choices) was highlighted. Empowerment through support groups and self-directed e-learning opportunities was valued, as the perception was that health professionals were time poor or did not offer explanations unless specifically asked.

“I have the Mayo Clinic sending me stuff virtually every week or second week because you can put in what fields you’re interested in, so I put in diabetes”.

– FG 7

Although participants were grateful to be educated by health professionals, the likelihood of ‘information overload’ or provision of irrelevant information was apparent.

“(…) look you get tonnes of paperwork. You get food pyramids and you get all of this stuff. And you’re just so overloaded that you just cannot absorb it”.

– SSI 5

Patients and their carers reported that they could be more empowered to manage co-morbid diabetes and CKD through improved education on specific topics including disease ownership, and involving more detailed explanations during medical consultations. Educational material could be simplified, and include more information on kidney disease (including early kidney disease and prevention of progression), specific dietary information for both diabetes and CKD (many patients received information for each condition separately and felt they could be contradictory), and practical information on self-management (such as accessing educational resources, social services, or dialysis when traveling). Education could be presented using peer support groups and opportunistically in clinic waiting rooms.

“I find that most people refuse to accept responsibility for their own health and doctors sometimes go along with that and are only too happy to have XYZ as a patient and treat the symptoms without educating the patient so strongly that the patient gets the message and wants to do something for themselves”.

– FG 1

#### Poor coordination and continuity of care

Most patients, especially those with CKD 4 and 5, and their carers, expressed that poor communication of medical information (test results, medications and medical histories) between hospital specialists and primary care physicians (PCPs) contributed to problems with coordination of care. Many patients felt that hospital specialists required constant reminders to communicate test results to their PCPs and that lack of communication lead to duplication of tests.

“(…) but if the main results from this hospital were sent to your PCP on the one system, everybody’s happy, but they’re costing the system a fortune, because everybody’s doubling up”.

– FG 1

Coordination problems regarding appointment times and provision of medical advice were reported, especially if patients had multiple comorbidities requiring input from multiple specialties. Segregation of medical advice according to specialties was an issue to patients, as they perceived that certain health professionals were unwilling to offer advice or help manage problems that didn’t fall within their specialty.

“Oh it’s got nothing to do with us, you’ve got to see your PCP”.

– SSI 5

A perceived lack of continuity of care in outpatient specialist diabetes and kidney clinics was apparent. Patients reported seeing different doctors, leading to different and conflicting medical opinions and plans, or even different medications being prescribed, leaving patients frustrated or confused.

Patients and carers expressed that coordination of care could be improved through mandatory communication of medical information and investigation results and a shared medical record between all involved health professionals could improve communication and coordination of care especially between tertiary and primary care. Further improvements could involve a combined multidisciplinary diabetes and kidney service with allied health input such as social workers, podiatrists, dieticians, pharmacists and nurse educators. This could improve coordination of health-care and communication, and decrease the need for multiple appointments and the possibility of appointment time clashes.

“I think (…) they would all know what is going on, it would be better coordinated”

[speaking about a combined diabetes and kidney service]–SSI 1

Patients also felt that there should be a designated health-professional coordinating all their medical care, usually the PCP. They commented that continuity of care could be improved by each patient seeing the same limited number of health professionals each visit.

#### Access

Problems with access were another barrier reported by patients with diabetes and co-morbid CKD stage 5 and their carers. Long waiting room times (up to 3 hours) at diabetes or kidney appointments and a lack of close, free parking were expressed as the greatest barrier, especially if the patient had disability as a complication of co-morbid diabetes and CKD.

“Parking. That is always difficult and it’s a long walk too for him, like he has got toes missing”.

– SSI 1

Some patients also felt that doctors did not spend adequate time in the consultation. Short consultation periods and long waiting times were attributed to a lack of doctors.

“I found as far as waiting times go, I come here prepared to allow for at least two hours”.

– FG 5

Others complained about difficulty contacting outpatient services to change appointments, the timing of available appointments (which often clashed with working or dialysis times), and the lack of availability of interpreters. The accessibility of medications and treatment was perceived as challenging due to the costs.

Patients and their carers reported that access could be improved by decentralising services to community clinics (decreasing travel times, improving parking and decreasing walking distances to the clinic); improving parking; greater use of outreach nurses making home visits, or transport services; reducing waiting times for medical specialists clinics by increasing medical staff; and a greater choice of days for appointment times (clinics not being held on the same day each week).

“Oh, cheaper parking. Most definitely cheaper parking”.

– SSI 5

#### Poor recognition of psychological co-morbidity

Patients and carers of patients with diabetes and co-morbid CKD 5 expressed concern about the lack of routine screening for psychological co-morbidity in patients (especially with advanced complications of diabetes or CKD). Psychological services were perceived to not be routinely available or costly to access in the community.

“People don’t seem to realise the needs of somebody that’s, if you were locked in this room day after day after day, month after month, it has a psychological effect on you. You mentioned psychiatrists, I went to one, or was sent to one, and the charge for just an hour was $500”.

– FG 12

Participants said that this could be improved by education about the possible psychological sequelae of the disease and routine screening for psychological comorbidity.

“The ward, or the PD department, ought to have a psychiatrist that is aware of the problems you are having in this sort of environment so that you can come and talk to them, and they understand what you are talking about. Just, just even bump you up”.

– FG 12

## Discussion

In this large multi-site qualitative study, we inform the development of person-centred health systems by exploring important factors relating to the health-care of co-morbid diabetes and CKD, from the perspectives of patients and their carers. Key patient level factors were patient self-management, socio-economic situation, and adverse experiences related to co-morbid diabetes and CKD and its treatment. Key health service level factors were prevention and awareness of co-morbid diabetes and CKD, poor continuity and coordination of care, patient and carer empowerment, access and poor recognition of psychological co-morbidity. Health-service level factors varied according to CKD stage with poor continuity and coordination of care, patient and carer empowerment, access and recognition of psychological co-morbidity emphasised by participants with later compared to earlier stages of CKD.

Previous qualitative studies amongst patients with co-morbid diabetes and CKD have not explored barriers and enablers to optimal health-care across CKD stages 3 to 5. One qualitative study explored self-management in patients with co-morbid diabetes and CKD stage 3 [12]. Other qualitative studies have explored either patients’ experiences on being referred to a renal specialist clinic [13, 14], a specific aspect of health-care—medication adherence [15, 16]–or patients’ feelings and their process of psychological adjustment to co-morbid diabetes and CKD [23, 24]. Our findings add to previous studies by describing the key determinants to achieving a patient-centred health service for management of co-morbid diabetes and CKD.

Self-management and patient and carer empowerment was identified as an important factor for optimal health-care across all CKD stages in patients with co-morbid diabetes and CKD. This was emphasised more by participants with CKD stage 4 and 5 compared to stage 3 and may be explained by those with more advanced CKD having greater awareness of the seriousness of their condition, or greater need for empowerment in the face of more demanding and complicated disease management. Evidence concerning the beneficial effects of greater empowerment through education programs is inconclusive. While a Cochrane systematic review studying the effects of education programs in diabetes and CKD concluded that the evidence was poor [25], more recent studies have reported that education programs improve self-management, glycaemic control, and prevent kidney function decline [26, 27]. Nevertheless, given studies showing the importance of patient empowerment and self-management in diabetes [28, 29] or in CKD [30, 31] and the emphasis that patients and their carers placed on the patient self-management and empowerment in our study, both factors should be considered central in a person-centred health system for co-morbid diabetes and CKD.

Participants with co-morbid diabetes and CKD emphasised poor continuity and coordination of care as a barrier. Participants suggested improving health-care by increasing coordination of care through better primary and specialist care communication, a shared medical record and a combined diabetes and kidney service. Greater coordination between primary and specialist care for diabetes through a shared care model has been shown to improve HbA1c and other quality indicators such as referrals for eye examinations [32]. One systematic review exploring interventions to improve diabetes management in community settings found that changes in medical record systems and postgraduate education in combination with other professional interventions (such as audit and feedback) improved process outcomes [33]. With respect to combined specialty clinics, observational studies in the United Kingdom have reported slowing of kidney function decline and improvement of target attainment, including HbA1c [34–37]. The exact reasons why these combined services achieve these benefits has not been explored. Our findings suggest that contributing factors may include improved communication, understanding of roles and coordination of care; as well as greater convenience for patients. Together, this work suggests that there are clear strategies to drive improvements in health services to not only make them more patient-centred but to improve outcomes.

Access problems were an issue mainly emerging from focus groups of patients with diabetes and CKD stage 5. This may be related to the degree of disability, frailty and multi-morbidity that may occur as the duration of diabetes and CKD stage increases. When patients are unwell, long waiting times in clinic and long distances to walk from car to clinic are likely to matter even more. One solution to improve access, offered by participants, was decentralising clinics from the hospital to the community. Similarly, a Cochrane systematic review [38] of such clinics has reported improvements in access. Our study, which is specific to co-morbid diabetes and CKD, explores this theme further highlighting that potential benefits may include improved access with decreased travel times, improved parking, and shorter distances to walk from car to clinic, which is an issue for patients disabled by diabetes and CKD.

Participants with CKD stage 5 described the psychological consequences, reactions, and adjustment to having diabetes and CKD and the need for routine psychological assessment and management. This is consistent with previous descriptions of development of fear, anxiety and self-pity in patients with stage 5 CKD needing renal replacement therapy [23]. Psychological morbidity, especially depression and anxiety, is common in patients with stage 5 CKD (rates of depression and anxiety may be up to 80.5% in dialysis units and above 30% among pre-dialysis patients [39, 40]) and associated with poorer quality of life [39, 40]. One longitudinal study found 22.1% of the cohort with co-morbid diabetes and stage 5 CKD to have depression and this was associated with increased mortality [41]. Furthermore, a qualitative study exploring psychological adjustment to diabetic kidney disease also concluded that there was a need for routine psychological assessment [24].

The qualitative approach adopted in this study, enabled a detailed examination of perceptions, ideas and expectations compared to that offered by a quantitative approach. The robust qualitative study methodology including separate focus groups for different CKD stages, triangulation of results with semi-structured interviews with carers, analysis and coding of data by 2 investigators, and the use of reflexive journaling for verification of findings, was a strength. The sampling across different geographical locations and of a broad cross-section of participants with differing severity of disease increases the transferability of results. By contrast, the inclusion of English speaking participants from an Australian health-care system, the lack of rural participants and the relative lack of participants who were female and who were receiving peritoneal dialysis may limit the transferability of results to other populations or non-English speaking health-care settings. However, the relative lack of female and/or peritoneal dialysis participants in the sample may be a reflection of the general CKD population in that males have a slightly higher prevalence of CKD compared to females and haemodialysis is the predominant dialysis modality for adult patients [42, 43]. The inherent weaknesses of qualitative research including the potential for researcher and participant bias and lack of large-scale generalisability of results, is also acknowledged.

Our qualitative study explored important factors and barriers to the optimal health-care of patients with multi-morbidity due to diabetes and CKD, across CKD stages 3 to 5. All patients and their carers emphasised the importance of patient empowerment and self-management, and public awareness of diabetes and CKD and its prevention, and identified adverse experiences related to co-morbid diabetes as a barrier. Barriers to health-care such as poor access, poor continuity and coordination of care and poor identification of psychological morbidity were emphasised more in participants of later CKD stages compared to earlier stages. A preventive, patient-centred health-care model, promoting self-management and targeted interventions, especially in later CKD stages, to improve health-care access, continuity and coordination of care, and recognition and management of psychological morbidity may improve health-care delivery. Our data on patients and carers’ perspectives may prove as important for the re-design and delivery of patient-centred health services for co-morbid diabetes and CKD as intervention studies.

## Supporting Information

S1 File Transcripts. (DOCX)

S1 Table Initial Questions inventory for focus groups and semi-structured interviews. (DOCX)
