# Supplementary material for: The Perspectives of Patients on Health-Care for Co-Morbid Diabetes and Chronic Kidney Disease: A Qualitative Study
Source: PLoS One. 2016 Jan 5;11(1):e0146615. doi: 10.1371/journal.pone.0146615 (PMC4701448; doi:10.1371/journal.pone.0146615)
Supplement: S1 File — (DOCX) [file pone.0146615.s001.docx]

**Supporting Material 2: Transcripts**

Please note that all participants’ names and mentioned doctors and hospitals have been changed to ensure they remained de-identified.

**Focus Group 1**

**Q: So, look the first question for discussion is, I'd you'd to think about your experiences that you have of diabetes and chronic kidney disease. Now based on your experiences, what would you say are some of the strengths of some of the health services here at Hospital 1 for your diabetes and chronic kidney disease? Now I know some of you guys see people outside from Hospital 1, but I know some of you guys will obviously have some interaction with Hospital 1, otherwise you wouldn't have been on the database here. So, what are some of the strengths of the health services here at Hospital 1, especially for the diabetes and the chronic kidney disease?**

John: I've only been to…

**Q: Oh sorry, can I also say before you have a comment, can you just say your name's John, just for the recording, so we know.**

John: My name's John and I've been coming to this hospital now for four-and-half years, since the accident I had. And my first impressions when I come here was, doctors would come up to me and they would know me, but I didn't know them, because when they chopped me, when I was in here I was in an induced coma. But, I've only had one session here with diabetes and now I transfer my information to the diabetes staff which is up on the second floor of the <inaudible> *0:01:19.7 and through e-mail they communicate with me and they finally put me on SoloSTAR. Originally I was just on a few tablets, but I think I'm getting going down a little bit and they're not doing their job anymore, the tablets I'm on. The impression I get with the hospital, the staff are very good, but the computer systems are actually crap. When you get a letter and it says a 2:30 appointment and they don't see you till 4:00 O'clock.

**Q: So, the computer system as in booking is it?**

John: And then when you come into the hospital and you go to one counter and tell them that you're there and then the doctor can't look at the same screen and have them input the times and it's just how your file's put on top of another one and some people come in after half an hour after you and they're seen to you two hours before. I think it's a very bad system, when you've got the staff at one end taking in who's come in and the doctor at the other is looking at another computer system and can't see who's come in at what time. That's my goods and bads.

**Q: Anyone else got any other comments? Look, maybe since we've started on a bad, why don't we just continue on the bad, then we'll turn to the good, so what are some of the bad things about the health services here at Hospital 1?**

Rob: I'm Rob and the problem I have is that in all the years that I've been coming here, which is six or seven, I don't think I've seen the same doctor three consecutive visits. They're all digging up things out of the system; they all want to try their own little hobby horse. None of them seem to take much notice of the fact of what I'm doing or what they're doing to me doesn't work and none of them seem prepared to take any radical choices to see whether something else may work.

**Q: Is this for the diabetes or the kidney disease or both?**

Rob: For the diabetes. My kidney disease is pretty well stable in so far as my only real problem with the kidneys is my creatinine levels and they can vary as much as 50 points from one visit to the next. But other than that, as I say, on the diabetes front my average is about 15 millimoles daily and no matter what I tell them, no matter what I suggest, they always take me back to the same boring basics which just don't cut it. So, I can't help thinking if they got my diabetes under control then maybe my renal functions would also improve, but it doesn't seem to happen.

**Q: So, your main criticism is you're not seeing the same doctor?**

Rob: That's the first part, yes.

**Q: And what was the second part? Sorry just to…**

Rob: They're conservative in their treatments.

**Q: Sorry John what was that you wanted?**

John: My name's John, can I just butt in?

**Q: Yeah of course.**

John: I agree with the doctor, where you don't see the same doctor every time and I think that's where a lot of the time is spent upstairs where I've got a file I know since my accident, like that and a different doctor each time's got to read down and see what the other doctor's said. And yeah you don't get the same sort of consistent communication.

Rob: And this one will describe one thing. This doctor will take you off what the previous doctor put you on and put you on this other product and then the next time you come back you see a different doctor and he takes you off that second doctor's prescription and puts you back on the first doctor's prescription. But, they don't seem to accept that neither of the products that you've been taking works and offer you a choice of something else; it doesn't happen.

**Q: Anyone got any other comments about weaknesses?**

John: I've got one more.

**Q: John, yeah I was going to say, don't <inaudible> *0:05:56.0 again.**

John: I put myself onto the Australian government myGov where all of your medical records can be looked at from everyone and I've asked here many times, why can't my records be put on the same system so I can log in there or go to any doctor, give them my code and they can look it up on their computer; why? They're the ones that's available at the moment on the system, I don't know if you're aware of it.

**Q: Yes, no uptake hasn't been great.**

Rob: But that's a federal government option and it has a lot of problems already, that system.

**Q: Rob, why do you say that or..?**

Rob: Because, ever since it was originally started, there were problems with it and there are opposing forces working with it. One lot wants to give everybody access and another lot want to keep it all severely private in confidence. Now if you have a medical history, then it should be readily accessible by any registered medical practitioner, but it isn't. They've got to jump through all sorts of hoops in order to get into it, which is so frustrating, am I right John?

John: And the other thing too is when you log on. Being a government…

Rob: Website.

John: Yeah website, you would think that web browsers of any sort would be able to access it. I use the latest computers, I always have; it was my job building computers and the web browser will even at the moment, if you log on with it will just log you off. It's just not up to date and for a government access to be like that, it's pretty bad.

**Q: Thanks for that John and Rob, anyone got any other comments? Merle I think you wanted to say something?**

Merle: Merle.

**Q: Merle, sorry.**

Merle: One of the things that I found was that the doctors for that particular clinic, some of them didn't turn up on that day and that left a real backlog of people sitting waiting.

**Q: Sorry was this for the kidney or for the diabetes?**

Merle: Yes.

**Q: Kidneys?**

Merle: Yes and I don't know many doctors were supposed to come to the clinic, but some of them didn't, so that meant that everybody was just sitting waiting for hours.

**Q: But does that often or..?**

Merle: It only happened once that I was here. On other occasions I've been in out and very quickly, so that might've been a bad occasion, they might've been called out to something else, but the staff didn't seem to know that they weren't turning up.

**Q: Any other weaknesses that people want to mention?**

Rose May: I might just say because my…

**Q: Rose May, yes.**

Rose May: Yes my name is Rose May. I am trying to know because my husband, he got diabetes and he got amputation of the toes and when he said he had diabetes…

**Q: So you get amputation of the toes?**

Rose May: Not me; my husband, <phone ringing> but I would like to know, because I get diabetes. If something happened to me, the same thing and my kidneys are no good, I would like to know if that will happen. But, my diabetes is nine, sometimes 11, but my kidney for a long time I had kidney problem that is not worsen, but every time the doctor called me for blood test and urine test, but in between mine is sometimes six months, but I respond to their call, because yes. But, another thing, I have become old, I am not become young, I have to expect something worse, not better; you know what I mean? Yes, because I feel very grateful to the doctors, but I *0:10:31.5 just, I want to know my husband had diabetes, very severe diabetes; 22, 24 very possibly. But, that I'd like to know, if that's worsening, because for me it's nine, 11 and every time I see my family doctor I like to prepare myself for the urine tests and blood tests, everything.

**Q: What about the services here Rose May? I think you see the kidney doctors here. How do you find the services? Any weaknesses or things that you find frustrating here?**

Rose May: No, there'd be no problem, I come but like I say, you know they are ill and not only me; there are a lot of patients you know. But, I don't know, because it could be the gentlemen in the beginning they were sick before me, you know what I mean. But, if they can improve and get more doctors and can look after us, we'll be very fine.

**Q: So, did you say you were waiting a while? Was that why you wanted more doctors or..?**

Rose May: No, but when they tell me I wait sometimes, that's happened to me and I was waiting, waiting and waiting, but when I wait, I was waiting and often say the doctor is going for another thing, but you have to go. And I pay taxi in myself, I see the government is, you know they are going to pay taxi for me. And when I get my pension card, I pay $3-something for the train, but I get in the taxi and go to [Gretham] *0:12:15.8, yes I take the taxi and go to Gretham. I free, instead I take the car, not the car but the train. You know, I explain well, because my accent is different, I speak French you know.

**Q: Nice French accent, yes.**

Rose May: Yeah, thank you.

**Q: So, you're saying that sometimes the waiting times are long?**

Rose May: Not all the time.

**Q: But sometimes?**

Rose May: Only one times, two times, but what we can do, but could be the gentlemen, but today I don't know how long they were sick and how long they were waiting and this lady too. But, I don't know, I just say what it…

**Q: So, any other weaknesses people want to mention about the services here?**

Rob: Yes, the fact that they keep insisting on giving letters. You say, "No, I've got it already written down." No, they still send you out a letter. I just don't see the point and then if you come in…

**Q: Is this for an appointment time?**

Rob: For appointments, yes. If you come in and you quote your number, they go, "Oh no, what's your name please love?" And then another time they want your number, but whichever one you offer, it's not the one they want.

**Q: Any other criticisms? Frank have you got?**

Frank: Well, I've never been here; I've always been in the consultant rooms.

**Q: Any other comments anyone?**

John: A lot of the times when I come in and it might be a month or a two-month visit and it's shorter or longer and longer now, the times that I come in, because everything's been, since my accident it was very hard to stabilise and now they've got me down on track. But, when I come in, I come in a week earlier and give a urine and a blood sample for the test. That's great, but surely if there's nothing wrong and you wait upstairs for three hours, surely they could've phoned me up and said, "No need to come in, your blood tests and everything else is fine." Why sit there for a doctor; his time is valuable. A letter could've been sent then. Why sit there with 200 people when it's not necessary? If it's important, then the time that is important to people could be given to them. I still can't understand it. I've got another one this afternoon, as you know. It'll be the same.

**Q: Look, I mean I think that the way the doctors, speaking from a doctor's perspective, that the way that they evaluate you that the tests are just one way. Obviously they put everything together. I mean, obviously they'll probably have a look at sugars at home and that as well for the diabetes.**

John: For diabetes yeah, but for renal. I'm talking now as also with renal, not just diabetes, right with the renal health. You go and you have a urine test, I give a urine test.

**Q: And I think that there's other things that they look at, like the blood pressure and things like that as well.**

John: Yeah, but that's what I'm saying, if this was all in one. I go to my own doctor once a month and I also have to have one of them injections for my blood content, you know like they give the motorbike guys or the guys that run on pushbikes. I have to have one of them once a month to bring my blood…

**Q: Blood count up?**

John: Blood count up, right. Now I have that done at my local doctor. Now if that was all on the same computer system, how much time would it save?

**Q: Thanks for that John.**

Rose May: You know my husband was here, it will be 16 years ago. He was sick all the time. Now all the time he was there, but I can't say, look that's happened to me about one time, two time, but for my husband who was sick, I bring him because I don't drive, but I talk more, because I talk too much, I talk more than him, because he was looked like when he's sick, he's a bit shy. He's a man but he never talk, I have to talk for him. And they look after him very well, I can't say anything about, because he died in this hospital and one day-- because when you get diabetes and I think the bladder was collapsed and they have to put a tube in at the night time; in the morning I have to pick him up. When I come, I saw a lot of tube with him and blood with him and the doctor said to me, "He's got cancer in the bladder." I didn't expect because a lot of time he came here and they never said that he had a bladder problem. But, that's how, just turned like that, you get cancer in the bladder, but he died with this cancer in the bladder, but they worked then amputation of the toes, everything for the diabetes.

**Q: Thanks for that.**

Rose May: Thank you.

**Q: Let's move on, so we mentioned a lot about the weaknesses and frustrating aspects, what about the strengths? So, how's your connection with Hospital 1 Frank?**

Frank: Well with Dr AF she, because of my kidney disease, I was asked to come in one day with the prospect that I may have to go on dialysis and somebody spoke to me about that and informed what happens and what goes on. So, I found that useful; if it comes to that, I know what to expect.

**Q: Let's talk about the strengths now of the health services here at Hospital 1, what are some of the strengths you'd want to mention?**

Rob: Well, for me it's the fact that I can come to the one place and have all of my problems seen to in one go. Okay, I go to my GP when I need to, when I'm feeling sick. But, for my long-term, ongoing chronic medical problems, which don't really require a lot of input from a medical practitioner, I come here and they have a constant record going and if something changes, they're very quick at saying, "Right, come in a bit more often." And I have to admit I feel more confident about the stream of information that's available to medical practitioners in Hospital 1, as compared to what my GP gets.

**Q: When you say stream of information, what do you mean?**

Rob: Well in terms of…

**Q: Medical records or..?**

Rob: Well not just medical records, but interaction between doctors. There's a lot more of them in any given discipline and you find that if one doctor has a problem so to speak, he will discuss it with his colleagues in the same department and might get three or four different leads to go on with. Whereas a GP is a lot more isolated and quite often is so busy, he doesn't have the time to do the research to keep up to date.

**Q: You said it was good to have everything at the same time at the same place.**

Rob: Well yes.

**Q: Is everything always at the same time?**

Rob: No, no, not at the same time.

**Q: But at the same place you mean?**

Rob: But at the same place and the one medical record and they are aware of the different problems and they all seem to, how do I put it? Take it into consideration. If I'm talking to someone in renal about my kidney function, then they're aware of my diabetes and they understand also the medication I'm taking for my heart condition. And when I was having a lot of trouble with foot ulcers; that was all being taken into consideration at the same time. So, to me it's much more holistic.

**Q: Anyone else in terms of strengths about the health services here?**

John: I reckon that the services here, the doctors do know their job and essentially without them being here, I wouldn't be here today, I've got to say that. And the strength of the hospital is the doctors, but I think a lot of information, giving it to them could be sorted out better in their attempt, that they could even give you a better time.

**Q: Can you elaborate further when you say that information could be sorted? What do you mean by that?**

John: Well, for instance when I go upstairs this afternoon, I have to communicate that my local doctors is such and such and I have to get the records that I have here. Every time I come here, I have to tell them each time to send that information to my local doctor, so that my records are all up to date, so he knows what's going on, because I go and see him once a month as well. Now, the doctor, he's done his job, he shouldn't have to do that. That's more time that he's wasting, because of the process and I'll go back to it, the computer system, but the strength is the doctors are willing and know how to do it for you.

**Q: So, what you're saying is that the transfer of medical information isn't great?**

John: Yeah, yeah.

**Q: Does anyone else find that an issue?**

Rob: Yes.

**Q: Rob yeah?**

Rob: I find that every time I come in for a blood test, because if I'm a diabetes appointment or a renal appointment, I always have the slip beforehand and come in, like John does and do my blood and my urine sample a few days before my appointment. But, if I don't specifically ask every time for a copy of those results to go to my GP, it doesn't happen. It's like it's all being hoarded, "This is mine. We don't want anybody else knowing about it. No, no, no, just for us."

John: And the process of that is when I go to my local doctor, he wants to do the same thing. He said, "When was the last time you had this done?" And I said, "You should've got the results."

Rob: Yes, but he's going through one pathology lab and the hospital is going through a different pathology lab and they don't talk to each other.

John: No, but if the main results from this hospital were sent to your GP on the one system, everybody's happy, but they're costing the system a fortune, because everybody's doubling up.

Rob: I know, I know.

Merle: I have been to a number of clinics here at Hospital 1 and if I have to have any other treatment then I always ask please to come to Hospital 1. My GP sometimes wants to send me to her favourite and I say, "No, I want a referral to Hospital 1. I've got great confidence in Hospital 1."

Rose May: Yes, that's right.

Merle: So, I don't have any troubles. I'm old; I've lost my train of thought. Yes, I don't see the renal doctor here anymore, because he went into private practice and because-- this is like a big story because I live close to where he is and I was seeing him at Hospital 1b and now he's gone into a private room, so he's a bit cunning, because he gets more money out of me. But, I sometimes wish that I could see the doctor about my diabetes and the renal doctor on the same day, so that…

**Q: So she looks after your diabetes..?**

Merle: My GP, but I would be happy to…

**Q: Did you see a specialist for your diabetes sorry?**

Merle: No, I would be happy to do that, but because I'm having the same tests. Like my GP will send me for tests and then if I'm seeing the renal doctor the next week, he's sending for me virtually the same tests. I'm having the same things and I'm not sure how you would work it, but it would be good if I could see both doctors on the same day, with the one lot of tests.

**Q: So is there issues of transfer of information between doctors?**

Merle: Well my GP gives me the results of my blood tests to take my renal doctor, but he usually just throws them on the floor and says I've got that, which means he's got his results from the tests I've had for him, so he's not really interested in what the GP has done, because they're virtually the same.

**Q: But, does your renal doctor communicate well with your GP, do you think?**

Merle: I think so, I think so, I'm not really sure about that.

**Q: Frank, like obviously all your specialists are in the private system, are there similar issues? I mean if you're in the public system it sounds like there's issues of transfer of information.**

Frank: No, look I don't have any issues. All the doctors that I see, I get excellent service. I've really got no problems with any of them. Dr Amy Fah in particular with the kidney situation, she's really, really concerned about my situation and she's always hammering me about improving and stabilising, so I've got no problems. My cardiologist, same sort of scenario and they all communicate with my GP. Every time I go there he'll say, "Right I've received a letter." He'll say to me, "Oh well hang on a minute." He looks at his screen and okay, okay, you went and saw so and so and you saw so and so and everything's good there and the same with the diabetics." So, I really have no problem, but just quickly saying my father's in a public system and I take him along to his visits and it sounds like everybody says here, but what I find mostly is the timing, although he seems to see the same doctor all the time.

**Q: When you say issue of timing, what do you mean by that?**

Frank: Well you have to wait considerably. If you have a 10:00 O'clock appointment it's-- look I went last week and I think I got home about 3:00 O'clock, so.

Rob: Try the eye and ear hospital.

Frank: Yeah I've done that as well, so. But, personally I don't have a problem. This is probably a little bit off the track, but I had a nasty tumour removed on my heel. I had it removed 16 years ago and then it started to regrow and I had a situation where I was told by the GP, "If it's not giving you any trouble; don't worry about it." And I felt let down there because it's become a nasty situation. There was talk about cutting my foot off and the start of my leg and stuff like this, so I'm not happy about that.

**Q: Any other comments about strengths and weaknesses of the health services here at Hospital 1 for the diabetes and kidney disease before we move on?**

Rose May: Yes, but I get these from my doctor the other day, you know who knows me, it was Monday, but for the test, like for the blood test and urine test.

**Q: Any other comments anyone, before we move on? Strengths or weaknesses of the diabetes and kidney disease services here at Hospital 1 or have we exhausted? I think no-one's got anything further to say, okay.**

Frank: All I can say is most doctors that I've experienced, I've been in a couple of emergency situations and they all seem to try their best. And a lot of the times it's depending on why you're there, they kind of work on a bit of a guess work, but a lot of them really are trying to do the best they can, so.

**Q: How easy, especially for those of you who are seen here, how easy is it for you to get your diabetes and kidney disease health care in terms of locality, affordability and waiting times? Waiting times, I think we've spoken about already. What about affordability and location? How easy is it to get the diabetes and kidney disease health care that you need?**

Frank: In regards to affordability I think it's a huge rip off. You know, I see private people there and I might be there ten minutes, 15 minutes and I got $140 bill. And I think that's a bit ludicrous where all I get done is basically I take in my readings, he has readings from a pathology report and he might take my blood pressure; he might not. He just asks me a few simple questions, how I've been going and all the rest of it and I think that's outrageous, because in my field there's no way known that I could charge $140. I understand that the profession of a doctor is a little bit different than other people, but I feel that, I just had this operation in December and the surgeon I saw, they did warn me that I could be out of pocket, even though I got high cover of $1,500. But, when it's finished, I get a bill of $2,250. So, I haven't worked for six years, I haven't had an income for six years, so it becomes quite difficult. And I said, "Well I can't pay this now, I'll have to do it in sessions." And I said, "But I worked on $1,500 not $2,250." "Oh, but you know you had to have the skin graft." "Well it'd be good if you'd told me in advance. So, the costs of certain things for the amount of time that you're there is just, the affordability's not right.

**Q: Merle are you..?**

Merle: Yeah Merle. I find coming to Hospital 1 very convenient for me. I use the car park, which I always find very good and it's not expensive and it's convenient and yeah I don't have any worries about that. So, I would always choose to come to Hospital 1 above anywhere else.

**Q: Do you want to comment on the costs?**

Merle: On cost?

**Q: Yeah, so kind of a sense of the public versus the private since you've done a bit of both.**

Merle: Well yeah. Well as the same thing as Frank. I mean, I go to my oncologist in a private place and now I've been caught up with the renal doctor privately. I could probably rectify that by coming back in here, but again it's convenient to go locally, but the cost is a bit prohibitive and when you think about what you get back from Medicare and everything like that, but my oncologist has been very good and I just have a lot of faith in him, so I just put up with that expense.

Frank: In regards to sometimes there are certain doctors that their focus is the monetary value and what they can extract from you and I think that applies in general, but there's some doctors who their main concern is treating your health and their monetary return is secondary. And I feel those people there, they are really doing their job correctly because a person who puts the monetary value and I feel as a medical practitioner that's how it should be, that's part of their course should be trained in that manner. But, there are some guys who even though they're medical practitioners they are sharks when it comes to the finance and I feel that's a letdown overall, because a lot of people tend to tar everybody with the same brush and I think that's a major negative in regards to the medical.

Merle: I would have to say that we as a family have probably had thousands and thousands of dollars-worth of treatment here at Hospital 1 for nothing and we're very grateful for that and that's why I choose always to come here when my GP says, "I will send you so" and I say, "No, no, no, I want a referral to Hospital 1 please" and do it that way.

**Q: Anyone else got any comments?**

Rose May: I'm going to make you laughm I said to the doctor yesterday, I say when doctor was talking with me and I say, "Doctor, here I know where to go." But, today I didn't know where to go, because I missed the place and I say, "If I get a boy, I will call him Sof. If I get a girl, I will call the baby girl Sophia," because I am too long here. You know my husband, my daughter, myself, we all love this hospital; that's why I make a joke of this one, if I get it and the girl.

**Q: Anyone else got any other comments, how accessible things are?**

John: I really enjoy coming to this hospital. My local doctor, like I said before, I have to have a monthly injection and I have to keep it in my fridge and the TAC pays for it, because I went through a bad accident. And it was funny because I came in here and I go to the-- where you get all your medication from.

Rob: Pharmacy.

John: And I get four months of injections and it was five bucks. That's if I want to pay for it myself or I can say TAC. So, my local doctor said, "Oh you don't have to go in there for that, going there all the while. I'll order them across the road from the cheap chemist." They were $260 a needle. Like I know where I come for it and I know which ones I give back. So, when you come in here and go to the service, I don't why it's different outside for the injection than what it is here, but the service here is excellent, right down.

Rob: The reason is the doctors here can get exemptions on the prescriptions. When they write you a prescription…

John: But it costs me nothing anyway.

Rob: …they put a particular code.

John: It costs me nothing anyway.

Rob: Yeah, but the chemist you were saying was going to charge you for them.

John: Yeah, yeah but it should cost me nothing, because it's through the TAC, but they wouldn't accept it.

**Q: Any other comments about how easy, how accessible are services in terms of cost, location, waiting times?**

John: Well all I have to do is hop in a taxi and come in and like and then when I want to go home, I call a taxi and then I go home. It's no good me trying to park a car or trying to get a wheelchair.

**Q: Is that affordable? I mean is that affordable with a taxi all the time?**

John: Oh yes it is, because in my accident the truck run over my shoulders and all the muscles up here-- before I used to be able to lift up a box, 180 pound on my shoulder, but now two plates, the muscles here I just can't do it anymore. So, to me I can't even put a wheelchair in a car, alright. I've got to have a, if I'm having a heavy loader put into my ute. That's a device that sits in the back of a station wagon, where you press a button and it brings it round to the driver's side, you can get in and you press a button and it goes back again when you finish. But, the hospital here for me is next to none. With the time waiting, it drives you nuts. Sometimes I go through five chess games sitting upstairs waiting. I forget what I'm here for half the time.

**Q: So, Rob.**

Rob: I find for me it's convenient. I can nearly always find a parking spot. Rather than go into the underground car park, I will park over near the big park, where there's nearly always one or two spots vacant. If you're prepared to walk the extra bit, like I do, right up by the primary school, which is a good kilometres walk, there's nearly always a disabled parking spot there. So, alright I walk, the exercise is good for me anyway, but when I get here, no matter where I go, after a while they all know me, I just sit and wait like the rest of us. You come in here and you don't pay anything, so where's the grizzle you know, you can just take your turn. The only thing is, as I say, is I would prefer to see the same doctor on consecutive visits, but as I've found, sometimes a doctor will be working here for instance as registrar this year and next year he's gone, gone to a different hospital. So, then you get a different doctor, but other than that the service is consistent and I don't live that far away, so for me fine.

**Q: What impact has attending the health services here at Hospital 1 made to your diabetes and chronic kidney disease? What impact?**

John: Well for me, it means that I'm getting the right medication for my illness and it means a lot and they've changed it as I've gone, to get me stable they've changed it from when I first started to now, so I know it's not the best, but the situation I'm in, but it's better than I get anywhere else, even my local doctor. After I've been here and then I hear some of his comments, I wouldn't rely on him really for my diabetic medication. I don't think he's right up to scratch for that, but when the results come through from Hospital 1 and they are on his system, he agrees with them.

**Q: Any other comments anyone wants to make?**

Merle: I think it's perhaps given me a little bit more confidence in my GP, in that my renal doctor doesn't change my medication, takes note of any changes that the GP has made and tends to agree with it. So, it sort of gives me a bit of extra confidence in my GP, because I think well she obviously knows what she's talking about.

Rose May: I don't want to put anybody down, because I just think about my husband when he got diabetes, but for me, he smoke, my husband used to smoke, but not too much. But, one day, he always wake up early and the doctor tell him he stop smoking and when I get *0:42:18.5, I wake up and I saw him smoke and I didn't tell him anything. I said, "You are smoking." He said, "No, just a puff." I say, "Okay." And the doctor has to come this day and I say, "Doctor, can you remind [Ric] *0:42:33.6," his name was Ric and I said, "Can you remind Ric about the cigarette?" And the doctor said to him, "If you want to die, because you are sick enough, if you want to die, but why? You know, you don't have to, I told you don't smoke, but you did it in front of me, you did it front of anyone, you smoke, do you want to die?" And he just stay quiet and he say to me, "You with your big mouth," because I'm thinking <inaudible> *0:43:05.9 you know, because that's hurt me, because doctor told him, "Don't smoke" but he had a smoke. But, that's not good for you to have any medication is not good, you know what I mean, but you have to listen to the doctor, like I said, you have to listen to him too. We can't just say, "Listen to us," because if you want to smoke we smoke, if we want to drink we drink, you know what I mean? But, we have to listen to the doctor to get advice. But, that's look like me, I don't smoke, I don't drink, but my diabetes it's very long time I get diabetes, but I would like to know, if that will worsen and for my diabetes and kidney problem. I get it for a long time, I don't know, but I know new machine or thing just control for the blood test and urine test.

**Q: Let's move onto our experiences with living with diabetes and chronic kidney disease. In your opinion, what do you need to look after your diabetes and chronic kidney disease well?**

John: Can I start that?

**Q: John, yes of course.**

John: When I first came out of hospital, I see the need. I knew nothing about diabetes when I come out, so I went to Altona. I live in Altona, but in Altona Meadows there's a group there called ISIS and I booked myself in there to learn about the foods and the medication and how long it'd last and would I be on tablets for you know and after a while you will be injections and all that sort of stuff. So, we started up a support group called the Westgate Diabetes Support Group and you'd be amazed at the meetings we have there. We have it at the Altona Bowling Club. We have it there the first Tuesday of every month and you'd be amazed the people that come in there that have been diabetics for years and really know nothing about the subject. And we have nurses and also educators from a lot of the people that make the testing equipment and all that sort of stuff and we have like a theme every month of what goes on for our own benefit. And we have a few doctors and a few others and a couple of the chemists that surround the place to let people know what's available in the cause of diabetes. And the last two or three meetingsI haven't been, but before that I used to be on the committee; there was three of us on the committee. We had like a bank account and we had cheque book and we have an end of the year Christmas party and all that stuff; it was good. But, what I noticed was that during the three years that we've been doing it or four years now, not many men come. Not many men seem to want to go to those meetings. The women will come and a lot of the women come for their husbands, it's amazing and so we started up a little flyer for people to take home.

**Q: So, what do you find particularly valuable from that? Is it the peer support or is it more information and knowledge?**

John: Yeah support and knowledge of it, yeah.

**Q: So, is it more the knowledge or just the sharing of your experiences of diabetes?**

John: Well it's, people don't really-- we've all got different experiences while we've got it, but down our way, there's a lot of Italians. And the Italians, they approach it entirely different. The older Italians, they know why they've got diabetes, because they're not eating enough pasta or… These are the things that come out and I think my God, you're not eating pasta, it goes through the roof. And breads and what should eat, like I was always told that breads with a lot of seed is good for you, but I've noticed that if I eat it, it sky rockets.

Rob: Try focaccia.

John: So, I stay away from it, but unless you understand and read some of the books and you start to learn about why your blood sugars are high and it's because of the carbohydrate intake you have and it's your body trying to convert that sugars, that your own insulin is not doing it. So, when you take these other tablets and insulin to coincide with that, it's stopping your own system from going sky high and getting out of control altogether. If you don't control it, in the future your body system's just going to stop, it won't be able to do it anymore. So, it's very important that you read some of the books and you get a bit of an understanding of it, which I never had. Look, I run *0:48:48.6, I'm in the *, I've never done anything like this before, but after my accident and I had it because of my kidney problems and that, I had to learn about it, but you'd be amazed how many people don't. And it's a wonder that a lot of other people from hospitals like this are not taught about their local groups and you know you don't have to come to hospital for every little thing, you've got to go teach yourself and that's what I've been doing. And that's my two bob's worth anyway.

**Q: So, really what you're saying is having knowledge, perhaps. I mean it's stuff that you want to know as well. I don't know whether everyone wants to know and then just that peer support there.**

John: Yeah.

**Q: Any other comments about what do people need in place to look after diabetes and chronic kidney disease well?**

Frank: I think a lot of people are not aware of the consequences. Look myself; I'm the kettle calling the pot black, you know, because I tried…

**Q: Say that again.**

Frank: I'm guilty. I know that it's not a good thing, but I tried to control myself to a degree. For example, when I go into hospital for whatever situation, I always lose weight, because the portion controls in hospitals are better regulated than when you're at home and I think that's a big thing that a lot of people are not aware of and the types of foods. But, the consequences of having the illness, I think a lot of people don't want to accept or they're in denial, I don't know. I have a friend who was 55 and he was a couple of years younger than me and he used to talk about symptoms and I'd say, "Joe you've got this and you've got that" and I'd say, "When you go to your doctors mate, ask him about this." Anyway to cut a long story short, he has a heart attack and he's in the hospital and doctor's telling him, "Well you know about smoking." And he's turning around and saying, "Well all your nurses go outside and smoke." And I said, "Joe, he's telling you about your problem; don't worry about them." Anyway, he just dropped dead one day and I'm convinced that he killed himself from overeating, because he used to indulge in this and indulge in that. Look I do that myself and sometimes I think about it and when I came, his partner rang me, I went to see him at home there and when I came home, my son who was about 14 at the time, he said, "Dad is this a wakeup call for you?" And because he's always concerned about my health and I said, "Yes, it is a wakeup call."

John: Sorry, I've got to go. Thank you very much; I'm sorry I have to go.

**Q: I understand. No thank you John.**

Frank: I'm from Altona as well. I've seen you around and I can't…

John: On the bike, probably.

Frank: I don't know, but I've seen you anyway. I'll try and pop in one of those meetings at the bowling club.

John: Bowling club yeah.

Frank: First Tuesday.

John: This time next Tuesday.

Frank: Okay, I'll probably see you there.

John: Thank you very much for your time.

**Q: Anytime, thanks again.**

Rose May: What's the best for you?

**Q: Okay, so we'll let Frank back in.**

Rob: Well I don't think you should.

**Q: Okay, so I think basically you were talking about knowledge, about the consequences, perhaps being able to accept the fact that you've got…**

Frank: Yeah you've got a problem.

**Q: …the condition as well.**

Frank: And I think it's good if your family members know about it and my wife hounds me all the time and I drink a lot of water, but I've tried to reduce that, because apparently it's not too good, but anyway, occasionally though you want some different…

Rob: Flush your kidneys.

Frank: And I say to my wife when she does, "Can you buy some soft drink?" "You don't need it." But, occasionally we do it. We don't keep any at home, so it's not consumed, because once it's at home it becomes consumed, so things like this.

**Q: So, family support really?**

Frank: So, family support is terribly important and I have other people say to me sometimes, because I've got a sweet tooth and they say to me, "Oh chew it and then spit it out." So, you're getting the taste, but…

Merle: I don't inhale.

Frank: But look, like I say, every time I've been in the hospital I always lose weight and then for a period of time I come out with that mindset, I control my portions and my food and I continue. But then, because you've got access to more food and as John said, I'm Italian, come from Italian background and the thing is there's always too much food prepared and you have a decent dish and then when you're finished, your mother will say, "There's more there if you want some." And you say, "No, I'm fine." And what she's doing as you're saying, "No, I'm fine, that's enough thank you," she's topping up your plate and it's something that you like and you say, "Oh alright." So, it goes out of hand see, but you've got also look at the fact where they come from, with all that in mind, my father's 90. And he was a heavy smoker, but he led a simple diet and my brother and I sometimes look and talk about how he's just developed diabetes. A few years ago…

**Q: He has or just has?**

Frank: No just has, a couple of years ago. He's had medical problems developed in the last two to three years with it. He wasn't going to complain. Anyway one day he said, "Oh" because he works with asbestos, he said, "I think this asbestos has become active," because anyway he had developed shingles, but in the process, they checked his cardiac and respiratory system. His left lung is basically non-existent, but if you walked with him, you'd have difficulty keeping up. And the cardiologist said to him, "Are you an organ donor?" And he said, "No" and he said, "Well you should consider it, because your cholesterol's perfect, everything's perfect and this is 87." You understand me?

**Q: Yeah.**

Frank: So, he's not a person that looked after himself, but what I noticed was he would have a simple dish and that was it. He never got into the sweets, he never got into this and never got into that. And I think a lot of these problems that we're having, as I said earlier, are because government regulators and people are just trying to make that extra dollar at everybody else's expense, so.

**Q: What do you mean by that?**

Frank: Well, for example, I live right opposite a McDonald's store and now they've just planted a Hungry Jacks on the other corner and they have those cold drinks for a dollar and I said to my son, "Well when Hungry Jacks comes on it, that'd be great." So, McDonald's has gone to a dollar as well in competition. But then, just recently on TV, we noticed that it's got 16 spoons of sugar in it. I just don't understand that, because it doesn't taste that sweet. So, we've stopped buying it. We used to do it pretty regularly, but we've stopped buying it, because of that, so. But, Hungry Jacks and McDonald's is making a fortune out of it. I mean, everybody who's owned a McDonald's or a Hungry Jacks franchise has become a multimillionaire, but then we've got the government saying, "Oh the health system, this is this, it's deteriorating because we've got an obesity problem." There's too much food to start with, but then the quality of foods. See, being of Italian background, my mum used to preserve and make things, for example smallgoods. Now, I was waiting one day at a deli, buying some stuff and I picked up a piece of pork belly, which we would only cure with salt, but I picked it up, because now I'm conscious and I always read the packets when I go showing, about what's on them. But, unfortunately I don't know, I can't even read some of the words that are on there. Now, this piece of pork belly had sodium in it, but it had half a dozen other items in it. Now, the one my dad produces at home is tastier and it doesn't have all these other additives. On Sunday for example, I made my own sauce for the pastas and so forth. People say, "Why do you do that?" Because the one you buy has got ingredients in it which it shouldn't have; it doesn't need to have, but somebody's making money out of that and I feel the government, in one minute they're talking we've got an obesity problem, but then in another point, they're not trying to regulate certain people.

**Q: So, what you're saying is the government should put in some…**

Frank: Controls.

**Q: …controls for public health to help prevent some of the obesity <inaudible> *0:58:36.0?**

Frank: Exactly, for example an item that's produced today, I don't understand why it's got to last for ten years. You see it's good until 2020. Why does it have to last so long? Then we're talking about unemployment, well if you produce it every day, there's got to be people kept in employment to supply the market and it's a better quality. But, the employer, he's looking to cut corners so he's making a dollar profit, but that's not enough, it's got to be two. And then in the process, they're doing all these things. It's the same as the soft serves you get at McDonald's and so forth, they're absolute fat apparently.

Rob: But nobody forces you to buy them.

Frank: Exactly, but the thing is people do, because they then have their advertising campaigns, which they spend millions and the thing then people become addicted. Fortunately I've managed to cut. Look I used to have relatives come to visit me and they used to say, "Oh this is great, you live right opposite McDonald's." "No," I said, "it's not great, because when my kids become a bit older." They used to there, buy their stuff and come to my place and I said, "Look, I'll welcome your visit, but if you want to get McDonalds, you either eat it there, don't bring it to my place." Because I said, "I live right opposite and I've got to try and control my children from not becoming addicted from it" and I managed to do that. But the thing is I'm the only one, I've never really had a McDonald's meal. Every time I've been in hospital and the dietician comes to talk to me, they say to me, "You don't actually have a bad diet. What you have is bad eating habits." But, I'm always conscious of what I eat and we go out to the occasional restaurant and my children say to me, "Dad, you're always complaining. Why don't you set up your own business and cook and this and that?" Because I say, "This is shocking," pizzas for example.

**Q: So, what you're saying Frank is so you think the government should become..?**

Frank: Oh yes.

Rob: No.

Frank: Even with the schools, because see my wife's a teacher and she said, "Some of the junk that the children are given for their lunch is pathetic." Now, they tried a program there where they were getting fruit every week, once a week.

**Q: So, Frank you're talking about public health?**

Frank: Yeah.

**Q: So, can I just wind you right back? Apologies.**

Frank: Yeah.

**Q: So, just wind it back to I think, what does everyone else think, especially about things..? So, what you're saying is you think it'll be helpful, if you have had the government controlling food?**

Frank: Yes, the manufacture of food.

**Q: What do other people think in terms of factors, the things that they need in place to help them look after their diabetes and chronic kidney disease well?**

Rob: I would like to see more things made with artificial sweeteners and less sugar. You try finding on average four drinks that do not have sugar in them in the supermarket. But, a lot of people who are diabetic don't want to know. If you say to them, "Yes alright, bread converts to sugar" and they go, "Really?" And you say, "Oil converts to sugar, so does fat." "Oh really?" They might think about whether they should have that second piece of cake, because it's so sweet, but by the same token, the people who made those cakes don't ever consider making them with artificial sweeteners. No, they use sugar and why? Because sugar is part of what keeps industry going. Now, the government is not interested in trying to force people to eat healthy, which is basically what Frank's suggesting. People have the right to decide what they shove under their own noses and if they're not prepared to take the time and make the effort to look after their own health, then why should somebody else or the government do it for them?

Frank: What you fail to understand is that sometimes people, it's like children, if you don't guide them they're not going to get anywhere. And unfortunately people who are 70 at times need guidance as well, because see the government and the nation in general fails this, because then the government can't cope with the health system to keep all these people looked after medically.

Rob: Then why are they allowing smoking?

Frank: And then we suffer why, as you said about the time factors and all that. If people were healthy, they don't go to the doctors. A lot of people go to the doctors when it's too late. It's a place that people don't want to go.

**Q: So, we've got the regulated food industry, then I guess John was talking about being aware, education and then your main point was people need to motivated as well, I guess is what you're saying.**

Rob: People need to be prepared to accept their medical condition and most of them don't want to have to do the effort themselves. They want to go to their doctor and the doctor's got to give them a magic pill which means that they the patient don't have to do anything. It' doesn't work that way.

Merle: It would be nice.

**Q: Merle have you got anything you would like to add or the things that you think need in place? So, I think education, support, awareness; anything like…**

Merle: I think education is important. When I was first diagnosed, I think that I was in a state of denial and I did go to a couple of sessions at the Diabetic Association, whatever it was called then, but I didn't really take it in. But, now is the time when I'm looking for something. I'm looking for not only the correct food to eat, but probably the quantities and the food that is going to help me to lose weight. And so, I'm really searching at the moment for that right thing. I mean I'm very aware of what I eat. I lash out every now and again, but I'm very aware about sugars and fats and things like that. But, I see that they're advertising a new book coming out this week and I'm just wondering if that's a good one for diabetics. I sort of wish that there was a register or something where you could get up to date information on food and what's in it and what's good to eat and what's not, yes because I want to lose weight.

**Q: Look due to interests of time we're going to have to move on, we've got two more questions to cover. The next question is opposite to the first, so what things make it hard for you to look after your diabetes and chronic kidney disease well?**

Merle: Merle, my sweet tooth.

Frank: Just a routine, getting into a basic routine and trying to maintain that, because I know in my household, my wife tries very hard, but it gets let down from other things, but I think getting into a routine. If you could get into a system or there'd be some places where-- I suppose there's that light and easy for example, I know people that've gone on that and they say, "Well you just eat that." Because, if you look at the weight loss for diets in general, the common denominator is portion control and exercise and you don't need to cut anything out. If you've got a sweet tooth, instead of eating two cream cakes, you eat one and that's all you do, is that portion control. Portion control is the problem with weight, even if you don't do exercise and I'll give you the classic, my father brings this to me on many occasions. He said, "You look at the prisoners of war, they had no food. Were they fat? No." And he said, "In my day we were limited with quantities of food" and when I look at their photos, he's right. He said, "If anybody had a belly on them, it's because they were bloated for some illness, not because they were overeating." And everybody's nice and slim, because you see you only had a certain amount of food every day. You didn't have the fridge and so forth.

Rob: And a lot of exercise.

Frank: Sorry.

Rob: And a lot of exercise.

Frank: Yes.

Rose May: Yes, I will tell you what you have to do. This is a doctor from Prahran, I live in Prahran before. Unfortunately the doctor died now and when I get diabetes, I didn't know I get diabetes, so I say, I get like a system, each whatever. And my friend had diabetes and also my mother's friend and I say, "Oh it's why?" He said, "Oh, it could be you get diabetes. I used to have 15 cup of tea, three sugar in each cup; 45 spoons and after I was 160 pounds, very fat. And now I went to the doctor, he send me to yeah the hospital here, Hospital 1 and then they take my blood, take my urine and they say to me, you've got to go home, have something to eat before you get digestion you come here back. I went to my sister-in-law, I had a big lunch and something I didn't have to eat and she said, "Eat, you are going to the doctor; eat." So, I eat and went back. He said, "You [need] *1:09:30.6 that my diabetes is." What you are to do now doctor, I go back to my doctor, he says, "You have one poached egg, one slice of bread toasted. One poached egg, one toast, one strip bacon, people who have bacon, one."

Rob: Quantity.

Rose May: Yes and then, that's all. You drink milk and no sugar, you stop your sugar, stop your cake, everything. I try, because that's my sugar and I try no sugar, no cake. So, I listened to him, water, dash of milk, no a cup of tea or a coffee without milk, without sugar. Lunch time, at lunch time it is soup, it's clear soup, vegetables, clear soup and one grilled meat or fish.

**Q: So, it sounds like everyone's really worrying about their diet in terms of control?**

Rose May: Yes, yeah one, I want to tell you.

**Q: Quick question, what time did we come into..?**

Rose May: I lost weight, I lost weight. One-sixty, my dress become nine-and-a-half; I was 18, I was 18 dress.

**Q: So, it sounds like the food and diet's really important?**

Rose May: Yeah, diabetics, yes.

**Q: So, what time does everyone need to leave? Does everyone need to leave at 2:00 sharp or..?**

Rob: Time is not a problem.

Frank: No, I thought it was 3:00 O'clock.

Rob: Sticking to the program is the problem.

Frank: What time is it?

**Q: It's 1:30.**

Frank: Okay.

Merle: No it's 2:30, it's later than that.

**Q: No 2:30, so people need to leave at 3:00 do they?**

Rob: Yeah.

Frank: No, I'm happy to stay as long as you want.

Rob: I'm fine.

Merle: Yeah.

**Q: So, what things make it hard to look after diabetes and chronic kidney disease? So, it sounds like sticking to the diet is hard.**

Frank: Yeah.

**Q: Anything else which makes it hard? So, other people mentioned things like other illnesses, low mood, lack of support from doctors or family or conflicting information being given by doctors, anything like that. So, any..?**

Frank: Lifestyle in general is a major issue. Everybody's stressed these days, nobody wants to do any physical work at work and then they go and spend two hours at the gym. I knew guys and I know guys in the building industry, concreters for example. Well, they don't go to gym, but if you look at their physique, every muscle is solid as a rock, because they're fit. Carpenters, same thing, you know. I ran a mechanical workshop and I used to buy oil in and when I first started my workshop, the delivery guy would deliver 205 litre drums off a back of a truck and yet he would unload himself. He'd lift it up and roll it into my workshop.

Rob: A 44 gallon drum.

Frank: Yes, anyway…

**Q: So, are you saying you think that our lifestyle's too sedentary at the moment?**

Frank: Yes, yes.

**Q: And that makes it hard too, is that what you're saying?**

Frank: Yes, because people aren't exercising enough. We got these…

Rob: OHS.

Frank: …multiple dwellings. They've ripped down a home and they've put three dwellings on there, there's no backyard. You know, Australia was known for the great backyard; well the backyard doesn't exist anymore. I heard it this morning on the news there, on Channel 9 there, anyway, "We haven't got a yard and the kids are always on those gadgets."

Rob: <Inaudible> *1:12:59.4.

Frank: So, society in general is really killing itself, because they're going away from the basics. Rob was saying here about using artificial sweeteners. I don't agree with that, I think that's a lot of nonsense, but that's my own personal opinion right, but the thing is everything that's modified, somewhere along the line, it compromises something else, okay. So, the whole thing is why I say the government, because they are leaders in various fields and they are supplying the medical assistance to the society, well they should be the leaders. See now, they stopped apprenticeships a few years ago, they got rid of all the tech schools, now we haven't got enough tradespeople and they want the older people to work on longer. That's a lot of nonsense.

Rob: Yes it is.

**Q: But what do people think, like for yourselves, what makes it hard for yourself? I know you're talking about all this environmental stuff, but what makes it hard for yourself to look after your diabetes and chronic kidney disease well?**

Merle: I find that some foods which I think you have to work out what suits you and what changes your glycaemic readings against other people, because what affects other people sometimes doesn't affect you and the opposite way around. Like, people will say, "Bananas are good for you." Well, if I have a banana, I just go sky high, but other people have a banana and they're level, so you need to work out your own level in what you do and I think well for me, I just have to admit that I've probably done the wrong thing for a long time and I've probably left it too late to change too much, but I just have to be aware of what I'm eating and not be tempted by the things that I really love and just leave them be. It's a matter of self-discipline and that's not easy for most people and probably getting a bit easier for me and I've certainly reduced my meal sizes and things like that and I try not to have anything with sugar and stuff in it.

**Q: Now, there's been a lot of focus on food and exercise and lifestyle. Any other things which may act as barriers in terms of you guys having well controlled diabetes and chronic kidney disease?**

Rose May: I collapse about five times. Is it the diabetes make me collapse or the kidney problem? I collapse, like my blood pressure just gone, dropped for ten-point-two the other day and I collapsed, but that I come to hospital.

**Q: Let's talk about that later. I'm not sure that's due to either, but we can talk about that later.**

Rose May: Yeah.

**Q: Any other factors that act as barriers?**

Rob: I think a lot of the times too, you were saying earlier about mood swings, but just sheer depression for some people. They get depressed, they don't eat properly. They just, "Oh I'm hungry; I've got to eat something." They don't care what they shove under their noses; they just shove it in and if you look at some of the calorie content of what people put in. I mean even cornflakes, just bare cornflakes are what 1,600 kilojoules per 100 grams or something like that and then if you add the milk in, wow it goes right through the roof. So many people just don't think of milk as a contributing factor to glycaemic index, but it's amazing how much difference it makes, how much energy it carries, we don't get told about it. They say, "Oh I drink skinny milk." But, even skinny milk or skimmed milk is still very high in kilojoules.

Frank: On that romp, this is what I've been arguing all along is the fact, why does the manufacturer Kellogg's have to add sugar to it? Why not just grind the corn and just make flakes from it?

Rob: That is the bare content from the starch in the corn. It's got nothing to do with any added calories.

Frank: That's what they tell you.

Rob: Just the natural.

Frank: They are allowed to put additives in certain things. There's a certain amount of quantity of additives that they put in product that they don't have to tell anybody about. It's the same as, look on Sunday I made my sauce for the next 12 months. Now people say, "Why don't you buy it? What's that bottle cost you?" And I say, "I don't know, because I don't bother working it out." "Oh you can buy them for a dollar each" and this and that. But tomatoes, depending where they're grown, depending how they're grown have a different outcome, but when you buy the bottle, the contents and the texture is always the same. It's the same as making wines. All the books that I know that make wine, all they do is crush their grapes, press it and put it in a barrel. One year it becomes stronger than the other, because they don't regulate the <inaudible> *1:19:06.6 right…

Rob: Because of the sugar content of the grapes.

Frank: Exactly, so but Brown Brothers make a particular wine called Muscatel, every bottle you buy is the same. Why is it the same? Because it's got to be modified, because one year we had too much water and the grapes don't have the good sugar; next year we have great sun and they dry up nice, so they've got great sugar. So, they've got to regulate that and they modify it by adding and subtracting and all the rest of it. And this is what people don't understand about the problems. It's the same as plastic bottles, once you feed the children with the glass bottle, then they introduced the plastic bottle so they don't break. Well then we, "Oh if you leave it in the sun it expels chemicals." Well, if society knows that, why can't we go back to glass? No, because it's just too costly. Once upon a time we used to have a refund on the bottles. When I was a kid, I used to have a billy cart and I used to collect bottles all along to refund them back. No, it's easier to do it this way, because there's more profit in it. It all comes back to the dollar factor. This is what people don't want to understand, like I say, people are not prepared to earn one dollar; it's got to be two.

**Q: Thanks for that Frank.**

Rob: Not relevant to this place.

**Q: Let's move on. So, let's go back to think about the health services again, so health services for diabetes and chronic kidney disease. Now this question is the last one and it should be good fun as well and hopefully it'll stimulate a bit of discussion. So, everyone's made comments about strengths and weaknesses and likes and dislikes about the diabetes and kidney disease health services. Imagine if you became director of Hospital 1 and you had power to change things about the diabetes and kidney disease services. If we were going to design a perfect health service in inverted commas, which will meet all your needs, what would that include?**

Rob: A conveyer belt and a whip.

**Q: And then we also have with realistic funding as well, if we were going to design a better health service, what would that include? Let's open up the floor. Who wants to go first?**

Rose May: What you can see when the time's coming.

**Q: Rose May yes.**

Rose May: When the time is coming, they are going to decide what to do with us. We come here, they are looking after us. We are to be grateful, because the time is running out for when we get old the time is running out, you know what I mean?

**Q: Yes.**

Rose May: But, what we can do, we have some place they are going to take care of us, we are to be grateful.

**Q: So, what I'm getting at here is, what improvements can we make? So, if we've got…**

Frank: Trying to educate people about the consequences of doing the incorrect thing and to some degree it's the same as a person who's a criminal, to some degree you're being a criminal to yourself by not looking after yourself properly.

**Q: So, you're talking about the general population and you're saying..?**

Frank: Yes the general population.

**Q: So, you're talking about the general population, public health?**

Frank: Yeah.

**Q: Anything else you would suggest if we're going to improve health services here for people with diabetes and chronic kidney disease?**

Rob: I find that most people refuse to accept responsibility for their own health and doctors sometimes go along with that and are only too happy to have XYZ has patient and treat the symptoms without education the patient so strongly that the patient gets the message and actually wants to do something for themselves. There are so many people that come in here and they're sitting in the waiting room for the diabetes clinic and they've been downstairs, they had breakfast at about 8:00 O'clock, here it is about 10:30 and they've been to the cafeteria and they're stuffing their faces with something with high glycaemic content and a sweet drink, coffee or tea or whatever; chocolate. And if you say to them, "Hang on you're in the diabetes clinic, do you really think you should be having that?" And things like those lovely sausages outside Bunnings and places like that, they say, "Ah, but it smells so lovely, I just couldn't resist and you say to them, "Okay, at breakfast time at 8:00 O'clock, which gave you slightly more than you needed in terms of energy to keep you going till noon or later. Now, you've put in twice as much energy, which you don't need and you're still going to have your normal lunch. Now did you think about that?" "Oh yeah, but it's only once and again." And they just refuse to accept that it is there personal responsibility to look after their own illness. It's not up to the doctor to treat them all the time, they should be preventing. Now, like people who smoke. In Europe there are places where they go to a hospital and if they're still smoking, the doctors flatly refuse to treat them, because they're saying, "Why are we wasting our time?" And I'm beginning to think that in terms of the diabetes clinic for instance, doctors should start taking the same attitude, "It's your illness, you're responsible for what you put into it, now start taking care of it yourself."

**Q: Thanks for that Rob. Any other comments in terms of how things could be improved, if we're going to design a better health service?**

Frank: I keep harping about the education. When I was young, my father used to push me to go to school, because you need an education.

**Q: But do you think the education provided is adequate now?**

Frank: No, not at all, because not an adequate amount is provided at the right time. See they've tried to start at schools where they have their own gardens and they cook the stuff.

**Q: You're talking about public health though aren't you? You're talking about in terms of preventing, aren't you?**

Frank: But the thing is see if you go to Italy, everybody there's on portion control. The people that reside in Italy, that live in Italy are all slim. Now, my mother had some relatives come over and she cooked for them, invited them over and when they saw the table spread, one of the guys said, "Mate, I'm having a heart attack before I even start because," he said, "Who do you prepare all this food for?" Because, the mentality there is that you have something in the morning, then you might have something in the lunch and then very little in the evening. They've got the right pattern, because they've been educated that way to do that and everybody that I've seen or relatives, they're all slim. They see us when we send them photos and they say, "Gee you guys are big." Believe me, it's cheap in Australia, because meat's expensive over there so they don't have the barbecues like we have here. I mean you're supposed to eat so much meat, I mean I'll eat a dozen of those at times.

**Q: So, it's public health and education on portion control right?**

Frank: It is, but it's got to start from when, as if you're training a dog or a person, you've got to get people into a mode of things, you know.

**Q: So, what you're saying is public health even when people are young?**

Frank: Let me say this, if you have no regulations whatsoever and you have a buffet every day and you say to people, "Help yourself," what do people do? And if you go to a buffet it's, "Oh I'm going to go, because I've just paid, you eat as much as you like" and they overeat. Not that they really need to overeat, but they do it so they can get value for their money.

**Q: Okay, so public health again. Anything else? What about actually improving the health service here at Hospital 1?**

Frank: Well the thing is I don't know, because I haven't been here, sorry.

Merle: Merle speaking. Probably the thing that I would come back to would be perhaps the waiting time in some of the clinics. Some of the clinics are not so bad, but some of them are and there's probably lots of reasons for that, that people waiting will never understand, but that is a big thing. Probably some good reading matter.

Rob: Bring your own.

Merle: Ah well see, well not everybody thinks of that and it's nice perhaps to pick up something that you haven't had at home and not everybody can afford to buy magazines.

**Q: When you say good reading matter, what do you mean? Do you just mean magazines?**

Merle: Yes.

**Q: From other groups, some people have actually mentioned maybe them providing educational material while waiting.**

Merle: Yeah I was just going to say about educational stuff, yeah something like that, because in some of the clinics when you go there, there is virtually no reading matter at all and it gets very boring just sitting there for hours.

**Q: Any other suggestions or comments? I think both yourself and John, Rob were mentioning about, I think there was problems with information transfer.**

Rob: Yes.

**Q: And would you guys want to improve, if we were designing a perfect health system or a better health system, would you want to address that?**

Rob: I would say that there should be more integration between pathology services, because as I said earlier, they all hoard their own information. I suspect that there's a kickback system for doctors in terms of who they ascribe their patients to for pathology and that pathology labs do provide various forms of kickback in one way or another. But, none of it is in the interest of the patient. It is all interest of the health professional and we've got to keep it mysterious, we can't let the customer or the patient know everything about their condition that we know, because then we can't have our little mysteries. Now, in here…

**Q: Sorry, can I ask what do you mean by that?**

Rob: I mean that quite often, doctors will say, "Oh yes and so and so, such and such, but don't worry about that now," rather than them explain how this interacts with that.

**Q: So, you're talking about medical conditions or what are you talking about?**

Rob: I'm talking about medical conditions and the fact that doctors once again, try to hoard that little bit of information, rather than pass it on to the patient or, as I said earlier, my GP doesn't get my blood results from any tests done here.

**Q: So, you do think doctors don't explain themselves enough? Is that what you're getting at?**

Rob: They don't and I think in a lot of cases it's deliberate, because they like to keep themselves just that little bit above their patient.

**Q: Does everyone else relate to what Rob's saying about doctors not giving them enough information?**

Frank: Personally, my experience has been that whenever I've asked questions, they've been answered. I try and write questions as I think about them, so when I go in… I tend to understand where Rob's coming with that, because it comes down sometimes to the personality of the person, especially if he's busy and he's going to mention all these words that you don't know what they mean, they cut it short so firstly the don't confuse the patient, so they don't get paranoid about their situation. So, I think it's a bit of a balancing act and some have got bad bedside manner. I think doctors, in their field should be taught good beside manners. I just quickly went for carpal tunnel once and my GP said, "I'm going to send you for a second opinion." Well, when I went there, the guy doesn't introduce himself, he sat down, gets his diary out and he says, "Oh is the 18^th^ alright?" I was totally lost, but he's regarded as a real expert surgeon this guy. But, I had to go and get something else done and my doctor said, "Oh you want to…" I said, "No I don't want to see him." He said, "Why not?" "Because" I said, "he might be good, but he doesn't make me feel good."

Rob: Not sympathetico.

Frank: So, it's not a question of being sympathetico, it's just a question of mannerism. The surgeon I'm seeing now for my foot, I went to see him yesterday. Every time I see him, "How are you Frank?" And he shakes my hand. "Call me Nigel, don't call me Mr Mann." So, he comes down to your level with talk, "How's it going?" This and that, did I have any questions? Look that's what I find, that my experience is I try and think about questions and then when I go I ask him. But, you get shonky operators in all professions, there are a lot of doctors who are not doing. My brother developed bowel cancer six years ago and he was in a critical situation. They told him to go home and get his affairs in order and the following morning we're at an MRI; that afternoon we're seeing a surgeon and my brother says, "Oh look, you know" and he said to my brother, he said, "Mike, I don't want to be talking to you next week and you haven't decided what you're going to do." He said, "You haven't got that time." So, I have a cousin who her daughter has become a doctor and she's an oncologist. So, she said, "Oh I know so and so," ring him up, "Oh he's away." They text him, "Yes, I'll see this person; I'll be back in T City in two days." They formulated an appointment for him. We go there and soon as we enter his rooms-- he only works at the Mercy, nowhere else. Again my brother had full high cover, but the receptionist gives him a form and says, "Doesn't matter what cover you have, you're out of pocket by $3,000." So, we go in and he's harping at this and he's harping at that and he leaves the room and I said to my brother, "Mike, just book with this bloke, he's got everything covered."

**Q: So, you're saying bedside manners now or..?**

Frank: Well, no this is professionalism. He's a salesman, this guy. All he was interested in was making money, because the original surgeon he spoke to did this test, that test. So, when we go to see this guy, he wants to redo all these tests.

**Q: So, can we just leave that thought there for a moment Frank, because I just want to address what Rob was saying before. Rob was saying before about, that's right, doctors aren't giving you adequate information. Do you identify with that Merle?**

Merle: No, that hasn't been a problem for me. If I ask I usually get the answers. I've got an appointment with my renal doctor in next couple of weeks and I've got a couple of questions in my mind that I want to ask.

**Q: So, they'll always answer everything? They don't withhold information?**

Merle: Yeah, sometimes I don't want to know anything, but I'm getting to the stage where I want to know what this means and what that means.

**Q: And Rose, does anyone withhold information from you or when you ask do they tell you everything you need to know?**

Rose May: Yes, when I go to see my GP, my family doctor, if some problem, they send me to your hospital, Hospital 4. I am very grateful too; they look after me very well. But, because I was in Carnegie, that's my husband who died in the hospital.

**Q: What about the doctors here at Hospital 1, when you ask them, do they withhold information from you?**

Rose May: No, they're alright. I am appreciative. I am sick, they are looking after me, but what they say, I have to listen, you know what I mean?

**Q: So, any other ways we can improve the health services here at Hospital 1?**

Rob: More doctors possibly, so you don't wait as long. Now I know no matter how many doctors you get, there are always going to be more patients than doctors and there are going to be times when their load is just so high that they can't possibly see everybody in the time given. But, there's got to be a better way to manage their time and a lot of that is because of the admin they have to do. For instance, if I make an appointment, come in here; see the doctor, quite often the doctor then goes onto the computer system to make my next appointment. Now that's not a doctor's job. He should just give us a slip like some doctors do, they give you a slip and they say, "Give it to the receptionist." And the receptionist looks at it and say, "Oh yes, three weeks, three months," whatever and does the appointment, but quite often I've found the doctors will want to go in to make the appointment and they can't because the system is locked, because this person is using it over here, so that person can't use it there. So, to a certain extent, the computer system that is available to doctors ties them down. It needs to be a lot faster and a lot more integrated.

**Q: So, that's mainly the bookings system or are you talking about also the health records as well?**

Rob: The records as well, yes.

**Q: So, Merle you want to add on?**

Merle: It seems to me that all the appointments are made for the same time. I can't see why they can't be graduated a bit so that if…

Frank: Quarter of an hour apart.

Merle: Yes, so that if I get an appointment for 2:00 O'clock, so 100 other people. Well the doctors are not going to see 200 people all at 2:00 O'clock and I understand that, but then it's still a long time to wait. Where if I had an appointment for 2:30 I wouldn't come in till 2:30, so therefore I'm not waiting as long or it might be as long, but it doesn't seem as long, because I haven't been here since 2:00 O'clock. So, I just wonder why they make so many appointments all for the same time, because you get a great line up at the reception desk, so you know that everybody's got the same time appointment. It's a matter of who they've got in first and I understand that.

Rose May: Sometimes they ask me, what do you prefer; to come in the morning or you come afternoon for my appointment. So, because I have to come by public transport I say afternoon. Sometimes when I can come in the morning, I come in the morning, because before I come here, I have to come from Clayton, I stop Hospital 1a. Hospital 1a I change and after I come for a-- what are they called? A *1:40:17.3 and then I am coming here, that's the turn, but that's like I say, sometimes they ask me if you want to come early or you come late and I choose my time. But, like I say, if when I come here, something worse when I see my GP, something worse, I come to the hospital in Hospital 4. But, I come here all the time, because I am here when I was young. Thank you.

**Q: Any other suggestions in terms of improvements?**

Merle: No, just keep improving, because I'm sure you're looking at it all the time.

**Q: You mentioned before that you wished that you could see the diabetes and chronic kidney disease doctor on the same day. Is that something perhaps that could be addressed in improvements?**

Rob: I don't think so, because you find they use the same rooms to do renal one day, diabetes on the next, something else on another day, so you can't double book the rooms.

Merle: No, it's probably not practical.

Rob: That's where the practicality comes adrift.

**Q: Well what happened if, some people thought about combining and having a diabetes and chronic kidney disease service together; a clinic combining both. What do people think about that?**

Rob: Different disciplines, different doctors. You're going to have doctors standing by from two different sections to see one patient. Their questions quite often will conflict. Not necessarily conflict, but not work together and the patient is left doing the tennis match thing from one doctor to another. So, I don't think that would work.

Merle: I suppose you are talking about two different doctors, because not the same doctor wouldn't be doing renal as well as diabetics would they?

**Q: Well it depends. They're different variations that people have suggested. Some people suggested, but some people suggested a combined diabetes renal disease clinic.**

Merle: But are we looking then at two different doctors?

**Q: Where a patient might see the diabetes doctor and the kidney disease doctor all at once or some people have suggested even somebody who's trained in both.**

Merle: I think if they were trained in both, that would be good, that would be good.

Frank: I think it could work, if you have a particular place and this person's coming in today and the two specialists are available, that they would work a schedule between him, so okay I see him in the morning or which diseases takes priority over the other or which one needs to be seen first and so forth. I don't see why that couldn't work. It could work, I feel.

Merle: I think you need to have another building to set up that thing.

Frank: Yes, exactly it'd have to be a combined diabetics and kidney unit, whatever it's called.

Merle: Yes.

Rose May: Yes, see the same time, together.

Frank: Yeah.

Rob: The trouble there is there is so much to know in any one of these disciplines that it would be almost impossible for a doctor to keep up to date technically with both disciplines. They would be having so much time off to go to seminars and lectures and additional study that basically they'd have little time to see patients because they're too busy. As soon as you learn something on one discipline, then something else comes up on another one. So, the amount of information that's going into their heads is so much, it's almost impossible for them to keep up. And I respect doctors, they know a hell of a lot, most of them, but how much can you keep putting in and at the same time expect them to apply it to their patients.

Merle: I suppose I'm thinking that not all renal and diabetic patients would be seen in that clinic together, but people with particular needs in one area or something would probably still need to be seen by someone specialising in that area. But, for someone who's fairly on the level, like I spend, as I say, five minutes with my renal doctor and I probably don't spend much more time with my GP on my diabetes; it's usually on something else. So that, I'm not taking up a lot of time and so that you would probably need to select your patients that were going to go to that combined clinic. You wouldn't want anybody who was very bad in one side to the other, you know what I'm saying, for the doctor to be having to sort of specialise. But, for one of the minimal patients in that area, you probably need to pick and choose your patients.

**Q: So, what some people have proposed is a combined clinic that's multidisciplinary with your educators there, your dieticians there, your various doctors there and depending on the patient's needs they might see all or one or two of the people there. That's what some people proposed.**

Merle: Yeah it sounds good to me.

Frank: Well I feel with this illness, dieticians should be participating a hell of a lot more, but then I suppose the patients daily dietary intake should be better regulated. I know that's difficult again, but I think something along those lines. For example, when you go to a hospital and you get in your cardiac, well there's no salt there, none whatsoever. So, if you had a dietician where they can control those sort of things, your health would improve I feel, because it's difficult to take it upon yourself. I mean, I can do it at times, but then it just breaks away.

**Q: So, you'd like that constant reminder?**

Frank: Yes, you need, like I said, every time I go to hospital, I lose weight, because I adapt to the portion control there and I keep saying, "This is fantastic." And I come out with that mindset and I keep going to that and I participate with my activities.

**Q: So, what you're looking for really is more dietary support, I think is what you're saying.**

Frank: Well I think so. Again, it's education or support or something that a lot of people can't do it on their own and I break down myself, even though I think I'm pretty strong at times. I'll drink coffee with no sugar, I'd rather have no salt whatsoever in my food than be too salty now and things like this. And I know people who are supposedly fanatical about their health and they load up with salt and I say, "You know, you told me you're looking after yourself and then you take half a kilo of salt." "Oh it's got no flavour."

**Q: So, Rob you looked like you were going to say something as well.**

Rob: Once again, it's content, for instance fresh tomatoes to make your sauce with Frank, do you know the salt content.

**Q: Sorry, were you going to say something about what Frank was saying or were you going to say something else?**

Rob: No, say something about what Frank was saying. Do you know how much salt there is in a tomato?

Frank: I haven't measured it.

Rob: You'll find that it's a very high natural content.

Frank: Yes, I don't disagree with that, but it's a natural product that's in the item. I know where you're coming from, but the thing is then is it doesn't have any other additives about it that preserve it so it lasts longer and all the rest of it. See, a bit of everything does you no harm because that's how the human race is developed. The whole problem is that things have been modified and as you said earlier, my father comes from a background where he experienced starvation and he didn't have shoes until he went into the army, well…

**Q: Sorry, can we steer away from the <inaudible> *1:49:46.3?**

Frank: Alright, but all I'm saying is…

**Q: And the preservative and so on. I understand what you're saying. I understand what you're saying too Rob.**

Frank: Well you tell me, you don't agree that these additives, the preservatives are detrimental to society?

Rob: No, but look…

**Q: It's not my place to disagree with, it's a discussion.**

Frank: You're a professional, okay.

**Q: How about we finish this question and then we can talk about that, as we're in this question?**

Frank: Okay, alright.

**Q: So, things that we can improve, the perfect system, what else would people recommend? Was there anything else you'd want to say? Recommendations, things that we could improve the way things are done here at Hospital 1?**

Frank: I feel the way you can improve things is start before the problem actually develops. We're here today and all you're asking is how can we improve your services? You guys, as far as I'm concerned, should not be looking at how we can improve your services, but what can we do for your children so they don't end up like you?

**Q: Yep, we've definitely got that down. Sorry, Rose May.**

Rose May: Yes, when somebody like you are talking about is a little bit, what you are doing, whatever you are doing, but when we say to people, "This one no good for the diabetes," they say, "Life is too short, we have to eat it." But, that depend us *1:51:16.3 <inaudible>, you know. Life is too short, but we have is no good for the diabetes, we have, we don't have to eat it, but when we say that bit often, we don't say if it's good or not good, you see. But just sometimes because they know you know, because you are telling the right thing. They tell you, "Life is too short, you have to keep going." That's I can't understand sometimes. We have to listen to our self too, not the dietician and apparently might get diabetes before I get 40 and now I are 80, but my diabetes is eight, 11; eight, 11, all the time. But, I listen what the doctor says to me. I do and look like I have to do my test, blood tests, whatever, but I listen to them, what they ask to do, because the urine, you don't have to eat 10:00 O'clock in the evening. In the morning, you just got to the toilet and the second, you know wee you put in the bottle and you bring it for thing, but he said to me, "You start living well too?" I said, "Yes, of course I have to." He said, "If I want to improve my health."

**Q: So, I suppose what you're highlighting is the importance of listening to the doctors ask. So, has anyone got any other points they want to make in terms of how we can improve the health service? Like, I take your point about prevention. Anyone else? Merle, Rob? No?**

Rob: I have points, but I keep get the same stumbling block every time, that doctors can't chisel it on your forehead in letters of fire that you shouldn't eat this, this or that or in such quantities.

Rose May: They wouldn't do.

Rob: And that people are responsible for their own health and the one thing that seems to come across all the time is doctors are so busy trying to help people get better after they've got sick. But, the people themselves so often do not make any effort for themselves. How many times have you heard a diabetes doctor say to your patient, "Look, you really should exercise and you really should lose some weight." "Oh, but it's so hard doc. I don't like walking and I don't feel like doing any other exercise and I can't get on a pushbike."

**Q: So, you're highlighting the importance of the patient actually looking after themselves.**

Rob: Yes.

**Q: And moderation and I guess even you're sort of indicating a bit of what Frank was saying, I mean going further back, with that prevention, being healthy at the start and then you might not even develop.**

Rob: I live on 30% heart function. Right, my heart was so massive I lost 70% of my heart muscle. I still go swimming, I still ride a bike; at a slower pace, sure. I'd like to lose a bit more weight, but the only way I'm going to do that is to basically to reduce my intake portions to such an amount that my guts are not going to be functioning properly, because my blood sugars, I only have to look at food sometimes and it's gone through the roof.

**Q: But, I guess Rob, we totally understand that from the health profession perspective, but often we don't have a magic wand and we can't install motivation into patients.**

Rob: I know that, but that's what a lot of the patients expect.

**Q: So, I guess within those limitations, how could we do better is the question?**

Rob: I don't think you can to be honest. It comes back to the individual bottom line. Doctors, as Frank said can develop a better bedside manner or a better interviewing technique and be a little bit more at the level of their patients. And what I said before is I don't feel they try to withhold information from you, so much as they seem to think that because they're doctors, automatically they do know a little bit more than most I will admit, but automatically the patient should just take what they say as gospel, rather than the patient say, "Alright, you said I should do this, but do you have any suggestions how I should do this?" And that's usually just glossed over. "Oh get more exercise." Yeah right. "Eat less food." Yeah right.

**Q: So, what you're saying is like more of a partnership and problem solving, rather than just giving information and that?**

Rob: Yes and dieticians are wonderful, but once again the patient walks out and goes home, sits down, afternoon TV, a cup of tea or a cup of coffee and a couple of biscuits, because that's what they've always done. They forget that those couple of biscuits have got enough sugar in them to blow them out of the water for the next six hours. Take insulin before or check your blood sugars after you've had a biscuit, oh no. One, quite often they don't get told to do that. They might get told to check their blood sugars before their evening meal, but they had those biscuits at 2:00 or 3:00 O'clock.

**Q: So, I guess you're going back to more education?**

Rob: Yes and patients responsibility.

**Q: Merle, have you got anything?**

Frank: The doctors can't help it.

Merle: I think patient responsibility is a big thing. I mean we don't do anything unless we want to do it and I think it's the same in lots of areas until we come to the point of wanting to make a difference and do something about our condition, then all the education and all the talking from the doctor will just go over our heads, until we actually come to that point of saying, "Well I really want to do something about it." My doctor became quite firm with me and said, "You need to reduce your portions and you need to exercise." I can't walk because of my back, but I've got an exercise bike and I use it. I have reduced my portions and if my sugar goes up too much she says to me, "You're eating too much" and I says sometimes, "Yeah, alright, I can't eat any less," but I probably can. But, because I'm wanting to listen now, the responsibility is mine, not hers, but the responsibility is mine and if I don't want to take any notice of that, then that's up to me and no-one can do anything about it, so really the responsibility is mine to do something about it and to do it. Now I have lost weight through doing that, so I've proved that that's true and I know that, I mean I've known it before, but I've lost weight before then I've put it back on again, but I'm a bit more determined this time. But, okay until I'm prepared to take responsibility for myself and my own condition then all the other things just get blown out of the water, that's how I feel about me.

Frank: Replying on that Merle, I know of a couple of friends of mine that have passed away through the point of that the doctor said, "Okay, you're not looking after yourself; don't come to see me anymore." But, all I'm saying is, a lot of people say, "Oh that's not fair that the doctor said that to those patients." But, if they were educated to try and look after themselves, it's like you're saying it's my responsibility, but okay, but you come here to get assistance. So, it's okay for you to say, "Well I'll do what I want with myself," but then when you're not feeling well, you want to be looked after. And that's where I find, that unless people are kind of educated to make them understand, well if you want to be looked after, you've got to look after yourself, not that if you choose, because otherwise that's a form of abusing the system. And the general taxpayer's paying for this service and the government's screaming that they haven't got enough money here and then we've got this issue about that we're waiting too long. Well if people were healthier, we're going to get less people in the waiting rooms.

**Q: So, are you saying that-- sorry I'm just trying to put... Are you saying that the doctor said..?**

Frank: Merle said, "Look it's my responsibility to look after myself if I want to." She said, "Her own doctor had to be firm with her, because often she sort of either she wasn't taking notice or just wasn't going at it hard enough or whatever." And that's fair enough, everybody's entitled to do what they want, but the thing is can you walk down the middle of the road because you feel like walking down the middle of the road. No you can't because you're going to get run over and the same I feel with you've got a responsibility to yourself, but you've got to feel a responsibility to your fellow man, because the services have been overused unnecessarily. Because, McDonald's wants to sell their soft serve which is made out of rubbish and yeah because it's only 70 cents, but for her, for me, for Rob, most of the people in here, it's not good for us to have.

Rob: But nobody twists your arm.

**Q: So, for those people who don't take responsibility, what do you propose we should do for them?**

Frank: Well put them out to pasture. When you drop dead, we'll give you a funeral, because…

**Q: Sorry, are you saying the doctors should say for those people who refuse to take responsibility, they shouldn't see them, is that what you are saying?**

Frank: Well, I'm not saying you shouldn't see him, but what I mean is when people, you've been told and told and told. It's the same as a person or an infant, you say to a child, "Listen, don't touch them because I'm going to smack you, don't touch them because I'm going to smack you." And you eventually smack them, because they're not listening to you. Well it's the same with an adult. I feel, yeah you've got your own responsibility to look after yourself, but I don't think it's fair that, you know this friend of mine, he abused his life when he was young, a heavy drinker, bottles of strong alcohol. Well, he died from liver failure and he was only 62. Now, they wouldn't put him on the program for an exchange, because he brought on this illness in himself.

Rob: Self-inflicted illness.

Frank: Yes, it's self-inflicted and he said, "That's fair enough," and you know…

**Q: Do you think that was fair enough?**

Frank: Well, I think it was a bit cruel, but to some degree, yeah because he knew the faults of it, so look I'm speaking for myself and when I left school, I used to hang around with a guy who used to like to drink and all we used to do was get into fights and getting the teeth smashed in and all the rest of it. I never drank, I wasn't a drinker, but I used to at the end of the night carry these guys home. And I said, "Guys, this is not fun for me. I'll come out with you, but I'll come with my own car and when I want to leave, I leave." And I eventually pulled myself away from them. One of them died in a car accident. Two others had a serious car accident. They're in their 60s and they were still having operations from 40 years ago. Do you understand, because I can foresee, if they're going to keep going, they're going to hit a pole, injure somebody else or whatever. They say, "Oh how could you do that?" It's the same as…

**Q: Sorry, I'm just trying to understand what you're trying to say in terms of what people say. Like Merle's saying that in the end it's the patient responsibility and then I'm trying to understand what your trying to say?**

Frank: Well it is the patient responsibility, but at some times or that they're responsible for their own body, but because they don't have the understanding or the education behind it, they might say, "Well bugger this, I like to eat this cream cake and I'm going to eat it." But, they're doing harm to themselves firstly, but they're doing harm for everybody else as well.

**Q: So, how do you propose to enforce it?**

Frank: Well I don't know; that's a difficult thing. Maybe there should be a penalty, a fine. Like, you're speeding up the road and you don't intend to speed, but sometimes it's the same as they produce the cars that have got the power to speed.

**Q: Well no, but I think what Merle's saying is and what Rob's saying as well, is you can educate as much as you want, but if they don't want to do it, they don't want to do it.**

Rob: Exactly and there is a penalty, you die younger.

Frank: Yeah, but they don't realise that until they die.

Merle: I can take the opposite side if I wanted to. I'm not prepared to, but I could say, "Okay, I am 75, I have lived my life. I can just eat, drink and be merry, what does it matter? I've done the damage now." But, I don't want to do that, I'm not as negative as that.

Frank: Well, this is what my friend did, who was 55 and dropped dead.

Merle: But, I could say that and again it's what I decide to do.

Rob: But then you should not patronise the health services.

Merle: Well then I probably wouldn't. I probably wouldn't go back to the doctor again. I would just eat drink and be merry and go with the consequences. I mean I would do my own readings, if they go too much I might adjust myself a little bit and all that sort of thing, but I wouldn't necessarily go to the doctor and taking all that time.

**Q: Okay, we do need to close up as well.**

Merle: Yeah I need to go, my friend was ringing.

**Q: Any final comments before we close up?**

Merle: Thanks for the opportunity.

Frank: Yes.

Rose May: Thank you very much.

**Q: No, thank you for coming everyone.**

Rose May: Yes thank you.

END OF TRANSCRIPT

**Focus Group 2**

**Q: Okay, well the first question I had for everyone was, based on your experiences; so obviously, you've had some experiences with the diabetes services and perhaps some of the chronic kidney disease services. Based on your experiences, what would you say are some of the weaknesses of the diabetes and chronic kidney disease services here at Hospital 2?**

Jay: Jay here, I can't say anything about that with this hospital, because my diabetes services are at Hospital 10Hospital, but my service there is very good. I can't fault it.

**Q: And what about for the kidneys?**

Jay: Well kidneys I see Dr H here-- Professor H here and he sees me on a regular basis and he keeps me in check as to what's happening, because I have to have my normal blood tests the week before I come and see him.

**Q: And that's all good? There's nothing..?**

Jay: Yeah, yeah I know what's wrong and he just keeps it in check.

George: George. The service is alright. We get to see Dr T and the doctor-- I've forgotten his name, the name of the - what's the name? Anyway…

**Q: Your renal doctor, yes.**

George: And then checks the blood, I do the blood test and like I do the blood tests for T.

Mary: I'm Mary and I see my GP for my diabetes. She hasn't sent me to anybody else and with regards to the kidneys I see Dr Singh and I've been seeing him six-monthly and I just have blood tests before I see him and he's been happy so far with the way things are.

**Q: And you've been happy with the kidney disease services?**

Mary: Yeah.

**Q: So, you've known no weaknesses, no things you think need to be improved or anything like that?**

Mary: No, no I think it's been alright.

Warren: I'm Warren. I come every three months to medical centre to see Dr T. Now he's very good doctor and I don't have any problem and I see Dr W for kidney. Every six months I go and see him and he told me he can keep me going for the rest of my life, you know and I'm quite happy with that, you know.

**Q: So, no complaints at all?**

Warren: No, complaints.

Betty: Yeah, I'm Betty. I come through to hospital for the Professor D for many years and she's looked after me all that time.

**Q: That's for the diabetes or the..?**

Betty: For the diabetes, yeah. And then after, when she's left, they send me through to the Dr T upstairs and she's always checking me up every three months or every six months, depend on appointment. And one day, she sent me to have x-rays to see if my kidney and it's a little bit affected like it is. So they don't give me any tablets for kidneys or anything like that at moment, no I'm not taking anything.

**Q: So, are you happy with the..? Any weaknesses or..?**

Betty: Weaknesses, yes I have a little bit. I feel a little bit weak sometimes; sometimes, not always. And I come to the hospital for one week, because I havw too much fluid to my legs and they give me some injection from here, so they take the fluids out, but still I have to be careful a lot; what I eat and I continue to go to them every three months.

**Q: Are you happy with the service that you're getting for your diabetes?**

Betty: Oh yes, yes.

**Q: Anything that you think are weaknesses or..?**

Betty: I'm happy, but before they not send me to have x-rays to check my kidneys or to check my heart or to check anything. Nothing about that, so I'm not taking tablets from there, but still I have to drink only one litre of water a day for my kidneys, but I have a lot of sore up my legs, fluids a lot and I don't know reason where they come from.

**Q: Giuseppe or Antonio, how are you?**

Giuseppe: Giuseppe here. Well, I'm happy with the Hospital 2, because any trouble I have, they fix it for me. I don't have problem; good service, good everything and I go to my GP every three months and I get blood tests, they send the report to Dr F. Dr F is that impressed, I saw her every six months and then every year. Actually, I saw her about a month ago, but the diabetes, that's up to me; what I eat and what I'm doing. When I check my blood test, if your blood is a bit high, I know I've got to eat less or get moving. Well I am the doctor for the blood <laughter>; for the diabetes <laughs>. Because, if you stuff yourself-- I know when I go to have a pint or something the blood test goes higher. I can't help that and I worry about it. When the other pains go, I go back to normal with the table, well I control myself actually, besides the doctors. That's all I can say <laughs>.

Antonio: And what more's to say. I have for 20 years the diabetes, because they found it by accidentally other, I was a little bit ill and something and then I carried on with the tablets, but I look after diet a little bit as much as I can, but still play up.

**Q: And are you happy with the diabetes and chronic kidney disease services that you get at Hospital 2?**

Antonio: Yeah, yeah.

**Q: Any weaknesses at all? Any things that could be improved?**

Antonio: No, no it's okay, that is.

**Q: So, it sounds like everyone's happy with the service they're getting for their diabetes and chronic kidney disease?**

Jay: Yeah, well it's…

Antonio: No it's okay, it's individual.

Betty: Service is alright, I'm happy.

Antonio. It's individual.

Giuseppe: Actually my diabetes, the doctor said to me come out from stress when my mother my died, I got a shock, I got stress and that's when I started having the diabetes. That's what the doctor says.

**Q: So, some people from other hospitals, when I ask them a similar sort of question, some of the weaknesses that they brought up were that sometimes they had to wait for too long before seeing the doctor or sometimes the doctor didn't communicate well with them and give them enough information or things like that. Could any of you guys identify with any of those complaints or you disagree?**

Betty: Well, the people if they know what the diabetes mean, like I didn't know what that mean that mean, the diabetes. It was kind of annoying and we have to check, even if it's young or more earlier, so we know what--We start more earlier perhaps to diet or to check the doctors and he must send tablets to get rid of it, but you know, we don't know what kind on damage they do later and we find out…

Giuseppe: Well, that may be that when the sugar down…

Betty: Yeah, yeah.

Giuseppe: …you say, straight away you've got to put something in your mouth or you might collapse.

**Q: I understand.**

Giuseppe: That's when it's down. When the sugar is high, you get a headache <laughs>.

**Q: So, can anyone identify with those complaints at all?**

Giuseppe: Well that's what affect me, I don't know anybody else, but. And then when this happens, I know what I've got to do <laughs>

**Q: Thanks for that Giuseppe. So, can anyone identify with any of those weakness that I spoke about that other people mentioned at other hospitals, like they had problems with waiting for appointments or waiting times to see the doctor or poor communication between the doctor and yourself. Does anyone agree with those weaknesses? These were weaknesses that people mentioned at other hospitals. Has anyone experienced that here at Hospital 2 or no?**

Mary: No.

Betty: No, no.

**Q: Other weaknesses people mentioned were sometimes they had too many appointments for too many doctors and they ended up just spending a lot of time in the hospital. Can anyone identify with that?**

Giuseppe: No, not me.

Betty: No, because…

**Q: No, not Giuseppe, not Betty, no.**

Warren: Warren and no, I haven't come across anything like that.

**Q: And other weaknesses other people mentioned were sometimes they get conflicting advice between different doctors. Can anyone identify with that?**

Jay: Jay here. No, I've never had that happen to me, because usually the person that I see gives me the right information, which doesn't conflict with what my GP tells me.

**Q: Now the next question, is it's easy for you guys to get the diabetes and chronic kidney disease healthcare that you need? So, do you guys find it easy to get the healthcare that you need for your diabetes and chronic kidney disease?**

Mary: Yeah never a problem and Mary here, I just go along to the GP yeah, but I haven't really had any problems.

Jay: And Jay here. I've had no problems with my diabetes, because I've been one now for about 16 years and it's only since May that I've had to go onto insulin and other than that, I've had no real hassles.

George: George here. Sometimes, when you come o the doctrs and the one doctor, who I was given tok one doctor said , "It <inaudible> *0:11:06.0 and they don't whether or not and <inaudible> *0:11:11.9 there, say "Why did they give it to you ?" So, sometimes the kidney doctor also say, "Don't take it" and your heart doctor says, "Take it." So, we don't know really what <inaudible> *0:11:28.7

**Q: So, sometimes you get conflicting advice between…**

George: Sometimes, not <inaudible> *0:11:34.0

**Q: …your kidney doctor and your heart doctor, is that what you're saying George?**

George: Yeah.

**Q: So, for everyone else, is it easy to get the diabetes and chronic kidney healthcare that you need, in terms of is it easy to access it in terms of location, cost or waiting times for your diabetes and chronic kidney disease?**

Mary: Mary here and it's fine, I haven't had any problems. They just give me the appointment and I turn up. I just drive over here; I come by car all the time, so I don't have to do it by public transport. If I had to do public transport, it'd be a problem.

George: You have to pay for the parking, George here, you have to pay for parking, so I've had problems parking here.

**Q: Sorry did you say you have a problem with parking George?**

George: Yeah most of any of the time, I have a problem with parking here.

Jay: Yeah Jay. The parking has always been a problem here. I come over a lot with public transport, but sometimes I can sort of sneak some handicapped parking, because I've got my sticker, but the big bugbear with the parking here is that parking station across the road, because of the locals, they can't build a multi-storey car park there. The locals hit it on the head. They just want people to park out the front of their house, it's stupid.

**Q: So, is the problem with parking the cost or not enough parking?**

Mary: Not enough parking basically, I don't-- I go down the back.

Jay: Me too.

Mary: Yeah <laughter> although at the moment, there's not so much disabled parking down there at the moment, because they're doing some work, so which I hadn't realised when I came across, but I was very lucky, I got parking.

**Q: Right, so Mary, Jay and George have also got problems with parking. What about you guys? What do you guys think?**

Warren: Yeah, sort of, so do I. I always come by car. Parking is the problem, I suppose. Sometimes I spent half an hour to look for a place to park, you know and I was driving around and looking for a parking spot.

Jay: You use more in petrol than you do in parking fees.

**Q: So parking's also an issue for Warren, what about the rest of you guys? What about you Giuseppe or Antonio, any issues with parking?**

Antonio: No, I can’t do that for parking.

Giuseppe: Well for parking, now I found that where the land is heading down here, before they used to go to work in there and after finish work *0:14:01.6, you can park in there for six cars. Otherwise, I go right down on the bottom of the street. I don't mind the walk, because usually I go four Ks a morning, but otherwise it's hard for parking, naturally. Besides, it's three hours a park, depends on what you come in the hospital for. Sometimes, you've got to wait two hours before you can see a doctor, because you're meant in emergency, oh gees. If you've gone on time, you can take a pillow with you and sleep <laughter>.

**Q: So Giuseppe, you're saying so sometimes you're waiting two hours for the doctor, that's only in emergency though. What about when you're seeing the doctor for your kidneys?**

Giuseppe: My doctor, when I've got appointment, well that could be ten minutes, could be an hour sometimes because it's busy, if he had some emergency or something, but otherwise I got no problem with my doctor. I got Dr F, no problem for appointment. I got Dr [Newey]-- Professor Newey actually, for the stomach, I've got no problem for appointment. And I used to have it, because I used to have it, because now, I've got no more [stone] *0:15:13.3, Dr Louis T, no problem for appointment. But, if you come in the emergency hospital, because I come a few times <laughs>, but you can't help it, because it's right there, sometimes it's two or three hours, you can't complain.

**Q: Well, what do you think Antonio or Betty about the parking or..?**

Antonio: Parking, I've got no problem.

**Q: ..or getting to Hospital 2?**

Antonio: I've got no problem, because I know the area, so I turn around in the street if I can't find that [close] *0:15:49.6 and then I've got no problem. So, having to walk ten minutes is okay.

Giuseppe: That's it, I'm going to walk <laughs>.

**Q: No problems for Antonio, what about yourself Betty?**

Betty: I come on bus.

**Q: You come by bus?**

Betty: Yeah for centre of Hospital 2, much better, I take the bus and come here ten minutes.

**Q: So, we've talked about parking and getting here, seems to be okay with everyone and the waiting times do seem to be okay for the appointments, I'm gathering, but what about the cost- the cost of healthcare for diabetes and chronic kidney disease, how do people find that?**

Jay: Well Jay here, for me, I originally started coming here to Hospital 2 in the early days and I used to see Dr Carter, but when I went to see him some years ago that meant that I would've had to pay, because there was no clinic anymore with him in it. So, eventually my GP referred me over to Hospital 10hospital, because it is closer to where I live, but they look after me over there and it's all done in the clinic there and it costs me nothing. So, the only thing it costs me is community transport to get up there, because it's as bad as this place for parking and I refuse to park one to one-and-a-half kilometres from the hospital and have to walk there, because by the time I get there, I end up having to pop a couple of Endone for back pain.

**Q: And then for your kidneys, that's public too?**

Yeah, so here, Professor H, yeah.

**Q: And what does everyone else think about the cost for their healthcare for diabetes and chronic kidney disease?**

Warren: I'm Warren. I pay Medicare money for Dr W, I pay the excess as well, and what places are closer to parking you know. I go and see Dr W at-- just opposite the Canterbury Hospital. What do they call them? Medical centre, so that's ample parking you know, no problem there.

**Q: So, Dr W-- sorry I've forgotten Warren, excuse me-- so Dr W looks after your?**

Warren: Kidneys.

**Q: Kidneys, okay yeah.**

Warren: Yeah, Dr T look after my diabetic you know.

**Q: And Dr W's not based here, he's based elsewhere?**

Warren: I think he's got a clinic as well here and the medical centre, you know.

**Q: Here of in the..?**

Warren: Canterbury.

**Q: So, you see him over at Canterbury?**

Warren: Canterbury, because I…

George: See him over here, he comes to here too?

Warren: Yeah.

George: He comes here.

Warren: Yeah he comes here too now.

George: Yeah, I see him here.

**Q: Okay, but you see him over at Canterbury?**

Warren: Yeah, because I live Bankstown, you know.

**Q: And then you see Dr T here?**

Warren: Yeah.

**Q: So, the cost isn't an issue because of Medicare?**

Warren: No, the cost is nothing for me.

Mary: No, Mary here, I just sign Medicare whenever I come.

Warren: I do.

Mary: Yeah it's only your medications you've got to pay for until you reach the-- you know where they stop-- having to stop paying, but they're talking about stopping that, so.

Warren: What the Medicare?

Mary: No the…

Warren: Prescription?

Mary: Prescriptions when you reach a certain amount.

Warren: Certain amount?

Mary: Yes, because they've had petitions at the chemists to sign.

Jay: Yeah.

**Q: Oh okay, is that a labour or liberals doing that?**

Mary: I'd say present government, because they've had them there for a while.

**Q: Okay, take note of that for the election-- only joking <laughter>. What about you guys?**

Jay: It doesn't matter who gets in, they're all going to screw us around anyway.

Warren: Yeah.

Mary: Yeah, it's Tweedledum and Tweedledee.

Jay: They're politicians.

**Q: What about you guys down there, Betty and..?**

Betty: I will just with bus and I keep the ticket.

**Q: And the cost is okay?**

Betty: Cost, no, no, no, cost anything, because of the railway ticket, it's for a year, like every year around.

**Q: So the cost of healthcare is affordable?**

Betty: Like every year around.

**Q: You have a..?**

Betty: A year around, a ticket.

**Q: And what about the cost of the healthcare for the diabetes and chronic kidney disease?**

Betty: The cost for the dia..?

**Q: As in cost for the healthcare for the diabetes and the kidney disease?**

Betty: Oh I have the little card for the…

Antonio: Medicare card.

Betty: Medicare yeah, Medicare card and it not cost me much.

Jay: Jay here, the only cost really or the only cost as far as I'm concerned is either transport or the medications and I've only got four more to go and I'm on the safety net this year.

Antonio: I've reached it.

Jay: Actually my casettes for the blood sugar meter on script now and that adds up with the amount of scripts you use.

**Q: So, Antonio you looked like you wanted to say something.**

Antonio: I've reached my limit of what medication. I've got the-- What's the name of that?

Jay: Safety net?

Betty: Medicare?

Antonio: I'm a pensioner, so I've got the card] already, but…

Giuseppe: Concession.

Antonio: Yeah, yeah, but I reached my limit with the medication already, so for now I'm okay until next year <laughter>. And when you pay every time, it's a cost is fairly big for all medication. What do you do? It's a lot.

**Q: Oh so the cost is a lot for the medication?**

Antonio: Oh yeah, because it doesn't matter you pay cheaper, because you've got the card, but still about the amount you buy, it's quite amazing, it's too much money, but medication, you can't do otherwise, you have to take them.

**Q: Giuseppe?**

Giuseppe: I had a fight with the chemist in Hospital 2, because she said, "Do you want the cheap one or dear one?" I said, "What my doctor prescribed here and that's what I want." One day, she tricked me; she gave me the cheaper one. Cheaper one, it didn't work like the others and I got all a rash. I went to the chemist like that, I took my shirt off, I said, "Have a look, what do the tablets you gave me." "Oh sorry, sorry." I said, "Sorry, you can kill people to give the wrong medicine." And after that, they gave me the one that the doctor prescribed and they don't give me anymore. Why, they say they're exactly the same, but a different name? Impossible, because the other one, I took it for so many years, I never had any rash. They give me that one, I had rash, because they're not the same. See that's for the pay, once you reach so much money, well they give you two or three months without the pay, but it depend how much you cost, how many tablets you take and some tablets they're dearer than the others. Some of the government, they do not pay, you've got to pay full amount of money, that's it <laughs>, that sort of situation. When you've got something wrong with you, you've got to fight all the time. It doesn't matter what happens, you've got to fight all the time.

**Q: So, is the cost of healthcare alright for you Giuseppe? The cost of seeing doctors, the medication, is that all affordable?**

Giuseppe: For the doctor, I go with the Medicare, I don't pay nothing. Dr F, he said to me, "If you want to come private and be seen down stairs," he said, "You've got to pay. Otherwise," he said, "I'll book you upstairs." I said, "Book me upstairs, because I don't want to pay, I can't pay." <Laughter> What's so good? Instead I go on the first floor; I go on the sixth floor. It doesn't make no difference to me, the lift will take me up <laughter>.

**Q: Now, have you guys ever missed out on a diabetes or chronic kidney disease appointments before. Like have they ever clashed together or anything like that?**

Giuseppe: No.

Antonio: No .

Warren: No

George: no.

Jay: Jay here, I have missed, I think one of my appointments and for me that's highly unusual, because I carry my diary with me and I also use Google Calendar on the computer and it sends me an e-mail two days before my meeting, so I like to keep myself up to date, so I'm here for the vascular specialist that I've seen this morning. When I go home, I'll update that in the computer and that keeps me abreast of what's happening, because otherwise with the amount of things that I have to do, I forget because at my age, I have CRAFT disease as they call it; can't remember a flaming thing, some of the time <laughter>.

**Q: So, if any of you guys have missed out on an appointment, what was the reason why you missed out on appointments? Was it because the appointments clashed or you forgot or no-one's ever missed one?**

Warren: No, well this is one reminder of miss any appointment and also they are very prompt here in the medical centre. They, two or three weeks before they call you and make sure, your appointment will be that particular day, you know.

**Q: Is that for your private appointment or public?**

Warren: Oh public.

Jay: Yeah, they're very good like that here.

**Q: So, I know some of you guys don't see anyone for your kidneys at the moment or for your diabetes, but if your diabetes and your kidney disease appointments clashed, so the occurred at the same time, which one would you go to and why?**

Mary: I wouldn't have them clash, because I've always got my diary with me.

**Q: But, just say they did for whatever reason, if they did which one would you go to and why?**

Jay: Jay here, probably me, I'd make sure the diabetes first, because that's the one I've had the longest and I have to keep on top of it at the moment, because as I say, I've only recently gone onto insulin and I'm still feeling my way.

Mary: Mary here, I just see my GP for the diabetes, so I guess I'd go to the renal doctor first, because I could see the GP any time.

Jay: If you can get in. [laughter]

Mary: I always sit and wait.

**Q: What do other people think?**

George: George here and if I have to choose between diabetes and the renal appointment, I will go to the diabetes first.

Giuseppe: You live close?

Antonio: Uh?

Giuseppe: You live close?

Antonio: Yeah, not that far.

**Q: You'd go to diabetes okay. What about you guys down there, Giuseppe or Antonio, which one would you pick?**

Antonio: [Silviate amazing far dock] *0:27:20.0.

**Q: Sorry?**

Antonio: [Silviate amazing far dock] *0:27:23.3.

**Q: Oh no, no which one would you pick if you had a choice, if you had diabetes and a kidney appointment?**

Antonio: Oh diabetes of course, but I check all the time at home, which one is which. If I don't remember when they give me appointment, I ring them up after and I say, "I'm sorry, I've got another one there. Can you change me for next day?" And no problem fix that way. Otherwise diabetes goes first and I, especially when you've got a public you've got to make sure you attend that appointment, because otherwise if they cancel, you might have to wait a long time to be seen again. So that's first one.

**Q: What about you guys, Betty or Giuseppe or..?**

Betty: Oh for me once, I forgot to there's appointments and she's putting me off for three months. She says, "That's only I have." And from that time I've been and gone to my appointment.

Giuseppe: Oh there's one, I never come across both of the appointments coincide each other. If it is so, then I will go for diabetic, then going for the kidney doctor, you know.

**Q: Any particular reason?**

I think diabetes is really important, then kidneys.

**Q: Okay, let's move on. What are some of the things that you find frustrating about your healthcare for your diabetes and your chronic kidney disease? So, what are some of the things that you find frustrating about the healthcare that you get for your diabetes and chronic kidney disease?**

George: George here. I don't find anything frustrating.

Mary: No.

**Q: Mary's nothing either?**

Mary: No.

**Q: Nothing, you don't find anything frustrating about the health services that you get?**

Betty: No, it's okay.

**Q: Betty, okay yeah. <Inaudible> *0:29:39.9 Warren or Jay or…?**

Jay: Jay here, I don't get frustrated with any of it because I think the level of care I'm getting is 100%. I've never had any problems in this hospital at all. I've had two new replacements done here as well and last October, corrective surgery on my right leg, but I've never ever had anything I could complain about here. My service has always been top notch and so much so, that when I was here in April for three days with an infection in my leg where I had a skin graft, I ended up writing the NUM on six north, I ended up writing her a letter of thanks and congratulations on what her staff were like when I was in here. I believe giving credit where credit's due to the staff.

**Q: That's really good, yes. Anything you find frustrating about the health services with diabetes and kidneys, Warren or Giuseppe or..?**

Speaker: No, no, no.

Warren: This is Warren, I never come across any frustration, no and I found that they're operating there… they are…

**Q: Giuseppe or Antonio?**

Giuseppe: No problem.

**Q: No problem?**

Giuseppe: Never <laughs>.

Antonio: No the only problem was that…

Giuseppe: I never missed any appointment with my specialist, never. I'll write them down. <Interruption> If you missed or whatever, that's your fault.

Betty: Yeah.

Giuseppe: But the doctor's there to help you <laughs>.

**Q: Okay, let's move on. So, it sounds like you have only got positive things to say, which is good, so let's explore that a bit. So, based on your experiences, what would you say are some of the strengths of the diabetes and chronic kidney disease services that you have received?**

Betty: Well, not really.

**Q: Well what are some of the strengths of the diabetes and chronic kidney disease health services that you have experienced? What you've received here at Hospital 2?**

Warren: Oh this is Warren and I do see Dr T, every three months. I don't have a problem with losing weight. I put on some weight you know and when he found out and he arranged me physiotherapy and the dietician, to see them and bring it down my weight. So, they're doing okay now for me.

Mary: Well Mary here. As I say, I see the GP for my diabetes, see the renal, they're really just keeping an eye on it, because the function's gone down a bit, but not sort of dangerous, that I don't take any medications or anything. So, they're just really keeping an eye on it.

**Q: And any strengths about the services that you receive for your kidneys?**

Mary: Well, I just have my blood tests the week before and then I go along and he checks them out and says, "Everything's okay." So, I really haven't had any problems.

Giuseppe: And that's another thing, you go there and have a blood test and then you go back for the results <laughs> that's it.

**Q: Thanks Giuseppe. What does everyone else think?**

Jay: Jay here. I've had no problems at all with the kidney here, because Professor H keeps an eye on what I've got and he said that he'd "be able to keep me going for a long time yet" and I said, "No, no, I only want you to keep me going until I'm 80." I said, "After that, it doesn't matter," because my father died at 91 and he had dementia and I said, "I don't want to live that long." I said, "So, 80 will do me fine." And the girl that was there, the registra, she said, "Well that's about 12 years." I said, "That'll be it." I said, "You keep me going for another 12 years," I said, "I'll be happy. After that," I said "if I fall off my perch, I'll be happy." But, the strengths here, they just look after you and care about you. That's patient care with Professor H and crew are good. I've got no complaints.

**Q: Betty or Antonio, strengths of the services that you get from the diabetes and chronic kidney disease services here at Hospital 2?**

Betty: No, no it's fine. They check on everything, you know. There's trying to eat less and they give us to eat all the boring things so or something like this. Sugar completely down, there's three things, so that's okay.

**Q: Antonio strengths? What do you find good about it?**

Antonio: Good about it, to eat the right stuff and the right food. When you eat the bad food, that's when you get started here, that's when there's a problem. Start to go up and down sometime, that's when there's a bad diet. I look after myself as much as I can, but I lead a normal life, I'm not have a strictly diet. I'm still doing okay, I mean not 100%, I never do 100%. When you come to 60 years of age, you're always going to have chances there to go worst. Plus the problem with my eyes or whatever, but I don’t think it's not much of a problem, so yeah.

**Q: What do you..? Oh sorry George?**

George: I mean I feel the same. The same thing as with what, we don't know what other thing we would have got *0:36:29.3. If you can find the [courtesy] *0:36:38.2 now or the service not good, but I won't say I think it's no good.

**Q: Okay, what does everyone find particularly effective about the diabetes and chronic kidney disease services here at Hospital 2? So, what do you guys find effective about the diabetes and chronic kidney disease services here at Hospital 2.**

George: George here. Effective, I think that Dr T when I gave her the blood count, it tells you how much can go, can't go and then with her again.

**Q: So, what you're saying, Dr T helps to control your sugar levels and anything you need, he prescribes you other medication, yeah.**

George: Yeah.

**Q: Anyone else? What do you find particularly effective about the diabetes and chronic kidney disease services here at Hospital 2? So, Antonio?**

Antonio: I don't have problems.

**Q: You've got no problem? But what do you find effective about the service.**

Antonio: Because they look after you. They tell you what, if you've got a problem, they tell you what you can do, send you to a dietician or something you know, that's very correct. There's no problem, unless you want to look after for problems, then you've found it.

**Q: Anyone else?**

Mary: Mary here. I think it's just going and having the checks every six months, just to make sure that you're still keeping on an even keel and then if there is a problem, well then you'll know about it, maybe before it shows ups, before you actually feel it.

**Q: Anyone else? Warren or Betty or..?**

Betty: No, to me it's a little bit, if they see me, I still on the medicine, they affect me, my body. So, affect me a little bit, in fact I go to the doctors, so they be careful from that. They not give me too much strong medicine I think. And it's okay, they ask me if they <inaudible> if it worries me or anything like that and it's okay.

**Q: What do other people find effective about it? Warren? Sorry Betty, did you have something else?**

Betty: No, no that's okay.

**Q: It looked like you were about to say something else. That's alright, do you have anything?**

Betty: No, no, no I thought you asked me.

Warren: I didn't find anything bad that I can think of. Everything okay and every three months, they do a blood test, so according to that one, they increase the tablets, quantity up or down.

**Q: Okay, another question guys. What do you guys like about the diabetes and chronic kidney disease services here at Hospital 2?**

Antonio: It's the service, the service is not forgetting you, that's the one we like. That's pretty good so.

Jay: Jay here. I agree with what Antonio's saying about the service is second to none.

George: George here. The waiting period time is pretty good, they're not bad with the appointment.

**Q: The waiting periods aren't bad, yeah?**

George. Yeah, you have to wait for sometimes, maybe 15 minutes or ten minutes, never much more than that. The only thing is sometimes it gets busy and you get a lot of assistant doctors looking at you *0:40:50.1. So, I'm [not nobody really] *0:40:54.7 [what with] <inaudible> and the blood pressure is more.

**Q: How about you guys? Warren or Betty or Giuseppe, what do you like about the diabetes and chronic kidney disease services here?**

Giuseppe: The service is good, I don't have any complaints and what that I've found very good and they take serious about the procedures and Dr T is very good and he makes sure that everything's all working okay and Dr W also very good. He looks after my kidney's quite well.

Betty: Yeah, me I think it's-- but I like just to tell me exactly what I have, so I have to be careful, what I eat, what I drink something like that.

**Q: So, what you're saying Betty is that you like that they just tell you what you need to do, is that right?**

Betty: Yeah should really tell me exactly what I have, how dangerous it is, all the sickness, anything like that. If the high blood pressure goes high, it's happened there to you. For high blood sugar, it's going to happen there for you, because if we have that very high and we didn't know, it's high. Like, we have to know the truth to protect, that's only right.

**Q: So they tell you everything you need to know?**

Yeah, yeah, to me is that.

**Q: Antonio or Giuseppe?**

Giuseppe: Giuseppe, then I keep up with my GP regular and in the meantime, I'll go and see Dr Croft, she's in my specialist for the diabetes and my GP six- seven years ago, he said, "You've been diabetic, that's why," he said, "had the specialist."

**Q: So, you see Dr Croft here right?**

Giuseppe: Yeah, yeah.

**Q: So, what do like about that?**

Giuseppe: In the medical centre.

**Q: The medical centre here at Hospital 2?**

Yeah, yeah.

**Q: So, what do you like about the diabetes health service that you get here? What do you like about it?**

Giuseppe: Well, every time you come and the results from the blood tests, they know and they explain to me about the kidney function. That's why a couple of times, they changed me the tablets. They reckon that tablets they can affect the kidneys, but I had no problem, because they take urine tests and everything, every three months. They not find anything and the heart specialist, I go every six months, they put me on a machine, everything, perfect, but I go for a walk most of the time, I not feel anything usually, breathless or anything you know.

**Q: Has anyone got anything else they want to add about what they like about the diabetes and kidney disease health services here?**

Mary: Only you're just, you're being kept an eye on and yeah.

Giuseppe: That's it, that's it. I test it two or three times a day sometimes. A couple of times, every morning when I get out of bed, the first thing I do, I have a blood test. I tested the sugar every day. Sometimes, two times, it depend how I feel. If you feel like shaking, I know I have a blood sugar test and I know the sugars a down. When I've got a real headache or something like that, I know the sugar is up, but in the meantime, I make sure I test it. And the doctor does the best for you, but in the meantime, you've got to keep yourself, what you are doing.

**Q: I Agree, total, you're right there Giuseppe <laughter>. Okay now what impact has attending the diabetes and kidney services at Hospital 2 made to your health? So, what impact has attending the diabetes and kidney disease services made to your health?**

Antonio: Made me healthy.

**Q: Made you healthy, yeah**

Antonio: Well the tablets you take. If you took the wrong tablets, all the body is affect <laughs>.

**Q: So, Antonio says it's made him healthy, does anyone else agree or is there anything else?**

Mary: It just keeps you on the straight and narrow.

**Q: Thanks for that Mary, anyone else?**

Antonio: They try to look after yourself, but what else can they do for you?

**Q: Does anyone else agree that the kidney disease and diabetes has sort of kept them on the straight and narrow?**

Antonio: Me, yeah.

**Q: And at least made their health-- may even have preserved their health or made them better?**

Giuseppe: That's what I said, you'd got to look after yourself and properly. You see me, everybody not believe my age, but I am that old.

**Q: So, can I ask you how old you are?**

Giuseppe: Nobody believe my age.

**Q: How old are you Giuseppe?**

Giuseppe: Have a guess.

**Q: Sixty?**

Giuseppe: Yeah I wish I was <laughs>, 84.

**Q: Wow!**

Giuseppe: Do you see me. I feel like I'm 60, good job, a good everything. Yesterday I work in the backyard more than sit in the room, because I've got to plant some beans, but I keep myself busy working, I don't mind working. I've been working all my life, I don't mind. I've got to dig backyard, I put all of my veggies up and everything <laughs>.

Betty: So, spread the time, doesn't it, spread the time.

Giuseppe: Of course, of course. What's good is you sit on the chair, watching television or the television watching you.

Betty: Yeah, yeah <laughs>.

Giuseppe: That's no good. It's good for an hour, half-an-hour watch television…

Betty: You have to work everything [that you've got today] *0:47:38.6.

Giuseppe: But there's nothing good on television anymore. There used to be good shows and everything, good movies, now, all the cooking, cooking, cooking.

Antonio: For nothing.

Giuseppe: Cooking not good for us <laughter>.

Jay: Even pop starts don't look bloody good anymore <laughter>.

Giuseppe: Yeah.

Jay: It's a waste of money.

Giuseppe: Yeah.

**Q: It's alright. So Jay, we're just talking about what impacts attending the diabetes and kidney disease service has made to your health, that's what we were talking about.**

Jay: Well Jay here, the treatment I get has been very positive. They help me keep on top of it. The diabetes educator at Hospital 10they're very good with keeping a check on me, the same as Dr H and Professor H here, keeps an eye on my kidneys, so that is what keeps me happy.

**Q: And the other thing we've discovered is that I thought Giuseppe was 20 years younger than he actually is <laughter>.**

Giuseppe: Twenty-four.

**Q: Twenty-four.**

Jay: A bit of a charmer. <laughter>

George: George here. When I first came to Dr W, I think I had 50% function and what they gave me controlled it. It was great.

**Q: So, whatever the medication that he gave you for your diabetes…**

George: Stopped the deterioration.

**Q: …stopped the deterioration, yeah. Let's move on. Now we've certainly been speaking a lot about the health service that you guys have been receiving. Now I'd like you to, now let's start thinking about your experiences of living with diabetes and chronic kidney disease and having to look after. So, you're experiences of living with and looking after you diabetes and chronic kidney disease. In your opinion, what do you need to look after you diabetes and chronic kidney disease well? So, based on your experiences with living and looking after your diabetes and chronic kidney disease, in your opinion, what do you need to look after your diabetes and chronic kidney disease well?**

Giuseppe: You need the strength and good money.

**Q: Giuseppe, I go straight to the good money <laughter>.**

Betty: A little bit more exercise.

**Q: Sorry more?**

Betty: More exercise.

**Q: So, Betty says more exercise, yeah.**

Jay: Jay, I'd like to get more exercise, but with the problems with my knees and my back, that makes it very hard for me. At the moment, because I'm still getting a problem with my right leg, where I had an infection dressed, I can't go into a hydrotherapy pool, because with the wound coverings on, they won't let me. But, once that's all cleared up, at least I can go to my local pool on a regular basis, even though I can't swim, just get into the heated indoor pool and get and do a lot of walking, it'd be a lot better for me, but currently I can't. But, I find that since my car's been off the road, I quite often have to walk up to my local shops, which is probably about a kilometre or there abouts. By the time I get halfway to the shops, I feel like I need to pop an Endone, because I have problems with my lower back, so I'd like to be able to do more exercise, but I can't. I try to watch what I eat and I have running battles with one of my housemates about the amount of vegetables that I prepared with our meals, because there's three of us and we share the house and started off cooking lots of green and orange vegetables and that, because the dietician that I see down at R City Uni, says to me to cut out mashed potato as much as possible and have say, mashed sweet potato instead and I like lots of green veggies. "Oh you" David says, "Oh no, too much, too much." I said, "Well that's not what they tell me. The dietician told me I can eat whatever amounts I want of the green and the orange vegetables, but keep the potato et cetera, cut down," which is what I try to do, but I do try to keep down the amount of soda beans, but it's not always easy to do, because I've got to try and balance my meals out, because the educator has told me, to "Try not to go any more than five hours maximum without having something to eat." So, that's what I try to do.

George: George here, I think… my weight is making my back ache, yeah and ache in the legs (0:53;18.7)-- what I do is cut down on the meals I do- cut down on my meals that the you have, portions, like the portions.

**Q: Oh you cut down on your portion size that you eat?**

George: My portions, because of your weight. I will reckon I still have <inaudible> *0:53:46.6

**Q: Oh wow that's pretty good.**

George: And I feel that I'm better, but I'm better, yes

**Q: Okay, so what do people need to look after their diabetes and chronic kidney disease? Well, I think we've mentioned, I guess your own motivation and strength and having a good mind. We've mentioned needing to exercise and we had some problems with and we also mentioned diet as well and the importance of diet. What do other people think? What do they need to look after their diabetes and chronic kidney disease well or what are they doing to look after their diabetes and chronic kidney disease well?**

Mary/Angela: Angela here. I engage, do exercises. I go to Lilyfield. There's like help the aging, I think it's called and I go there for exercises and I absolutely hate cooking for one, so I've started buying that tender loving cuisine and that also governs your portion size, so it does make it that you eat less, yeah so I'm finding that a lot better.

**Q: Anyone else want to add anything?**

Betty: For me for diabetes, to try to knock out stress or anything like that or worry.

**Q: Okay Betty yes, so stress.**

Betty: Yeah to me, stress or worry. If you have stress a little bit or worry or something, they go off the high blood pressure and the diabetes and of course we have to eat less <inaudible> also, a few fruits or some drink with no sugar in it to keep it down. But, to me if some stress, some worries came, a lot of worries go by and I try to keep it more peaceful, more quiet, you know. If I work a little bit extra, I need to push myself to force myself, so that's why it's come, high blood pressure.

**Q: Anyone else want to add anything?**

Mary: Mary here, just sometimes stress isn't as easy as it looks to keep things on an even keel. There's always stresses in life. I've got a husband in a nursing home at the moment, because he had a major stroke and it's not easy.

Betty: Not easy yeah.

**Q: So, other people have mentioned <interruption> support from family, family support, having good support from their hospital or health professionals and also having adequate education as being important for looking after the diabetes and chronic kidney disease. Can anyone identify with any of those factors or does anyone agree with those factors? So, education, family or social support and also good support from their health professionals?**

Jay: Jay here. I agree with all I mean…

George: Yes.

**Q: And George agrees with everyone else yeah.**

George: I do [outside with the] *0:57:06.4 <inaudible>.

**Q: Sorry Jay, you…**

Jay: I had one of my housemates who was officially my carer and even though we come to verbal blows occasionally with food-wise, it helps to have him there, because I know that he's there if I need him, because sometimes I have problems trying to put my socks on. I have problems doing other things and whatever, but he's there to help me, otherwise I'll slap him <laughter>

Antonio: Can I ask you something?

**Q: Yeah, yeah.**

Antonio: Have we got long to go?

**Q: Sorry?**

Antonio: Because, I've got the car on a two-hour parking, I don't want to get booked.

**Q: Yeah, yeah, no we'll be done in another 20 minutes-- 20 minutes- half an hour, we'll be done, so...**

Antonio: Twenty minutes.

**Q: So, we'll be finished at.**

Antonio: I don't want to risk it.

**Q: I think we started at 11:00- something.**

Sarah: Oh we started

**Q: Yeah, we'll be done by one.**

George: At worst.

**Q: Yeah we'll be done in the two hours.**

Antonio: I've got to get to school and do the childcare.

George: Sometimes it doesn't pass here.

Antonio: What happens if I get booked?

**Q: Look, if you need to leave for your car, just leave if you need to. Okay I'm serious, if you need to leave.**

Antonio: I left the car 10:30, so I'm…

**Q: Okay, look if you need to leave to move your car, I don't want you to get a fine, so if you need to.**

Antonio: And there's no-one going to pay for me, so it's an extra <laughs>…

Giuseppe: Extra bit.

Antonio: …extra cost.

**Q: You know if you need to leave to move your car then…**

Giuseppe: Extra <inaudible> *0:58:58.8

**Q: …then leave when you need to okay.**

Antonio: If I can, then I will please.

**Q: Yeah, I think when you need to leave, just tell Sarah.**

Antonio: Well I've got to go now, because I otherwise will be a bit late.

**Q: Okay.**

Antonio: 12:30 is two hours and I don't want to…

**Q: That's fine, we're just still going.**

Antonio: Okay.

**Q: So, if you speak to Sarah and we'll just keep on going.**

Antonio: Okay, thank you very much.

**Q: Thank you Antonio. So, next question is what things make it hard for you to look after your diabetes and chronic kidney disease, so what things make it hard for you to look after your diabetes and chronic kidney disease?**

Warren: Oh this is Warren. I've found sometimes eating the food, getting the right amount and I don't want to eat more bread or rice, then we ended up doing the vegetables and other things, so that part is pretty much what I found difficult you know, to take the quantity of the food you know and that's it. So, if the quantity's, it's not very good; then sometimes if I cut down a little bit, I feel hungry in the middle of the night. Then sometimes I have to go back and some more you know.

**Q: What do other people think? What factors make it hard? What things make it hard for you to look after your diabetes and chronic kidney disease or..?**

Mary: Oh Mary here. I find stress mucks me up, because when I'm stressed, I eat and that really is a big problem.

**Q: And Jay's agreeing?**

Jay: I'm agreeing, Jay's agreeing, yes. I mean stress can cause you to have too much cholesterol and have coronary bypass, like I had 19 years ago. Because, before I had this stressful situation, my problem with cholesterol was probably zero, but the company where I worked, I was at a sales office and had a very high stressful job, I ended up going in for quadruple bypass and I put all of that down to the stress of the job. The way I am these days, stress does cause a part of it, because my housemate David, I was stressed out worrying about him for about three years, because of his problems with alcohol and finally he's on the wagon, so that's reduced a lot of my stress, so. But, I agree with Mary, that stress is one of the things that I've got to try and keep on top of to make it easier to manage everything.

**Q: What about anyone else? Any other factors which make it hard for you to look after your diabetes and chronic kidney disease well? So, we've mentioned the food and sometimes once the food mentioned about stress. And we've mentioned that link there, that sometimes stress, especially with some people can make them eat more or perhaps they comfort eat a bit. Any other factors that anyone wants to mention?**

Warren: This is Warren. The doctors all the time tell me to reduce weight. I do walk, but doctor, he far you know.

Giuseppe: You stay away from the table and then you lose the weight <laughs>.

**Q: So, stay away from the table.**

Giuseppe: It doesn't matter how much you walk, if you still eat, you don't lose the weight.

Betty: Well I think for stress…

**Q: So, Giuseppe has to stay away from food.**

Giuseppe: And that's the only way. Look I've got a son, every time you look around, he's into something, I can't make him stop, because he’s a bit backwards.

Betty: He's used it. Yeah if your body's used it.

Giuseppe: Yeah, he got to eat, he got to eat; he got to eat.

Betty: It's like a clock, but other times 12:00 and we can't do that.

Giuseppe: It doesn't matter what you do, you can't rock it; you can't rock the [food] *1:00:24.8

Betty: Exactly or you wake up at 6:00 O'clock in the morning and you wake up 6:00 O'clock every day, at exactly the same minute, they wake you up. The body is like this. <Giuseppe laughs>

Giuseppe: Me it's different.

Betty: It will become a little bit less and less and less and less eating.

Giuseppe: We had nine in family.

**Q: Sorry, let Betty finish please.**

Giuseppe: Oh alright.

**Q: Sorry.**

Betty: It becomes less and less eating, so you use it to become less eating and to me it's 5:00 O'clock, 6:00, stop, nothing. After that, it's one fruit or one glass of water till the morning. And I'm used to it now, but before of course I work, I come late; I eat late, no good.

**Q: Sorry Giuseppe you were going to say something.**

Betty: Sorry Giuseppe, I cut in.

Giuseppe: No that's alright, that's alright. Depend vary it years. I have one biscuit in the morning and a cup of coffee, but at 10:00 O'clock, I feel I want something. I'll have another little cup of coffee and one biscuit. Now I have a lunchtime, I eat a little bit extra at lunchtime, because that's my doctor advised me to eat like that, then nothing all day until night time. Night time, I have a very light meal, very light, because when you have light meals at night time, you sleep better.

Betty: Better, yeah, yeah.

Giuseppe: You sleep better, because if you fill the guts, you not sleep better. Otherwise between meals, I not touch anything, nothing at all, only drink water.

**Q: Well, you sound very disciplined Giuseppe <laughter from Gisueppe>, so that's… It's not easy for everyone to be… you're lucky. You're lucky, then.**

Giuseppe: No, no, no as I say, I've got a son, <inaudible 1:05> who likes to eat. I can't stop him.

Betty: Pensioner good.

**Q: So, other factors people have mentioned that made it hard for them to look after their diabetes and chronic kidney disease in other places-- Well people mentioned diet. People also mentioned a lack of support, having a lot of other illnesses, people said impacted on their ability. Some people also mentioned that the disease itself also made it hard, just the consequences of the kidney disease or the diabetes. Can anyone identify with any of those?**

Jay: Jay here, yes I can. Multiple medical problems, yes, that does to me impact, because I have the problems as I say with my back, I've had my knees done and I also have bronchiectasis, so I have problems with coughing a lot of the time and that's stressful on my body and makes it a bit hard to do things. And all of that's because of other people smoking.

Giuseppe: Excuse me, the stress depends how you take it. I speak by experience. I took a contract with the water board. I had a 20 men work for me, everything went wrong, even I lost my house. I went broke completely. Well, that you know, call a strain. Every day something would go wrong, every day something would go wrong, alright.

Betty: I think the body's got <inaudible> *1:06:51.2.

Giuseppe: I said, "Okay." Then I'll find a way to start in business again. I'll finish my own business, I have to move a business, you know, clear land and maybe build dam for farms,. And I used to work in Liverpool and from Liverpool, I went to South Coast, near Milton, Ulladulla. For there I work for four years. You know what I had in on top of me. This bloke he had shed for the [techie] *1:07:24.2. He can go underneath it with the front end louder to take the manure and he had these tablets like this, where they-- otherwise we can go there. On top of me, I had a couple of sheets iron. When dew was in the morning it drip on top of me, but I had to decide there, becasue that's the way my mind was to build myself up. A couple of friends of mine-- because I brought them on from this-- do you know what they said to me? They said, "I can't believe you." He said, "If you ask me, I will go jump in the R City Harbour Bridge or disappear from R City." I said, "I know." I said, "You work very hard, you lent me some money, I've got to pay you back." That's my way to do it. That's one story. When I talk to a person, he's in the Paddington Market selling the fruit; sell there. When everything goes slack, you feel as though, what do you do? You light a cigarette.

Betty: Yeah usually.

Giuseppe: Walk around you light a cigarette, see.

Betty: Stress, yeah.

Giuseppe: That's stress.

Betty: It is stress, yeah.

Giuseppe: But, you've got still to try to control to beat the stress, that's what I did. That's, as I said by experience, that's what I did. After years-- That's a long story, I got married. My marriage lasted six months. After three months, she had to go in the hospital. Six months, she had the baby, three months premature. Five days after, she die, left me with the five-days old boy, born premature. That's strength.

Betty: Oh yes.

Giuseppe: I had to-- my get the strength for myself to be strong, upbringing my son. When he was 18 months old, he got a brain convulsion. I nearly lost him. Even had a fight with one doctor in the hospital, because he told me I can take him home. I said, "No, I can't take him home, because I know my son." When my son he lay down on the cot, he jump. My son, he can't jump, because of they give him a needle in the back. Anyway my sister-in-law looked after my son. I had to leave my job, because after that, she's scared to be by herself. Anyway, I bought a little shop here in Hospital 2 West, to be there in case my sister-in-law need me for my son. And then after five years, because my sister-in-law got sick a few times, she had to go into hospital and my brother said about, "The only way- only solution is for you to get married again." I got married again. Well I got a nice daughter and she got two kids. We are nine in the same house, because after we build a new big house. You think it's most strange, you feel, if the kids scream or something, you think it's strange.

Betty: It happens, it happens like that.

Giuseppe: But, when the kids start to scream, I walk away, come back again in an hour, that's all. But, I still enjoy taking all my grandchildren with me. My son is not well, because he's backwards, you know. After the temperature, the brain <inaudible> *1:11:12.8, he done damage to the brain a bit, I know what I had and I'm still happy.

Betty: What do you do..?

Giuseppe I find my life is ok <inaudible> *1:11:22.6 I <inaudible> there because the one that got the meat on the table, they’ve got no strength they just got to shit and eat. I have to prepare my meals <laughs>.

Betty: The life is cruel, that's the life

Giuseppe: Yeah, that's life, that's life.

**Q: Sorry to hear Giuseppe.**

Giuseppe: That's life.

**Q: I'm really sorry.**

Betty: How the life is come.

Giuseppe: And you've got to consider somebody else, in case they are the same condition, but a lot of people, no they not consider, so bugger, yeah, no. That words, I not use, bugger, bugger, no.

**Q: So, thanks for sharing your story Giuseppe. <Giuseppe laughs> So, I think so we did mention that stress can affect the management of your diabetes and chronic kidney disease and I think we also mentioned that having a lot of other illnesses can. And other people mentioned lack of support, do people identify with that, lack of support from family?**

Warren: I've got family support.

Jay: Jay here, I have no problem me.

Warren: I think family support is very important. Otherwise it produces stress on you.

**Q: Thanks for that...**

Warren: Warren.

**Q: …Warren, okay. Now, other people also mentioned having like depression and stuff like that can affect their ability to look after their disease or also perhaps having trouble to adjusting to the fact that they've got diabetes and chronic kidney disease. Can anyone identify with those?**

Giuseppe: No, I don't have nothing of that.

**Q: Giuseppe you don't, what about Betty or George or Mary or Jay?**

Mary: I do take something to give me a bit of a pickup, but I think it's a whole life, everything that's being going on that affected that, but I find walking a problem, even though I do do some walking when I go to the exercise place, because I do have a lung problem and that makes things, a little bit difficult too, because I get very out of breath.

George: George here. I have to say <inaudible> *1:13:34.8 I'm bipolar, but I haven't had problem. Probably the last depression I got five years ago.

**Q: Last episode of depression, five years ago <inaudible> *1:13:45.5**

George: That's when I had to sell my business and <inaudible> *1:13:47.9 and go back to the start and then from then on, I was just a little bit sick then and I said “I must find a psychologist”, and they found there was no actual depression with the psychiatrist.. but within five years I developed DM and renal problem.. *1:14:10.6

**Q: So, you said that over the last five years, no issues with depression, because you saw a psychologist, but the diabetes and chronic kidney diseases developed right?**

George: That's right and I didn't know before that I had Diabetes, I didn’t know I had a problem

**Q: Betty have you got anything you want to add in terms of anything which makes it hard for you to look after your diabetes and chronic kidney disease?**

Betty: No, but to take care of about-- my husband is still at home and help me a lot, but sometimes I want to go somewhere, my husband come behind me to look after it, because the problem is for me, I have fluid a little bit for my body inside, a lot of fluid. I don't know where these problems came from and when I walk outside, I have the same problem as Mary, I can't breathe much, when I walk, so there's my husband's come and walks with me.

**Q: So for you, having your husband to support you is very important?**

Betty: Yeah, yeah, for normally my husband's there.

**Q: Let's move on a bit. Now this question is a bit of a fun question, but imagine if you became director of a hospital and you had power to change things about the diabetes and chronic kidney disease health services. If you were going to design the perfect health service for diabetes and chronic kidney disease, which would meet all your needs, what would it include and what would it look like? So, I'll just repeat the question. Imagine if you became director of a hospital and you had power to change things about the diabetes and chronic kidney disease health services. If you were going to design a perfect health service, which would meet all your needs, what would it include?**

Jay: Jay here, I'd probably shoot every state and federal politician that has <laughter> anything to do with healthcare and steal the money out of treasury and build the best centre that I could. <laughter>

**Q: And what would that look like? What would it have in it; <laughter by others> especially for addressing diabetes and chronic kidney disease?**

Jay: It would have everything that endocrinologist would want to treat his patients. I don't know what all of that would be, but he would have unlimited funds. <laughter>

Mary: Wouldn't that be lovely?

Jay: It would yeah. I mean, government tries to screw us around now, with what they do, but when you've got a pay-- I've heard good and bad things about other hospitals, but with this one, I think the renal unit here, that Professor H has is second to none around R City. I like his new centre over there on the third floor of the medical centre, but I would just make sure that both types would have what they needed. I'd get the money from somewhere.

**Q: So, what do other people think? So, other people have mentioned things like better education, cheaper parking, some people even mentioned a combined clinic or regular pharmacy contact, so what do you guys think?**

Mary: Mary here. I think a combined clinic would be great; then you'd only have to go once.

Jay: Yeah and Jay here. I think better education, because from what I've read and I go to my local diabetes support group at Hospital 10and I think better education is needed, because there are a lot more people out there with diabetes that don't know they have it, than those of us that do have it. And I think when people know what to look for that they're educated enough as to what to look for, it would be easier for them to know when they're suffering from diabetes and they could do something about it, they could hopefully nip it in the bud early.

**Q: Sorry, I'm going to go off on a rabbit trail here. Taking a step back, do you guys, while we're on the topic of education, have you guys found that you've received adequate education from the health service about your diabetes and chronic kidney disease?**

Jay: Jay here. Yeah, I feel that I'm getting educated enough, because if I've got any questions, the educator at Hospital 10tells me what I need to know or she'll make suggestions. And then when I go to our support group-- of which I'm going to become secretary next week.

**Q: Oh is this diabetes support group is it?**

Jay: Yeah, yeah. Yeah, I put up my hand, but even though I won't be at our AGM next week, I put up my hand, because I'm going to Brisbane on Saturday week, so but no we bandy a lot of things around there. We've had a psychologist there talking to us. One of the nurses takes…

**Q: At the support group is it or..?**

Jay: Sorry?

**Q: At the support group or..?**

Jay: Yeah, yeah, yeah, she's from the diabetes centre at Hospital 10Hospital, she's come along. Last week, I went to see my podiatrist at the medical centre at Hospital 10Hospital and when I told her that I was going to be the secretary of the support group, she offered to come along and talk to our group and tell people about their feet. Well, I was always told with the diabetes, both extremities; eyes, feet.

**Q: So, does everyone else have a diabetes support group?**

Jay: If you ring Australian Diabetes Council and find out where your local one is, they are very interesting, very interesting, because they get different guest speakers along and it keeps you up to date with what's happening. I mean, some of the people that are in my group at Hospital 10we're all oldies, they're a lot older than me, but they have been going there for years.

**Q: It sounds like you find it valuable?**

Jay: Oh I do, I do and that's why on the first Thursday of the month, nothing gets in the way of it.

**Q: So, if you were designing a perfect health service, would you include a support group in it?**

Jay: Oh yes, yes, most assuredly so because I find sometimes you only pick up little bits and pieces there, but it all helps and you're all there, as our convener put it, the beginning of this month, "We're a family there." We've got our own diabetes family there and we're there to look after each other. We're not just there to go once a month. If we've got any problems, we should be able to sort of talk to each other or to talk to our leader. So, he's a good guy and he seems to be running it for so long, it's not funny.

**Q: So, does everyone else find that they've received adequate education?**

Mary: Well only from my GP and probably not.

**Q: So, Mary says probably not.**

Betty: No.

**Q: Warren or George?**

George: I would say that if I were director, I would-- it's all about doing something meaningful for diabetes and renal working together, hopefully a little bit more.

**Q: So, you wouldn't want them together, you'd want them separate?**

George: No I want them together.

**Q: You want them together?**

George: Yeah and then have a unit for education, to educate what else is going on because at the moment, I don’t think people have enough knowledge about what is diabetes and what renal function is *1:22:18.2. <inaudible>

**Q: So, is this if you've got it or is this for everyone in general, the education unit? Is it for you specifically, for people with diabetes, chronic kidney disease or the whole population; everyone?**

George: Well it can be the whole population, but only ones who've got it or are diagnosed with diabetes [and] *1:22:44.5 <inaudible> and to tell them what diabetes is and what will happen with it.

**Q: So, do you think you get enough information or no?**

George: Not really.

**Q: Not, really okay. What does everyone else think?**

Warren: I think <inaudible> *1:22:59.7 and probably more doctors and they can cut down their pressure and they look after a patient easier really.

Betty: To me it's just to have a problem somewhere, to tell us about what that mean the really sickness like diabetes. It's really dangerous, they can do damage to the kidney, they can damage the eyes; they can damage somewhere, in their body, to let the people know what that means. Some people, they don't know.

**Q: So, Betty are you talking about people with diabetes or the whole population?**

Betty: I'm says the whole population, they have one problem, [on the TV] *1:23:52.7 really. Diabetes is made that damages. The high blood pressure is [meant stroke] *1:23:59.5 or anything like that or their diet or something, there's problems to teach the people, because where they go the people who work, the educator, what times? To me they go to the local doctor and they tell some, but they not tell exactly. The doctors try quickly to tell, you're right, but to find exactly that the diabetes do damage to this, this and that. So, the doctors they know every sickness, but the people, they don't like that, what kind of damage they can do to their body.

**Q: Sorry guys, I've got to take a bit of a step back, so just in terms of you getting enough education, I think Jay said that he did, George said he didn't. Do you guys think that you've had enough education about your diabetes?**

Mary: No.

**Q: So, Mary doesn't think so, Warren?**

Warren: Well since I have been about 20 years a diabetic, I have learned over the years, how properly to take myself.

Betty: Yeah because I have 20 years…

**Q: So, Betty do you think you've had enough education?**

Betty: …and before 20 year, because I've never had a high tablet quantity *1:25:06.8 and now I take tablets and take medicines and I'm not used to it, who say, "Oh this diabetes" and they do their dance like that and people they don't know exactly. And I say, "What is this?" I say to the doctor, "I never hear." I never hear all the diabetes there, but also said to me, "Too much sugar inside the blood" and they do this dance to you; to the eyes, to the feet, to the kidney, to the stomach, everywhere. To me only, I found out the stomach they had too much air for my stomach, it's there all the time, like a burp, air or something and I've been to the doctor and I say, "What is it there?" And he says, "It’s come from appendix or something?" He's do the appendix to me and after nine months, he says…

**Q: Appendix is it?**

Mary: He said, "It's come from appendix."

**Q: Appendix, okay yeah.**

Mary: Yeah, a specialist, I go to a specialist and he says, "You have to take it out." I take it out and after nine months, it's continue the same thing and I go to him again and he says, "It could be come from the blood pressure, you got to have x-rays. It could be it's come from the blood stone," whatever you call them. It's the…

**Q: Gall stones.**

Mary: Gallstones.

Giuseppe: Gallbladder.

Mary: Gallbladder.

**Q: Gallbladder <inaudible> *&1:26:29.2.**

Mary: And I go to have x-ray of that and he says, "We must take the gall bladder out, because you don't have stones or anything like this, but the gallbladder is leaking into the stomach." Leak into the stomach is the gallbladder and I say, "Now what?" Because I have that exactly the same and I don't know what's that or the specialist and he's the specialist and I go and get the x-rays to my doctor. I do operation first, after nine months and I go to show them to my doctor, the x-rays, because the specialist will do the operation, he is sending me for x-rays. They say, "Go to the local doctor," who says, "What's this?" And when I go to him, he says, "You don't have appendix. You not have x-ray; you not have a gallbladder, why's he take it out of you?" "What?" I say to him. I go back to there, because he is operator doctor. He's not like an ordinary doctor and he says, "Oh probably because the way you are breathing." What I tell to him, nothing, so that's why the people, they don't know how about what is the sickness.

Giuseppe: Excuse me, I give you advice, I have the same trouble.

Mary: Yeah.

Giuseppe: Every night, one O'clock started pain and wanted to bring the air up.

Mary: Yes, yes the air up.

Giuseppe: Uh-uh all the time and I went, a couple of doctors, they couldn't find what it was. Even they stopped me to have a soft drink, the beer everything, still there. 2:00 O'clock I had to be in the markets and it bloody come out at 1:00 O'clock, 1:30 and then I feel a little weak. Anyway I went to…

Jay: IBS?

**Q: Maybe or [acid reflux] *1:28:23.2**

Giuseppe: My doctor, the GP, he send me to Professor Newey here in the medical centre.

**Q: Professor? Sorry Giuseppe, Professor?**

Giuseppe: Newey, Newey.

**Q: Newey, yeah.**

Giuseppe: You know him?

**Q: No.**

Giuseppe: It's in the medical centre. He checked me, he put me tube in and out <whistles>.

**Q: Ulcer is it or reflux?**

Giuseppe: Yeah, he give me tablets. They're called Losec.

Mary: Losec?

Giuseppe: Losec. He said, "To start with take one in the morning, one at the night and then after, then you should take one." Since then, no more burp problems, but I still take the tablets.

**Q: So, probably…**

Mary: But you still take it, you still take the tablets?

Giuseppe: Yes, but no problem anymore. It was acid in the stomach.

Mary: Acid in the stomach.

Giuseppe: That's what it is.

**Q: So, sorry I know we've gone off topic a bit, let's come back again, sorry guys. So, the question was, if you became director of a hospital and you had the power to change and improve things and you were designing a new diabetes and chronic kidney disease health service, what would you include in it? What would it look like? I think we've mentioned more resources. We've mentioned perhaps a combined clinic. Actually a lot of you guys agreed on that I think. We might have some better education. I think it was more, better education, was it both for patients and the whole population, is that correct?**

Betty: For the patients, for the patients I say.

Jay: Definitely for the patients.

Warren: Yeah.

**Q: And what else would other people include?**

Warren: One shop for all, you know. Do one shop where all the doctors and the patients meet together.

**Q: Oh and I think we said we'd include a support group there as well.**

Jay: Yeah, yeah.

**Q: And you're saying you can see your diabetes doctor and your renal doctor at the same time?**

Warren: Yeah, kidney doctor and the…

**Q: Anything else there Warren?**

Warren: Dietician and physiotherapy.

**Q: How would that be helpful?**

Warren: Then they kind of do get the patient more efficiently.

**Q: Anything else, anyone else wants to add?**

Mary: I go to the dietician and they give me what I eat. Very light, that's the rule for diabetes.

**Q: So, other people also mentioned better parking as well.**

Oh yeah, that is very important to me.

**Q: Well, I'll just save that one, instead of people laughing at it. Anything else people want to include?**

Jay: Yeah Jay here. Better parking would be good, especially round here, but as I've said, they can't build a multi-storey car park, which would be a godsend around here, because of the locals. And the parking across the road can be a bit expensive, especially when you're on a pension and you’re watching your pennies, which I have to do, because I've just had to find a fortune to get some work done on my car and other things have had to sort of take a backseat. So, that's one of the reasons why I come over here by public transport on numerous occasions, because I get the bus from home to Granville, the train from Granville to Strathfield and then the bus down here.

**Q: Sorry guys, another question. Some people have mentioned they've found that sometimes the communication between the specialist and the GP's not great, so when they go to the GP, the GP doesn't know what the specialist's said and stuff like that or sometimes when they go to the specialist, the specialist hasn't got their results and they have to ring around and they have to ring around or stuff like that. Have any of you guys found that as an issue.**

Giuseppe: Well I mention my doctor all the time. I said, when I go to the specialist, I said, "Will you fax this to Dr Paris?" "Oh, yeah, yeah, yeah, yeah." When I got to Dr Paris, has she got the report? Because, has she had the fax? But, I remind the specialist to send it, because it happens a few times. He had the blood test and on the other side they did not have the reuslts *1:32:44.4 <inaudible>, but if you mention, remind them <laughs>, well you're alright

George: When you come to think of it, George here, blood tests have been sent in one week before and the doctor, first Dr T or sometimes Dr W too, so that's a big problem.

**Q: Sorry George, so your renal and you diabetes doctor hasn't got the results is it? <he nods> Okay.**

Mary: When I go they just look them up on the computer.

**Q: So, Mary's a bit-- Oh so you do the blood tests in Hospital 2 do you?**

Mary: I do the blood tests in Hospital 2 and then I go to the doctor and they just look them up on the computer, but when I do go, I always say, when it says to send to somebody else, I usually write my GP's information there, so hopefully they'll send the copy of the blood tests to the GP as well.

**Q: Ok now, has anyone else got anything else they've come wanting to say, that they haven't said? About their experiences with diabetes and chronic kidney disease, about their own personal experiences on caring for it all or important things or their own experiences with the health services, so did anyone come in wanting to say something that they haven't had a chance to say?**

Giuseppe: Well listen, although I've actually got to talk about the expert, because I talk a few times with the expert. What the lady said before, you can lose your eyesight, you can lose your tooth, you can lose even your toenail.

**Q: That's correct.**

Giuseppe: Or fingers.

Betty: They tell you, yes.

Giuseppe: Because a friend of mine, he lost-- I saw him here in the hospital. I went in the hospital to take off polyps and I knew him for longer. He said, "I come in here, so that they've got to take my toes off." Because of the diabetes, what I know and what I explained to him, it's a different diabetes.

Mary: Yeah, that's why I said it, it's a <inaudible> *1:34:57.5

Giuseppe: We are here. We aren't all the same. We aren't all the same diabetics, we have and they haven't and when they don't know they have it, that more danger than us.

**Q: Sorry, say again?**

Mary: Because they don't.

Giuseppe: Yeah.

Betty: They don't know they have it.

**Q: So the one's that?**

Giuseppe: The ones that they have a different diabetes, they are different to me.

**Q: Different dia..?**

Warren: Diabetes.

**Q: Diabetes, oh yeah.**

Giuseppe: Different, but the same. There was a bloke work in the markets, every day he get the big bread roll, salami, eggplant fried and forgot they got too much oil and he shout out, "I want bottle of beer." I said, "Johnny," I said, "Take it easy." <laughter> I used to tell him, many times you know. "Oh one cigarette a day. I'll die on the full stomach." He went with this brother for a holiday in the Gold Coast and he come back in a coffin. He die over there, because he eat the rich food, he's not supposed to eat it.<laughter> Well I'm not into that. I like eggplant, I like [capsaicin] *1:36:05.1, I like that, but I might have one or two slice, not bloody that big bread roll full and beer. Once the doctor told me, no drink beer or soft drink, I never touch it, I don't touch.

Betty: That's right, they tell us, because we don't know.

Giuseppe: Yeah, doctor tells us from the…

Betty: We need to stop for the diabetes, yes.

Giuseppe: …heart specialist, he said to me, "You can have a small glass of wine, only ever with your meals," but he said, "A red one."

**Q: So, what you're saying is it's important to see the specialist and listen to what they're saying, is that what you're saying?**

Giuseppe: Yes, of course. What's the good I go to the specialist, if I don't listen <laughs>?

Jay: Jay here. Plus, I think you've got to ask them questions as well, if you've got anything.

Giuseppe: About the parking, if I was to suggest, because at the hospital, they collect a lot of money for parking, they could build double-storey, plenty more cars can park, it clear the street.

Jay: The residents around here don't want it.

**Q: Don't they?**

Giuseppe: The residents here, they alright, because they mark you can't park in front it's a driveway, they all mark <laughs>.

Jay: But, the residents here did not want a multi-storey car park across the road. They tried it a few years ago and the council had a meeting, it was knocked on the head.

**Q: Sorry Jay, I think you were mentioning something before. You were saying, it's also important to ask the specialist questions, is that what you were saying?**

Jay, Yeah, yeah if you're thinking of different things, you must ask them, so that you're clear in your mind as to what your situation is and...

**Q: What happens if you don't ask them?**

Giuseppe: You don't know <laughs>, if you don't ask them.

Jay: Well you don't know. You're like the mushroom then.

**Q: What do you mean by that?**

Jay: They keep you in the dark and feed you full of bullshit, pardon the French <laughter>, but I mean, if you don't ask, you don't know. So, if you've got queries, like I always ask my GP, "What about this here? What about that?" And he'll tell me and years ago I went to a doctor who had a whiteboard in his office and if you had something wrong with you, he would draw a diagram with a marker pen on the board, to show you what was wrong and he'd explain it to you. He's the only doctor that I've ever had over the years that has done something like that.

**Q: Does everyone else agree that you should ask questions?**

Giuseppe: One in a million <laughs>

Warren: Yeah.

**Q: So, I think Mary and Warren, would you guys think that you get less, if you didn't ask questions, that your healthcare wouldn’t be so good?**

Jay: Jay here. No, I don't think less than satisfactory, but I mean it's in your own interests all the time to ask questions and it's not as if you're putting the doctor on the spot or anything, it's just that you need to ask what you need to know to assist you in managing your problem.

**Q: The only reason why I brought that up, was an interesting theme from other places, was especially in places where doctors were busy, if you didn't ask questions, you never got any information.**

Betty: The doctor sometimes is busy, yeah.

Jay: Yeah, I agree with that.

Warren: Yeah.

Giuseppe: Yeah it depends on the doctor. Some, they are very polite to you, they try to explain. Some of the other ones aren't because they are too busy.

Betty: So quickly, yeah.

Giuseppe: And they want to kick you out as quickly as possible.

George: I agree *1:40:01.5.

**Q: Sorry George?**

George: I asked the doctor, Dr W, renal doctor. Something <inaudible> *1:40:07.4 <someone coughing> ask him <inaudible> was controversial, he always pack it in <inaudible> *1:40:13.2 panicking and blow it. He will not tell. He will not say anything, he would not say anything. He [wait] *1:40:22.8 what to you do <inaudible> *1:40:23.5 [questions] <inaudible> something like that, you know.

**Q: Well, we're almost reaching time guys. Has anyone else got anything else they wanted to mention? Any burning things they wanted to mention before we close up?**

Mary: No.

Jay: I think we've said enough.

**Q: Well, if that's the case, we've just got one quick survey for you guys to fill out. It will take less than a minute and then we're all done.**

**I'll just hand these out to you.**

END OF TRANSCRIPT

**Focus Group 3**

**Q: As a quick starter, the first question I’ve got for all of you guys is based on your experiences, what would you say are some of the weaknesses of the diabetes and chronic kidney disease services here at the Hospital 3? So what are some weaknesses or areas that could be improved?**

James: I have nothing to fault the service that I have received over the last 12-13 years is A1, they’ve been right on the ball, great support.

Dana: I’ll back James up, I think the service at this hospital and the care given to patients at this hospital is unbelievably fantastic.

Valerie: I don’t have any trouble with the Hospital 3, they’re really great.

**Q: That was Valerie, that’s it.**

Michael: I’d echo the comments that sounds like a bit of me too-ism, but I’ve been a patient of one of the doctors here for about 15 years and he’s been fantastic. When I had a bowel with cancer about seven years ago and the chemotherapy played merry hell with my medication for diabetes and he was on the phone and available to me 24/7 for the entire treatment period and I couldn’t fault that.

**Q: Sorry Michael, just to interrupt for a second, who looks after your diabetes?**

Michael: P X.

**Q: And do you see him privately or publically?**

Michael: Publically, I see him about every three months.

**Q: And what about for your kidneys?**

Michael: Kidneys I see Dr T.

**Q: And do you see him in the private?**

Michael: I see him privately, but only every 12 months.

**Q: Does he bulk bill you?**

Michael: Yes.

**Q: Sorry, going back to the initial questioning, Zeb did you have anything that you wanted to add or agree or disagree with everyone else in terms of weaknesses or things that could be improved?**

Zeb: Well I, like the rest of the group, I can’t pick on any weaknesses besides this leave that open that I may find something in the future, but I haven’t used a diabetes a lot. I was up in the country living and you sort it out yourself mostly living in the country. When I came to R City, Michael, I had a multiple myeloma and, of course, with the chemo that the diabetes went completely haywire and Lyndal Tacon from this hospital here was just on call whenever I needed her, she was just brilliant and so I had no problems there whatsoever. As far as the kidneys are concerned, I was under Professor Richard Apple for 30 years.

**Q: You saw him publically?**

Zeb: No, sorry, private and I wasn’t a good patient. I was supposed to see him every six months and I did a phone call saying if you don’t come and see me, then I’ll have to visit you in the dialysis rooms, that was his way of getting me to come a bit more regular and, as I said, he looked after me for 30 years, then he died and I went on to and met [Muga Tuong] and he’s been brilliant. Can’t fault him whatsoever.

**Q: That’s what it sounds like that you guys have only got good things to say, so that’s excellent. Is it easy for you guys to get the diabetes and chronic kidney disease care that you need, so in terms of locality, cost and affordability and waiting times, is it easy for you get the diabetes and chronic kidney care that you guys need?**

James: I say yes, very easy and the pharmacies around our way, we go to two different pharmacies; one for convenience, one for continuing and both of them are superb, they’ve always got resources we need and so on.

**Q: And in terms of accessing the diabetes care that you need from the nurses or specialists and the kidneys, that’s easy as well?**

James: It is easy, yes.

**Q: Locality’s okay, cost is okay, waiting times all okay?**

James: Well we live about 20km, I live at West Pennant Hills, so we commute here because we originally lived in this area and so we stayed with this hospital, but still, there’s no difference, the service is still good and the pharmacy, of course, is supportive too and you need pharmacies.

**Q: What does everyone else think?**

Michael: The only comment I would make is I can’t fault the services and the availability. Like everyone else, I guess, the cost is an ongoing thing because the drugs required are not cheap and if there was any way to diminish that cost factor, I think everyone would benefit from that.

James: I would agree with that, I think it’s about a $600.00 limit. Now I’ve got a chronically ill wife as well, so for the first few months of the year, we’ve got to pay that $600.00 in the first two or three months of the year, because that’s how heavy our burden is and that’s the gap and that hurts at the early part of the year.

Michael: Particularly as you get older.

James: That’s right.

Dana: I live way out in the west so the problem I have actually is unless the ambulances bring me, I don’t have that much access to the hospital and the health service out the west for diabetes and chronic disease are not that good.

James: That raises a point, my wife said to make a point of this, we’ve had a few instances where an ambulance has been called and they’ve wanted to take us to Hospital 3a. Now where we live, Hospital 3a might be the closet if you draw a circle with a compass to get a distance, but in time, I live right next to the M2, and the M2 is only ten minutes by ambulance from my place to here, but Hospital 3a is about 25 minutes and they’ve raised the point as to our priority, we’ve been focusing on here for over 40 years and if possible, we’d like ambulances to note that this is where we’d like to come. Time is the essence-- I’ve got heart and, of course, with a hypo and that sort of thing, these are dangerous situations and distance is not the equation, it’s the time. As I said, I live right next to the M2 and we’re here in no time, so that is one little weakness, so we’ve had to argue with the ambulances. They just oh no, this is the instruction, nearest hospital, well nearest in distance and nearest in time are two separate things and also the quality at Hospital 3a is very different. <laughter>

Michael: Exactly, if you’ve ever been to Hospital 3a for anything <laugher>, you’d much prefer to come here. <laughter>

James: Yes definitely

Zeb: It’s got a reputation.

James: Yes sadly

Dana: If you complain about Hospital 3a guys, try Hospital 7. I told them that I have chronic kidney problems, I know I was sick, I know I needed an antibiotic, but I know also that you don’t give vancomycin to someone to someone with chronic kidney and if you do, you have to give them a low dose and spread it out over two or three days. They gave me 1,500g of vancomycin twice a day, so my creatinine went up from 119 to 204. They don’t even listen to you, so it’s really bad out there, as I said, I’m actually going to apply to move closer to here because I’m in fear of my life if I live out there much longer.

**Q: Because of the medical care there?**

Michael: Or lack thereof.

Dana: You can’t see a specialist out there and you get a new doctor every three weeks and I’m sensible enough, I do have some medical knowledge.

**Q: It sounds like it, when you just said that, definitely that that’s right.**

James: The measure of time just listening to here is that this hospital is a beacon

Zeb: Yes

Dana: Absolutely

James: we’re getting probably the best service you could get anywhere, but it’s a beacon for a whole system and if it could be the environment that we have could spread to other places, it’s certainly wise. People shouldn’t be having these sort of problems.

Zeb: I’ve got a little bit of a different story, my closest hospital because I moved from Mona Vale to Campsie because my carer, my niece, I have Canterbury Hospital to compare with and I was reluctant to go there, I used to talk the ambulance guys into taking back to Hospital 3 and they always did, never have anyone refuse me really, but then eventually one day it was too far, I had to go to Canterbury and I was just blown away, you know, certain departments in the Canterbury Hospital how brilliant they were. In fact their heart section, this is in the last 12 months, I thought was better than here and so my story is a little bit opposite, so it’s nice to tell.

Michael : Don’t go to Hospital 3a.

James: Yelp

Zeb: Don’t go to?

Michael : Hospital 3a.

Zeb: No, you’re not the first person that’s said that.

James: Well I was living in the country for a while and I was living in an obscure place called Taylor’s Flat, which is between Cowra and Crookwell and we had a choice of about four or five hospitals, we had Cowra, we had Boorowa, we had Young and we had Crookwell and also there was Yass if you wanted to go a bit further, but the services in that area, believe it or not, we were about 50km from the nearest centre, we had great backup, medical services were excellent. I didn’t feel uncomfortable at all.

Zeb: Very similarly, I lived in Hunter Valley up until I moved down to R City in a little place called Brooke.

James: I know Brooke, headlines at the moment.

Zeb: Yes, it is too.

**Q: Can I say for the purpose of the recording, it’s Zeb and it’s James speaking.**

James: Sorry.

**Q: Keep on going, just for the purposes of the recording. Keep on going Zeb.**

Zeb: But in the Hunter I had Singleton as a local and Maitland as a slightly larger area and then Newcastle, John Hunter and they were just excellent. As per normal in the country, you have to allow each time that you could get to these places, someone to drive you and when I was told that I had cancer I thought this is the end, this is the pits because someone’s going to pick you up after having, this is what I thought about, having chemotherapy, that someone picked you up and you said hello and then threw up in their car and they took home and washed their car out. I found out it’s James and it’s not like that anymore, but other than the time situation, the services were brilliant.

**Q: I think we’ve definitely heard from the right side of the room or my right side of the room. How in terms of accessibility, so easy it is to get the diabetes care at the Hospital 3, what do you guys think on my left, in terms of ease of getting diabetes and chronic kidney disease health care that you need in terms of locality, cost and waiting times, how easy is it for you to get it?**

Valerie: I live on my own and I do everything myself, except I have a cleaner come every two weeks and I have the council bus come in every two weeks takes me shopping, picks me up and bring me home.

**Q: That’s pretty handy Valerie. What about in terms of getting the diabetes care that you need and the kidney care, how easy is it?**

Valerie: I only live at Chatswood and I only got to cross over the road to get the Manly bus to come here.

**Q: That’s handy, what about waiting times, waiting times all okay?**

Valerie: No, never thought.

**Q: And cost?**

Valerie: No doesn’t cost much at all.

**Q: What do you guys think Zeb and Dana?**

Dana: I mean once I get to this hospital the care is absolutely unbelievable. Yeah, sometimes you have to wait for the doctor to run late, who cares, as long as you know that that doctor is going to see you.

**Q: Is this in a clinic?**

Dana: No, I see Bruce privately at Northern Specialist and I see T there as well and I used to see P X there as well. I suppose you come to clinic one, in the old hospital, for about 25 years, I saw P X there. So as far as accessibility, I get my kidney injections from the outpatients pharmacy downstairs, so I get a lot of my services from this hospital and I’m eternally grateful for that.

Zeb: I haven’t got much else to report other than to praise everyone at North Shore.

**Q: So it’s accessible for you?**

Zeb: Yeah, with major assessment because thing was we either stay in this area as I moved to Campsie, which is western suburbs, or move into the western suburbs thing and I’m so comfortable here because this is where I started 40 years ago that I’d rather drive or be driven from western suburbs to the North Shore to know what I’ve got at the destination, which is everything perfect.

**Q: Different question guys, sort of related, what are some of things, if anything, that you find frustrating about the services that you get for diabetes and chronic kidney disease here?**

Michael: I’ll go first. I think coming here to see P X for about 15 years I honestly cannot say in 15 years that I’ve been frustrated by him at any time. He’s an extraordinarily courteous man. If ever he can’t make an appointment, you get in plenty time, advance warning that he has to change the appointment and it’s always asked in such a way that what is convenient for you, can we and you get alternatives. I see him mostly about 7 o’clock in the morning and that doesn’t worry me because I live very close by to here and getting here at 7 o’clock is fine.

**Q: Early starts.**

Michael: We’re all of an age, who is awake after six, who’s asleep after six, we’re all awake at six.

Zeb: I am.

Dana: I am.

Michael: You’re lucky. I’m always awake at six, getting here by seven is a doddle and I don’t mind it. I find it very convenient, very courteous, very punctual, I’ve never been kept waiting more than quarter of an hour and I’ve got no complaints whatsoever.

**Q: And in the kidneys?**

Michael: My kidney complaint is very minor and it’s really precautionary more than anything and I see Dr T once a year. I’m usually kept waiting about five minutes and I’m in to see him for no more than five minutes and then I tell him I’ll see him next year and that’s it.

James: Well I’m with Dr Gad Frank, I can’t say anything negative whatsoever. The service is prompt as an appointment, but hang on I’m amazed how accurate he is with his timing, that sort of thing and he’s patient with me. He has my blood test and he goes through each one of those, if there’s anything where he sees I’ve been naughty and he picks me up pretty quickly. He’s not rude about it, but he highlights it and he tries to correct it, so I couldn’t wish for better. There’s just no room for improvement.

**Q: And the kidneys?**

James: He watches the kidneys, but he does know that I’m with Dr Killshed and they do exchange reports, so he watches, I’m currently about 220 creatinine and that’s being watched. It’s actually fluctuating, of course, I thought it just went up and up, but …

**Q: Anything frustrating with Dr Killshed or?**

James: Oh no, she’s brilliant and she’s great fun, she’s got a sense of humour and she’s a very pleasant lady to deal with and she talks right at you, same with Dr Frank, they just talk straight at you rather than beat about the bush or even try to become academic with you.

Michael: Can I just make an interjectory comment here, the thing that I think I’m continually amazed is the way the records at this hospital are kept and the IT system is such that any doctor at any time, for any reason, can access your records and know exactly what is wrong with you, what your blood type is, what your blood sugar levels are, what your creatinine level is, everything. It doesn’t matter who you go to see, they can bring it up and it saves just so much time and it’s so efficient. If you could have that Australia wide into any hospital in Australia at any time, that’s about the only reason

James: Oh Yeah

Michael: I think, the NBN is worth having.

James: Oh Yeah

Michael: Sorry for the political interjection.

**Q: Provided you vote labour <laughter>**

James: They are working on it, as a matter of fact, the last time I came out here there was some people from a health organisation trying to promote a common records system, I forget what they call it now, but I’m seriously …

Zeb: E-health.

James: Yeah, that’s it, yeah, well that sounds a wonderful idea that you can go to one doctor and they’ve got the records, it’s wonderful (Michael agrees), yes, I feel it should be compulsory, it should be just done.

**Q: From this side of the room, any frustrating?**

Valerie: No, my doctor, Mayhead, he rings me up and make another date very good, I’m very pleased. I wouldn’t go any other hospital, I’ve been coming here for years.

**Q: So it sounds like your happy Valerie, what about you Zeb or Dana, any frustrating?**

Dana: The only frustrating thing I have or negative thing I have from this hospital is that I have to arrange with T X for me to come to emergency and I get sort of a negative feeling like what are you doing here, you’re from the west and I feel that is the only negative part, but once I get in there, the treatment’s great, but it’s just, oh well, you know, you should go to Mount Druitt or Hospital 7 or one of them and I don’t feel comfortable there. My doctors are here, I’ve got six specialists in the place, they’re all here, they’re not over there, they’re not out there, they’re here and I obviously can’t get an ambulance to take me from Mount Druitt to here, so I was sick about three weeks ago and I had to hoof it by train to get here, but lucky T was on duty and I rang him up and said T I need your help, can I meet you emergency and he asked me and we talked about it and he said yeah, you’d better come down, I’ll have a look at you. He ended up admitting me for four days, so obviously I was sick and I had to hoof it by train because I couldn’t get an ambulance to bring me here and I knew if they did emergency would turn me away and I’ve actually been turned away twice by emergency even though I’ve been coming to this hospital for 30 years or more.

**Q: Is that cause they say that you live out west?**

Dana: Yeah, I think that’s where it comes from.

James: I didn’t think they could do that.

Dana: Well they can’t, but they do because …

Michael: They put it to you on the basis that you can stay here if you like but the waiting time is 37 hours.

Dana: That wouldn’t worry me.

MIchael : I’ve had that experience, yes.

Dana: That would not worry me, as long as I was being watched and all the rest of it and I actually, when three weeks ago when I went to emergency, I actually had to stay there one night and three quarters of the next day to get a bed in 8C, which is the endocrine ward, but I got to 8C and that was important to me too.

Zeb: I never felt any of what you’re talking about when I’ve arrived here. I always say to them, look I haven’t gone locally to western suburbs because all my specialists are here and they say, yeah, we understand that and I’ve never had a problem, not once.

Michael: I haven’t had it either

Dana: I’ve had it twice now.

Zeb: No, never.

Dana: To the point where I had to write to the health minister.

Zeb: I’ve had the other where they say downstairs why don’t you come directly here.

**Q: Have you guys found it an issue with communication between the specialists?**

Michael: No.

James: No.

Zeb : No.

Michael: Because of the centralised IT system communication between the specialists in the hospital, even external specialists that I’ve had to go for alternative reasons, I had to go to a gastroenterologist recently, mind you, only a block away from here, but he had my complete history in front of him and it just never ceases to amaze me. My GP is near my home, which is in Northbridge, which is not that far away, a couple of suburbs away, a few southerners who don’t understand it, and he has my records on IT instantly.

**Q: So everyone say there’s been no issues and you’re saying also there’s no issue between specialist and GPs, is that everyone else’s experience as well?**

Dana: Yeah, Roy X writes to my GP every time he sees me and he also cc’s the letter to me as well, which I find really good. My GP’s pretty proactive and my GP actually has a special interest in diabetes, works out well.

Valerie: My GP is very good, he lives at Willoughby and I’ve got an appointment to see him tomorrow, I’ve got have a blood test done tomorrow.

**Q: That’s good Valerie, so the communication is good between them?**

Valerie: He’s very good, Dr Koo.

Zeb: Mine’s not quite as good as they have, I do turn up at my GP and I’ll say have you had such and such and she’ll say no, so I guess she’s not as good as yours, but that’s when I have to take into consideration that’s western suburbs compared to northern suburbs.

Michael : But that shouldn’t be the case.

Zeb: It shouldn’t be the case.

Michael: I mean automatically every time, like the other people, every time I go to my GP he brings up letters from my various specialists and vice versa and even the gastro guy I went to the other week, he had letters there in front of him from the various specialists and my GP.

Zeb: I haven’t found exactly that, it’s like 50/50, it may be there, a letter, because I’ll say okay I’ve seen such and such a specialist and it may not, it’s happened …

**Q: So it sounds like there seems to be a bit of a disparity not only in the hospitals, but the primary health care between the northern suburbs and the western suburbs.**

Dana: Big, huge.

Michael: They don’t like us out west. <laughter>

Dana: I wouldn’t say that, I’ve been living there for years and I love yous.

Michael: Joke darling, it’s usually the other way around. <laughter>

Zeb: It does seem to be showing up.

Dana: Just basically what I think it is, is that they wonder why like what I feel the three times I’ve been to Hospital 7, they wonder why people like me because we live out there come here in the first place and I think there’s a big division out there. We know as much as they do is the feeling I get and yet by experience they don’t. When I gave you that previous experience about my kidney and the vancomycin, they obviously don’t. Poor old Dr T had to fix me up when I came out of that hospital because my creatinine shot up from 119 to 204 and I worked hard at my kidney and I work at my diabetes and to have a hospital muck it up for me didn’t make me too happy.

**Q: So for a non-R Citysider you’re going to have to educate me a bit. You spoke a bit to sound like there’s some rivalry between the northern and the western suburbs, I don’t know whether I’m picking it up correctly, but is that what you were saying there? What’s this thing between the northern and the western suburb or what’s the division, sorry, you’ll have to educate me, I’m from T City, I’m not familiar with.**

Dana: My understand is that they think well if you live in our area you should go to us, but then I don’t want to go with you guys because you have no idea what to do. I don’t think the doctors are as good as the ones we have over here. My GP’s in Leichardt, so I don’t even go to a GP out there because …

Q: Sorry, Leichardt is?

Dana: Leichardt is like the inner west of R City, sort of like …

James: Carlton.

Dana: Camperdown, Stanmore, Five Dock, Haberfield, Leichardt.

James: It’s Carlton, Fitzroy, that sort of situation

Zeb: inner west.

James: I know a little bit about inner T City. <laughter>

Dana: I’ve been going to Leichardt for 20 years.

Michael: Just one comment, I come from T City, I’ve been here 30 odd years, when I left T City I’m sure you had the same feeling about the Royal T City Hospital and the inner city hospitals. If you lived in Frankston or Sunshine, that’s east and west of T City, if you had a choice, you wouldn’t go to Frankston Hospital or you wouldn’t go to a hospital out in the west, you’d always go into the centre. In the 30 years, maybe things have changed, I’ve noticed Frankston Hospital is now quite a large seemingly well positioned hospital with all the service, at least my sister who lives down tell me this and things may have changed in Victoria I think really what everyone’s saying is the services out west are not as great, perceived by most people to be not as great as this hospital here.

Dana: Absolutely.

James: I presume that T City has a cultural divide just like R City, R City certainly has, you’ve got the east side and where the line is, is hard to discern, but there is a mental difference, you’ve even got different sports, people from ethnic groups even concentrate in different areas, say you’ve got a lot of Chinese in the northern side, where we are there’s a lot of Indians and the further you go out you’ve got Arabs and this sort of, so there’s a different cultural divide.

**Q: So where are the cultural divides in R City?**

James: It’s sort of east and west.

**Q: Where’s that line drawn?**

James: It’s hard to define, but more or less …

Michael: North Shore and eastern suburbs are really the east. The west is sort of like …

**Q: What about Hospital 2?**

Michael : That’s west.

Dana: No, it’s inner west.

Michael: Inner west

James: But Hospital 2 would be the start of the west.

Dana: Parramatta Down basically, Penrith <overtalk>.

James: yeah it is

James: Bridgetown, Auburn, all that area’s west, but they’re even attracting different cultures. A lot of the migrants coming into the country are going out that way. You don’t find too many Arabs say on the eastern side.

**Q: What about Westmead and stuff like that?**

Dana: Yeah, that’s west.

James: Yes, you’ll find them there and Somalians, you go to Parramatta …

**Q: So what’s the perception of hospitals like Westmead or Hospital 2 or stuff like that?**

James: Well I have a tendency to think that the people out west, and you could correct me, tend to be a little bit complacent compared with the people over here and also there’s a community spirit. People are very sensitive who are part of the North Shore Hospital community, they’re very sensitive to the standards and, of course, the hospital staff and board are responsive to this. There’s also a community effort, a lot of facilities here in the hospital have been given by people within the community. I notice that Dr X’s name is actually on the roll, or used to be on the roll of honour that they had as one of the major donors to this hospital to some of their functions and so on, but you go out to the western suburbs, I don’t even think they’ve got a roll of honour for people who’ve made major contributions to the development of the hospital.

Dana: I think Westmead does.

James: That’s a children’s hospital.

Zeb: International is not the word, Hospital 2 being, sorry Roy speaking.

**Q: Zeb.**

Zeb: Zeb, thanks cheeky.<laughter>

Dana: That’s why I didn’t change my name. <laughter>

Zeb: I’ll never do it again, but I have had a reason to use Hospital 2 and as that was the Repatriation Hospital, I think it sort of swings all of R City respects Hospital 2 perhaps

James: Yes

Zeb: and now it’s swung over and I was there recently and they needed a test for my kidneys and they said, oh you’ve had it done and I said yes, well they said we’re the only one that does it. So they sent me from here to go to Hospital 2 to have this test because they’re the only one fbecause it’s a Repatriation that gets them

James: yes

Zeb: millions required for this purchase of one machine, so it was servicing the whole of R City.

James: Hospital 2 is actually owned by the Australian government. Heidelberg in T City’s the same, they’re both owned, yeah, they’re Australian government owned, but they’ve opened it up …

Michael : They’re both repat hospitals.

James: Yes.

Zeb: They’re both repat, yeah.

James: Yeah, repat hospital, yes.

Michael: My recent experience at Hospital 2 was nothing short of disastrous

Zeb: Yeah mine was brilliant

Michael: and I wouldn’t send anyone to Hospital 2 if I could avoid it.

Zeb: I went to a man that’s regarded as brilliant in the kidney, I can’t think of his name just now, but I thought and a couple of specialists said to me you might want a second opinion, so I went to this guy at Hospital 2 having gotten a second opinion and I have to say it was just absolutely brilliant and the end result was Mugat Wong at Hospital 3 is doing everything spot on, straight on fact and that’s the result I got from going to that guy, so there must be a few brilliant people out there at Hospital 2 still, that’s the way I’m looking at it.

**Q: I might just move on cause the interest in time, so what would you say are some of the strengths and I think we’ve mentioned some of it already, we’ve said the services here are really good, but what would you say are some of the strengths of the diabetes and chronic kidney disease services here at Hospital 3? So I guess you’ve all said that it’s good, but I guess this sort of question is why is it good, what are the strengths?**

Michael: I’ll go first. I’ve been coming here for 15 years. I think the accessibility of the specialists, the help you get at all levels in the hospital, the correct diagnosis and treatment and prescriptions that you get and the care that they show, I’ve not had any alternative experience, so I guess maybe my sample is a sample of one, so therefore we’re not getting much comparison, but like the others, I tend to think that even if I moved out west, I’d still come back in. Although I don’t know what’s going to happen when P X retires this year.

Dana: Yeah, he’s going up to Chambers as far as I know, hopefully he might transfer you to his son, his son is nearly as good as his dad.

Michael: Good, we’ll see, but I have no negatives at all because I’ve got nothing to compare it with.

James: I’ve been coming here for about over 40 years now and we raised one of our daughters who was a type I diabetes and the service we got for her was brilliant. She had about five years of hell, young girls going through puberty and with diabetes is absolutely hell and I think she spent about 50% of that time in hospital and we’d have her home and then the signs would be there, hypos and hypers and this sort of thing. We got her into this hospital, we only had to go to the triage person, half the time they knew who she was she was in here so often, but as soon as she arrived, the triage people just put her straight into the system and they had drips on her and this sort of thing. So our image starts with the way she was treated and, of course, as we got older, our health went down and my wife and I we’ve got different illnesses, but we’re treated very, very well. There’s just no room for even suggesting an improvement and you wouldn’t dare complain, there’s just nothing to complain about.

Dana: I think the reason it’s good is if I leave a message for Dr T X or for Dr T or for Dr H or one of my other specialists in the joint, they’ll call me back and I think that’s the important thing. Having the accessibility, I don’t abuse it, but if I need to get hold of one of them, they will call me back. It mightn’t be the same day, but I know that eventually T will ring me back and I think that’s, to me that’s the important bit and you would not get that anywhere in another hospital I don’t believe.

**Q: So you didn’t get it at Hospital 7 for instance?**

Dana: Oh God no, I couldn’t even see a specialist at Hospital 7.

**Q: Couldn’t?**

Dana: Couldn’t even get a specialist at Hospital 7. I died and got resuscitated and I went to Hospital 7 cause they didn’t know what I died from and I was there for eight days and I got frustrated cause they weren’t doing anything and they were giving me an antibiotic and I mean I died, I got resuscitated, I had no infection, so I didn’t know why they were giving me antibiotics, which sent my bowel burko and then I said to the doctor, can you give me a signing sheet, I said, I’ve had enough. Oh, you can’t do that, I said, oh yes I can and I said, well tell me what I died from, we don’t know, it’s all inconclusive, so I just looked at them and said give me the sign out sheet will you, I’ll sign myself out. Oh you know it’s against medical advice, blah, blah, blah and I said, yeah, just give me the sign out sheet, I’m going and I didn’t tell them I was going over here, but I just said I’m going and got a friend of mine to pick me up from there, came home for about 40 seconds, came over here, went to emergency, within three days they told me that I had a respiratory, that I died from a respiratory attack, from undiagnosed sleep apnoea and so to know that. It took this hospital three days, I was in there for eight, we don’t know and I think that’s the difference.

**Q: Valerie, these are strengths.**

Valerie: I’ve been going five years since I found out I was a diabetic and I’ve had no trouble, they’ve been marvellous and everything and it can’t come appointment, they’ll write to me or they’ll ring me up and make another appointment, so I’ve got no trouble with hospital here, they’re wonderful, wonderful here. Even the nurses when I was in the hospital were wonderful.

Zeb: It seems to me I don’t keep a handle on my medical things quite as tightly as some people do, I’m a little bit easy going with it and so when I’ve used the endocrinology here is because it’s an emergency basically and when everything’s running normal I tend to skip a few appointments. So it’s hard to say on a continuity basis, I’d be one person that couldn’t say, but if anything goes wrong, it picks up so quickly it’s as though I never missed anything and …

**Q: So that’s a strength, is it?**

Zeb: The strength?

**Q: Yes.**

Zeb: Yep, so the strength is they, although I’m not a goody goody, they pick it up and carry it and there’s no complaints from my end, but I admire what they do to put up with me.

**Q: This is a similar sort of question, what impact or difference has attending the diabetes and chronic kidney disease health services made to your health?**

Dana: The answer is I’m still alive, then I shouldn’t be, that’s my answer.

**Q: Does everyone else agree?**

James: I’d agree with that yeah. I haven’t come to a death knell with it, but it’s nice to know that that resource is there if you’re in danger, that resource to protect you is there and help.

**Q: What does everyone else think?**

Michael: I’ve not had any experience with any other hospital for any comparative point of view, but all I can say is that when I was diagnosed with diabetes about 15 years ago, I had a blood glucose reading of about 23, which they suggested I might like to do something about rather rapidly and I came here and the first person I saw was X who took me under control very quickly and got everything in shape and when I had cancer, they didn’t tell me at the time, they told me later I had the chemo and radiation not work, I had three months to live and X was on the phone every day, whether I rang him or he rang me, he was on the phone every day checking my levels, making alterations to the medication and actually taking the time to come down to the radiation area, which was quite a walk from his office, to see me on a couple of occasions, just to consult me. He gets my tick of approval.

Dana: When you talk about the wonderful P X I’ll back you all the way. I went to have major surgery on my eye at the Eye Hospital and that night after the surgery, after he finished a whole day at this hospital, a whole complete shift, he was over at the Eye Hospital beside my bed.

Michael: Which I might add is about half a mile from where he lives, but that’s. <laugh>

Dana: Yeah, I know, but you know what I’m saying. He didn’t have to do it

Michael: that’s true

Dana: He doesn’t work there, or he did at the time, he did one clinic on a Friday morning for half a day, but he didn’t have to go there on his way to see me and for that I’ll be eternally grateful.

**Q: Does everyone need to leave at two?**

Michael: I’m sorry?

**Q: Does everyone need to leave at 2 o’clock?**

Michael: Reasonably close to it.

Dana: I need to get out of here by five to, to be transported to Staff station for an EMT.

**Q: In that case I might just move on a bit just so we can cover all the questions. Now lets move on to your experiences with living and looking after your diabetes and chronic kidney disease. So in moving from talking about the health services, now it’s your own experiences with living and taking care of your own diabetes and chronic kidney disease. Now in your opinion, what do you need to look after your diabetes and chronic kidney disease well?**

Michael: A nag for a wife. If it wasn’t for my wife keeping an eye on me, I think I’d probably slip off the straight and narrow more often than I do. She’s forever reminding me that you really don’t need to eat that, I think you’ve had enough to drink and etc. etc.

**Q: You agree James?**

James: Oh yes. I went to dinner last night and exactly as that experience.

Michael: You’ve met my wife too have you? <laughter> I don’t know about everyone, I’ve met diabetic people outside of this room who are so scared of it that they’ll eat a lettuce leaf and a piece of fruit and forget about everything else and only drink water. To me that’s not living, now you’ve got to be able to live within what’s wrong with you and still enjoy life because if you stick to the letter of the medical law as closely as some people indicate, then you may as well just jump off The Gap and end it all. So that’s why I think we tend to rely so heavily on our specialists to keep us back on the straight and narrow or to make up for our shortfalls.

Dana: I’m in a rather difficult predicament because I can’t actually stick to a diabetic diet. I have to have high protein, low fibre diet in order to control another part of my body and so it’s really hard.

**Q: Sorry, do you mind if I ask the reason, or if you don’t want to share, that’s okay.**

Dana: No, it’s irritable bowel syndrome, but I have the gastric one rather than the constipation one and so fibre and stuff like that aggravates me, so I eat what I can eat to keep the bowel under control and I’ve got to be very careful too, but then I expect my sugars to be up a little bit more because I can’t have low GI food, I can’t have fibre food and I have a high protein diet because my kidneys are leaking protein. I’m anaemic and I need iron infusions and all that is connected to my kidney, but as X said to me on numerous occasions, you can have the odd treat and not feel bad, as long as you don’t do it every day.

**Q: So what does everyone else think in terms of what you need to look after your diabetes and chronic kidney disease? I think Michael and James have expressed perhaps a supportive relative or family that will help them keep on the straight and narrow or supportive specialists. Anything else?**

Zeb: I had a nice easy one to have. I, when I had Multiple Myeloma and had the chemo I lost 20kg in ten days and I just couldn’t eat, but I’ve never been a big fat person, but I had a massive stomach because I was on high steroids and everything for the chemo, CPT thing and then I lost this weight and it’s like a God gift, it’s like winning your wife because I’d been off all tablets, it’s purely dietary control my sugar was down and for the want of the chemo, I don’t seem to be able to put the weight on and therefore I don’t have a lot of problem with sugar. So it’s a nice natural story, isn’t it really?

Dana: It’s lovely.

**Q: So in terms of what do you need other people in other places I mentioned, education or motivation, having finances and things like that, do any of you guys identify with any of those things?**

James: I think education is an important one. We’re a bit fortunate in one respect that we had a daughter and we had to live through her experience and, of course, there’s a wonderful endocrinology unit, it used to be down the back here, Reserve Street, and had a wonderful team down there. It was fun by Dr, I forget his name now, but we were virtually tutored. We’d see the doctor and he would be educating to do this, this is the diet, we had to study portions. They had sisters, they were then nursing sisters who were trained in endocrinology and we would spend many sessions with them being trained on how to even feed our daughter. That was important because, as I said, she spent about 50% of it, between ten and 15, in this hospital and when she was home we’d try to keep her out of hospital by giving certain diets and we learnt about portions. Now my wife feeds me in those same portions even though I’m type II, I keep saying saying I’m type II, not type I, you’ll have those portions, it doesn’t matter, you’ve got diabetes and I’m prone to being overweight, my father was 19 stone and I’ve climbed up to about 17, but I try to keep it down as much as I can. It’s not easy to do when you’re on portions and my wife says you’ve got to eat that portion, not that’s the minimum, you’ve got to have two portions of this and three portions of that and so on and that actually makes it hard to keep my weight down.

Dana: Yeah cause diabetes you’ve got to balance your food.

James: You’ve got to balance it yeah.

Dana: I am a fat person, but I’m one of the very, very rare people who is medically fat.

James: That raises some issues as to what is fat? You get these models of if your six foot high you’re not supposed to be more than about 82kg or something and you try confining yourself to that, you’d be eating lettuce leaves, that’s how bad it is, they’re not real and I find that every time I’ve gone on a diet, oh yeah I’ve lost weight, but if I stay on that diet, the weight creeps up again, it’s like a sponge, you seem to squeeze that weight out and then it just seems to come from nowhere and the older you get, the less you absorb and it manifests itself into increased weight.

**Q: So has anyone got anything else they want to add in terms of what they need to look after their diabetes and chronic kidney disease? We’ve mentioned supportive relatives, supportive specialists, education, anything else?**

Zeb: When I was having problems with reading around 38, 42, with sugars, I’ve had some real problems.

James: I ain’t been up that high.

Zeb: That’s under chemo as well, but that’s what I had to struggle with for a while.

James: Gee What’s the maximum, pardon my asking?

Zeb: Sorry?

James: What’s the maximum before danger level?

Dana: Twenty-three up.

**Q: That’s getting pretty dangerous.**

James: Seventeen’s the highest I’ve been.

Zeb: Everybody’s running.

Dana: I’ve been 32 before I was diagnosed.

Zeb: What I want to say is that I did find that sometimes to my personality, some specialists pushed, not specialists, but really doctors and even the nurses, push everything too far. I know they’ve got to make it to an average, but I felt it was just pushed that little bit further than what it should’ve been that put me right off and you start feeling contrary and saying well bugger it, I’ll eat what I want and start taking that attitude and it’s really amazing that when I did start taking that attitude and then, as I said, lost weight and then sugar to drop down to being fairly normal, part of it, I think, may have been my own attitude that helped to do all that in that cycle I was going through. So what I’m saying is that there’s a little bit against too much guidance. I say that and I think that it’s certain people in the positions they get, they get carried away with it and they don’t look at it from an average point of view to spread it.

**Q: Other people mentioned self-motivation; do people think that’s important?**

Michael: I’d agree with that.

James: We want to be here.

Michael: If you rely totally on the medication I think the inevitability of you going downhill is going to see you in the face. You’ve got to help it along, for it to help you, you’ve got to help it. We don’t all achieve that.

**Q: What about things like finances and stuff like that?**

Dana: I’m always broke. <laughter>

**Q: Some people also mention that as an important sort of factor as well, being able to afford things and stuff like that.**

james: No, no.

**Q: Most people disagree.**

James: I’ve only raised the point that, as I say, the first three months of the year you’ve got for a couple about $600, I forget what the limit is, but we’re spending all that in the first two or three months of the year. It hurts, if you can spread it over a year it’s only about $50 a month or something, but when it’s $200 a month, then you start to feel it, that’s the main thing.

**Q: So James disagrees and you disagree Zeb, was everyone else disagree with that?**

Michael: I raised the issue earlier that I think the cost of medication can be expensive. I’m still working, but a proportion of what little money I earn goes in medication and every time I go to the chemist it’s about $150 and I look at that and think wow.

**Q: Some people also mentioned the cost of parking or cost of getting transport.**

Michael: That’s a good idea, are you guys paying for our parking today?

Zeb: yes, As I walked away today I thought what a wonderful system where you drive into a hospital, you park, they don’t charge you and you get in the car and drive home. <laughter>

Michael/James: Where are you parked that you don’t pay?

Zeb: <laugh> Umm I’ve got a …

James: Oh yes, I’ve got a disabled one too.

Zeb: Yeah, it’s disabled, so you drive in, you park right near the hospital (James: yes) and it’s an excellent system.

Michael: How do I get one of them? <laughter>

James: I think you might be able to get one for diabetes.

Dana: No, you can’t. <overtalk>

Q: So do people agree with the fact that the cost of treatment or parking is an issue?..... NO?

James: No

Michael : Five bucks is not going to break me.

Zeb: Mine is not a high thing when it comes to health in any way really, is it?

Dana: I think it’s important, sorry.

James: Yeah

**Q: In some other places it has been, just because they’ve had so many appointments. Each time they come they might pay 20 bucks parking and then they spend a lot of money on fuel driving to appointments in some other places. (James saying yes after each sentence)**

Zeb: Yeah, I suppose it’s sign of the times.

James: Yeah, petrol is, yes.

Dana: Well I still owe my pharmacy 153 bucks for drugs, so there you are. It’s just not easy cause the diabetes medication is fairly expensive. My Minidiab is something like $11 for a packet and that’s not the PBS $5.90, it’s $11 and any other drugs got more money added onto it so you pay a fair bit for medications and then there’s medications that are not on the PBS and that makes it difficult. I’ve spend, if you only have to spend $600 to get your exemptions, you’re doing really well, I’ve spent over $2,500 to get to my PBS level for the free list.

James: Yes, you’ve got a lot of drugs that aren’t on the PBS. I’m fortunate, I’ve only got a couple and then they’re only two or three dollars more.

Dana: You’re lucky, I’m paying $52 for my vitamin D. I’m paying $15-16 for Caltrate and Caltrate for kidney disease should be on the PBS.

Zeb: This is one side of medicine in Australia, I mean I go to a lot of other countries, I’m a traveller and I can tell you that we’ve got a wonderful system, when you see it physically and you’re there.

Michael: Try going to America.

James: Went to the doctor once there.

Zeb: Anywhere else practically, but one thing that bugs me is, what do you call that, the medicines that are made under pharmaceutical, like Blackmores and those and that does annoy me in Australia tremendously, the fact that it’s not regulated so much and they charge outrageous prices, like you were just saying, if there’s any political side to me that would bring me out, this is one and when you think you’re taking drugs to keep you alive and it’s not costing you next to nothing, you’re taking bloody drugs that they make out of natural stuff and charge for the earth for it.

Dana: Shouldn’t be taking that stuff if you’re diabetic.

Zeb: You what?

Dana: You shouldn’t be taking that stuff if you’re a diabetic.

Zeb: You just said you were taking vitamin D and stuff.

Dana: Yeah, but that’s given to me by a doctor.

James: Yeah

Zeb: Oh yeah <overtalk>.

Dana: And if it’s not given to you by a doctor, you shouldn’t touch the stuff. Most of that natural stuff damages <overtalk>

James: yeah, yeah, I see what you mean

Zeb: Magnesium is and all those other things are, but they’re not on the PBS and I think it’s an interesting point.

James: My wife’s just been put on vitamin D as well.

Dana: No, that’s okay to be put on by a doctor.

James: Oh she has, yes.

Dana: But what I’m saying is a lot of people go and take that what I call complementary medicine, I don’t even call it that, I call it murder medicine, and they pay this big money and all it does is damage your kidney, so it’s no good if you’ve got kidney disease to take any of that natural product.

**Q: Sorry, again, I might just move this on a bit, I’m just looking at the clock, that’s all. Now this is a similar question, but I’m flipping it, what things make it hard for you to look after your diabetes and chronic kidney disease well?**

Dana: In my case the loss of my community nurses.

**Q: The loss of your community nurses?**

Dana: Mmm.

**Q: What happened there?**

Dana: I had a surgery over here, got discharged, they didn’t stitch up my wound, I got the usual discharge summary from here, they didn’t want to listen to the discharge summary, they backed me in a corner, I got upset and I said I’m not prepared to have you using the letter F, murder me and she took offence of it and now I’m a dangerous person and they’re not going to come to me.

**Q: Is this like the district nurse that comes?**

Dana: Mmm.

**Q: Oh okay, so basically loss of some medical support.**

Dana: So I’ve lost the checking of my feet cause I’ve had an amputation, the loss of checking my stomach, caring for my wound properly, the wound should’ve closed up by now, but it’s still open, just things like that.

**Q: It sounds pretty hard Dana. Anyone got anything else they want to add, any factors that make it hard for you to look after your diabetes and chronic kidney disease at all?**

Michael: Just your own weaknesses.

James: I have a tendency to forget my medicine sometimes, but my wife will come running and roaring.

Speaker: I try to reduce my beer intake because it’s loaded with sugar, so I don’t drink very much beer these days. I like a glass a wine and I do have a sweet tooth, but mostly I’ve been able to control that, occasionally I break out.

**Q: Valerie, have you got anything to add, you’ve been a bit quiet? So what things make it hard for you to look after your diabetes and chronic kidney disease well?**

Valerie: Nothing, my sugar is no higher than 6.1. <clapping>.

Speaker: I wish I could do that.

Valerie: I watch what I eat and I eat plenty of fresh and I have a glass of wine once a week.

Dana: You go girl!

Valerie: On Wednesday I have lunch with my girlfriends, I go to the RSL club and I go to bingo and I win <laughter>. They tell me to go home.

**Q: So other people have mentioned things like conflicting information from doctors, lack of support, having a lot of other illnesses or having a lowered mood, do any of you guys identify with any of those factors, so how lowered mood or lack of support or having a lot of other illnesses?**

Speaker: One of the things that I would like to say, I get sick and tired of it over the years watching people, doctors and researchers having a mic stuck under them and saying we’ve discovered a cure for diabetes. This has been going on for the 40 years I’ve been involved with diabetes and there’s always these prominent people, they’ve discovered this and they’ve discovered that, I wish they’d just shut up because now there’s no credibility. Over the years we’ve watched the research development and it’s there’s been virtually nothing. There was the Islet of Langerhans at one stage and the transplants of them, I don’t know where it’s at with that, but that was 30 years ago and I don’t know whether it’s been abandoned, you never hear of it now and there’s been a few other developments, but you hear these announcements and they sound so positive, but they’ve got to the stage now where they’re offensive, but they’re still coming.

Speaker: If you’re trying to flog a new product, no one’s going to buy it if you’re negative.

Speaker: Yes, we people would like to see something that they’re saying about reversing diabetes, they’re talking about eliminating juvenile diabetes and is that true, is any of it true?

Dana: It’s a long way down the track. The research is a long way down the track.

Speaker: Yeah, that’s what I think.

Zeb: They’re all crying out for money aren’t these with these sensational headlines. I’ve got a little one for you all anyway that may make it interesting. My <overtalk>, my profession is a winemaker, I was talking about all these things <overtalk> that was the difficulty from the winemaker and <overtalk> it’s a bit hard, but I managed through it.

Speaker: I like a glass of wine, I love it.

Zeb: I don’t drink the end result.

Speaker: I still do.

Zeb: What I love is winemaking <overtalk>

Speaker: I can only drink white, I started off drinking red when I was a youngster, but I’d be ill for about three weeks, so I had to give it away.

**Q: So I think we were talking about factor that make it hard for you to look after your diabetes and I think you were just mentioning people saying they’ve got a discovery and then before that I was just mentioning that some other people had mentioned having a lowered mood or lack of support or having a lot of other illnesses that impact on their ability to look after their diabetes and chronic kidney disease, does anyone identify with any of the factors that I’ve mentioned? Dana’s put up her hand.**

Dana: I’ve got 20 illnesses. As I spoke to you before, the issue with me is finding an even balance between my diabetes and my bowel, that’s one issue. The other issue is my rare endocrine disorder that makes me feel hungry 24/7.

**Q: What’s that?**

Dana: Okay, I’ll tell you, being an endocrinologist, I have a non-function hypothalamus that gives me symptoms.

**Q: Oh yes, makes you hungry.**

Dana: Mmhm, of Prader Willi, so it’s all those factors. There’s all those factors and that’s the reason that I said I’m medically fat.

**Q: Yes, that’s hard when you’ve got a disregulation and you feel hungry and it’s very hard to curb.**

Dana: And yet I’m sensible, I had two sandwiches and a small snack pack of chips and that will last me now till I have dinner, yet I could go and eat another six sandwiches, but I don’t.

**Q: I think you’ve done well given that, you’re showing great restraint.**

Dana: I think it’s like depression does have an effect on you because when I’m depressed, that’s when I really want to eat sweets, so when I’m really depressed, that’s when I can’t control that hunger, so I do tend to eat a bit more and it’s then that I want to eat the sweets and then I keep having to say to myself, you’ve got diabetes Dana, stop it and stuff like that.

Speaker: God, I’m glad I’m not a winemaker <laughter>.

Zeb: Oh, you learn to live.

Speaker: Oh no, I don’t think so.

**Q: Sorry, you’re adding being a winemaker to the list as well? <laughter> Any other factors guys, ladies and gentlemen? Okay, now imagine if you became a director of-- now I’m just switching tact here a bit, imagine you became a director of the hospital and you had the power to change things about the diabetes and chronic kidney disease health service or you’re designing a new diabetes and chronic kidney disease health service. So if you were going to design the perfect health service, which would meet all your needs, what would it include?**

Dana: The only thing I would do is I would combine it, so I would have diabetes, the kidney people and the vascular people and the eye people in one area of the hospital where they can all be connected to one another rather than it spread out all over the place, that’s the only thing I would do.

Speaker: Makes sense.

Speaker: It does.

Michael: I’ve never thought of that because I don’t have multiple or connected diseases, the only one being kidneys and I find, at the moment, X and Dr T are sitting in offices next door to each other in the specialist centre, but even when X was in clinic one, it’s not an inconvenience in this hospital necessarily because everyone’s relatively close to each other. I think that’s probably the case in most large hospitals. I would think in hospitals in other areas take Hospital 3a, which I’ve had a little bit to do with, not for my own self, I have a son who was there for a while, it’s a one level hospital and it’s spread out over acres and you could find, I imagine, one specialist there and the other half a mile away and walking between them would be a bit of a problem, but I think this hospital is pretty good. The only thing I would do to improve if you were going to have setting a new directorate or whatever, I’d make P X renounce any idea about resigning and put him as the boss.

Dana: Yeah, I second that.

**Q: Heard from Dana, heard from Michael, what does everyone else think, Valerie or Zeb or James? If you could design the perfect health system?**

James: Well I couldn’t find anything wrong with the other hospital and I was amazed that such a wonderful building and I know I have heard from the doctors …

**Q: Which other hospital?**

James: The other building, the old building, the old Hospital 3, I remember when it was built and I’m a bit surprised that they decided to pull it down and then build this one, but coming in here and seeing the differences, I didn’t find much difficulty with the other one, but coming in here and the compactness of it, you go into the ambulatory care area on this level here, I think it’s brilliant they way they’ve done it. So easy to, oh yeah, I just go there, you just look in one spot and you could find where to go, it’s so easy, whereas it was like a rabbit warren in the other place, although that never worried me. I thought it was a waste of the building coming down, but they tell me that it’s had serious problems, the weight loads on the floors and so on, so it had to come down, but it’s marvellous, absolutely marvellous. I have been an inpatient here as well, I had a back seizure about three or four months ago and brought in here and had to get a spinal injection and so on and zip, zip, zip you’re in that spot and you’re in the right spot very quickly and you can move around the hospital, it’s so compact, but again, the ambulatory care section here is magnificent and I think it goes a long way to dealing with the problem that you’ve raised. So I think they’ve actually had your thoughts in mind in the way they’ve developed this, this is wonderful.

**Q: So it sounds like you won’t improve anything.**

James: I’m usually a whinger too.

Michael: It’s very difficult for people like us who are outpatients to be able to give comment on something that is really an internal organisation.

**Q: Oh not really, cause we’re asking about the outpatient services, so if you were designing the ideal service, what would things include, so I guess you’re the end users, we do want to hear what you guys have to say. Valerie and Zeb, any comments?**

Zeb: Not really, I’m not bright enough to get too deeply into that one.

Speaker: Too much wine.

Valerie: I’ve a power of attorney, he lives in Southwest Rocks and they’ve got to contact him any time that.

**Q: Power of?**

Valerie: Power of attorney, he lives in Southwest Rocks, so I’m going up there the end of next Monday, he’s getting married.

Zeb: It’s a lovely place.

Valerie: Yeah, I’ve been there before.

Dana: Where’s that at?

James: Southwest Rocks up near Kempsey.

Dana: North coast?

James: Yes.

Dana: I’m going to Urunga in January.

James: That’s a lovely spot.

Zeb: Nice area, the whole area.

**Q: If you were the director of this hospital and you had the power to change things about the diabetes and chronic kidney disease health services and you were designing a new health service, would there be anything that you would include in that health service?**

Valerie: No, everything is really good here. I’ve never had any problems with Hospital 3, they’re all wonderful here.

Zeb: It’s always managed to work somehow, always, even when you have to go down the bowels for your x-rays or <overtalk>.

Valerie: Being a diabetic and I’m an asthmatic as well.

**Q: So other people have mentioned from other hospitals and again, this is very hospital dependent, I think some people, well some people did mentioned combining the renal and diabetes clinic, other people mentioned regular pharmacist contact or regular nurse educator contact or better education or cheap parking. Does that speak to any of you guys?**

James: No, not really.

Dana: Can’t drive, I’m blind.

Michael: Normally when I come to see my specialist I park in the half hour free parking zone anyway, so it’s only when I come to see you that I’ve got to pay for it.

**Q: Another quick question, what do you guys find particular effective about the diabetes and chronic kidney disease health services here at the Hospital 3?**

Zeb: Find effective?

**Q: Yes.**

Michael: I think the fact that they keep very good records and they know where you stand at any one time. As I said, I think the most effective thing is that they know, at any one time, where you’re at. I find that very impressive.

James: My thoughts exactly, my words were going to be exactly the same as Michael’s.

Zeb: Whoever was the footprints for the medical histories and combinations, the whole hospital thing is just a touch of brilliance.

James: They’re brilliant this hospital, brilliant.

**Q: Valerie and Dana, do you have anything that you want to add?**

Valerie: No, haven’t got anything else to say, everything’s okay.

Dana: As far as this hospital is concerned, I believe it’s perfect. I think the most important thing is you have faith in your doctor and you respect your doctor and you do the best you can to listen to them and not be afraid to ask questions from your doctor, because they are the ones that know the answers and they’re the ones that are going to put you on the right track, so I think communication’s really important.

**Q: Actually, just a quick question, has anyone had any issues with bad communication here or the doctor not telling you enough information? Now this has been an issue in other hospitals, is it an issue here?**

Zeb: I can remember on this pure basis when I was diagnosed with myeloma, someone from the Leukaemia Association and we are and she was talking very personal and she said what doctor are you under and I said so and so specialist and she said I was under him but I left because he couldn’t tell me enough. I said I’m sorry, that’s exactly why I’m with him, because I have an attitude that I like my doctor to worry about it and I’ll just get on with living, which will help him get along with his work, so that’s my attitude and compared to someone else who’s totally the opposite.

Michael: A funny little story I’ll pass on. When I was diagnosed with cancer of the throat my surgeon took me down to radiation and the radiation oncologist was giving some sort of class with students and so forth and he really didn’t have time to see me, but he sat me down in a chair in the middle of the little auditorium down there and all these students around me and he stuck a camera up, down my throat to have a look at the tumour and put it up on a big screen and I couldn’t see the screen and he’s just talking medical lingo with all these undergrads and so forth and da, da, da, da, da, da and then he yanked the thing out of my thing and said, okay I’ve got to go, come and see me tomorrow and walked out of the room. So he was a Kiwi, what else do you expect? They beat us at football and they’ve got no manners <laughter.> Anyway, the next day I went and saw him and before he started talking to me from on high, I said, listen, I think you and I are going to see a bit of each other it appears, we’ve got to understand each other and he said, what do you mean? I said, you’ve got the worst bedside manner of any doctor I’ve ever met in my life and he said, what? I thought that’s either done it for me or, you know, from that moment on <snaps fingers> we clicked, he was fantastic. He was shocked that someone should think that of him and he backed off at a million miles an hour and we sat down, we had a really good conversation about it, I’m sure he spent far more time with me than anyone else and he treated me well. As my head, neck and throat surgeon said to me later, that guy saved your life, so, I guess the story is, if someone gives you the shits, hit them straight back in the face with it.

**Q: Just really quickly I remember one of you guys who just saw people privately, who was that? So you’re purely looked after privately, no public clinics at all, is that correct?**

Dana: No, I’m going to a public clinic now.

**Q: But I think there was one.**

James: I did originally see Dr Frank privately, but as I’ve got older I find it easier to work with the hospital and have it all combined.

**Q: The only reason why I raised this is just in five minutes, and I’m just looking at the clock here because I know some of you guys have to leave, any differences that you guys notice between private care versus public care?**

James: Absolutely not, no.

Dana: Not at North Shore, no.

James: I’ve given away the medical fund for that very reason. My wife’s still in a medical fund, she wants to be able to choose private hospital as against public hospital, me, I don’t give a hang. I’ve been to private hospitals, I’ve been here, I’m happy here.

**Q: So as far as you’re concerned James, the Hospital 3 is as good as private?**

James: Yes, it’s not worth paying insurance.

Dana: Yes, it is, to have a surgeon of your choice.

James: But you end up with the surgeon.

Dana: No, you end up with the registrar love; believe me, here as well.

James: I had a seizure of the back and I was going, privately I was going to a rheumatologist and in fact I only saw him two days before the incident happened, but I had a row with the hospital here, there was a fellow wanted to throw me out of the hospital saying look go and have, because I came in as a private patient and I said okay and he just said go out and get your own x-rays and this sort of thing, we’ve got a 24 hour delay on x-rays and so on and I said no I’ll wait. So finally I got through to my specialist and I changed from private to public and he was quite happy with that and I was straight in, straight into hospital, I had to wait the 24 hours for the x-ray, but I got a hospital bed because of it, but I changed because I just felt I’m running into some difficulty here. This doctor is saying if you’re a private patient you can go out in the street and go to a x-ray fellow anytime, but I was fearful, they unlocked the back, but I was fearful of it locking again, just go out on the street and what are you going to do?

Dana: I haven’t got private health insurance except for extras.

James: Oh I have that, yes.

Dana: But you should have the choice of a doctor in a public hospital.

James: As I said, when my back seized, I was able to get onto my private specialist and I switched from private to public and he was happy with it and I got a bed instantly and he looked after everything and it was the usual doctor. I have a heart condition and I’m a public patient and whenever I’ve come in here I’ve had about six heart operations and it’s the same doctor each time, it’s the doctor I usually go to, so I don’t have any trouble. The hospital here seems to look after you if you’ve got a usual doctor. He’s the fella that gets allocated if you’re a public patient, it’s the same, they’re pretty good here like that.

Dana: But what I’m saying is when they actually do the surgery, it’s not necessarily that doctor, that’s what I’m saying.

James: Oh, I see, yes.

Dana: The registrar could do the surgery.

James: Oh okay, well I’ll take the risk.

**Q: Has anyone got anything else they want to add, otherwise we’ll close up?**

Zeb: Just high praises for Hospital 3.

**Q: Yeah, sounds like it.**

James: Definitely.

Michael: Who are you with in T City?

**Q: Hospital 4.**

Dana: Can you get an order to move me?

**Q: We’ve got some surveys for you guys to fill out.**

END OF TRANSCRIPT

**Focus Group 4**

**Q: As an opening question, could we quickly go around the circle? We already spoke about our names, but perhaps if we could quickly share how long we’ve had diabetes and chronic kidney disease for, and how long we’ve been attending the Diabetes and Chronic Kidney Disease clinic, if we are. If we start with Arun perhaps, in no particular order?**

Arun: What do you like to know?

**Q: So how long you’ve had diabetes and chronic kidney disease for, and how long…**

Arun: When did I get it, or…?

**Q: Yes, when did you get it, and how long you’ve been attending the clinics.**

Arun: It started in my country in 1985, only I- I didn’t know that I had diabetes, but I had sweating, and very often urinating, and all sort of thing. So I was wondering what it is, I didn’t know what it’s about. Then I went to clinic where they test the blood, somebody said, “We will test your blood and see whether anything.” So I went and tested. I went, there are people who are testing has gone somewhere, so they said to wait. I waited for one hour and nobody there, so I went to the front coffee shop, and had some tea and something to eat, and everything, and came and waited. They took the blood. After taking the blood they said it take for three hours you wait, we’ll give you results. After three hours already doctor, she came in immediately put me on diet. She said, “You are in a coma state, you cannot move.” I said, “I don’t feel anything, what is this?” “You are having diabetes.” Then only I knew that.

**Q: Okay, so that was in 1980…?**

Arun: Yeah, 1985.

**Q: ’85. And how long have you been attending the Diabetes and Chronic Kidney Disease clinics?**

Arun: From ’85 I’m just monitoring from there. Only pills I am taking them.

**Q: Okay. And what about attending the clinics here at Hospital 4, how…**

Arun: Yes, here in Australia, after coming to Australia, I came to my GP, and GP referred to the Hospital 4 Diabetic Clinic, and every six months I have been called here. And their *0:02:25.

**Q: Okay. How long have you been attending the clinics here at Hospital 4 for?**

Arun: From ’98.

**Q: Wow, that’s quite a long time. Okay.**

Arun: Yeah, from ’98 I am attending.

**Q: And that’s the diabetes clinic, what about the – do you also <over talk>?**

Arun: The diabetic clinic, when I was in the diabetic clinic, one of the doctor did a urine test and they said “One of your kidney’s leaking proteins, so that – you have to go to the renal clinic also.” So they referred there, and every three months I am attending that also from 2000. 13 years.

**Q: 2000, okay. How about every one else, how long have they been attending the diabetes and the renal clinics for?**

Rod: For my story’s a little similar like him, but with a later start, some in 1995, like that is diabetic. First clue about that, given my GP. She tell me when I look at all my lab tests, she tell me that I’m going through the diabetes. At that time was just solely around the seven, little bit below the seven, but going to be increased, that’s happened. And after then all the time I was monitoring about that. And from kidney disease, happen somewhere about 2000, 2001, something like that, the first inflammation about then. Because my kidney *0:03:51 on the top, it produce more protein- protein than usually do it. And after then getting worse and worse.

**Q: Yep, okay. And how long have you been attending the diabetes and kidney services here at Hospital 4?**

Rod: After 1995 with diabetes, and kidney disease, about ninety- actually 2002, 2003.

**Q: Okay. How about you guys, how long have you been attending the diabetes services here, and the kidney disease services?**

John: Well the kidney disease service only this year. But as far as the diabetes is concerned, it would be about 16 years.

**Q: Okay, quite a long time.**

John: Around about something like that.

**Q: What about you Joe, or Costas?**

Joe: Well I started with diabetes way back, I can’t remember the dates. But my GP used to tell me, “You have to lose weight, otherwise I put you on medication for diabetes.” So I lost weight, and he didn’t put me on medication. But eventually – Then we discovered the - my kidneys were deteriorating, and they put me on medication as well, but that didn’t help. So after about a year or so, he decided to return me to GP at Hospital 4.

**Q: For both the diabetes and kidney disease?**

Joe: For the kidney disease, yeah.

**Q: For kidney disease as well, okay.**

Joe: Then diabetes, that came later. Yeah.

**Q: Okay. No probs. And Costas?**

Costas: More than 20 years.

**Q: For?**

Costas: <laughs> Diabetes. More than 20.

**Q: Okay. And the kidneys?**

Costas: Kidney?

**Q: Yes.**

Costas: I can’t see them.

**Q: You can’t see them, okay. So as far as you know, kidneys are okay, good. Now we’ve been talking a bit about our diabetes and kidney disease, I want every one to think about their experiences with living with the diabetes, kidney disease, and also looking after their diabetes and kidneys. In your opinion, what do you think you need to look after your diabetes and kidney disease well?**

Costas: Don’t drink beer and whiskey. <laughs>

Arun: And avoid white sugar, and salt, to bring down the pressure.

John: Well I walk every day if possible, an average of 40 minutes a day.

Arun: Exercise is most important.

John: And I- and I try and not eat foods that are – But sometimes you can’t help it if you’re at a function or something, but that’s the way I – See, I didn’t know I had kidney disease until about three years ago, where I had a quadruple bypass surgery, and they found – When they did that, they found that I was “kidney impaired” was the word that they used. But they never told me what my count was. And it was only when I was talking about that – so you know, just on three years ago, and then I went to the kidney section here to find out what my count was. And the lady doctor told me that my count was that I can still have a good life with it.

**Q: Okay, yep.**

John: So that’s the kidney part.

Costas looking awy and tapping – paying attention? Rod and arun listening, Joe looks away

**Q: So what about for everyone else, from your experiences what do you need in place to look after your diabetes and kidney disease well? For example some people say, you know, they need education, or they need a supportive family, or they need a supportive medical care, and things like that.**

Costas: <over talk> injection. One injection, go to the cemetery quick.

<laughter>

**Q: Okay, what does everyone else think?**

Rod: The way I will live.

Joe: Well I’ve got no problem with anything as far as diabetes, and I just do my injections three times a day, and get on with my life. Same with my kidneys. I’ve got a kidney transplant now, I just take care of that, and get on with my life.

Rod: What I say. For me it’s very tricky, when I coming to the diabetes clinic, every time when I’m coming its another doctor. And he look at just only on the computer, last check up, and listen to me what I tell him, how’s it going with the sugar level in the booklet that I use every day, and after then going to main doctor, and talk with him, and bring to me his opinion. And I will – I never been introduced the main doctor, when I spoke with him, then he look at me how I looks like. That’s a little bit tricky. I mean, there is – looks like, then he just told me in numbers on a clinic, nothing else. Maybe I’m wrong, but…

(Costas looks away)

**Q: So you feel like just a…**

Rod: I feel like just a number, just only 15 minutes at a check up, and “Thank you, we see you in three months time, one months time,” depends on the situation, and go home. Without any improve that they decrease or increase the medication. That is similar happen two days ago when I was the 23^rd^ when I was in the Hospital 4a diabetes clinic, and I tell him the result of last month’s what I’m supposed to do, and what I’m doing, there was a satisfactory to say what I’m doing, but “We see you in September.” I really don’t know who will take about me. All hospital, all doctors, or someone else, because all the time it’s someone else, not… (looks sad)

John: Oh, I see.

Rod: Then I go just to you, or to there.

**Q: So you feel there’s no – You feel almost that you’re just part of a system, and that’s…?**

Rod: Something like that.

**Q: Does anyone else feel like that, or does anyone else have a similar experience?**

Joe: No, no.

John: Yeah. I feel that given the number of patients that the clinic has to see, I think the progress is pretty well, because they test you for other things, like weight, and feet, and blood pressure, and all that sort of stuff, before you actually see the doctor. So by the time you see the doctor here, he has got a certain amount of information on hand that he can assess the patient fairly quickly. So therefore I think the alternative, if you want to see the same doctor every time, well it’s sort of calls to see a private specialist. But in a system like the hospital, I don’t think it would be economic for them to process all the patients. But the way they do it I think’s fairly sound, because they do have these checks before you actually see the doctor.

**Q: Does anyone else feel like what Rod said? Does anyone else identify with what he said? Sorry can I also – I forget, we should also say what our name is.**

Arun: My name is Arun. You know, when I go to the clinic, the doctor call me there, and he looked at me blood report and everything, and he will say, “Oh right, right, right,” like that. Check the pressure, and all sort of thing, and immediately he can adjust the medicine. I say, “Why?” “Oh it is – protein is too much, I’m decreasing to 12.5 from 25, like that, you take like that.” But we don’t understand why they are doing that, so we want some more explanation. Or if doctor has no time, we can ask somebody else on a different time to explain what is happening from the blood result, and why this increase has come, what it is protein leaks, and why it is happening, and all that. So we don’t know anything about the medical background, you know?

**Q: Yes. And you feel that the doctors don’t have time, or not explaining to you?**

Arun: Yes, almost no time for doctor to explain.

**Q: Does anyone else have Arun’s experience as well with the doctor?**

Joe: No. When we go to the renal clinic, first thing we do is we get a blood test. Then we see the doctor, and it check the screen for inflammation, he does, he check you out, blood pressure and so forth, and your stomach and that for the kidney, and then he tells you whether you need change of medication, or anything. If he doesn’t – if there’s a change in the medication, sometime they call me on the mobile. Yeah.

**Q: So do they tell you why they’re doing it, and things like that? Like I think Arun was saying they say something, but he doesn’t know the reason why. Do they tell you the reason why, or I guess you’re not…?**

Joe: Mainly because of the – what do they call it? Creatinine.

**Q: Creatinine, mmm.**

Joe: And they alter the, no, not mycophenolate.. no no the tacrolismus.

**Q: Okay. What about yourselves, John, or Costas, or Rod, anyone had – any of you guys had a similar experience to Arun, where you find that they don’t have time to explain enough, or give you education, or tell you why they’re doing something?**

John: I haven’t had a negative experience in all the time I’ve been here. I consider that – I mean, you do your blood test, the hospital has that the week before you see them, and so they know all that information. I make it a custom for the blood test to also go to the local doctor as well, and therefore both my local doctor combined with the hospital here, I think I’m getting a pretty good analysis of my condition.

**Q: Sure. Anyone else got any other comments for that, what we’ve just been speaking about?**

Joe: No, I’ve got the same experience too. They share the information with my GP, which is pretty good.

John: There is one – only one negative feeling I have with the hospital, but that applies to the other hospitals in the group, is that if you want to call the hospital you can never get on, you can wait two hours to get through. And I think that if you could get through a bit more quickly, you – that would be more satisfactory.

**Q: Okay, well while we talk about negative experiences, why don’t we move on to that question then. So based on your experience what would you say are weaknesses of the diabetes and chronic kidney disease health services?**

Joe: I can’t see that they’ve got any weaknesses.

John: I agree.

Arun: I think they are checking only the blood in the diabetic clinic, but diabetic patients should have something to check with the eye, the foot, all this. But I have never seen podiatrist or optometrist person in the clinic. At least once a year, or something, somebody must attend and just check the diabetic people about the eye, and the foot, and everything, what is happening. Because we don’t have that facility here, in the clinic.

John: There is an assistant who before you see the doctor checks your weight, sometimes takes a little sample of blood to see what you are, your blood pressure, and they ask you, “Have you seen a podiatrist, or had your feet seen to?” So they – I find they cover that. They just ask you the questions, they don’t look at you, and that also goes on the report before you see the doctor. There’s a special lady who does that, yeah. And it saves the doctors time, you see, and he has – By doing that, the doctor can be more efficient, because he’s got all the information in front of him, and see more patients.

Joe agrees, Costa shakes his head

**Q: Have you guys, Costas you haven’t said much, or Rod? Any comments about weaknesses?**

Rod: I don’t have any comments, because that doctor is very good, going on the check up, do it most of the time all the things what John said, in Hospital 4a, or check up the foot. And for eyes I was in the eye clinic in Hospital 4a, I check myself. But not really checking in the hospital, must do it myself.

**Q: Okay. So some other people have commented that some of the weaknesses could be that there’s too many appointments, there’s sometimes different doctors tell them different things, or I think as you said, someone said that they felt that they didn’t – they weren’t interested in the patient as a whole, but the doctors were just worried about a certain part of the body, and that was it. Or some people felt that even that they could have better education. Do you guys identify with any of those experiences, just thinking more about some of the weaknesses?**

Arun: Because every time different doctor…

John: (nods) yeah but

Arun: comes to the clinic, so we never meet the same person at every clinic, you know? So each doctor have a different opinion about medicine, one will say, “Oh this medicine no good you take this one.” So I said that – the doctor told me to take only 12.5, no, no, you take 25. So every doctor having a different opinion. I don’t know whether they are studying on our body, how it’s reacting, or something like that. So if it is, if they go through the file and see what the earlier doctor has done it, and why he has done it, and then he – I don’t know why they are all ruling, or sometimes.

John: Do any of the patients here, do they show their book to the doctors? Like every – three times a day I take my blood count, and I write it down in my book, and I find that if I present that to the doctor they get a better indication of how I’m going.

Costas: No comment. <laughs>

Rod: Most of this situation is similar, just on a little bit less or more what’s happening in, or when you’re going on a ordinary check up to doctor, but most of the nearly the same, just only a few check up, and that’s all. And after then we see them in two, three months. When I put that comparison between the diabetes clinic, I’m going to the same hospital, in Hospital 4a, I’m going to cardiovascular clinic, all the time it’s a different doctor, similar things. One guy work in a job about my medication, look at what I use, how many used, and he put his opinion, then I’m going something to change or not. And all that information is going with me to my own doctor, to Dr Jackson, who is the chief of the cardiovascular.

**Q: Sorry the mind doctor?**

Rod: Yeah, main doctor, Dr Jackson.

**Q: Oh main doctor?**

Rod: Main doctor, yes. Going to him, and he spoke with me. And I expect that’s happened that in a diabetic clinic. Then I spoke with main doctor. But never happen. Most of the people, they got similar things, never seen main doctor and spoke with him, and someone must know who will take care about you. But in diabetic clinic in Hospital 4a doesn’t happen, maybe in the other clinics it happens that.

John sighs

**Q: Okay. So has anyone got any other weaknesses that they want to mention about the diabetes and kidney disease services? (silence) Joe, or Arun, any other comments? Or Costas?**

John shakes his head

Joe: What do you mean by weaknesses?

**Q: Weaknesses. So things that you don’t like, or think need to be improved.**

Rod: Probably always satisfied.

**Q: Sorry?**

Rod: Probably always satisfied.

Arun: no weaknesses.. I don’t see any weaknesses.

Joe: Like I say, I can’t complain. I mean, I don’t see the same doctor every day, every time I go. And they’re all pretty good. I mean, like that.

**Q: Okay… So a related sort of question. From your experience, what do you find particularly frustrating about the diabetes and chronic kidney disease services? Anything that you find frustrating?**

John: No.

Arun: Waiting. Waiting for the doctor to call.

John: Well

Arun: You’re appointment is 11 o’clock, you go there, you will be called at 12 o’clock, 12:30.

John: Well. <laughs>

Joe: That’s like everywhere else.

Arun: waiting 1 hour

Joe: That’s like everywhere else.

John: Yeah, like everywhere else, yeah.

Arun: So they are running out of funding.

John: Yeah, well…

Arun: That is the only thing… that’s all that I have

**Q: Anyone else got any other comments?**

Rod: No.

John: No. No, I’m very happy.

**Q: Okay. So we mentioned one or two weaknesses I think, so the waiting times, what was the other ones we mentioned? And I think you also felt that there wasn’t a main doctor looking after you, and sometimes you were a number.**

Arun: Yeah, every time it’s different.

**Q: And then someone else mentioned also perhaps different doctors tell you different things. What do people think could be done to improve on these weaknesses?**

John sighs

Rod: It’s very hard to tell, because we are no doctor.

**Q: Yeah, of course. But what – if you were to suggest any improvements, what we can improve it, what would you guys think?**

Joe: Improve what, to see your own doctor?

Joe and Rod look like not quite sure about question

**Q: Well**

Arun: Improve means one doctor, if he does something, he should explain more to us why he is doing that. Because he’s overruling the earlier doctor’s decision.

Joe: Isn’t it up to you to ask that – to give him that question?

Arun: Yeah, he must tell us why he is doing that. He must say that medically this is wrong, this is right, this level is high, this level is low. We don’t know. Diabetic level goes up to eight or nine, they well tell you are diabetic is more, your control is very fine. You continue like that. They always say, “Ah, your kidney is stable, you continue the same thing.” Somebody say, “No, you’ve been leaking too much ,you increase the Atacand to 25 milligram from 12.5.” (John sighs) So every time they change, we have to go for a prescription, and throw the earlier one, and bring the new one, like that.

**Q: Sorry Joe, you were saying something in response?**

Joe: No. To find out what he has to tell us, isn’t it up to you to ask him the question what’s – why is he changing the medication?

Arun: Yeah, yeah, the doctor must explain to us.

Joe: Yeah.

Costa: They’re too busy.

Arun: Why he is changing any medication, what is wrong with my kidney.

Joe: But you should ask him the question why.

Arun: Because he don’t have time to explain all those too, because he has, I think, limited time for – there are so many patients. If you talk about half an hour, about three patients will be frustrated.

Joe: Doesn’t matter <over talk>, I don’t think so.

Arun: So here, we go fast, you know? So we have to think about that also.

John: I think the more information a patient can give the doctor, the more doctor understands your situation.

Joe: Yeah.

John: Like, as I’ve said before, I bring my result book in, and they can look at the book, and they can see all the highs and the lows, and all that sort of stuff, and I think the doctor is limited by the amount of information a patient can offer. But I haven’t found one doctor in the clinic yet that I haven’t got along with.

**Q: Okay, yep. And does everyone think that the – in the diabetes and kidney services, that the doctors and the staff are giving you enough information about the disease, and things like that? Yeah?**

Joe: No.

Arun: I think not enough information. They have to give more information, and only we also can continue with that, you know?

**Q: Okay… Let’s, Let’s**

Arun: Because every clinic is six months difference, so once you go now, after six months we forget the whole thing. So we must be educated, or done something to continue doing that. The kidney clinic is every three months, so why is that diabetes is two for a year, and it is four for a year? Can’t they combine both together, or not?

**Q: Well I think that’s why we’re doing this. <laughs> That’s why we’re talking to you guys. Costas, have you got anything you want to say? You’ve been a bit quiet.**

Costas: No. Because its like take tablets keep quiet.

Joe: <laughs>

Arun: Is it that the diabetic doctor, and kidney doctors are different, or both the same doctors can look after both diseases?

**Q: They’re different specialities.**

Arun: Oh, that’s what I’m asking you. Diabetic is different, and this is different?

**Q: Yes.**

Arun: Oh, okay.

**Q: They’re linked, but they’re different. So for a related question, everyone, does everyone find that it’s easy to get the diabetes and kidney services that they need? So when I say “easy” I’m talking about in terms of affordability, waiting times, and also location.**

Joe: As far as location, I’ve got to drive half an hour to get to the hospital. That’s the only problem I have. Because you’re not here in time, then you have to wait.

**Q: Yep. But in terms of parking costs, affordability, everything else is okay?**

Joe: Oh yeah, well parking, I’ve got a friend of mine that lives in Clayton Road, not far from the hospital, I park my car there.

**Q: <laughs> So you’re lucky. What does everyone else think?**

John: Well I caught the bus today. I mean, I’m three streets away from the bus, and it takes me straight out the hospital, and the same when I go home, so I’ve got no transport problems. My wife can…

Rod: For me is a little bit tricky situation, because I live in Boronia.

John: Oh wow.

Rod: I’m very close to Maroondah Hospital, but I never been there because my neighbour and the other friend who lives around me tells me that Maroondah is not good enough like a hospital. I really don’t know why. And most of the time I will go into Hospital 4a, and to Hospital 4, and a little bit far, about 30 minutes to Hospital 4a, and 35, 40 minutes to come here. Before then, I live in Boronia, I was living in Hospital 4a area, and Hospital 4 and Hospital 4a was very close to me, and I am satisfied with the service. Much better than I find what’s happened in Maroondah Hospital, because I really don’t know who is the doctor there, and how it’s working, and plus the people says it’s not good enough.

John: Yeah, well <laughs> You should hear the things that I hear about the Western Hospital over at Sunshine.

Rod: Yes (laughs).

**Q: What about other…?**

Arun: I travel from Lyndhurst, it takes about half an hour, or 25 minutes, along the M freeway. And the clinic is only at the Hospital 4, not the other places. In Cranbourne also there is the Southern Cross, I ask them whether they are in clinic there, they said no, only you have to come to Clayton. So every time I have to travel half an hour and wait for about one or two hours, three hours gone. And I’ve got parking fine also two times.

**Q: Oh no.**

Arun: $65.

(several people laugh)

Arun : Parking will kill us everywhere.

John: Yeah

Arun: I park in a church, and I’m behind that church three hours. So when I came there more than three hours, $65.

**Q: Oh no.**

John: $65?

Arun: Yeah, not now, five years back. After that I never parked there.

John: I was going to say, they’ve got a car park here now, which is not bad actually.

Arun: Now I park near the railway station and start walking, good exercise.

John: Oh yeah. Yeah, that’s good, that doesn’t take long.

Arun: Yeah, railway station, walk, then go back and get that done.

John: Yeah, fine. That’s good. (smiles and nods)

**Q: Costas?**

Costas: I have no question. I’m easy.

Joe: You’re easy coming, easy going, hey?

Costas: <laughs>

**Q: Is there a comment about how easy it is to get to the appointments, or the clinics, the parking, and stuff like that?**

John: Yeah, well.

Costas: I’ve got a special ticket… special parking.

John: It’s the same every hospital, half a mile from the hospital, you’re lucky to get a car parking situation. (laughs)

**Q: Okay now, this question might be a bit funny. Have you ever missed out on your diabetes or chronic kidney disease appointments, and if you did miss out, what were the reasons why you missed out?**

Rod: I missed one time, because I mixed the date. I thought – for example I thought it is the 16^th^ of January, but it was the 12^th^.

John: (laughs…) .Yeah well.

Arun: Yeah sometimes….

Rod: But I just put in my mind, not put on the paper, that’s the reason.

Arun: Yeah, sometimes we forget the dates, you know? And after one week only we come, my god it’s finished, it’s like that. Nobody remind us before one week, or…

Rod: Correct.

Arun: So somebody has to remind us by telephoning, or sending a letter, or something like that, “Your clinic is supposed to be next week.” Or email at least, if you have the email address.

John: Which gets back to one of my original things, the telephone, it’s very difficult unless you do it in writing to contact the clinic because of the phone situation. You know, you’re lucky to get it in two hours time.

**Q: What about you guys in terms of have you reasons… for missing appointments?**

Joe: No, I’ve never missed an appointment, no.

**Q: Never missed, okay. Now if you guys – now most of you guys do go to a kidney disease appointment as well, is that correct?**

Joe: Yeah.

**Q: So if you had – just say if the kidney disease clinic appointment clashed with the diabetes appointment, so they all happened to be at the same time, and you had to pick one, which one would you go to?**

Costas: Kidney.

John: I would go to the diabetic, because the last visit that I had here they did make the kidney clinic an hour and a half after the diabetic clinic. So we have lunch here while we waited, and that worked out beautifully.

**Q: Yep. What about everyone else?**

Arun: Yeah the same day, they can have both together. One in the morning, one in the evening, like that, it would be easy.

Joe: Well mine, the diabetic clinic, it’s on the Thursday, and the renal clinic, it’s on the Friday.

**Q: Okay, but just say if they both clashed together, which one would you pick, diabetes or kidneys?**

Joe: I think I’d go to the kidney, it’s much easier.

Arun: Kidney.

Rod: I prefer kidney as well, because diabetic I may control myself.

Arun: Yeah. Kidney’s are better – worse thing, you know? It is more important.

Rod: I take less carbohydrate, my sugar level going down.

**Q: Okay, good. Now we’ve talked about some of the weaknesses of this diabetes and the chronic kidney disease services, based on your experiences what do you think are the strengths of the diabetes and chronic kidney disease services?**

John: I believe that the strengths are the fact that they – you have a blood test a week beforehand, and when you come to the clinic, the lady does your weight, takes your blood pressure, all that sort of thing, asks you questions, and then she has that put on the file, and I find that then if you go – when the doctor comes, you – I give him my diabetic result book, they can tell how I’m going. So I think the information that we can give the doctor has an important part on the quality of service.

**Q: Sure, yep. Any other strengths?**

Rod: I agree with John. That’s happened like that. If someone doing some mistake, everything going wrong. But if you’ve got a procedure like that, everything will be all right.

(Arun nods)

Arun: Yeah, that’s – what John said is correct. All the information already given to the doctor beforehand, we go there. So that means that he can diagnose us better than asking.

**Q: But what do you find good – Okay, I’ll rephrase the question. What do you find particularly good about the diabetes and chronic kidney disease services?**

Costas: Eat well, Eat well.

(several people laugh, including Rod and John)

**Q: So what do you find particularly good about the services, or effective about the diabetes and chronic kidney disease services?**

Rod: We find everything is good because we don’t know what is the difference.

John: <laughs> That’s a good one.

Rod: We don’t know anything.

(everyone laughs)

Arun: Yeah, we don’t know the difference.

Rod: <laughs> Different to what we thought is bad. Maybe they give us that one, that menu with that menu, give you – we take with us, and it is a good one. But not when they give us something bad, and how it may be bad.

[Joe doesn’t always smile (made a face not sure if he disagreed)]

**Q: Well what do you guys particularly like about the diabetes and chronic kidney disease services?**

John: I like the efficiency of the system.

**Q: Okay.**

Arun: Yeah.

Joe: Yeah. Efficiency is good, yeah.

**Q: Any other comments?**

Rod: Yes, certainly now, yeah.

Rod: I really don’t know what makes change in that system. That system is a good one, is not 100% good, depends on the people who working in that system, but it’s a good one. Give us a lot of medication in a good price, and we may afford it. There is that medication is 100% secure for us or not, we don’t know. But we can’t change. (Arun nods)

**Q: Okay, another question for you guys, and sorry just remember to say your name before you make the comments, sorry. I know it’s hard to remember. So what impact had attending the diabetes and kidney services made on your health?**

Joe: I think it’s a great impact. ‘Cause you can’t eat what you want, you can’t – wherever you go you’ve got to take your insulin with you, and your medication, you know and just you’ve got to keep- keep taking it all the time.

**Q: Yeah, anyone got any other comments about what impact, so whether it’s a good impact, bad impact? As in terms of attending – so what impact has attending the services made to your health? Has it made it better, has it made it worse, in what way has it made it better or has I made it worse?**

Arun: The clinic, attending the clinic, we have been given more medication, and everything, which is good for us, because otherwise we won’t know the condition, how we’re reacting. Because in the next blood test we can see how the medication has already worked, and they say it’s stable, and your diabetic control is very good. So we have been monitored in a way so that our lifestyle will be a good one, with exercise and – otherwise we would have been very bad life, without the clinic.

**Q: What does everyone else think?**

Rod: I thought there is clinic is very good for us, in our sickness. Big help for us is our good.

**Q: Yes.**

Rod: They give us lot of effort for that, then we health is put on the side, just live a bit, we must put a lot of effort for them. Looking on the food, looking on the exercise, looking on everything. Some people need to do it extra, like John, but most of the people, they can’t do it, they’ve got some different operation, and heart disease, and something like that.

John: Yep.

Rod: For that people it’s very hard making any effort for exercise, because the heart, they don’t have enough power for that. And every people, every patient is a different story. In that case I thought that there is most big issue for us is own effort. Your own effort that you make, to live with that disease.

**Q: Yeah. So while we’re talking about your own effort and things like that in terms of living with your sickness, so going back to a question, in your opinion what do you think you need to look after your – so look after your diabetes and chronic kidney disease well. So some examples of some answers that people might have given before, like a supportive family, or supportive health service, or things like that (**Rod laughs at supportive family and service). **So what do you guys need to look after your own diabetes and kidney disease well?**

Costas: Eat well and sleep. <laughs> (Joe laughs)

Arun: We want a supportive health service that is – that only it will be with us. We don’t know anything about medications, and how we – so service if you’re provided by this clinic, that is could be a very great thing, you know?

**Q: Yep. Anything else?**

Arun: And also the pharmaceutical benefits, because of the medicines, if you don’t have the health care card, or anything like that, every time we are to go buy $32, $40 for like Medicare, it’s only $5.90.

John: Yeah, well that’s helped. <sighs> (Rod smiles) I’m a bit biased because this hospital treated me so swiftly, and so well when I had a near death experience on the long weekend – last long weekend. And you know, if I hadn’t been treated as quickly, I might have died. So you can see I’m up beat about the hospital.

**Q: Yes. So yeah, so has anyone else got any other comments about what else they need to look after their diabetes and chronic kidney disease? I think Arun’s mentioned supportive service, and at least making the medications affordable.**

(John looks puzzled)

Arun: Affordable, yeah. (Rod and John also nod)

**Q: Yeah. Any other comments about what you guys need to look after your diabetes and chronic kidney disease?**

John: Well first of all we’ve got our local doctor, okay? And then there is also a nurse that you can ring, I’ve got private health insurance, thank god, and they’ve got a system where the nurse rings you up, or you can ring them anytime you’ve got a query or you want to know something. And then I believe doesn’t this Hospital offer the same sort of thing, there’s a nurse you can ring, and they can tell you as well.

**Q: Yes, for the diabetes, yes.**

John: Yeah. So with all those things combined, plus the clinic, I think you can get a very, very (Arun agrees – says very good)

John: good constant treatment here

Rod: yeah

John: even just by way of advice, or actually coming to the clinic, seeing your own local doctor.

**Q: What do other people think, Rod, or Joe, or Costa?**

Rod: I’m agree with Rod and John what they said, there’s a pretty good doctor.

Arun: we want support

**Q: Yep. So just you need just support really,**

Rod: yeah support

Arun: we want support

**Q: Support frohospital. What about in terms of family support, or things like that, or education, or even self motivation?** (John looked with eyebrows up with mention of self-motivation)

Joe: Well probably self motivation that I need, because I don’t exercise or anything, I just sit down and watch TV, and sometimes I go out. That’s all. I don’t do anything at home.

John: That makes it hard, yeah.

Joe: And as far as help from here, I always get it. I can’t complain from the clinic, whether it’s diabetes or kidney, they’re all helpful. (Rod nodding)

**Q: Costas, Rod, anything further to add? No?**

Rod: I remember a few months ago I watched TV, I really don’t know that is a joke or not, but some America doctors talking about some – on the TV like doctors.

John: Oh yeah, Channel Ten, or something.

Rod: Something like that. And talking about some people sickness. And one of them ask the doctors, “What makes big help for the people over 60 age?” And one of the doctors said, two girls of 25. And I ask my doctor, GP, and I tell him, “Listen I saw on TV,” what I see, and I explain that, and he tell me, “Yeah, you are right. The two girls of 25 will more help you, than going to Greek owned cemetery.”

**Q: Sorry, I…?**

Rod: That’s a joke. <laughs>

**Q: Okay, yes. <laughs>**

Rod: In that case nothing to help us more than we want.

(No-one really understood his joke)

**Q: Sure. Now if – Okay, thanks for that. If no one else has got anything further to add I might switch questions. On the flip side, what things or what factors make it hard for you to look after – or actually, before I even skip to that, so we talked about supportive service, and things like that, and self motivation, and also affordable medication.**

Arun: Subsidised.

**Q: Subsidised..Are all these factors in place at the moment for you to look after your…?**

Arun: Yeah.

Joe: Uh-huh.

Rod: Yes.

John: Yep.

(all agree except Costas)

**Q: Okay. Now on the flipside, what makes it hard for you to look after your diabetes and chronic kidney disease well?**

John: Well I’ve only had one visit to the kidney clinic, so I can’t really comment much about the kidney. As far as the diabetic is concerned, it can- it can change very quickly, unless you put a constant surveillance on it. I find that the clinic is very helpful. When you see a doctor, you can ask them questions and they will respond. And I think the more you have questions for the doctor, the more the doctor can see what’s bothering you, or what’s ailing you in particular. And I’ve made the effort to really keep the doctor informed of my progress, which means I probably get a better result from the doctor than what I would if I didn’t.

**Q: Okay, thanks for that, John.**

Arun: I want to tell about the diabetes, because the diabetes can be checked every day, or every week, or I have got a glucose meter, and we know the level of sugar in the blood, and we can control that. Because every clinic is six months difference. Every six months only we go. But kidney clinic, we don’t know what is happening to the kidney, and we don’t know why it is happening, and we don’t monitor it at all, we don’t know anything. Unless we do go to the clinic, we won’t know what has happened within the three months. So after going to the clinic only with the blood test and they will say, “Creatinine is so much, it is so much as well, it is like that, your kidney is like this function.” So until then we don’t know what we are taking, what we are eating, because we don’t know how to control it like diabetes. Kidney controlling is not in our hands, because we are eating the normal food here and there, you know? That is our country traditional foods, spices, and everything we are eating. It will be harmful for the kidney sometimes, all the spices sometimes. So that maybe the reason why it is leaking more protein, or creatinine, or something like that. So the doctors have not given us full information, what are the diet that you are taking, you stop this one, don’t eat this one, like that. At least a dietician must tell. So these are the results of a physical exam.

**Q: So what you’re saying is that you don’t feel empowered by the kidney doctors on how to look after your kidneys, is that what you’re saying?**

Arun: Yes, that’s it.

**Q: Okay.**

Arun: Only they tell, “Now it is like this, it is stable, the creatinine is going too much,” they increase the medication, that’s all. So only the medication they are telling. They don’t tell about the other factors influencing the kidney.

**Q: Okay. Does anyone else have that…**

Arun: Like exercise, or something like that.

**Q: Does anyone else identify with Arun on that, that they don’t feel that they’re told on how to prevent their kidneys from getting worse?**

Joe: Yes.

Rod: Most of us. What’s happening in my case, my kidney working about 30%, 70% now is gone, but in that case nobody tell me what Arun said, what kind of food I must use, what kind of medication, except Crestor, just only Crestor, nothing else. There’s all drugs, only Crestor is medicine for kidney, or they got a lot of different medication. If my kidney working just only 30%, and put a lot of fluid in the body, what you supposed to do for that? Just ask what to take fluid out of my body, or crestor?

**Q: Well before you guys leave I can talk to you about that later very quickly, after we finish this group. Any other – so any other things which make it hard for you to look after your diabetes and chronic kidney disease? So I think basically what you’re saying is lack of information.**

Arun: Lack of information.

**Q: Some people have also mentioned lack of support, or being unwell, or having multiple medical conditions, and things like that. Has anyone else got any – identified any of those things, or any other things they want to add in terms of things which make it hard for you to look after your diabetes and chronic kidney disease? Other people say lack of support, lack of support from family or friends, lack of understanding of friends with the foods they’re eating, and things like that. Anyone else identify with those things?**

(Costa often looking down and around the room)

Arun: No, we don’t have those problems. Family is supporting, and everything is going very well. About others, I don’t know about other people. I don’t have any.

John: Yeah.

Rod: This is a maximum from the clinic, what they give us, we are satisfied, because it is the maximum.

**Q: Also other people say lowered mood, or lack of motivation make it hard as well. Does anyone identify with those factors?**

Rod: No.

Arun: No.

John: No.

Joe: No.

John: My only problem is the medication. I was told when I first went that I was on – had been on Diabex for too long

**Q: Okay.**

John: So they put me on Micro…

Rod: Diamicron.

John: Yeah, Diamicron. That brought me out in a rash which lasted for about three months. And because of that, they said, “Well look, you’d better go on insulin, because we don’t know what tablets to give you.” Now I don’t like – I’m quite happy doing it with the needle, then I’m at a meeting or a function, or something like that, and then it gets sort of rather embarrassing doing it. So that’s the thing that I don’t like. The only good thing about it, I find that if you take a tablet you get the same amount of the required medicine in your body, it doesn’t differ. The only good thing about insulin is that you can vary it according to your readings.

Rod: When John talking about things, I have one question.

**Q: If we’ve got questions, we can – unless it’s for the group, I can answer medical questions after we finish.**

John: Okay.

Rod: Okay.

**Q: Okay. Well someone else mentioned conflicting information made it hard for them to manage their diabetes and chronic kidney disease, does anyone identify with that?**

Joe: No.

Arun: Conflicting information?

**Q: Yeah. So different doctors told them different things and make them confused.**

John: No, I don’t have a problem with that.

Arun: No, that is all medical doctors, they can change the medication at any time. But we don’t know why they are doing it, they may be good for us I think.

**Q: So your main issue seems to be lack of – if anything, the main information seems to be lack of information given, which means that you can’t look after…**

Arun: Yeah, that’s right.

**Q: I guess my next question will be what do you think could be done to improve this situation?**

Arun: Yeah, that they must tell us, the doctors should explain, “You are being given the medication like this. But according to your blood test, your creatinine is so much because of this one, I am now increasing this one to make it less, stable.” So these are things that they must talk.

**Q: Yes, okay. Now we’re moving to a – we’re going to change tack a bit, this is a bit more fun sort of a question. So let’s move back to thinking about the health services that are offered for diabetes and chronic kidney disease. Obviously everyone’s made some comments about strengths and weaknesses, what they find frustrating, and perhaps what they find particularly effective. Now as I guess people who use the services, we would like you to make some recommendations. So imagine if you became director of the hospital** (John and Rod laughs), **and you had the power to change things about the diabetes and chronic kidney disease service, if we were going to design a perfect health service which would meet all your needs, what would that look like, and what would that include?**

Joe: I think the service that we get provided with, whether it’s kidneys, or diabetes, I mean, it’s all good. I can’t complain.

John: Me neither.

Arun: The clinic is good, we don’t complain anything like that, because doctors are looking after very good all the time. Only difference is different people come, different doctors. That is the difference. Every time we see, there will be some doctor another time, a male doctor, a female doctor, like that, everybody changes.

**Q: Can I just explore that a bit? What’s the main issue that you find with that? Like I know…**

Arun: But some say, “I am not a doctor, I am a qualified nurse,” or something like that. They’re also coming and doing diabetic clinic. So we have to listen to everybody, you know?

**Q: So what would you improve?**

Arun: One day I saw the chief diabetic doctor, or somebody, a lady, she is more looking after the person by looking at their leg, looking at their hand, toe, nails, everything. Pressure. Other doctors don’t do all those things. So I asked, “Are you a doctor here?” “No, I am the Chief of Diabetics in Hospital 4a.” So why she is doing that like that while other people are not doing that way? If she is looking after more than 15 minutes, taking the pressure, you first lie down, then stand up, two times she take it. And after that, she said, “Remove the socks, I want to see your legs.” So she is looking after, “Oh that little swelling is there.” Right, everything she is telling. So that as a very good, this thing, but she came only once I think for the last ten years I have seen, only once. After that I have not seen her. (all laugh, esp John laughs)

**Q: So what would you – so if you were to change something, as in – sorry, so I guess what are you – if you were to summarise your main point in one sentence, what would it be? So I’m just trying to understand what you’re saying, and the main point you’re trying to say. So what would be the main…?**

Arun: More supporting. The checking the things is not enough. The clinic, they just say, “Oh you are stable, it’s all right, continue.” That’s all. That’s not enough for us. (John smiles)

**Q: Okay. Any other comments from anyone?**

John: No, everything’s good.

Rod: He tell everything.

John: Everything’s good.

**Q: Okay. So no one would want to change anything else?**

Joe: No.

John: Only the telephone system. That’s it. If you want to cancel an appointment, you should be able to get through the clinic by telephone. If you want to – you know, that’s my only thing about the hospital, it affects all departments, is to be able to get through.

Arun: Can’t they inform us at least one week before the clinic? Because they always give a paper saying that your next appointment is September 15^th^. So now another four, five months, we sometimes forget. So is there any way of sending another letter?

**Q: Well that’s why we’re doing these sessions. So any other suggestions guys?**

Arun: No, that is better idea….

**Q: I mean other people have commented on having a combined diabetes/renal clinic, where you have both kidney and diabetes doctors together, (**Arun: that’s what I said**) or other people say better education, or stuff like that. So has anyone else got any other things they want to add in?**

Joe: No.

Rod: I can’t find anything else.

John: The only thought that comes to me is that things change, and opinions change on what’s good for both conditions. It would be nice if we – if there was a pamphlet or a booklet, if things – if the type of treatment changes because of changing trends, or new inventions, or things like that, be nice if there was a pamphlet or a booklet given out on diabetes, on kidney, and any of the other clinics that have got it as well, that might be a good idea.

**Q: Yep. Just like an update sort of?**

John: Yeah.

**Q: Sorry Arun, you were sort of – when I when I was mentioning some of those points, you were sort of saying something?**

Arun: Yeah, yeah.

**Q: Which one were you particularly talking about? Or which one were you speaking to when I was mentioning some of the points, like the combined renal/diabetes…**

Arun: Yeah. I thought in the morning to have the diabetic, and in the evening have the kidney. So the whole day we can just go to the clinic, and go once, and go home. Otherwise, in the morning from eight o’clock to 12 o’clock, like that.

**Q: What about if you saw them both in the same clinic?**

Arun: Yes, that is also a very good thing. But you said both doctors are different, can they come like that? I don’t know.

John: Everyone’s different.

**Q: Anything’s possible, maybe. <laughs>**

Arun: And also when they have the blood test, when they say, why can’t they give a copy to our GP also? But sometimes they give, sometimes they don’t give. So I have to ask them, “Can you give a copy for my GP?” and I have to take it from them and go. So as soon as they said why – then only the GP, I can go and monitor the clinic, it has happened, they have changed the medication, so give me a prescription, and like that I can follow up with the GP. So follow up with the GP. So some never come to the GP, the blood tests, and all sorts of things.

Joe: I always get a paper from the clinic here to give to my GP.

Arun: They give it to you?

Joe: Yes.

Arun: Oh, that’s good. That is a way that I like.

**Q: Costas, have you got any comments about if you were to make a perfect system?**

(everyone laughs)

Costas: No I don’t.

John: There is no perfect system.

Costas: I see them, they do what they like, they send me home. <laughs>

**Q: Okay.**

Arun: Are you satisfied with all of the ….?

Costas: Yes, I’m still alive.

Arun: Yeah good

Costas: I don’t complain. (everyone laughs) You can’t complain to the doctors. I go see my local doctor, he doesn’t even touch me. Three times I went, “Sign here, come back in two weeks. Sign here, come back in three weeks.” <laughs>

John: Three weeks, that’s pretty good.

Costas: So what can I ask him?

**Q: Would you like to ask him something?**

Costas: What? He said I’m okay. He said look here, you look all right. <laughs>

**Q: Okay, good-oh. Well we’re coming up to the end, this is the last big question before we’ll get you through to go through a very quick three question survey. So as you know, I mean the main purpose of this group is because we want you to help us evaluate these services for diabetes and chronic kidney disease, we want you to help us to improve them given that you’re – the services are really for you. So is there anything that we’ve missed, or that you came wanting to say that you haven’t?**

Rod: Just missing more information about kidney sickness, because we don’t know nothing about them. Nobody tell us anything, tell us about kidney. How it’s working, why working, what’s happened, how we’re diabetic, they got a problem. Why kidney got a problem with diabetes.

**Q: Yes, okay. Yep.**

Rod: Just more information about it, then we may survive much better than we do it now. Because sugar, we may control, we eat, we do exercise, a lot of things, but about kidney, we don’t know any idea how then can I help me.

**Q: Okay, yeah.**

Costas: They give you injection in the kidney.

**Q: Sorry?**

Costas: They give you injection in the kidney.

Rod: Cemetery.

Costas: Yeah, I’m taking it.

Rod: <laughs>

Costas: I bought it myself.

Arun: Yeah, if the information is there it is easy for us to see the kidney is a little better. Otherwise we don’t know, we are in the dark, you know? It is infuriating sometimes, like that. It cannot function for a long time.

**Q: Any other comments John, or Joe, or Costas, besides the injection?**

John: Well not...

Costas: No, no, the less I talk the better. <Joe laughs>

**Q: Sorry. John?**

John: I said I can’t comment on the kidney, because I’ve only had one visit.

**Q: Sure. Well what about for the diabetes then, anything else you want to say?**

John: Well the diabetes, as people know, can fluctuate from time to time, for not always apparent reason. And just on – So therefore I have to live with that.

**Q: Does anyone want to make any further comments about the services, or anything else, in terms of ways we can improve it before we finish up?**

John: Well I think the idea of combining the kidney visit and the diabetic visit on the same day means that the patients have less travelling to come to the hospital, apart from anything else, and you get it all done in one visit, and that I think is probably an advantage to the patient.

**Q: Yep. Has anyone else got any other comments before we close up? The last opportunity to make any comments in terms of improvements that could be done?**

John: Not really.

Joe: NO, Nope,

John: Because the clinic has to get through so many patients at a time, and therefore some patients’ troubles are more advanced or complex than others, so I think under the circumstances the hospital clinic does very, very well.

Arun: They are doing very well now, because even more patients are there, but they are looking after them.

John: That’s right.

Arun: And two or three, four or five doctors, each room they are calling.

John: Because the diabetic position is getting worse and worse.

Arun: Worse and worse, yes.

John: Every day. More people are getting it, every day more.

Arun: That is the main disease in Australia. 70 percent of the people are having diabetes.

John: Yes.

Joe: too much food on the table

**Q: Okay, well if no one’s got anything further to add, then we might distribute the quick survey.**

END OF TRANSCRIPT

**Focus Group 5**

**Q: Just as a starting question perhaps if we could just quickly go around the room and just say how long we’ve been followed up by the kidney doctors and the diabetes doctors. Or even if you go privately, how long you’ve been seen privately. I don’t know you want to start, but remember to say your name first before…**

Manny: 2010.

**Q: Is that for both?**

Manny: Yes.

**Q: Okay, yep.**

Freya: Diabetes was about 15 years ago, and the renal was about four year ago.

**Q: Okay, yep. And they’re both private, is that correct, Freya?**

Freya: Yes. Well the diabetes in Cairns was with the Diabetes Centre, which was connected to the Base Hospital there. And also the renal section was there as well. But in T City, it’s private.

**Q: And how long have you been in T City for, sorry?**

Freya: Two years.

**Q: Two years. Okay, yep.**

Akira: Now I’m not sure about the kidney, I think it’s been a couple of years.

**Q: Well how long have you been seeing the kidney doctors here for, for the kidneys anyway?**

Akira: I think it’s a couple of years. Now the diabetes, let me see now, I think I was originally diagnosed in the late eighties, when I was working at RMIT. Let me see now, I think – yes, after I was diagnosed I think I started coming here. So I think that answers your question?

**Q: Yep, definitely. And?**

Margaret: Diabetes is about 20 years, but it hasn’t always been here. That was at Maroondah.

**Q: Okay, in the public or private there?**

Margaret: Maroondah hospital is the public hospital. Then when we came here, it was about 15 years.

**Q: And you see Professor Kom…?**

Margaret: Professor PK privately.

**Q: Yep, yep.**

Margaret: There’s kidney, I’m not sure really, but I put down 15 years, because I think it started when I – about the same time as that, because of the – reckon it’s the medication that I had.

**Q: And how long have you been seeing the kidney – you follow up at the public clinic here, don’t you Margaret?**

Margaret: Yeah. Yes.

**Q: Yes. How long have you been there for?**

Margaret: I’m not sure, but I did say it was about… five or six years probably. I put ten or 15, but probably not that.

**Q: Okay, so everyone here’s had some experience with some of the public clinics here at Hospital 1.**

Manny: Sorry to interrupt. With the diabetes I was initially diagnosed in 2007, but I didn’t actually like do anything about until 2010. Just in case you…

**Q: No, thanks for that. Thank you. So it looks like everyone here has experienced some of the public services here at Hospital 1, or maybe at other places as well. So I’d like you to think about the experiences you’ve had with the diabetes and chronic kidney disease services, and I guess for yourself, you might be able to compare private and public, and I think yourself as well, and we might ask that a bit later. But what would you say are some of the weaknesses of the diabetes and chronic kidney disease services here at Hospital 1?**

Akira: Now I guess one of the things for me is that, let’s see, when I did biochemistry many years ago, we covered diabetes, and we just talked about what it basically was. I didn’t at that time know anything about the side effects. Now since I’ve started coming here, there wasn’t a lot covered in the side effects. There was more information given, but still it’s like, “Oh yeah, it will be fine,” you know? And they go, “Oh, you go blind in about ten years,” and go, “Oh yeah, it will be fine.” Whereas I recently went to a seminar on end stage kidney disease, and they talked about some of the consequences, some of the changes to lifestyle, stuff like that, which I found very helpful. Scary too. And while some of the stuff with the diabetes I discovered the hard way. Like, “You know how you did that exercise, well now you’ve got to have your toe off.” “Oh, okay,” that was an unpleasant surprise. <laughs> ‘Cause people can’t really predict what you’re going to do, if you do X, a bad thing is going to happen, because they say, “Look, there’s 50 million bad things that can happen, we don’t know which mistake you’re going to make, and therefore we can’t cover it.” So some of those things I found out the hard way.

**Q: So would you say a lack of education, or what are you…?**

Akira: I guess…

**Q: Or better education for the diabetes?**

Akira: I guess lack of knowledge of…

**Q: Complications?**

Akira: Yes, complications, and how quickly they can occur. Like the toe incident, basically I decided I was going to do some exercise, so I did some really brisk walking. I noticed a bit of a swelling in my foot, so the next time I went to see the podiatrist, I said, “Look, I’ve got a bit of swelling.” She said, “Can’t you smell that?” and go, “No.” She said, “You’re in hospital tonight, the toe’s coming off.” Wow, all this because I just tried to do a bit of exercise? (Everyone looks shocked, Marg said wow)

**Q: So perhaps more education about the complications, perhaps?**

Akira: Yeah.

**Q: Or what to look out for, danger signs, perhaps?**

Akira: Yeah. And also how quickly that things can come on. That was an unpleasant surprise.

**Q: What does everyone else think?**

Freya: Well in Cairns when I was diagnosed with the diabetes, I had diabetes educators and discussion groups, and things like that, and I think they really were very, very thorough. And they did explain the complications, and it wasn’t really until I was in the system with the- with the diabetes in the Diabetes Clinic, and I was working, and they finished work – they finished the clinic at about four o’clock, so I had to really scramble to manage to get there in time. Had very, very hard time getting appointments. And the staff changed so much that when I found out my kidney disease had happened was because the staff had changed, they didn’t communicate to me what there was a problem with, I found out more or less by accident about six months after they worked out that there was a problem. And I was really quite annoyed about it, because they had a lot of intern doctors who didn’t really explain – One just said, “Oh look, don’t take that medication, I’ll give you another prescription.” But he didn’t. And I didn’t know why I wasn’t to take the medication, and I had it so I kept on taking it. And it turned out it was really doing damage to my kidneys.

**Q: So was that a lack of communication…**

Freya: A lack of communication.

**Q: Between the doctor and you, or between doctors, or both?**

Freya: Between doctors, and between doctors and me, and between the change of doctors they would have – there was one head of department I saw occasionally, but he was always going to conferences overseas, and they had a lot of very knew beginning doctors, who were really nice, but didn’t really know a whole lot. In the end I think I knew more than they did about the diabetes. I didn’t know about the medication and prescriptions though. And I really was pretty damned annoyed that it was through lack of communication that I think I got it as bad as I did before I realised. (Manny looks at paperwork) So they were changing buildings, and things like that, so there were reasons, but it doesn’t really make a lot of difference when you’re the one that’s screwed up.

**Q: Sounds like you were happy with the education there though?**

Freya: The education was terrific.

**Q: So what sort of education did they have there?**

Freya: Well they had diabetes educators, and meetings, and we also had group discussions such as this in the beginning.

**Q: But with other patients?**

Freya: With other patients. Well let’s say the…

**Q: As in were they patients who’d had diabetes for a long time, or were they new ones?**

Freya: Well the one that’s memorable was when newly, just diagnosed diabetes people, and there were about eight of us all in this room, all a bit shell shocked. There were doctors and educators and all in there, and there was a lot of discussion and stuff. But it was quite a good learning experience. And then the diabetes educator was one-on-one. There was problems as far as timetabling as far as I was concerned, because I was working, but still they were very good. So it wasn’t really until they changed buildings and everything went a bit haywire.

**Q: Okay, well focusing back on the weaknesses, can anyone else identify anything that Akira or Freya said, or any other weaknesses people wanted to bring up?**

Manny: Yeah, my greatest frustration is the transfer of information from one department to the other, to my private doctor. To my GP. I will visit the renal doctor, and he’ll be looking at information and making a diagnosis, and then I’ll point out to him/her, “Hang on a minute, this is the information from two visits ago,” or it might even be three visits ago, “What happened to the information from…” Like I came in four weeks ago for example, and there’s nothing on the screen, he’s working blind. He’s working on past information. The same thing as far as from the renal to the diabetes, and the worst of the lot is from Hospital 1 to my GP. My GP looks at – I go there virtually every two weeks, and he looks at information, I can say I saw him last Tuesday, and he pulled his screen up, and the last information he had was 2012. Now since then I’ve been in hospital for two weeks, I’ve had a renal examination, I had a diabetes examination, and none of that has filtered through to him as yet. I mean, I can understand that there is a – you know, like obviously you can’t expect it to be there the next day, but we are in a computerised age, and you would think that there should be a better method of getting the information, because like I think I’m not quite sure who mentioned the change of medication, like the renal doctor – I’m going back a year ago now – changed some medication for me. Now that affected my diabetes. (Marg – no, no, no) It went back to my doctor, and my doctor said, “No, you can’t do that.” So he put me back on what the original was. And then I came back to the diabetes, and they changed my medication. And for the last – since – for the last three or four months I’ve been paying – like I’m on disability, I’ve been paying up to $100 a month for medication, which for me is way out of – where it normally is about $30 – because of all the changes in medications. And I have to do it, because I have to listen to what you, the experts, are telling us. (all nodding, in sympathy or agreement)

**Q: So do you find – From what you’re saying it sounds like different doctors are giving you different advice?**

Manny: Yes. Well I mean, the public system, you can’t be perfect because you can’t – if you had continuity then it will be of course the doctor will become familiar with you. But that cannot – you know? But in the last – like in the last visit I only check my notes because Professor Vue actually…

**Q: Manuel, yeah.**

Manny: Yeah. He was my examiner. When I was in hospital, there was a lady with – I mean, fair enough, you can’t do much about it, but there’s still not a same-ness. (Freya nods)

**Q: So your two main points are number one, trouble with information or communication between doctors, and number two perhaps different doctors giving you conflicting advice?**

Manny: Which probably agrees with what you said (Freya), where you know, like different doctors are giving you different…

Freya: Yes, this was in Cairns.

Manny: Yeah. But I mean, it relates to me anyway.

Freya: Oh yes.

Manny: So you know?

**Q: Can anyone else identify with anything that Manny has been saying?**

Margaret: Yeah, me. I found that it doesn’t matter which department you – clinic you go to, they’re still not passing on the information. With the kidneys, I – it was a big argument, because I’d had a heart transplant, and they were blaming the immune drugs that I was taking, the kidney doctors were blaming them for my problems with the kidneys. Where I knew jolly well that the transplant clinic wouldn’t do – change them. And so to stop all the arguments, and toing and froing, I had a kidney biopsy, and it proved that they were all wrong, because it was diabetes what was causing it. It’s just that sometimes they just don’t communicate between departments. And I’ve noticed that.

Manny: Or it doesn’t filter through quick enough.

Freya: In Hospital 7, because I am private in Hospital 7 with occasional correspondence from Hospital 1 to my renal doctor, everybody – well the diabetes doctor actually stays out of it, because my GP is so competent. But whenever I have any sort of pathology it goes to my – It goes immediately to my GJ, my renal doctor, and the diabetes doctor, the results. And the my doctor, my GP actually rings the renal doctor. When we were worrying about some sort of medication at one stage and he wanted to change it because the medication the renal doctor had given me, I don’t usually get side effects with drugs, but this particular one I did. And they said a dry cough can – and I was dry coughing all night and not able to sleep. (Marg nods) And my GP took me off it and put me on another one, and contacted the doctor to see whether that was all right. It was a similar sort of one. As it turned out, I went off both of them. But it is good communication, at least in the private sector.

**Q: We might talk about private versus public a bit later. Any other weaknesses that people can think of?**

Akira: Now this business about communication reminded me of something that happened that, you know, the longer you’ve been here of course the bigger your file gets. And at one stage I came in and somebody had mislaid my file for about three months. And so they were all guessing what they had to do. And eventually some doctor had put it on a desk somewhere, and they’d finally found the thing. Now if this were computerised, it wouldn’t be a problem, but the doctors are quite resistant to computerising it. And there’s also this privacy thing, but really there should be a central place, not just a piece of paper which someone can mislay. (Freya nods in agreement)

Margaret: I think it is now. I think the files are now going on computer.

Akira: Every time I go in, I’ll say, “I need a prescription,” and they’ll say, “What drugs are you one?” And I say, “Look, it’s in my file.” And they go, “Oh yeah, but it’s probably on page 15, and I’m not going to look that far.” You know? So the last time I checked, maybe there’s some stuff on computer, but they’ve got this file with a whole bunch of stuff in there which they can’t be bothered looking in. And I say, “Look, I need a prescription for my,” whatever it is, and they go, “Oh, what’s your dosage on that?” Can’t you look? Isn’t it on the last page of my file?

Freya: I found that too, there’s problems with remembering medication, and my doctor actually printed me out the updated versions, and I carry it around with me so I have what I am on, and the milligrams, and all. It makes life a lot easier. (Akira nodding mmm)

Manny: Yeah, same thing. I get very frustrated every time I go to hospital, because I virtually live here for the last two years, I’ve been – you know, I’ve got virtually an appointment every week, and the same question. Like, “What’s your medication?” (Freya constanltly nodding) Surely you guys are looking on the screen with my name on it, you know? Surely there’s something there that says, “Manny, dah, dah.” I’ve got 50 medications, unless I bring the script from the chemist, I’ve given up on names, and because there’s generic, there’s brands, I mean, you guys know it. And I’ve got five different names for the same medication, I’ve given up. (Freya and Akira nodding Akira says mmmm) There’s the list, if I bring it. If I don’t bring it, they’re lost. Oh sorry, you guys are lost, because somehow they cannot find it in the system. Whether it’s there or not, I’m not sure, but they cannot bring it up to have a look at it. (Freya nods)

Margaret: My doctors now have a copy on my file. <laughs> They – I always have a copy with me, and they just go and photocopy it and put it in the file. Unless of course it gets changed. <everyone laughs>

**Q: Any other weaknesses, everyone?**

Manny: This isn’t – I’m not sure if it’s a weakness, but again it’s a variance of how you get the information from different people as to how they perceive the condition you’re in. Like with my kidneys, the measurement, or the – you know?

**Q: Yes, the eGFR/creatinine.**

Manny: Yeah. Like my kidney has been growing a graph that’s been going down, down, down for the last two, three years. (Freya nods) Now initially I was told, I think the number was 15, I’m not quite sure at the moment, and I was told that’s getting to the critical stage. And I’ve said to the dialysis education person. Now since then I might have had about, I’m guessing another two, three, four visits with the renal, and they’re telling me that 15 is, you know? You don’t have to worry about it. It’s when you get to the fives that is a concern. (Freya - “oh really”, looks surprised and thinking about this),,,, You know? So I mean, I’m aware that it’s going to happen, and I keep on asking the question, what they think. “Oh, you’re quite stable,” I think I was 12 or 13 last time. And I was sort of – I was told, you know, “You don’t have to concern yourself, it’s going to be at least possibly nine, 12 months before you have any…”

**Q: So yours is 12. Does everyone else know what their GFRs are?**

Margaret: Not off hand, but I’ve been told they’re stable.

Freya: I don’t know what my last one was, but I was – when you were just saying something, Manny, you were saying about 12. I think I was about 2.7, or something, and my renal doctor was going on as though if I was going to get dialysis within two weeks. So I don’t know.

**Q: Yeah. Everyway, obviously this is a bit hard, I think we shouldn’t speculate without looking at the tests. So any other weaknesses that people can think of? So some people in other places have mentioned waiting times (Freya nods) and appointments, or too many appointments, or parking issues? (Freya nods) Do any of you guys identify with any of those?**

Manny: I found as far as waiting times go, I come here prepared to allow for at least two hours. (Marg nods) And if I do that, which with the renal actually is quite good, it’s probably the best of the – because I do opthamology, and I do the diabetes, they’re normally two to three hours. Renal is probably hour, if it’s over an hour, it’s a bit slow, in terms of the service that I’ve been getting. So in terms of waiting times, I’ve got no issue.

Akira: Now my experience is that if I come in the afternoon, it’s quite unpredictable and can be quite long. And sometimes you think, has everyone gone home? If I come in the morning, particularly like nine o’clock, maximum I have to wait is half an hour, it’s usually a lot less than that.

Freya: Well I’ve only been here twice, and the first time was in the afternoon, I had an awful job getting a parking spot, and when I actually found out where I was going, I was only there for about ten minutes before I saw somebody, then I was skidding – being moved from one place to another, and it was all a bit bewildering. But it was very quick. The second time I came, there was a massive delay through circumstance beyond everybody’s control, and I was there – everybody was there for about three or four hours with nobody moving. (Akira nods and smiles)

Margaret: I’ve found that with the renal clinic, they’re not bad. But I think it depends on the person and how patient they are. <laughs>

**Q: Okay. Now do you – does everyone find that it’s easy for them to get the diabetes and kidney health care that they need? When I talk about how easy is it, I’m talking about locality of the service, the cost of the service, and also waiting times.**

Margaret: I come here for both the diabetes and kidney, I’ve found that it’s okay. (Akira nods)

**Q: So it’s easy to get what you need, and people are contactable?**

Margaret: Yeah. And if I need anything, I just have to ask the doctors that I need it. I need any scripts.

**Q: And when you want to contact them, it’s easy to contact them?**

Margaret: Yeah, well I’ve got to get the renal clinic phone number. <laughs>

**Q: What about everyone else?**

Margaret: To get in contact with that. Paul Komesaroff, for the diabetes, I’ve got his mobile number. And he said I can ring him any time.

**Q: Yep.**

Akira: Now the location’s quite convenient, and generally I don’t have to wait too long. If I want to make an appointment, generally I have to wait like a couple of weeks. I have no idea what their emergency care is like, because I haven’t had to call on it. (Manny: hmmm, huh, laughs)

Freya: It’s not really relevant for me since I don’t come – I hope never to come here, because it will be dialysis for me if I come. And otherwise I’m capable at home – at Hospital 7.

Manny: With me it’s perfect, because I can walk, I can come by car, I can public service, transport, so there’s no issue.

**Q: So it’s easy to get here, easy to get what you need?**

Manny: Yeah.

**Q: The people are contactable?**

Manny: My appointment are pretty regular, within four to six weeks. Yes, there’s no.

**Q: Yep. Have any of you guys ever missed out on either your diabetes or chronic kidney disease appointments, and if so, what was the reason?**

Margaret: Professor Komesaraff goes away overseas quite a bit <laughs>, and usually my appointments, they’re only about two – they’re three or four months a part, and he’s got to sort of get in with the school holidays, and with his trips. But there’s only been a couple of times it’s been cancelled, but they’ve made another appointment for when he’s back.

**Q: Yep. And you’ve never missed out on your kidney disease appointment?**

Margaret: No.

**Q: Okay. What about everyone else?**

Akira: Yes, I’ve missed out on a few appointments. Sometimes I get my letter, and I don’t set up reminders, and things like that, so I’ve actually missed out on about three – I’ve missed out on my eye appointment, I’ve missed out a kidney appointment, missed out on a diabetes appointment. And I’ve thought to myself, look every time I go in, they just say “It’s pretty much the same old same old.” So just time went past, and then I went – I was invited to go to a thing on dialysis, and stuff like that, so I thought, oh this can get a bit serious. And then I found I was getting a little bit of an ache sort of in the kidney region, so I thought, yeah, maybe I should do something about this. So I made a new appointment. And then a tooth fell out, and I thought, look, I can’t let everything fall to pieces all at once, I’ve got to take care of one thing at a time. <everyone laughs> And I wasn’t sure the best way to do it. I know a couple of times I’ve communicated through email, and they’ve been quite good about that, but I thought, look it’s been awhile, maybe I might need another referral or something. So I went to my doctor, and the doctor goes, “No, no, you don’t do anything. We’ll write you a new referral, we’ll make you an appointment.” And after a couple of weeks I thought, I’m just wondering whether I should see someone? And then the appointment notification came in the mail, and everything was fine.

Manny: I find the service pretty good, I normally get a reminder on the phone.

**Q: As in you put the reminder yourself?**

Manny: No, from the hospital.

**Q: Oh, the hospital reminds you.**

Manny: Yes.

Margaret: I get a text message.

**Q: From the renal clinic?**

Manny: Yes. Renal, and…

**Q: Diabetes as well?**

Manny: I’m not sure about diabetes, ophthalmology, renal, and I’m not quite sure about the diabetes one.

**Q: Okay.**

Akira: Yes, now I just want to say I do get reminders, but my old phone, when I got a message I’d pick it up and it would say, “You got a message.” My new phone doesn’t do that, so if I don’t remember to check my messages, I don’t know.

Freya: When I was in Cairns I had to remember, there was no reminders. Locally my – the renal doctor doesn’t remind, the diabetes and the GP both send text messages. But I’m pretty organised, I really dot I’s and cross t’s.

**Q: Now some of you guys – someone else actually complained a weakness about the system was too many appointments. Does anyone identify with that?**

Akira: Yeah, look I find that sometimes I get appointments around the same date, so I turn up and I go, “I’ve got my diabetes appointment.” “No you haven’t, you’ve got a kidney appointment tomorrow.” “Oh, okay.” So I don’t have a lot of appointments, because generally like I sometimes come here for ophthalmology, I’ve got the kidney, and I’ve got the diabetes. But sometimes I’ll get the date of one and the time of the other, or something like that. When they’re about the same date, and I don’t write down – I don’t give myself reminders, and stuff like that, I get confused. Totally my fault.

**Q: Okay. So what does anyone else think about too many appointments, or not an issue?**

Manny: Are you asking us of the servicing?

**Q: No. Some people have complained a weakness is that there’s just too many appointments. And they’re wondering whether they can be squashed together.**

Margaret: I did that once, and I got into trouble for doing that. I had one appointment in the morning, it was a renal clinic one, and transplant one in the afternoon, and I got into trouble because the file had to go from one place to the other. That was their excuse. But no. (Freya and Akira laughs)

**Q: Okay, yeah. Another question, just say if your diabetes and your kidney disease appointments clashed, which one would people go to, and why?**

Akira: Well quite simple, they’re on different days, so it’s not a problem.

**Q: Yeah, but if it did clash theoretically?**

Freya: If that happened I’d actually ring and expect them to alter it.

**Q: Okay.**

Manny: If I was to make a choice it would be kidney.

**Q: Why is that?**

Manny: Because I feel that the diabetes I can control it myself more or less (Freya nods), whereas the kidney, I don’t know what I’m doing. You know, an internal organ more or less, and it’s an expert opinion, I wouldn’t have a clue what is going on.

**Q: What about yourself Freya if you had to pick?**

Freya: I would certainly pick the kidneys. I think that it’s – as Manny says, it’s the unknown really there. (Marg.. Mmmmm, nods) Because you don’t really – at least as far as I’m concerned, I don’t really feel any different. (Manny nods) But now I’m very conscious about the food I eat, and whatever.

**Q: How about you guys, Marg and Akira, if you guys had to pick one?**

Margaret: I’d say kidneys, because I can manage the diabetes.

Akira: My experience is the diabetes is a slow thing, unless I don’t take my medication or don’t eat, there’s no immediate problem, it’s a slow thing. So if I screw up one thing, it’s not going to have much immediate effect, except for that. Whereas the kidney, I’m much more uncertain about. (Freya nods)

**Q: Okay. Let’s move on a bit. What are some of the things that you find frustrating about the service?**

Freya: Are you referring to Hospital 1 service, or just generally?

**Q: Hospital 1 service. But if you wanted to talk about your experiences with private, or with Cairns, that’s okay.**

Freya: It’s – I found that with the diabetes, as I said, in Cairns you were educated. With the kidneys in Cairns and here, you really are thrown to the wolves. (Manny looking at notes) You’re just told you’ve got a problem, and in the Cairns one, the nephrologist there whom I saw twice in a year, did explain to me about the complications that some medications could cause with kidneys, and told me a few things about pain killers, and which ones never to take, and which ones were safe. But no mention was ever made of what foods to eat, or foods to avoid, or any other sort of education. And I felt that I’d inadvertently further damaged my kidneys because of that.

**Q: Is it any better here when you’re seen privately?**

Freya: The renal doctor, after the initial – well after he’d sent me to Hospital 1 and came back and we decided that we’d leave the dialysis preparation for a later time, did put me on to a dietician. And I had quite a long wait for her. And she gave me a lot of information, and did say that I could contact her any time. Well I didn’t, I sent her a couple of texts one time asking her if certain foods were really bad or not, because they weren’t on the computer, under the potassium levels. I mean, I’ve actually taught myself a lot, but otherwise there’s been no help really.

**Q: Where? At Cairns, or here, or both?**

Freya: Well certainly not in Cairns. And here, I had the dietician, I had to see a private dietician on that. And she gave me advice as far as food. And that was really the – I suppose, I don’t know whether the break through, but I know now foods to avoid. Whereas before that I had no idea that potatoes and tomatoes. I sort of knew that bananas were not good, but I didn’t realise about all the vegetables that you couldn’t eat if you wanted to keep your potassium low. And so it was the lack of education with that, I found that the diabetes, the medical people seem to take the diabetes far more seriously in a way. They’re teaching you about that. But the kidney’s, “You’ve got kidney problems.” “Okay.” That’s it. And you really don’t know. (Marg – yah, nods; Akira nods)

**Q: So does anyone else identify with that lack of education, with what – So what about those people who come here to Hospital 1 for renal and diabetes, are you guys educated?**

Manny: Well in terms of myself, like I said, whoever the doctors are at the time, they told me my levels are getting to a dangerous level. So I passed onto the dialysis educator, so that was part sort of – it was an experience for me, because I wasn’t aware of anything like that before. Just coming back to the dietician, I’m not sure, were you referring to the dietician here?

Freya: No.

Manny: Because I find it a little bit of a problem with the dietician here, you’ll be given a sheet of paper, which is a standard, or I’m assuming it’s a standard paper, with certain generalisations of foods,(Freya nods) where I’ve – personally I would feel better if it was a more accurate breakdown of foods. Because when you said, “You can’t eat bananas,” or like at certain times I was told that oranges and mandarins, what you call the citrus food, are not good for your kidneys. Now I was in hospital for two weeks, they were aware of my kidney problems because it was on the board, and I was getting an orange for afternoon fruit. You know? (all laughs; marg: orange juice) I was getting a banana for afternoon fruit. I didn’t ask for it, it wasn’t as though I ticked – because I was sort of – But that just sort of…

Margaret: But that’s the dietician?

Manny: Well I’m not blaming the dietician, this is while I was in hospital. But I also, when I seen the dietician here, which I haven’t for awhile because I’d been seeing a dietician at the community centre, it sort of in more of a generalised version, “Oh yes, this is good, this is bad.” But when you’ve got multiple problems (Freya and Akira nods), like I’ve got swelling on my legs, the diabetes, the renal, “Oh yeah, you can have a banana.” “Oh no, you can’t have a banana, because it’s not good for your diabetes,” or whatever. (Hmm, Freya laughs) “Oh yeah, have an orange, that’s no problem. Have a tomato.” “Oh yeah, but you can’t have a tomato, because the tomato is not good for gout, or whatever,” you know, they found for me not so long ago. That sort of very frustrating, and very – for me (Freya nods), for my diet, I’ve been as good as I believe I can be, obviously there are those improvements, but I can’t say with certain authority that I’m not going home and eating a block of chocolate, or four oranges, or six bananas, or potatoes, or sauces, I’ve cut out salt. You know?

**Q: So it’s just different advice that you get from the dieticians?**

Manny: It’s not formalised, you know? “This is not good for kidneys. To improve your diabetes you shouldn’t be eating this.” And then you can make a choice, “Oh yeah, this one matches with that.”

**Q: So you want specific advice on diabetes and kidney disease?**

Manny: Specific advice.

Freya: One of the things that I found also very frustrating, as Manny has, is if you follow the diabetes diet, you’re pretty much doing the complete opposite from what you should be doing with the renal diet. And also online, I’ve been studying it, and one site says – some say watermelon is good for low potassium or whatever, and another says you can’t have it. (Manny: sugar, nods and laugh) Same thing. I mean, not even with diabetes, just with the potassium for example. And they don’t know about it. And I mean, really hard getting recipes that you can have, and I’ve worked out – my doctor’s saying, “Eat vegetables,” and I say, “I can’t.” And this is my GP.

**Q: So basically what you’re looking for is someone who will give you advice suitable for both, rather than just for one or the other, and then you work out what to do?**

Freya: It would be wonderful if you could get recipes for both. I mean, I’ve picked up recipes that were supposed to be for that, and they are completely wrong. I mean, I know enough about it now, and…

Margaret: I get Diabetic Living book.

Freya: But I’m not talking about the diabetes, I’m talking about diabetes and renal, you need – because the diabetes ones sometimes can be almost – you almost think you’re poisoning your kidneys by using the diabetes one. (Marg looks surpised) For example, potatoes, pumpkins, sweet potatoes, parsnips, all of those things are really high in potassium and really bad for kidneys. Tomato’s no good.

Margaret: What can I eat? (Akira laughs)

Freya: Well I told – My GP was saying how as I going, and all the rest of it, and I said, “Well, I’ve worked out that the only thing that really is compatible with both diets it’s porridge.” <laughs, Akira laughs, Manny says that is good> And then you can’t really – And porridge with cream, because you’re not supposed to have skim milk, or yoghurt, a bit of full cream milk is all right on a very limited basis. So porridge it is, made with water probably. <laughs> (Freya looks frustrated and shrugs shoulders)

Manny: I just add quickly. With the porridge and all that, you can’t put sugar obviously, but you can put cinnamon on it.

Freya: Oh, okay. But you can also have…

Manny: Is this is something that you guys are aware of, or are we telling you something new?

Margaret: But who wants to eat porridge morning, noon, and night? <laughs>

Manny: But that brings other issues, you know?

Margaret: Probably. <laughs>

**Q: Any other frustrating things?**

Akira: I just want to say, let’s see, I went to a session where they were just talking about dialysis, and the affects it’s going to have on your life. So until today I thought, well look I’ve really got to look about my diabetes diet, I can eat anything I want until I’m on dialysis. Yes, I will have to give up coke, and things like that, yes I will have to give up tomatoes, and that’s it. But that’s when I’m on dialysis. So until today, I had no idea that there was any restriction. I mean, I come in, and they’d say, “Oh your potassium’s a bit high.” What does mean? “Well it’s higher than your sodium.” Yes, so what do I have to do? “Oh, don’t eat so many stone fruit.” Okay, that’s it? Okay. That’s it for the education, I didn’t actually have any education. They just said, “Okay.” And because I went to this particular session, they said, “Okay, if you’re on dialysis, here’s some restrictions.” That was it. They didn’t say, “Well before you’re on dialysis, you also have to avoid certain foods.”

Freya: Well my potassium went from – well according to my doctor, to dialysis urgent stage, to cutting out the potassium rich food that I knew about, to, well I think it’s now 4.7, which I think is normal. Mind you, I occasionally sin. I had some baked beans the other day, and I would have thought that I’d been spiking with cocaine, the guilt I felt. <Akira laughs>

Margaret: I haven’t been told anything about any foods I’m not about to eat for renal.

Manny: Well there you go.

Freya: Oh gosh.

**Q: Oh look, I mean, I think it depends on how exactly bad your kidneys are, and then whether you’re having troubles with the potassium. I think. So it does depend. If you’re not having any trouble with your potassium, then it’s less of an issue.**

Freya: Okay.

**Q: I know with Freya, she’s been having a lot of trouble with her potassium, so yeah. So any other – is there…**

Manny: Just an observation from at least three of us, <laughs> I think it comes back to with the education part of it being more specific in terms of listing – you know, even if we have to list either food groups, or individual, right? And have like especially with multiple situations, renal, and diabetes, and then you can sort of look at it and say, like I said before, it says, “Orange here, orange here. (Freya nods) It should be okay to eat. Pasta here, nothing there, it means should avoid.” That’s, you can make a better decision yourself. (Akira and Freya nods)

**Q: Okay, for the interest of time I’ll move on, unless – Do you guys all have to rush off particularly at five?**

Akira: Not particularly.

**Q: I guess if we can stay a bit longer than we can be a bit more freer, but I think we should move on anyway. Next question, some of you guys – I know some of you guys are seeing the public system, some of you guys see a private one, private another, or some people have done a bit of both. What do people think of the differences between the private and the public? Like going to a private clinic versus public clinic, especially those people who have experienced both.**

Akira: I have to say, when I was getting my eyes done, I went to the Diabetes Institute, and there was a fellow there that had to laser my eyes, and it was very uncomfortable. And he’d be going, “Look, my other patients don’t complain, why are you complaining?” You know? And then he’d be talking about his expensive car, hoping that I’d be impressed I suppose. And when I went to the Eye and Ear Hospital, the staff were always lovely. And if they were going to be a bit rough about something, they’d say, “Look, I’m going to be a bit rough, I’ll be a bit careful.” And I’ve found them or caring.

**Q: Okay. What do other people feel, or think, or have experienced?**

Freya: Well I’ve found that in my – when I went public, if under normal circumstances I was very pleased with it, except that it was hard to get appointments. And if I could go public in T City, I would, but I don’t really want to travel all the way to Hospital 1, and I don’t know whether there’s any other alternatives. I’m happy with the doctors that I’ve got, but I mean, it’s pretty expensive, and so from that point of view I’d rather go public.

**Q: In terms of the services that are offered private versus public, are they similar, worse, better?**

Freya: Well I think that the Diabetes Centre in Cairns was more thorough than the private diabetes doctor here. Mostly because I see them – I saw them more often. However, my GP is very involved, and I’m very happy with him. And the renal doctor is also very involved. I would just like to add one thing, which I’d forgotten about, which was connected to Hospital 1, is in a few months ago – actually probably six months ago now, Hospital 1 hosted a meeting in Cairns for renal patients, and I thought it was fantastic. They did a wonderful, I was terrified about dialysis, completely terrified, and they really went through it very well. And afterwards there was a young woman who had been doing dialysis on herself, haemodialysis, for some time, because of a genetic problem, she didn’t have diabetes. And she was smart, up to date, and was managing to work part time. And it really sort of cheered me up, it wasn’t sort of like a death sentence, which is the way I’d actually thought of dialysis.

**Q: Private versus public, anyone else want to make any comments?**

Margaret: The private doctor I see, Professor Komasaroff, he’s gorgeous actually, he’s good. I mean, I can tell he – I mean, he’s thorough. Quite often I’m frightened of going to see him, if I do something wrong, <laughs> I think, oh he’s going to yell at me. But he doesn’t. But he has given me his private phone – he did offer me his private phone number at home, but I’ve got his mobile number. If I have any problems. Because a few times I’ve collapsed, gone into – well it’s not actually a diabetic coma, but it’s similar. (Freya looks surprised, and Akira nods) But I’ve ended up in hospital with it. That’s my frightening thing, but I don’t let things get that far now. And so it was basically to tell him what happened, or things like that. Or if I end up in here.

**Q: And when you compare that to your experiences with the renal services?**

Margaret: I did do that at Maroondah for a little while. I don’t think there was…

**Q: What, the diabetes, or the kidneys?**

Margaret: That was with the diabetes. There wasn’t very much, it’s just a diabetic educator came down and told me that I had diabetes, and that was it.

**Q: So some people like private versus public because they see the same doctor, and some perceive that they might get more holistic care, of more continuity of care. Does anyone identify with that?**

Manny: Yeah. With my experience with private doctors prior to going on disability, I find that the public system is actually better than the private doctors, because there’s more expertise 9Freya nods, is she surprised or she agrees?). If you’re going to go into hospital, if they find out that there is a real problem, then you will call Professor Komasaroff, or professor this, or professor that, to actually look, you know, the experts to have a look at it, whereas the private doctor, he will use whatever, you know? He might not have the advice available there and then to him. That’s what I’ve found.

**Q: Let’s move on. What are the strengths of the services here that you’ve received for your diabetes and chronic kidney disease?**

(All thinking for a moment, Akira looking up and frowning)

Manny: I mean, as far as I’m concerned it’s a reliable service, I haven’t been let down as yet.

Margaret: I haven’t been let down either.

Akira: I have to say that my experience, my personal experience of the public sector has been quite good. However I remember when my son was quite young, he was – he had pneumonia, they kept on trying to dump him into a cold bath, which he was quite frightened off, and he was quite feverish, he had quite a high temperature. And they kept on trying to feed him ice cream, he couldn’t keep anything down at all. So I stayed by his side for 24 hours, the next day he was out of hospital, I couldn’t believe it. So basically people weren’t going, “Well look, what’s the best thing for him? Yeah, he’s panicking, should we ignore the fact that he’s panicking?” So the fact that I was there to make sure that nothing unreasonable was done. So from that point of view, the general staff didn’t know the best thing to do, and he needed individual care. And the individual care got him out in one day. So I’ve observed that. But my personal experience has been quite good.

**Q: So basically everyone’s saying that it seems to be effective, and it’s working for them?**

**(Manny and Marg nods)**

Freya: Well I can’t really make a judgement.

Manny: I’m saying that in terms of also what you hear from other people, like part of my family uses the Hospital 4, and they don’t necessarily say that it’s a bad service, but when they tell me the waiting times, and how long the appointments, and whatever, against here, sometimes I’m flabbergasted at it’s – I go back to saying, never had any complaints to this hospital. (Akira nods)

**Q: And what do you guys find particularly affective about the diabetes and chronic kidney disease health services here?**

(Akira looks up thinking, silence)

Manny: That we’re still here, still alive. <laughs>

Freya: I don’t really know, but I hope I don’t have to find out. I can keep away from the dialysis.

Akira: Now I have to say one of my good experiences with the podiatrist, who had good communications with the other departments, and called them in when necessary, and the seminar that I went to on the kidney stuff, that was quite good. The normal service I find unremarkable, but…

**Q: When you say normal service, what do you mean?**

Akira: Well just my normal appointments, you know? I find adequate, unremarkable. But the extras, like the seminar I went to, that was good.

Margaret: I’ve been into emergency a few times here, and the last time I did, I had one of my little turns, by going into a hypo, bad hypo. And I tell you what, I was out of emergency pretty quick. They just gave me the syrup.

Manny: I went to emergency and I came out 12 days later. I think I was in emergency, I’m not sure.

**Q: Okay, what impact has attending the diabetes and kidney health services here made to your health?**

Margaret: I think they’ve done reasonably well, except for – I’ll be talking to the renal doctors when I see them next, about what food I’m supposed to thing-oh.

Manny: Going to complicate your life now.

Margaret: Yes. That will complicate Weight Watchers, won’t it. And I think that would be about basically it. The diabetes ones, I basically know what to do.

**Q: What does everyone else think about impact?**

Akira: Now I have to say having a history of results has been quite useful, because the HBA1C in particular, because there have been times when my sugar control has been very yoyo, sometimes been high, sometimes low, and to know that my HBA1C is about right means that I’ve sort of balanced it out, but I’ve got to work on doing it more evenly. So that was a useful thing to know, to go, “Okay, look I haven’t completely ruined myself, but not so high, not so low, work on that.” So that was useful.

**Q: For the diabetes services.**

Akira: Yes.

**Q: Anyone else, what impact, positive/negative impact?**

Manny: I just reiterate that like with me where I’m situated, where my doctor is, my GP is, all the services here, and like with the diabetes, you be coming regularly, which I have been for – now it’s been spaced out a little bit longer. It used to be virtually every month, and now probably a month to two or three months. And as long as you keep control of your – at home, and you try and do the right things, and you know? My sugar controls have been sort of reasonably good, with the occasional bump, but over time it’s been quite good.

**Q: Okay, let’s move on. Let’s think about your experiences with living with and looking after your diabetes and your chronic kidney disease. So just bring that into mind, what’s your experience living with and looking after your diabetes and chronic kidney disease. In your opinion, what do you need in place for you to look after your diabetes and chronic kidney disease well?**

Akira: Well certainly I think I should get, what you call it, a session with a kidney educator so that I can factor that into my meal planning. (all nods)

**Q: What does everyone else think? What do you guys need in place for you to look after your diabetes or chronic kidney disease well?**

Manny: Well I have to say that again, maybe – I’m lucky maybe that’s just happened. But with Hospital 1 services, are pretty good, and I have also placed myself with the Prahran Community Health Centre, and they’ve been absolutely terrific in terms of offering different services. I live on my own. In all facets, with the dietician, I have to do some physio work, I had to do – had an operation on my arm because of the diabetes, the carpal and the nerve release, and I have left the service at Hospital 1, they had therapies, and it’s been taken over by the Health Centre.

**Q: So what you’re saying is you seem to be well supported by all the…**

Manny: Yes. I have a network sort of..

**Q: Of support.**

Manny: And a lot of things were initiated by Hospital 1, sort of they put me onto the Centre, and so on. And they released some of their – Like I said, I’ve seen the dietician here, I’ve seen the educator here, now I’m doing it there. Home services, and so as far as in my case, at this stage, I’m in a good space.

**Q: What about everyone else? What does everyone else need to be placed for them to look after their diabetes and chronic kidney disease well?**

Freya: I’m just sort of muddling through, I’m not quite sure, I don’t think I could pinpoint any specific thing I need. It’s just a matter of eating enough to stay alive, without – and trying not to damage my kidneys further. But Manny, you mentioned about your carpal problems, I had problems with that last year, I didn’t realise there was anything that could be connected to diabetes, and I tried to go to a physiotherapist, and was in a splint for six months. I’d been offered diabetes educators, and really beyond that I think I’m perhaps a bit cocky, but I think I know what I should and shouldn’t be doing.

**Q: So some people thought that they needed education, or they needed support of family, or they needed supportive relatives, or supportive hospital, or they needed motivation, needed to be in place to look after their CKD and DM.**

Freya: Well I’m widowed, so I’m on my own. I have family close-ish, but they don’t know a lot. They’re lovely and supportive, but I battle on, on my own, and I can cope.

Akira: Now I think that when I need information it’s more basic information about food, because if I walk into a dieticians, and they say, “What’s your favourite food?”.. Char Keow Teow? What is that? (everyone laughs) And I say, “What basic foods I should avoid, and I can say yeah that is in Char Keow Teow, and that isn’t and so forth.”

**Q: I can identify with you Akira. Okay, Marg, have you anything to add on what you need in place for you look after your diabetes and chronic kidney disease well? Or what do you think is important to be in place for you to look after your diabetes and chronic kidney disease well?**

Margaret: At the moment, I’m retired, I’m a pensioner, and my husband’s my carer. And he does most of the work. I’ve just found out that I’m weak in the arms, have trouble opening a bottle. I’ve got daughters, who are supportive, I’ve got three daughters who are supportive. One of them is diabetic, so wait until she hears about that. <laughs> I’m talking about what she’s not allowed to eat. No, I think I’m pretty right.

**Q: So it sounds like what’s important for you is the support of family.**

Margaret: Supportive family, and if I’m in trouble I can always turn to the GP.

**Q: Or the hospital?**

Margaret: Or Maroondah Hospital, I’ve done that before. Or anything else.

**Q: Does anyone else want to add anything else in terms of things which are important to be in place for them to look after their diabetes and chronic kidney disease well?**

Freya: One thing I did mention before, it would be really nice if some sort of food guidelines were put out for people who have this. Not just kidney disease, but the diabetes. (Akira: Hmmm, nods)

**Q: Anything else people want to mention?**

Akira: Well another complication that I have is that I’ve got optic nerve damage, and I really need to make another appointment and get my eyes checked out to say, well okay do I need to do anything about that, or is it just going to go downhill and I have to live with it?

**Q: Okay, well if no one wants to add anything further, I might move onto the next question.**

Manny: Can we generalise other than renal and diabetes services?

**Q: This question is what do you need in place for you to look after diabetes and chronic kidney disease well? So we’re looking at not just at the services, but globally. What do you need in place? Have you got anything further to add?**

Manny: It’s virtually going back to the same point of communication between different services. Like with the ophthalmology, I’ve been a patient here, but I was sent to the Eye and Ear for – I had bleeding in the eye, so they had to do it there, they couldn’t do it here. (Akira nods) Now that was back in November last year, and I’ve been back there for consultations, to just see how I’m going. My last visit there was on the 1^st^ of March, and on the day that I left the hospital they had the paperwork ready, and they said they were sending it to Hospital 1 so I can return here. Now it’s been March, April, May, June…

**Q: And they still haven’t sent it to Hospital 1?**

Manny: I just a letter last week to tell me that they’re going to try and get me an appointment here, but in the meantime I should go back to the Eye and Ear if I have a problem. And then yesterday I received an appointment for the 15^th^ of July. So where I was coming on a monthly basis to get laser treatment, and a needle in the eye, after I had the bleeding operation, and I’ve recovered enough for them to send me here, I virtually haven’t had any treatment for four months. (Freya looks sympathetic, Akira nodding) Well I’m not going to have any treatment until mid July. And my eyesight is, I’ve got limited eyesight in the right eye, and this my so-called good eye, which is – it’s a grave concern. Your sight it always – you know? I would put that ahead of my kidney (Akira nods). But that’s my case. And that goes back again to what happened to the letter that the person was actually holding in front of me then (Akira nods), when he came out he passed it onto the lady at the reception and said, “Do it now,” I heard him. And it got lost. Like he was saying about manual sort of stuff, it got lost.

**Q: Okay. A similar sort of question, but on the flip side. What things make it hard for you to look after your diabetes and chronic kidney disease well?**

Freya: Finding the right sorts of things to eat.

Manny: I’d have to agree with that, that seem to be the main issue with me.

Margaret: I agree. <laughs>

Akira: Now I found that when I was diagnosed with the diabetes, it was less of an issue than it is for a lot of people, because I’d started to change my habits to the better. Now if I knew more about what I need to avoid for my kidneys, I could start working on a change. Because I find that looking for things that I like is a better motivator than going, “No, can’t have that, can’t have that. One gram of that, two grams of that.” That doesn’t work for me. But if I say, “I love this. Yeah, well the fact that I’m missing out on that doesn’t matter because I love this.” So if I know certain things that I can have as much as I want of, or I can take a moderate amount of, as opposed to completely avoid, then I can make a plan. (Freya constanly nodding, Marg nods)

Manny: Just a comment, someone could make a killing in devising a book of that. Did you have the same reaction from different groups on that issue?

**Q: No. Actually for some reason this group, we’ve focused a lot on diet. Education was something, but this was very focused on diet. Any thing else, barriers or things that make it hard for you to look after your diabetes and chronic kidney disease?**

Freya: Well before we got onto that one, my sister-in-law and I, when we found out I had to worry about the type of food I ate, we really did investigate, and we came to the conclusion that caterers who wanted to actually specialise in specific diets, you know, Light & Easy, and kidney and/or diabetic or something, could make a killing and be franchised all over Australia, and the world, if they concentrated on that. Because in the beginning it is so hard, you just really have no knowledge.(Akira nodding, Manny had head in hands during this part) And I’m sorry, I forgot what you were saying there.

**Q: So what other things make it hard for you to look after your diabetes and chronic kidney disease well?**

Margaret: Doctor Komasaraff threatened me with a lap band, actually put in a referral for it, because I was well over weight. But I tricked him (Akira laughs), so did lose the weight on me own. Because I didn’t want the lap band. I went for most of the things, but I ended up telling them that I wasn’t doing it, because I was losing the weight anyway. At that stage I was doing Zumba, and you can do Zumba, and it doesn’t matter who you are. Then I started doing Weight Watchers.

Freya: Can I just mention about the lap band. I had a lap band put in ten years ago, because my husband at that time had been feeding me up, like I’d gone from a normal weight into fat. And when I got the diabetes finally decided that something – and he actually worked out that he was killing me. And I went and had a lap band, I didn’t have any medical insurance so it cost about $12,000. (Akira and Marg nods) And it didn’t work. I ate, I’ve not eaten anything – I’ve been coping the sandwiches today, but I didn’t have anything other than breakfast, which is my excuse. But I didn’t eat any plates larger than a bread and butter plate, and I still don’t, and I lost five kilos in all those years. And I recently went to a lap band doctor in T City, because I’d been in Cairns, and he used to adjust it for free. And I’d got the way that I couldn’t eat anything because I was regurgitating everything. (Akira nods) So he loosened it and explained to me that he’d actually stopped – in the majority of performing lap bands, because they didn’t work. (Akiraand Marg nods) He now does sleeves or gastric bypasses. But I got very tired of the people who knew sort of saying, “What’s wrong with her? Is she stuffing lollies down or something?” And I hadn’t been. I’d been to so many nutritionists I was just about a member of the nutritionist association. So I think you were wise avoiding the lap band, you saved yourself a lot of money for perhaps dubious results.

Margaret: Yeah, well I’ve lost over 15 kilos.

**Q: Well done. Sorry to interrupt guys.**

Margaret: That’s still eating bananas too.

**Q: Just to get back to the question, so what things make it hard for you to look after your diabetes and chronic kidney disease well? So some people talk about having a lot of other illnesses, or having a lowered mood, or lack of support, or lack of education.**

Margaret: Mine is basically possibly medication I’m on for, not necessarily for diabetes, for the transplant which I had.

**Q: As in they make your diabetes worse?**

Margaret: Yeah, it went up sky high afterwards.

**Q: Yep. Any other things, or barriers?**

Akira: Now I find that because I’ve got numerous complications, and some of which I have to take multiple medications for, it can get a little bit difficult keeping track of medications. Like let me see now, how many tablets do I have to take in the morning? About three tablets in the morning, and about three tablets at night. Some for blood pressure, let me think. Now at least one for kidneys. And let me see now, so there are tablets that I have to take once a day, there are tablets that I have to take twice a day, which is not too bad except sometimes I forget whether I’ve taken a tablet or not. Then there’s another one that was recommended as an optional thing, which is every two days. But not every two days, it’s three a week. So I can’t go, oh it’s the second day, I’ll take one, because I go, no that would mean I would have to take four in a week. So no, don’t take that one. And someone said, “Don’t keep taking that, because that’s bad for you.” Did I take it, or didn’t I?

Manny: You can get a pre-pack.

Akira: Well no, that’s not a problem, because I drink a lot of fluids, and I eat a lot of, what do you call it? Fibrous foods, so that’s not generally the problem.

**Q: I think what Manny was saying is you can get a pre-pack, right?**

Akira: Pre-packed.

Manny: Sorry, so you know what you’re taking at all times.

Akira: Yeah, and some of them I go, look I don’t know whether I took one or not. If I don’t take one it’s not going to be a huge issue, because I just take it the next time, I just take it. But nevertheless it can get a bit confusing. So that is a problem sometimes. (Freya nods)

**Q: Any other factors, or problems, or barriers, that make it hard for you look after your diabetes and chronic kidney disease?**

Manny: Yeah. Just probably say that probably agreeing with what Akira maybe said, where the fact that you have so many issues going. Like it’s very difficult to get a balance from one to the other. I mean, I mention that only recently I’ve been getting fluid in my legs, and my doctor attributed my weight problem went up 20 kilos. Now my doctor was attributing five, six kilos of that weight to the fluid, but in the meantime I was, as far as I was concerned, I was eating the right things. So why did it go up 14 kilos, and why can’t I get it rid of that weight now?

Freya: Are you on insulin?

Manny: Yes.

Freya: Because I’m on insulin too, and one doctor said to me that it creates – it’s inclined to put weight on.

Manny: But I’ve been on insulin for like two years, and this has only happened in possibly the last six months.

Akira: I just want to mention that Freya also mentioned it, but the contradiction. Because I remember when I went to the session on kidney disease, they’re going, “Well look if you’re on dialysis, you have to reduce your liquid intake.” But as a diabetic I’m drinking all the time, so drinking is good. Now they say for kidney disease, at least if you’re on dialysis. I’m not sure whether it is an issue whether I’m not on dialysis. But I’m thinking, yeah look, I feel thirsty quite often, and certain things I say, “Yeah that’s sugary, don’t drink that.” Other times, what’s wrong with drinking a lot of water? So I don’t know whether I have to consider that as well. (Freya constantly nodding)

Freya: Akira, actually I suddenly, after being in Cairns and drinking an awful lot of water, because you sweat so much and stuff, I came down and I found that I wasn’t drinking much. This is after the kidney business. And I was worried about it and mentioned it to my doctor, because sometimes I could go for half a day and I wouldn’t have had a drink. And he said, “That’s fine with your kidneys. Fine.”

**Q: Any other barriers or things that make it hard?**

Manny: Just on that point, when I was in hospital only recently, I was on 1.5 litre water.

**Q: Fluid restriction.**

Manny: Fluid restriction, yeah. And again, one of the specialists came in and he actually queried it, why I was on fluid restriction. I was left on it, but I never actually got a clarification on what the problem was in terms of – as to why I should be on that.

**Q: Anyone else want to add anything else?**

Margaret: I’m on fluid restrictions, and I’ve been on it for years. (Manny nods) I’m only allowed to have 1.5 litres. Because of the swelling in my legs, because I’ve got that too, the oedema.

Manny: Well there you go.

Freya: Nobody’s told me that I should be limiting my fluid.

**Q: I think it varies depending – I think it depends on your, and your height, and stuff like that. So just moving away from the fluid, has anyone else got anything else they want to mention in terms of things which make it hard for them to look after their diabetes and chronic kidney disease?**

Akira: Yeah, look one of the things which is not central to this, but just the deterioration of vision. Let’s see, I was told that – I can’t remember the type of dialysis. Well if you haven’t got good vision, this isn’t for you.

**Q: Haemodialysis.**

Akira: Anyway, the thing is when I take insulin, if I’ve forgotten to take my magnifier with me, I can’t see how much I’ve dialled up, so that can be a problem.

Manny: What was the name of the dialysis?

**Q: Haemodialysis. Anything else people want to add before we move on? This question’s a bit more fun. Now lets move back to the health services provided for the management of your diabetes and chronic kidney disease. All of you made some comments about strengths, and weaknesses, and things which you find affective and frustrating. Imagine if you became the director of Hospital 1, and you had the power to change things about the diabetes and the chronic kidney disease services, if we were to design a perfect health service which would meet all your needs, what would that include, and what would it look like?**

Freya: Have branches outside the city.

**Q: So you’re talking about localised care?**

Freya: I’m talking about say have like a campus of Hospital 1 in say places like Braeside.

**Q: So basically you would like the service localised to your community where you are?**

Freya: Yes.

Akira: Now I see this sometimes at doctors and you know, GPs, and I see this sometimes in chemists, you’re sitting there minding your own business and there’ll be little leaflets saying “Have you got this condition? Maybe you should look at this.” How something like that would be a good idea, as well as actual education services, so you can say, “Oh, I’ve got chronic kidney disease, oh I should be looking after this. Maybe I should make an appointment with a dietician.” That sort of thing. (Freya nods.. mm agrees)

**Q: So leaflets in the waiting room, is it?**

Akira: Yes.

**Q: Yep, education leaflets. Anything else people would want to have or include?**

Manny: This is more probably a question, so I can maybe ask another question or put a comment. What’s the percentage of diabetes/renal complications, as in combined? By that, I’m saying how many people actually have diabetes that leads to…

**Q: Diabetes is now the most common cause of chronic kidney disease in Australia.**

Manny: Okay. So they’re related?

**Q: Yes. Why do you ask?**

Manny: I was asking in terms maybe there should be – don’t ask me, I’m not – don’t know the medical part of it, but there is a pretty – there should be more maybe closer relation in terms of appointments, and maybe more interaction between the two departments. Like they should be maybe made into one (Akira nods… Hmmm), and sort of that way you can – everybody’s on the same page. (Akira nods)

____________________

**Q: Yeah.**

Manny: I don’t know, I mean, that – you’re telling me I’m the director of Hospital 1, so I made a decision. So yes, I would say that.

Freya: This isn’t about that, but what is the proportion of diabetics who develop kidney problems?

**Q: How about we just address the focus group, and then we can talk about other medical issues after. Anyone else got other suggestions? So why would you propose that? How would that make it easier, or what benefits would that have?**

Manny: Well I believe that – I mean, I would even go as far as, don’t know how it would work, but having a consultation with maybe not at all times, but at a certain point, when you’ve done your first, second, third consultation with your diabetes, your renal, whatever, you can have the renal expert, the diabetes expert in the same room with you. So I know what the problem is in my body, you guys have told me what my problem is, or you have and you have, now do you agree with you’re in the same – you know? (Akira: Hmmm, nods) You’re not necessarily going to fight in there, if you don’t, but like you can get an idea of where you’re at. Do you agree? Is there something that you missed? Is there something that the other gentleman missed? (Akira nodding) So like I said, not necessarily every visit, but maybe on an annual basis, or whatever the – you know? The organisation decides, you have a combined consultation. (Akira nods)

**Q: What do other people think?**

Akira: Now I’ve had one of the medications I was put on was [Avapro] *01:31;10, and when I started seeing the kidney doctor, the kidney doctor goes, “Yeah, I don’t think Avapro’s a good idea.” Next time I saw the diabetes one, they said, “Well what medications are you on,” and I told them, “The kidney doctor took me off Avapro.” They didn’t question it, or go okay, so I’m thinking, does he not know what this stuff it? What is the deal here.

**Q: What do other people think? Do other people agree with Manny, or does anyone want to add anything, or propose anything else?**

(Silence)

Manny: I’d like to quit as a director. <laughter>

**Q: Marg or Freya?**

Freya: No, not me.

**Q: If you were helping out Manny as director, what else would you do?**

Freya: Make more parking available. <laughs>

**Q: Anyone else? Marg? This is your chance guys.**

(Margaret thinking)

Margaret: Is this going to the directors?

**Q: The final report may, but this is just in what ways can it be improved, what would you want to see?**

Freya: From a position of almost complete ignorance since I’ve only visited here twice, and I had one meeting in Hospital 7, I think it seems to be fairly good. However the second visit that I had, the doctor didn’t have a clue why I was in there, and if I hadn’t argued about the necessity for having an operation for a dialysis shunt pretty much immediately, and he actually looked up the information and discovered that I was telling the truth, I wasn’t that impressed with that. I thought if I’d been some meek little thing which was in awe of the medical system, I would have been well and truly on dialysis by now, and possibly not need it. (Akira and Marg nods)

**Q: Anyone else, do you want to add anything, change anything?**

Manny: In terms of the consultation, I have been to consultations and I had to, the person would be looking at the screen, looking I’m assuming that the blood test, or something like that, and he will write his notes, and I don’t know if that’s the experience of other people, but like there’s no actual physical check. I’m not sure renal, kidney’s you can actually check, if you can actually- (Akira frowning and listening) but I mean, last time they check my blood pressure, I don’t know if there’s a relation with that to the kidney function, but is there meant to be like a more hands on approach from the doctor rather than coming in, sitting down, looking at his screen? “Oh yes, you are…” I’m not even seeing the screen.

**Q: What do you mean hands on? I mean, they should examine you at least once a year, and take your blood pressure, and look at your feet, and listen to your chest, and stuff like that. They are doing that, aren’t they? (Marg nods)**

Manny: Not unless I point it out. It’s more or less a sit down session, and like I said, they will look at the results.

**Q: Is this for kidney or diabetes?**

Manny: Kidney.

**Q: Yeah look, I don’t know, I can’t comment.**

Manny: Yeah, I’m just saying. Like occasionally they will do the old – you know, the checking your – the knocking on the chest, listen to your chest. I mean, I’ve had a kidney – a tumour removed, I don’t know what – whether it’s a requirement or not, but nobody has looked at it. I mean, I don’t know if it tells you anything, I’m assuming you’re probably more concentrated on what the blood test, whatever says. But at least, if it’s a kidney, “Oh yeah, how you’re feet going?” You know, that question doesn’t come up. “Oh doctor, my feet are really hurting me?” “Oh okay, let’s have a look at them.” If it’s a problem and it came up on the last visit, then surely there should be a line there saying whoever the next person to check you, please check feet, (Freya nodding) please check fluid retention, please check swelling.

**Q: So it’s not being communicated from appointment to appointment?**

Manny: Well obviously not. But I’m also going back to the fact that the actual consultation is more of a computer session. You know, the doctor concentrates on what’s in front of him. And I’m worried because, like I said before, some of that information is not up to date anyway. (Akira: Mmm, nods)

Akira: Yes, it’s that you’re not a person, you’re just a bunch of symptoms.

Manny: No, no. I got no complaints in terms of – like I said, the service as such. It’s just the consultation needs to be more inclusive, as in if nothing is showing on your screen, and you show on your screen like December 2012, hang on a minute, that’s four or five months ago. “Have you been here in the meantime?” “Yes doctor, I have.”

Akira: So an overall thing.

Manny: Yes, and overall thing. But also there should be more of a…

**Q: What do you mean by no overall things?**

Akira: Well he’s just looking at this number and that number, and not taking a big picture approach. Is that what you mean?

Manny: Yes. To combine the information on the – what’s coming up on your screen, which is the…

Akira: The basic data.

Manny: The experimental part of it, like what your blood came up, or whatever it came up, your sugars and that, and to combine it with an actual physical as much as you can go. Because like I said, I don’t know how much physical you can do with a kidney. But I mean, the old days, I’m old enough to remember that people used to feel, and tell you, “Oh you’ve got a problem.”

**Q: Yeah. I mean, the kidneys are more been following a blood test, having said that. But what about the things that Akira brought up, like do you feel that – do people feel that they’re just a number, or do they actually feel they’re getting a personal service?**

Akira: Now I find it depends on who I get seen by. Because I problem that specialists ten to have, like I went to see a dermatologist, and the dermatologist said, “Yep, yep. Know what that is, yep. Take this.” And I’m going, “Yeah, what’s the cause? When should I stop taking this?” And they just sort of wrote the prescription and I’m out of there, and I’m going, “What was that all about?” You know? So there’s – I’ve got a whole bunch of creams that I’m supposed to take for a skin condition, and I go, “Well look I’ve found that when I get itchy if I take that one, that’s good. If I’m not itchy I don’t take it.” And that seems to work all right. Or another doctor, I go in there, and they go, “Yeah, you can take this. Take my word for it, just take it at night.” “Why?” “Don’t know.” You’re just a dumb civilian, you wouldn’t understand my explanation, so just do as I say. You get a bit of that.

Margaret: That sounds like a lack of communication or a lack of interest.

Akira: It’s something that specialists tend to do. They go, “Look, I know this stuff, and if I try and explain it you really wouldn’t understand it.” I’ve done a year of biochemistry, I do understand a little bit more than average.

Manny: I just thought of something. When you go for your appointment, and I understand it’s a public hospital, and you’re moving – we’re not numbers, but still that’s the way it is, you’ve got to keep moving. And you had, for example, I had Professor Vue last time. Now if Professor Vue happens to be as a consultant on the day again, because I’m assuming it’s rotating, it’s not the same people all the time. If he was there on the day, why not put my appointment card after I’ve been booked into Professor Vue’s tray?

**Q: So you’re seeing the same person? (Akira nods)**

Manny: Just to have a little bit more continuity. Now it might not work, because he might not be there on the day, but where it was possible, and you can sort of see him again, unless of course you end up with 20 appointments in his tray and one on the next one, there’s got to be issues with that. But it needs a bit of work. But why not also try to have some continuity? Because what I’ve found with my diabetes, whenever I get – should I mention the name? There’s a Doctor Arnott that now I’ll call it luck or design, over a three year period let’s say I might have had, I’m guessing 100 appointments, I might have seen him for 40. And he sort of remembers me, and he knows my – because he treat me initially, so he’s aware. Now it doesn’t mean that I’m going to get him forever, but if he’s available. You were saying before, Professor Komasaraff, if he’s available, (Points to Marg) put Marg’s name in his tray. You know? Just a thought.

**Q: Yep. Now Akira’s also mention something – I think you were mentioning that they were treating an organ rather than the whole person. (Manny nodding in agreement)**

Akira: Yes.

**Q: Other people have actually complained about that as well, that when they go to diabetes, they just worry about the diabetes, and they won’t talk through anywhere else, they just say, “Go somewhere else.” Or the kidneys. Do people find that, and do people find that frustrating?**

Akira: Well…

**Q: So you’re nodding, are you Manny?**

Manny: Oh yes. I will wait for Akira to finish.

Akira: Yeah I had an instance where I went and said, “Look, I’ve got this thing with my skin,” and they said, “Oh yes, you need to see a vascular surgeon.” So they wrote me a reference to a vascular surgeon. I went to see a vascular surgeon, he said, “No, skin problem.” So I had to go see someone about my skin problem. <laughs>

Manny: Stand back everybody. <laughter> Actually when you brought it up, that’s a huge issue with me, very – I get – If I do get angry with anything, even though I said it’s a pretty good service, that will be it. Because of the fact that I have so many issues, and the swelling feet have nothing to do with me, you have to go see a podiatrist. The fluid, it’s nothing to do with me, - I’m talking about the podiatrist, because it might be a renal or other function, you know? “But I’ve got a high sugar levels,” or whatever, “Yes, make an appointment to see whoever the department is.” And so on, and so on.

**Q: So do you feel that anyone’s pulling it all together and knows what’s going on?**

Manny: No. And this is where I was saying that parts or maybe some sort of solutions, is occasionally during the year to have a consultation as a – you know?

**Q: Does anyone else identify with what Manny’s saying?**

Freya: I don’t really, because my GP does that. Do you have a GP?

Manny: Yes.

Freya: And he doesn’t coordinate it?

Manny: That’s when I mention initially that nobody gets – My GP looks at his screen, and the last information he’s got from Hospital 1, it was December. So it’s nothing to do with me, because I was in hospital a month ago.

Akira: I’d just like to comment, that let’s see – what goes against is certain individuals, like I had a podiatrist who was very good at coordinating stuff. She’s say, “Well I’m dealing with your feet, but you have another problem, you need to see someone about blah.” And she would tell me to make an appointment. Okay, I’ll go make the appointment then, you know? So that was an individual. So she was giving more than the system was asking her for. And I think any individual can do that. I mean, if you go an see and endocrinologist, and the endocrinologist says, “Yeah, you need to see a vascular surgeon,” that’s that individual, it’s not the system that’s doing that.

Manny: But then you need consensus that this person doesn’t just do it for you, that he does the same service for you, you, you. So there is other underlying issues there. (Akira: mmm nods)

Akira: Yeah, it’s a little something extra that they don’t get paid for, but certain individuals will do that.

**Q: Marg, do you have any comments?**

Margaret: My GP had put me on [Sign Me Up] *01:47:17 for podiatry, when I booked the podiatrist. And it was through Medicare, and it was five visits, but I can get – I asked the doctor whether they can be a repeat, or I can go do it privately.

**Q: So I guess do you agree with what Manny’s saying?**

Margaret: Some of the things, yeah.

**Q: What about his last point about a lack of holistic care, and just care being segmented all the time?**

Margaret: With me I still get me blood pressure taken, I still get me chest – breathing done, the lungs. They check my legs because of the oedema.

**Q: Who’s they?**

Margaret: This is in renal. Renal they check that. In the diabetics I get me blood pressure done, I get me chest – you know, chest thing done, and whatever else I’ve got.

**Q: So you don’t feel like there’s a lack of holistic care?**

Margaret: No, I ask questions.

Freya: Well because I probably talk too much, I let my GP, who is wonderful, know everything. And actually I talk their legs off, all of them, so they all know they can communicate. But getting back onto Hospital 1, my renal doctor didn’t get any response from – No. He didn’t acknowledge that he’d had any contact with Hospital 1 after he’d made the initial appointment, and that was fine. But he was – came out of the blue, when Hospital 1 asked me to go back. He didn’t know what, and I told him when I saw him, and I said, “What happens if Hospital 1 contacts me again?” and he said, “Ignore them, and contact me.” But as I said, the doctors do talk to one another, so it’s – and I’m seeing a podiatrist next Friday, but she works in with them as well. So it’s just a matter of me being pushy, and the GP being lovely.

**Q: So if we’re designing the perfect health system, has anyone got any other suggestions, or we’ll move on.**

Freya: One thing just as a general comment. I’m really impressed with the – and I know it sounds like I’m crawling, but I’m really impressed with the medical services in Victoria, and Queensland actually, in Cairns. But I keep on thinking how lucky we are in Australia to have competent and capable, and such a wide variety of medical specialists that we can see. I mean we’d be paying hundreds of thousands of dollars if we were in the States. (Akira nodding)

**Q: Okay, well has anyone else got anything more they want to add in terms of designing the perfect health service, before we move on? Okay, now finally, is there anything else that we’ve missed out, or that you came wanting to say, that you haven’t said?**

Margaret: No.

Freya: I’ve said probably far too much. <laughs>

**Q: So has everyone said everything they came wanting to say, no one else wants to add anything further to the discussion?**

Akira: I guess, how can I put it? Well for instance, I’ve had experience with the Childbirth Education Association, and what that was is a bunch of people that had an interest in home births, and stuff like that, so they had a network of people who were interested, had experience. And maybe if hospitals were to have some sort of community branch where they’d perhaps visit once a month, or something like that, and people can go and discuss it, and they can say, “Well who do you think’s a good doctor?” “Well they’re good because so and so.” And the doctors come in there say once a month and have their say, and say, “Look any problems we should be addressing?” That kind of thing. So more community integration might possibly be an idea.

**Q: So explain that further?**

Akira: Well it’s a bit like the bare foot doctor concept, although I’m thinking more of a – how can I put it? More integration with community health centres, or something like that, so that you could send people out occasionally, but mostly they’d have strong communication with a hospital. (Freya nods)

**Q: And so people can give feedback, and it’s like a community group sort of?**

Akira: Yeah.

Manny: That’s what you were asking for before, isn’t it?

Freya: Yeah, sort of. I think that’s a wonderful idea, and a bit like the Legal Aid Service.

**Q: Can you just flesh that out, I’m trying to picture what you’re saying.**

Freya: Perhaps like neighbourhood centres or something, where a paid doctor comes out representing whatever, sort of to generally be available for questions, and problems that people have.

**Q: So in terms of feedback?**

Akira: Yes.

Manny: I think I can put it in more clear terms, as far as I’m concerned. You want to go to a – and that’s not putting down the system. Do you want to go more to a third world system, where like the doctor visits the – you know, the village once a month? Except we’re talking about a modern society, but in the same principle where just breaking into communities and servicing those communities. So instead of Freya coming to Hospital 1, she can be serviced at Hospital 7.

**Q: Is that what you’re talking about?**

Akira: Well my idea is just something to keep the specialists in touch with just the ordinary people, and not necessarily coming out for a consultation (Freya nodding), but just getting feedback and saying, “Well okay, the people are…”

**Q: Just talking to them and providing education?**

Akira: Yeah. Or saying, “Look, I’m busy, but if you want to do this, you can go to this department. Or if you have a worry about this, I can set you up with the educator,” or something like that. Not necessarily consulting actually as a doctor, but just coordinating, that sort of thing. (Manny nods)

Freya: Probably a nurse would do. (Akira nods) Nurses are wonderfully knowledgeable. And maybe a team of them could – there must be lots of people in the community who have got medical concerns, and possibly are wondering if they either can’t afford…

**Q: So just an open thing where people can just ask questions?**

Akira: Mmm.

Freya: And get advice. Sort of like a coordinator that can send them to different places, or say “No look, you’re worrying unnecessarily, there’s really no problems.” (Akira nods)

Manny: Isn’t that the system that the government is moving towards anyway? In terms of make it into a more or less using nurses a lot more, and using doctors on a more spread basis than centralised? The way I understand it.

Freya: I think they were talking about that a few years ago, I haven’t heard anything about it lately. They’re talking about some super centre, at least in Queensland, where instead of concentrating on either hospitals or doctors, they’d have sort of like a halfway one between hospitals and doctors that people could go to. (Akira nods, HMMM)

**Q: Has anyone else got anything further they want to add before we close up? Anything burning that people want to say?**

Margaret: I was just going to say that isn’t there something like that now with nurse on duty, or – it’s a phone call that you can make?

Freya: Yes.

**Q: Yeah, there is a phone call that people can ring up at night, or something. Yeah, with the nurses, I think.**

Freya: Yeah, I think you’re right.

**Q: Yeah, you’re right. Nurse on Call, or something like that. There is a service. Anyone else got anything else they want to add before we close off? No? Okay, good oh.**

END OF TRANSCRIPT

**Focus Group 6**

Theo: I've had approximately 10 years I’ve had kidney problems, not bad, but I’ve been under the doctors for about 10 years. And I’ve had diabetes probably six or seven years, but again I was on three tablets a day and I lost a lot of weight and they’ve taken me off the diabetes tablets.

**Q: Billy?**

Billy: Firstly I not <inaudible> in English anyway, I am from Korea and I find out I have diabetes since about 20 years. At that time I didn’t know, because I had four sisters and brothers and I second and my mother diabetes history, but some other brother and sister that’s ok no more, but I think especially only I have got the diabetes, because I am here single, single long time. And then one day about 20 years ago I working in the night time work in the cleaning and when I driving and some day driving and coming back home I stop in the traffic light, I stop and that time I sleep, too tired. Many times repeat that and so I don’t know why like this, it’s maybe just I am tired too much. I think, but then I stopped that job and I try to be drive instructor, I tried the test and I passed and knowledge pass and until they say they give him the form, check health test. I see the doctor, doctor say oh you have diabetes at that time and the doctor give me first time Diamicron only half per day and next day only half tablet I took, no thirsty no much, before too much drink the water, but stop it. Very good, then I control the medicine something like that, many times I see the doctor and from last year I find it my kidney also the function not good from this hospital. And last May and so after then routine I meet in the <inaudible>, now already improved.

Margaret: I’ve been a diabetic for about 25 years and I’ve had kidney problem for probably 10 to 12 years, that was caused mainly through an infection when I had to go onto antibiotics. I got a prosthesis in my right shoulder and I got a very bad infection in it and I had to have a couple of operations to sort it out and I’ve been on antibiotics for many years and that caused kidney problems.

Tom: My name is Tom Mervan, yeah I’ve been a diabetic for 25 years and I’ve only just recently found out that I have kidney disease in the last 12 months.

**Q: And you're off all your medication now aren’t you?**

Tom: Yeah I’m off the diabetic medications.

**Q: Pretty remarkable for 25 years.**

Tom: Yeah so it’s diet controlled.

**Q: Now let’s move on to the first question, so obviously many of you guys have lived with diabetes for quite a long time and maybe chronic kidney disease for less long, but I’d like to think about your experiences that you’ve had with the diabetes and chronic kidney disease health services that you’ve experienced. Based on your experience what would you say are some of the weaknesses of the diabetes and chronic kidney disease health services that you've received here at Condord Hospital?**

Margaret: Well I haven’t been here other than this focus group, this group I’m with with Jenny and I’ve only been on it since June.

**Q: So what's the group that you're…?**

Margaret: I take a tablet, it’s to do with chronic kidney disease, what they're trying to do is remove phosphorous I think it is out of the blood and that’s the program I’m on.

**Q: But you see Dr. J here don’t you?**

Margaret: I saw him initially when I first came, but I don’t see him, don’t actually see him, I haven’t seen him since other than the initial day.

**Q: So is your chronic kidney disease looked after here though at Hospital 2 or who looks after your chronic kidney disease?**

Margaret: Dr. A, well he is here. Sorry I see him at his rooms at Canterbury, not at Canterbury Hospital, but he’s got rooms there opposite Canterbury Hospital and I see him there.

**Q: What about your diabetes?**

Margaret: Diabetes I see someone at Canterbury Hospital, I used to go to Miranda but I've been going to Canterbury Hospital this year.

**Q: And what has your experiences been like at Canterbury Hospital, any weaknesses the way the services could be improved there?**

Margaret: No, no I think everything’s been; as I say I’ve only been going to Canterbury for a little while. Dr. A is very good and he asked me to go on this other program. No I can’t complain about the services that I had. I’ve got other specialists that I go to as well for other things and the health services look after me fairly well.

**Q: What about everyone else, I presume most of you guys are looked after here for your diabetes and chronic kidney disease or at least one of them?**

Tom: Yes they just took me through a dialysis unit two months ago. They took me through the dialysis unit, nurse Glen Stuart, so they took me through there and showed me what could be happening. And I see Dr. J, he’s my specialist.

**Q: So how has your experience been with the health services, any weaknesses or things that could be improved do you think?**

Tom: No I find it pretty good.

**Q: And the diabetes side is just with your GP is it?**

Tom: Yeah GP.

Theo: Look yeah I was in hospital here for two months last year and I’ve been a patient of Dr. G for many years, I couldn’t fault him, he’s an exceptional doctor. My diabetes is done with the GP, when I was actually here and Dr. G wasn’t there, he did actually go away for a little while over to America and he had a lady doctor and she was quite good. And when I was actually in for two months here Dr. A saw me quite often and the diabetes guy, can’t remember his name, he saw me there. But as far as the treatment of this hospital and as I say I come here still every Thursday for the high risk foot clinic, every Thursday and I’ve been doing that for about seven years. And look it’s faultless, the medical treatment that I’ve been… The only problem that I’ve ever had with this hospital, when I was in intensive care for two weeks and five days and when I come out of that you're always very sick when you come out of that, and I just got treated pretty ordinarily by some of the nurses here. But as far as the medical treatment that was spot on.

**Q: Billy, any weaknesses from the services that you’ve received here for your diabetes and chronic kidney disease?**

Billy: About two years ago I found my *0:09:43 a little low and now from that time I continue and * a little bit improved now, before I never * but from last year I got * like that. And now there’s * alright a little bit, but different from last two years ago, two years very good, but now is really not that good. And also blood pressure also, before normal usually, but from two years ago something not normal, sometimes a little high, sometimes low like that. Today check two times, three times, but today alright they say. And also every diabetes the same cases day by day get a little tired and then a little lose my way and I check * weakness my muscles also skinny like that. But still nowadays better than a couple of months and also good medication almost six kind, maybe seven kind, routine it take…

**Q: But how do you find the health care offered for your diabetes and chronic kidney disease from Hospital 2, any weaknesses or things that could be improved?**

Billy: Yeah here improve.

**Q: Are there any factors that could be improved or any weaknesses to the health care that you’ve noticed or things that aren’t as good?**

Billy: I think usually from two years ago continue come to here to see the doctor and day by day improve.

**Q: Ok as in your kidney function improved yep, but in terms of the services offered, like the health care offered by the hospital, are there any aspects which aren’t as good, not so good?**

Billy: Yeah very good for me.

**Q: Now is it easy for you to get the health care that you need for your diabetes and chronic kidney disease? So do you find it easy to get the health care that you need for your diabetes and chronic kidney disease in terms of locality of the service, affordability and also waiting times?**

Theo: Well I go to Dr. G here in the clinic and it doesn’t cost me anything, I’m a pensioner so everything is fine, you’ve got to travel to get here, but I was offered when I move to go to the Hospital 3, but I chose to come the extra distance to come here because…

**Q: Where did you move to?**

Theo: I moved from Strathfield to Dee Why and I still come here, because it’s always been so good for me.

**Q: And a bit of a travel though isn’t it?**

Theo: It is a bit of travel, but I’d rather do the bit of travel to know that I’m getting; as I say I’ve been under Dr. G for years, I’ve been coming to the high risk foot risk clinic here for about seven years and I’d rather do that. And I’d also done what Tom said about that dialysis thing, the lady that puts you through that, because he thought about five years ago I’d have to go onto dialysis. And touch wood it’s never got any, the condition hasn’t got any worse.

**Q: How about the waiting times to get the service? Is there an issue with that? In some places people complain about waiting one, two hours, things like that.**

Theo: Well look I mean I go every Thursday at 10 o’clock to the foot clinic and I go straight in and Dr. G I’m always with him at half past eight or quarter to nine, so I’m either his first or second patient in the morning. I did have a problem here with a shoulder guy once and I forget his name, he was an orthopedic surgeon and I was booked in for 12 o’clock and I waited for 2:30 and he still hadn’t fronted, so I just left. That he could have been pulled away for something urgent, no one sort of knew what was happening or anything with him. But other than that I haven’t had a great problem.

**Q: What about other people’s experiences?**

Margaret: No I haven’t had any; I haven’t been coming here other than to see Jenny next door for as I say from June. I don’t have any problems…

**Q: So is that for a particular study is it with Jenny?**

Margaret: Yeah it’s about the phosphorous, that study, you can get the information, Jenny can tell you what the actual, but it is chronic kidney disease. I have no problem with Dr. A, I go every three or four months to his rooms in Canterbury.

**Q: And the cost is ok?**

Margaret: It usually costs me $10 above the Medicare, I don't pay at Canterbury Hospital, I didn’t pay when I went to the other endocrinologist that I had, Miranda, I didn’t pay there. I’ve got other specialists and other than the orthopedic surgeon, the others don’t charge me. In fact tomorrow I’ve got to go and see a heart specialist at Burwood, he hasn’t charged. Whether it’s because I’m a diabetic as well or not I don’t know, but I’ve had quite a few tests, including one here. And but I’ve been quite happy with the treatment I’ve been getting.

Tom: Yeah I’m happy, I see Dr. Singh in the chronic kidney disease and he’s quite an excellent doctor. I also see a Dr. R here in I think it’s immunology and he’s an excellent doctor.

**Q: So in terms of health care for the chronic kidney disease it’s accessible, locality is ok, cost is ok?**

Tom: Yeah.

**Q: Waiting times are ok?**

Tom: Yes.

Margaret: Hm.

**Q: Not waiting too long?**

Tom: No once I had to wait an hour and a half, but when that happens, it was only once.

**Q: And how about yourself Billy, how easy is it to get your diabetes and kidney disease health care in terms of cost, locality and waiting times?**

Billy: I think for my case that’s ok, everything that’s alright.

**Q: So cost is ok?**

Billy: Yeah.

**Q: You're not waiting for too long?**

Billy: Yeah doesn’t matter.

**Q: Location is ok, you're not needing to travel a long way?**

Billy: Yeah.

**Q: In terms of accessibility, some people complain about car parking is an issue here at Hospital 2, has anyone in terms of accessibility of the health service, having trouble finding a car park or needing to walk a long time?**

Margaret: Yeah.

Theo: I think the people that park over there, I’m pretty lucky, because mine is podiatry, so with podiatry you come in through the back gate and I just park right at the bottom of the stairs here. So I don't have any problems. The biggest problem I found with this hospital, and this is because I spend so much time here is the quality of the building. The government hasn’t kept the place up with the quality of the service it provides, that’s the biggest problem here.

Tom: I park at Rose Bay Reserve and walk up through the park. There’s a bit of exercise.

**Q: A bit of a hype <inaudible> about exercise.**

Tom: Yeah.

**Q: Billy any issues with parking?**

Billy: Nowadays I can’t drive, because the eye special doctor said is better not to drive, that’s why now I don’t drive, by public bus.

**Q: Is that an issue, does it take a long time?**

Billy: No, no. That’s enough, I have enough time, that’s ok.

Margaret: At times I find the parking, I’ve been able to get into the parking area each time, but sometimes it’s a fair distance to walk and I’ve got, I’m rather breathless, which means that I have to stop every little while, but otherwise there’s parking there. So and today I got in for free, so I’m not complaining on that. The gate was up, so I just drove in. But there’s not enough parking there, because they're parked on the pedestrian crossing, they’ve taken up half the area where you drive backwards and forwards, half the driveway’s been taken up. But I was lucky there was a space, so I just got into it.

Theo: The parking here even down in there, I don’t know they probably need a lot more disabled parking, you got disabled stickers?

Margaret: Yes.

Theo: They probably haven’t got enough of those there where you can park near the gate, they need more disabled parking stickers. But here like it’s so much different coming here, I have to go sometimes to Camperdown at HOSPITAL 12 and it’s just absolutely hopeless there.

Margaret: I’ve got a friend who goes to HOSPITAL 12 and it costs…

Theo: Sometimes it costs a fortune, it costs you $16, $17 to go to the doctor there and that’s if you can get in. Most of the time even you can’t get in to pay that so you might have to walk one, one and a half kilometres.

Margaret: Parking here is good compared to HOSPITAL 12. HOSPITAL 12 is pretty chronic.

**Q: Now similar question, what are some of the things that you find frustrating about the diabetes and chronic kidney disease health services that you receive?**

Margaret: I can’t, I’ve only been on this chronic kidney problem just recently. That’s not quite right, I’ve been going to Dr. A and prior to that I was going to another doctor and they were just checking out with blood tests and that to see how things were going. I can’t say I’ve got any problems with it, just frustrates me that I’ve got the problem. But it’s brought about mainly by the shoulder problem.

**Q: You’ve decided there’s nothing frustrating you find about the health care?**

Margaret: No not really.

**Q: No bug bears or anything like that?**

Margaret: No.

**Q: Anyone else?**

Tom: I’m the same as Margaret, I’ve only been diagnosed in the last 12 months, so I haven’t had that much to do with the dialysis or…

**Q: There’s nothing that you find frustrating?**

Tom: No as Margaret said the disease.

Margaret: yes, yes

Theo: I’d say I’ve been coming for 10 years, I couldn’t think of a complaint that I have with here.

**Q: Billy?**

Billy: For me don’t like <laughter>

**Q: So in other places some people have mentioned things like long waiting times, different advice from different doctors, doctors not communicating well with them, or even some people mentioned a lack of holistic care, so they see many different doctors and everyone’s just worried about their little bit and no one is pulling everything together. Do any of you guys relate to any of those complaints at all?**

Theo: I don't know what you mean by that, do you mean when we go to an outside specialist or just to the treatment in this hospital?

**Q: Treatment in this hospital, these are from other hospitals, not specific in this hospital.**

Theo: Well the only problems I have is going to outside specialists, when you go to their surgeries, you can get an appointment there for 10:30 or 11 o’clock and you wait an hour, an hour and a half. But that’s only in specialists that have got private rooms. I don't have any problem with the hospital here.

**Q: Anyone else, does anyone relate to any of those complaints or disagree with them?**

Margaret: No I wonder at times whether when you go to another specialist if they contact your GP. I know I have, ok I’ve only been with this GP since Christmas because mine moved and I wasn’t going; I’d been going to a medical centre, but I decided I would find another doctor. And I don’t know how well they’ve been, the different specialists have been communicating.

**Q: It’s hard to say because you don’t see any specialists here, but for Canterbury Hospital, do you think they communicate…?**

Margaret: I think she communicates with my doctor, I know Dr. A does. And I know the heart specialist that I go to contacts Dr. A, but I don’t know whether the others do at times or not.

**Q: Does anyone relate to that of problems with…?**

Theo: Yeah I come say every three months to Dr. G here and every time I go to the GP, I go to the GP every two weeks and my heart specialist and every time I go there they’ve got a letter, every time I come here, doesn’t matter if there’s nothing on, Dr. G writes and suggests things or tells them the process and anything like that. So they have reports from him all the time.

Tom: No I see no problem with it at the moment. I see Dr. R and Dr. J and they seem to converse with one another over there.

**Q: Billy?**

Billy: That’s ok for me.

**Q: The other thing some people mentioned is also lack of education or inappropriate education as a complaint or frustrating factor. Do any of you relate to that at all?**

Theo: What do you mean by that?

**Q: Some people mentioned that they haven’t received enough information about their chronic kidney disease or their diabetes, other people say it’s inappropriate, that information they’ve given them is like a telephone book and they don’t understand, there’s too much to read.**

Margaret: You're referring this to other people that have been through like a focus group?

**Q: Yes in other places, do any of you guys relate to that?**

Theo: No I must have a particularly good doctor, because he’s very thorough, he explains everything to me every time.

Tom: Yeah the question on what kind of disease I have, because there’s several kidney diseases and I’m just not sure which one I’ve got. I haven’t been really told, because they're a bit, the doctor said to me, I had the biopsy and they can’t seem to find just what’s causing this, so I’m not sure what disease it is.

**Q: But I guess if they themselves aren’t sure, they're not educating you or it might be hard to tell from the biopsy. Margaret or Billy how has education been for your diabetes and chronic kidney disease?**

Margaret: Yes I think I went to a; there was a program that Diabetes Australia had with some of the hospitals and I went to one at Marrickville Medical Centre. And I think I learnt more at that then I learnt in the previous 20 years sort of thing, but it was supposed to be like a group like this, it turned out that I had someone from Diabetics Australia and a nurse from the medical centre and me. So it was one on two sort of thing and it was a PowerPoint presentation and I learnt a lot there. And it went for two days, so each day I was the only one there. Apparently they're trying to get these sort of programs around the hospitals and that for diabetics, but doctors are not referring too many of their patients to this program and it’s not getting the information out. I went a couple of months ago to a diabetics meeting at Riverwood and I’ll probably go again next Tuesday afternoon and I intend to rejoin Diabetics Australia, because I get support through there.

**Q: Has anyone got anything further they want to add about that?**

Tom: No.

**Q: Have any of you guys ever missed out either diabetes or chronic kidney disease appointment? And if so why?**

Theo: I’ve never missed an appointment.

Tom: No.

Margaret: I don't think so, but I can’t be absolutely sure, because I’ve been going for so long.

Billy: I think that’s ok.

**Q: So you never missed out on one?**

Billy: Never.

**Q: The reason I was asking was I was just wanting to ask if just say your diabetes and your kidney disease appointments clashed, which one would you go to and why?**

Margaret: I make sure they don’t clash.

**Q: But if it did happen to.**

Margaret: Probably the kidney one, because I can control my diabetes probably a bit better than I can control the kidney problem.

Tom: Yeah same as Margaret, I make the kidney priority.

**Q: Billy or Theo?**

Theo: I’m not really sure, I’d have to really think about it, I don't know enough about either at the moment, the decision.

Billy: Maybe I not understand anyway, but kidney just I find two years ago, last year maybe, I forget sorry, from last May. When I find it, when I early morning wake up, my leg is so swollen and so at that time I ask to see the doctor and I know that the kidney problem from that time. And now almost gone the swollen, also good now, now better. Anyway day by day improve.

**Q: Let’s move on a bit, it sounds like everyone is quite happy with the service they’ve received?**

Yeses.

**Q: Now I guess this question is addressed to why you're happy with the service that you receive. So based on your experience what would you say are the strengths of the diabetes and chronic kidney disease health services that you’ve experienced?**

Theo: Why you got to think about that one, I’d say well because you’ve just got faith in the doctors. Some guys you go to the doctors and if you don’t get the satisfaction or you don’t feel better after you’ve had the treatment you think well you wonder if he knows what to do. But if you go to a doctor and you think that you're getting improvement in your health, well you’ve got confidence in him and I’ve got confidence in the bloke that I go to. So that’s probably the main reason I think.

Tom: I can’t really comment, because I’ve only discovered that I have this disease about the last 12 months.

**Q: Or even from your experience over the past 12 months or so, what would you say some of the strengths are of the health services for kidney disease?**

Tom: Well I see Dr. J and he seems to be; I don’t know the strengths would be, what they do they ring you up just in case you don’t remember the appointment, that’s a good, that’s one of the strengths.

**Q: Any other ones that come to mind?**

Tom: Just the receptionist that they give you good service, they look after you. I think that’s some of the strengths there.

Margaret: No I have no complaints about the service I’ve been getting. Dr. A is always very good with the information he gives. He’ll tell me if my blood sugar is up or down over the three monthly period, tells you whether my kidney problem has got any worse or whether it’s better, if it’s not as good as it was the previous month. Get to it and make sure it’s better next month sort of thing. No I can’t complain.

**Q: Billy what do you find good about it, what are the strengths of the diabetes and kidney disease health care that you have, what do you find good about it?**

Billy: I get the feel day by day is better everything and so I’m fine nowadays.

**Q: So can anyone list any other strengths? Some people say the doctors are good, they give good service or stuff like that or they give me information or any other strengths that come to mind for people?**

Theo: I don't find anything bad in it so I suppose they do their best to give you as much information as they can, so I suppose that’s all they can do.

Terrance: Yeah I’ve got the same opinion.

Margaret: Yeah.

**Q: What do you find effective about the diabetes and chronic kidney disease health services that you’ve had?**

Margaret: Well the service that I’ve got it’s kept me as well as I can be, if I wasn’t going to these doctors I, whether I’d still be here or not is another matter. I can’t really complain about anything that they do or tell me. I think that’s sort of it, I get as much information as they can give me as far as I can work out.

**Q: What do other people think? What do you find effective about the diabetes and chronic kidney disease health care that you’ve had?**

Theo: Well I suppose everything that they tell you is effective, because you only come here because you're really crook and they help you get better, so that’s effective. They give you good information about what you need, what would happen to us if we didn’t have these people to do it for us?

Tom: Yeah I got the same idea. Just the information that you receive.

**Q: Billy do you have anything further?**

Billy: No that’s it.

**Q: What impact has attending the diabetes and kidney services made to your health?**

Theo: Well it’s had a big impact on my life, I suppose once you’ve got kidney disease, you’ve got diabetes and that, you're going out the back door pretty quick, so if they can arrest it, stop it, stop it from getting any worse, that’s what they’ve done with me. He had me going under that dialysis four or five years ago and I still haven’t gone on it. Yeah I’ve hung out from then and same with the diabetes, I was on three different types of tablets a day with diabetes and it was pretty high sugar levels and now they're very controllable, they're under five. Whereas they were up between 10 and 12, but that’s from weight loss. That’s contributed to being here for a couple of months I suppose.

**Q: What about other people’s experiences in terms of the impact that the diabetes and kidney health care has made to their health?**

Tom: It’s just with the kidney disease at the moment I’m kind of up in the air a bit, I don't know whether I’m going to go on dialysis or whether they're going to put me on tablets, I don’t know what the procedure would be. So but the impact is pretty stressful, I can say that.

**Q: What about in terms of the kidney health services have made on your health, what impact has it made? Do you think it’s improved things?**

Tom: I suppose it’s improved things because they found out I had kidney disease and they’ve given me some information as far as diet , just what the symptoms are and that kind of thing.

**Q: Anyone else want to share their experiences in what impact it’s made on their health, the services?**

Margaret: I don’t know whether it has any impact on other health problems or not, possibly has, because I find I get very very breathless, but that’s probably nothing to do with the kidney problem and the diabetes, but other than sort of having to take all of the medication that I have to take for other things, it’s I suppose I’m lucky that I’ve got the medical treatment that I’m getting to keep me going.

**Q: Got any comments on that Billy, just in terms of the impact the attending the health services has made on your health in terms of has it been good impact, bad impact or?**

Billy: Everybody maybe the similar cases, but for my experience easily our style in Korean food is very salty, too salty and then doctor said to me reduce the salt, reduce, but it’s not easy, because from young I took the salt food. And nowadays I reduce the salty easily and that’s why improve my kidney, I think so.

**Q: We’re going to move into questions on your experiences of living and looking after your diabetes and kidney disease. Before that can we quickly spend one minute doing the surveys, because that’s all about healthcare and stuff like that? Is that ok, because we’re just going to move into another section of questions, but I thought before that we’ll do the; this will take less than one minute and then we’ll keep on talking. [END PART 1] [START PART 2] So the next lot of questions we’re just thinking about your experiences with living with and looking after your diabetes and chronic kidney disease. So in your opinion what do you need in place to look after your diabetes and your chronic kidney disease well? So in your opinion what do you need in place to look after your diabetes and chronic kidney disease well?**

Theo: I think that I don’t know how bad mine are, but everything seems to be under control. I’ve got my diabetes under control now that I’ve got my kidney specialist I go to every 20 months and he said they haven’t got any worse, they’ve been going up and down and he just changes my medication as it moves, but hasn’t got to the stage in the last couple of years where it’s got any worse. So there’s probably nothing more I need than the support I’ve got now.

**Q: So you mentioned support, so some people mention family support or health professional support?**

Theo: No my professional support.

**Q: Ok any other factors that people think they need in place to look after their diabetes and chronic kidney disease well? So Theo’s mentioned having a good support from health professionals.**

Tom: Just a dietician, I haven’t seen a dietician in a long time, combined with the kidney disease, that’s one of the other…

**Q: What you can eat and what you…?**

Tom: Yeah what I can and what I can’t eat.

Margaret: Mine is the same, last time I was at Canterbury I asked the doctor there if she could get me in to see a dietician, but they don’t have a dietician at the hospital anymore to see outpatients, but that’s one thing I would like to be able to get into and to see a dietician. I know what I should eat and that sort of thing, but it’s just a matter of I think being reinforced by a dietician. In other words sort of say righto yes it’s ok to eat this, but in moderation sort of thing. So that’s the only thing I’d like to be able to do, but apparently the dietician has been taken out of Canterbury Hospital for outpatients.

**Q: How about yourself Billy what do you need to look after your diabetes or what do you need in place to look after your diabetes and chronic kidney disease well? So some people mentioned they need good education or they need support from family or some people say motivation is important to look after diabetes and chronic kidney disease. Do any of you guys agree with those factors?**

Margaret: Yes.

Tom: Yes.

**Q: Which one?**

Margaret: All of them.

Tom: Yeah the motivation. It’s a very hard thing to overcome.

**Q: So other people mentioned things like good social supports or even, some people even spoke about their social circumstances or even having enough or economic factors as being important as well.**

Tom: Yeah that was right, the economic factor, the affordability of things.

**Q: Do you relate to any of those factors Billy just in terms of what factors are important in terms of looking after your diabetes and chronic kidney disease, like family support or dietician or having your own diet or things like that?**

Billy: I must measure and control by myself, now I live with my son, just he visit only and everything myself and then but important for me I follow the Hospital 2, the doctor, doctor’s orders.

**Q: So what you're saying is your own motivation is important and also having the health professional support there is important as well is that right?**

Billy: Yes still that’s ok still.

**Q: So what you're saying is it’s important for you to have your own motivation in looking at diabetes and also it’s important that you have the health professionals to support you. Is that what you're saying?**

Billy: But anyway just I not understand where anyway, but I think in my situation something different from others, but I think that’s no problem for me.

**Q: So has anyone got anything else they want to add in in terms of what factors are important?**

Tom: I think support, family support. Some people don’t realise; if they haven’t got diabetes they don’t realise the impact of it or the implications.

Margaret: That’s true too. I’m unfortunate in one respect that I don’t have any family support here in R City, so I rely on friends and that sort of thing, my support and most of them are pretty good.

**Q: Anyone got anything else they want to add in terms of factors which are important or that they need in place to look after their diabetes and chronic kidney disease well? No ok, let’s move on. So similar question, what things make it hard for you to look after your diabetes and chronic kidney disease well?**

Tom: Well at the moment I’m still working and I do shift work. You're not getting the right sleep, your diet is not always the best, but I try and stay away from junk food and I eat a lot of fruit. But it’s the sleep that mainly is a very big problem, and I think with the kidney disease as well that apparently that you wake up of a night and that you get broken sleep. So I find that a bit hard to overcome.

**Q: Anyone else got anything?**

Margaret: All the nice things you see in shops that you know that you can’t eat.

Tom: Like cake shops.

Margaret: Yes.

Theo: I think probably broken sleep is one of the real bad things, you <inaudible> on that, you start getting up four or five times a night, that sort of <inaudible> especially when you can’t get back to sleep.

Margaret: Yeah the broken sleep is not good.

**Q: So other people in other places mentioned things like having a lot of other illnesses can affect their ability to look after their diabetes and chronic kidney disease.**

Margaret: Yeah agreed, agree with that.

Tom: You mean a partner or?

**Q: No having other illnesses as in they themselves having a whole lot of other illnesses or other diseases can impact their ability, if they’ve got a lot of medical problems.**

Tom: You mean the individual?

**Q: Yes. Some people also mentioned their partner having medical problems and them having to look after their partner.**

Tom: Is that the question?

**Q: I’m just mentioning what other people have mentioned and just seeing whether anyone agreed with those.**

Theo: I think if you have them yeah of course it would be a problem.

**Q: Other people mentioned just feeling bad from the actual kidney disease itself has affected their ability to look after their diabetes and chronic kidney disease.**

Margaret: I don't know that the kidney disease has any effect on me. I don't know that I’ve noticed any change since I was told I had this problem. So I don’t know about that, I know I’ve got hypertension and that sort of thing, so that could probably affect me in other ways, but I don't know that the, I know that I’ve got to be careful with food and that sort of thing. I used to drink a reasonable amount of coffee and that sort of thing and I was told I shouldn’t drink too much coffee. And any caffeine drinks I was told to steer clear of them, but not that they made that much change in my lifestyle.

Theo: With the kidney thing there’s not a lot of pain, you just know that it’s there. So you’ve got to control it with what you eat and all that sort of stuff. Margaret just said with the coffee, I drink the decaffeinated, I find I have one cup a day of the decaffeinated coffee and a bottle of soft drink lasts me for probably a week, and it’s the decaffeinated one. So the doctors just told me make sure you drink a couple of litres of water every day and I make sure that I drink a lot of water, it’s not a pain thing the kidney thing, it’s a problem that’s there that you’ve got to cope with.

Tom: Yeah as Theo said, it’s not a pain thing, it may come later I don’t know, but I find it a bit stressful, a little bit, because you don’t know what the end result is going to be, go on dialysis or you stay on tablet or you don’t know what's down the track or what's going to happen to you. Especially when you get a bit of age on you, no one lives forever I know. I find it a bit stressful I got to be honest, yeah.

**Q: Other people have also mentioned things like having depression or lowered mood or some high anxiety might affect their ability to look after their diabetes and chronic kidney disease and also having a lack of support as well from family or friends or stuff like that. Does anyone identify with any of those?**

Theo: I’ve got plenty of support, I don't need it anyway, but I don’t have any anxiety or stress, it really doesn’t affect me, so I don’t know how it affects people that have got those problems. You’ve got to have those things to know if it makes any difference.

Margaret: I just get a bit stressful when I can’t sleep at night, but that’s not maybe it’s my shoulder at times, because it aches at times. I’ve been free of pain today, so that’s been a good thing, but other times it’ll just ache all day. When that happens I take a coupe of panadol osteo to help it along, but today it’s been good on its best behaviour.

**Q: And other people have mentioned things like getting conflicting information from different doctors or for some people the cost of medication or the cost of traveling has also made it hard for them and things like that.**

Margaret: Well I still drive, so the only time I get annoyed is when petrol goes up to about $1.50 or $1.60 a litre and I’ve missed out getting it, but I think I get very anxious if I lost the use of my car, because I hate public transport. I know other people have to rely on it and that sort of thing, but I just never had to rely on public transport and I couldn’t go to some of the places that I go, so I want to keep as well as I can so that I can keep my car.

Tom: As I said, just I find it a bit stressful, I try and forget about it, I try and put it out of my mind until I’ve got to see the specialist and it all comes back to me. The last time I’d seen him I was a bit pleased coming out of there, because apparently it’s improved a bit. It was going north instead of south, so a slight improvement yeah.

**Q: How are you feeling?**

Theo: I’m fine.

**Q: Do you identify with any of those factors, so things like conflicting information from doctors or the cost of transport and all that sort of stuff?**

Theo: The cost of transport that affects everybody, we’re all tied by that. Costs a lot to travel and something you can’t do anything about it. Everybody faces that, doesn’t matter if we didn’t have a problem, we’d still have the cost of transport.

**Q: Other people mentioned having too many appointments, because often as things progress as the diabetes and chronic kidney disease worsen, people tend to accumulate several other medical issues. And one barrier that people found was just having too many appointments and having to travel to them all the time and they found that they just were going to appointments to travel much of the week.**

Theo: I suppose it depends on what you've got, I mean this week I’ve got Monday, Tuesday, Wednesday, Thursday, I've got Monday and Thursday every week, next week I’ve got Monday, Wednesday, Thursday, Friday, so have at least three appointments a week, sometimes more, but at least three every week. So it’s a lot, but you know…

**Q: Some people find that a bit of a pain or barrier.**

Theo: It is, but what are you going to do about it? It’s better than the other thing isn’t it? You’ve just got to do it I think.

**Q: What do other people think or what have they experienced?**

Margaret: Yeah I’ve got a specialist appointment tomorrow and I know that next week and I think next one week I looked at my calendar and I think I’ve got Monday and Tuesday I have regular programs that I do and then Wednesday, Thursday, Friday I think I’ve got doctors appointments each day. But that would be unusual, it’s just that they’ve all turned up at the end of the year sort of thing. I try and work it so that I don’t have them every week like that. But these things happen and you just put up with it. I’ve got once Dr. A I know that the first and second Wednesdays in the month are free and he’s only at Canterbury on a Wednesday, so I know that the first and second Wednesdays is when I see him sort of thing and the other doctors you can only see them on certain days. So you’ve got to sort of try and spread them out.

Tom: I’m just a bit fortunate at the moment that I’ve just got the one appointment, at the moment.

**Q: How about yourself Billy, what things make it hard for you to look after your diabetes and chronic kidney disease well?**

Billy:.. sorry?

**Q: What things make it hard for you to look after your diabetes and chronic kidney disease well? … <silence>…… You know not enough information from doctors or they're not explaining things to you that you can understand or any of those things make it hard for you?**

Billy: AHH…. I think no hard thing for me anyway, just <inaudible> yeah.

**Q: Has anyone got any other factors they want to mention?**

Theo: No my wife she won’t go to the doctors, but she’s sick all the time, she’s always got her tummy or her back’s crook and she just won’t go and I made her… She won’t go to the doctors, I make appointments for her and she’ll ring up and cancel them. I say you <inaudible> you're coming with me and she just won’t and yet she’s always got problems. I’ve got only these problems, which doesn’t make me feel sick and I’m pretty healthy otherwise.

**Q: Ok now this question is a bit of a fun question, imagine you became a director of the hospital and you had the power to change things about the diabetes and the chronic kidney disease health services. If we were going to design the perfect health service which would meet all your needs, what would that include?**

Theo: I’d first modernise it, that’ll be the first thing and then they built the thing on the third floor over on the other part, have you seen that, that kidney thing they’ve got there, they built a brand new whole floor and they’ve done good there, but it’s always full, so they probably need another floor like that. And I don't know much about the diabetes thing, because I go to another doctor, my own doctor for that, but that’s what I’d do with the kidney thing. I think they’ve done well with it here in the last year. But it’s sort of it was like the tunnel they built to the airport, like by the time they finished it was obsolete sort of thing, it wasn’t big enough, so they probably needed more space, but the facilities and that they’ve got there are good. They just need more of them.

Tom: Well you could pour more money into it, you could pour millions into research. Well there is enough done in this country, but I think there could be more done. Looking after the elderly is something that’s not, but once you’ve turned a certain age you're on the scrap heap. So if I was director certainly as Theo pointed out, this building looks a bit dilapidated at times. I don’t wish to condemn it but…

**Q: No this is where honesty is appreciated.**

Tom: Yeah but it could do with a good paint job yeah.

Margaret: Yeah I suppose I agree with those sentiments. I think one thing if a kidney problem and diabetes run hand in hand, I think probably have the clinics together, so that and also that have enough conditions there that they run on time. I can’t complain, because when I go to mine they seem to run pretty much on time. I don’t take any notice if it’s half an hour, I object when I have a three o’clock appointment and I see the doctor at a quarter past four, which happened to me last week, but it wasn’t for this. But I think that’s a bit much. And that does happen with doctors, I know they can’t help it, but yeah if the two are running together and it seems to be that diabetes and kidney problems seem to be hand in hand a little bit. If the clinics could be in the same area and the people could be referred to the one area sort of thing to see them, maybe run it so that you could see your diabetic specialist at three o’clock and the kidney man at four o’clock, so that you only have the one day that you have to be up. And for some people that could be a help.

**Q: What do other people think?**

Theo: I think that’s a great idea.

Tom: Yeah.

Margaret: But put that to the government and they’d say oh no we can’t do that.

Tom: Yeah I was about to say do you think it will ever come about, because…?

Margaret: I’m just hoping that should there be a change of government, that this program continues on, I don’t know whether it’s been as they say in the thought estimates that it’s been accounted for or not.

Tom: I don’t mean to be too political, but if the next guy gets in, I don’t like your chances.

**Q: When you say next guy are you talking about Mr. Abbott?**

Tom: Mr. Abbott.

**Q: Which one is worse?**

Margaret: Pick a coin.

Tom: Yeah they're all tarred with the one brush.

Margaret: I was listening this morning and I don’t know, this is probably a little bit political, but the ABC, I listen to the ABC full stop, and Linda Motram had the Greens leader on this morning and she’s saying what she’s going to do, her budget she’s got a budget for this and a budget for that and a budget for something else. And I thought to myself how the hell is she going to do any good with what she’s saying, because if Abbott gets in she’s history, if Rudd gets in again she’s still history. So what she is spruiking about, she’s got $100 million for this and $50 million for something else, I thought how is she going to do anything?

Tom: The Liberals they’ll run the Senate, so that’s…

Margaret: Yeah well good, oh Mr. Katter or Mr. *0:26:43 party might get in.

Tom: Oh god help us. Barnaby Joyce.

Margaret: I don't mind Barnaby.

**Q: Sorry guys so we’ve gone from hospital to now politics, let’s go back to the hospital, so you mentioned the joint clinic, so benefits would be?**

Margaret: The benefit would be if it was a joint clinic you’d have appointment for three o’clock and appointment for four o’clock, so that you’ve only got that; for older people like me, one visit out is better than ok I come here today and I got to the other clinic next week and I go to the next one and then three month’s time I’ve got to repeat it again. Or two month’s time however they decide my condition requires it. And that would be a great idea, but as you say you can’t see it happening.

**Q: If we did have a combined service what sort of things would you like to see in that service? Obviously the diabetes dock.**

Margaret: And kidney.

**Q: Anything else?**

Margaret: Maybe have there as well a podiatrist that you could see, because most diabetics see a podiatrist at least every; oh I go about every two months, because I can’t cut my toenails anymore. Other than that what else could you put in a clinic like that? A dietician, so that you've got all those auxiliary things together.

**Q: What do other people think? Do they agree with that idea, anything else?**

Theo: I think that’s a good idea, they’ve got all those in the building over there now, but you can’t coordinate them. As I say I go twice to the high risk foot clinic a week and they're Monday and Thursday and when I go to my kidney specialist I try and coordinate, but he’s only there Fridays.

**Q: What about things like diabetes nurse educator or renal nurse or something?**

Margaret: Yeah. That can help you with any problem that you've got.

Tom: You mean coming to the house or you mean the clinic?

**Q: No through the clinic.**

Theo: They’ve got them in the level three over there, the renal nurses and that.

Margaret: I don’t come here, so I don’t know what's available here, I go privately or to the hospital.

Tom: Yeah I’d seen a renal nurse when they took me through that dialysis unit.

**Q: Is there anything else other people would include, anything else people would do, if you changed things? Other people mentioned things like better education, either to the whole population of better education to patients, or things like pharmacists, contact or cheaper parking or stuff like that?**

Margaret: Easier parking, easy parking.

Tom: Cheaper drugs.

**Q: That’s the government’s job.**

Theo: The drugs are reasonable again.

Tom: There we go again.

Margaret: I can’t complain about drugs, I pay $5.90 for my regular prescription drugs. Some of the other things that you get, I’ve got bad arthritis so I take not fish oil, the other one.

**Q: Glucosamine.**

Margaret: Glucosamine; I went to my chemist the other day and I saw it there for $40 for 300 tablets or something like that and there was an el cheapo chemist not very far away.

**Q: Chemist warehouse?**

Margaret: Well it was one of those yes and I got the same thing for $30. So and they were talking last night on TV that we pay $30 for something that you get in America or England for $10. Now I know it’s like combining apples with oranges, we’ve got a small population and it’s a big, but most of that stuff are produced here, a lot of it is not imported, so why it costs so much here? Then they were saying if they reduce the prices it’s going to put the chemists out of business, but if you can get something for $30 at one place and $40 at another, someone is making a hell of a profit.

Theo: If you went to Costco you’d probably buy it for $20 at Costco.

Tom: You’ve got to have a card there.

Theo: I tell you what it’s worth it mate, you save the first day you go there.

Margaret: You probably do yeah, it’s like some people go to Aldi.

Tom: Just on that question of cheap drugs in America, but you wouldn’t want to be sick in America.

Margaret: Oh no way. The health care would kill you.

Tom: Yeah.

**Q: So anything else anyone wants to add? It’s not every day you can be director of a hospital and you get to try to change things, you get to help design a diabetes and kidney disease health service, what else would people want in there?**

Margaret: Decent numbers of staff to look after the patients that come in so that they're not waiting all these hours that…

**Q: This is where they come in to hospital is it for in patient or out patient?**

Margaret: Oh a bit of both.

Theo: But the wait in hospitals that’s controlled by doctors, not by the nurses and the secretaries, it’s just the availability of the doctor. They can’t do anything about that, the nurses that work there. That’s a great suggestion if you can go and have it all in the one thing, say it was the second, third and fourth floor or something over there, you had the clinics on each one, it’s very hard in a small hospital like this, because most of the clinics aren’t open every day all day. It’s only the kidney thing is open there all the time, but the diabetes one is not and the foot clinic is not open there. It’s only three times a week.

**Q: Any other ideas or people would like to include in the ideal service?**

Tom: More nurses.

Margaret: Yes.

**Q: A pharmacist contact?**

Margaret: I can’t complain about pharmacists, because I go to the one pharmacist all the time and ok she’s not a pharmacist that I speak to most of the time, but if I ask a question there’s always someone there that will come and answer it for me. If I get a script and they're very good in this that if I get a script for something different, they’ll come and say have you had this before or you shouldn’t take this with that. And so I can’t complain about my pharmacist there, but that is something you do need a good pharmacist.

Theo: I don’t have any problem with pharmacists.

Tom: No I can’t say, I got another condition, but I had to ring up and get the script, sometimes you’ve got to ring a week before you run out of your medication, otherwise they may not have the stock, so that’s not their fault, that comes from somewhere else, but that’s the only thing. But I find they're pretty efficient.

**Q: What about better education?**

Margaret: Yeah could help.

Theo: For who?

**Q: For you guys with diabetes and chronic kidney disease?**

Tom: Yeah.

Margaret: I think better education for the general public too overall.

Tom: Yeah overall.

**Q: Can you explore that idea?**

Margaret: Well they say there are almost as many diabetics that don’t know they're diabetic as there are diabetics. I’ve got a couple of friends who I think would be borderline diabetics, but I’ve sort of, have you been checked out for diabetics? Oh no I’m alright, my blood tests are ok, but does make me wonder at times. I think better education generally can be important.

Tom: Yeah I only found out I had diabetes because I got a great big cyst under my armpit, sorry it was an abscess and I went to the doctor and she said oh you're diabetic aren’t you? Then I wasn’t really taking any serious notice of it at the time and; but at that time I wasn’t really educated, the doctor, I don't want to talk about the doctors, but the doctor I was seeing before that he didn’t give me any information or tell me that it was a very serious disease.

Margaret: Yeah I think probably it was my mother that picked up that I could be a diabetic, she and I went away for a week’s holiday and I was drinking water like it was going out of fashion. And she said have you ever been checked out for diabetes? I said no why? She said well I think you should, so when I got home I went to the doctor and yes I was diabetic, so that was how that was picked up.

Tom: My mother is diabetic. So I don’t know whether it’s genetic, it’s hard to say or is it just a bad lifestyle. Because I was a bit like Theo, I was carrying a lot of weight at that time and I lost about 30 kilos at least.

Margaret: Wish I could do that.

**Q: So anything else people would want in an ideal health service for diabetes and chronic kidney disease? So we mentioned a joint service with diabetic doctors there, the renal doctors there and perhaps a whole raft of other Allied Health people, podiatrists [overtalk].**

Theo: The other one with those would be I think the dietician for something.

**Q: Dietician educators, maybe podiatrists?**

Yeses.

**Q: Eye doctors maybe some people have mentioned.**

Margaret: Yes.

Tom: Yes.

Theo: You can have all sorts of people in the clinic, but the three most important are those three.

**Q: So you reckon the diabetes and renal the dietician?**

Theo: The dietician, well if you can control your diet you can control your kidneys and your diabetes.

**Q: Ok anything else people want to mention? Billy anything you want to mention?**

Billy: No.

**Q: Were you going to say something Margaret?**

Margaret: I think in the ideal clinic like we’ve mentioned would be the main things.

**Q: This is the last question, I just want to put it now, is there anything that anyone hasn’t mentioned, that they would like to mention or that anything that I missed asking you that you would like me to ask you about in terms of your experiences with diabetes and chronic kidney disease or your experiences with the health services? Any burning points or things people want to mention?**

Margaret: I think the health service is probably doing as well as it can, I think there’s probably always ways that it could be improved. If the facilities and the money was made available to it, but otherwise I think we’re as far as I’m concerned I think I’m fairly well looked after in the health service.

Tom: I think all problems come back to money I guess, if there’s more money poured into it, it could be improved a lot.

Theo: One of the things that I’d like to see them do, it sounds a bit, I’d like to see them make a video of diabetes and kidney problems and just make a video, so rather than giving you sheets of paper like that that people find it hard to read, stick it in and watch the DVD.

**Q: So what you're talking about is educational video DVD or something?**

Theo: An educational video on what causes it, what you can do to stop it or what you can do to arrest it and all that sort of thing, where you can put it in and watch it and do it in your own lounge room and it’s there for you to see and you can write, rather than reading a ten page document or something like that, that you don’t understand or anything like that. But it’s explained properly on a video of both of the things. I thought it would be I suppose a bit of money in the making, they can make videos now for a few cents each. And just to get one there and you can learn so much more from finding why you got it, what you do to stop it, what help you need and that sort of thing, what medications and what diets you should be on for just both of those things. I just think that would be such a great help to people.

**Q: It’s an interesting idea Theo, so you were mentioning too, you were thinking <inaudible> Margaret?**

Margaret: That program that I went to at Marickville Medical Centre, that is very similar to what Theo mentioned. I felt I got a booklet with it and information with it, but it was done on a PowerPoint presentation and it was excellent, it sort of explained how everything worked. Now I think it was run by Diabetics Australia in conjunction with various hospitals that had diabetic clinics. I don't know whether they’ve ever done it here or not, but I felt that it was something well worth seeing and as I said I learnt more from it than all the other things that I had been to.

Theo: I think they're great things, but I just think it’s much better if it’s on a DVD and they give it to you.

Margaret: Well they can probably put this on a DVD.

Theo: It’s much easier to look at than <inaudible>.

Margaret: Yeah well he just had his computer there and he brought up the various slides as they came, but I’m pretty sure it could be put, probably could be put to a DVD.

**Q: Well it’s interesting you mention that, because in another place someone mentioned that people would be screening educational stuff on the TV while they're waiting in the waiting room, instead of watching Fox News or Fox Sport or something.**

Tom: That’s right yeah.

Theo: That’s good, but you can’t beat the one to take home, chuck it in your DVD thing at home and especially us people that have got it, it really explains things to us, right the whole thing. It need only go for 15 minutes, but it could be really absorbing for you.

Margaret: Well this program that I went to, it lasted I think it was two till four, it was like a two hour program, but it was supposed to have been like this, a group, but it turned out to be just me. I should have learnt you know a heck of a lot, which I did.

Tom: When I went through the renal unit the nurse at Glen Stuart she gave me some DVDs, but that was only on how the machines work. You can do it at home, you go to hospital, there’s some ways of doing it.

**Q: So Tom they gave you was it like an education session on dialysis and the options?**

Tom: Yeah I took it home and watched it, but it didn’t tell you about the diabetes or what the implications were combined with kidney disease.

Theo: What causes, what the implications are, what you do to arrest the thing.

Tom: Yeah that’s my biggest concern is that so far I haven’t been told what I should be doing.

Theo: That’s right, but that could be all on the DVD.

Tom: I might be on the wrong diet, I don’t know. I don’t drink or smoke, so I’m a bit fortunate there, but otherwise food wise I only found out from the GPs, I don’t eat a lot of salt, which I did. And then Margaret mentioned coffee, which I didn’t know about.

Margaret: Caffeine.

Theo: Well you get decaffeinated coffee.

**Q: Anyone got anything else they want to mention about their experiences or health services, about diabetes and CKD? That was an interesting point that you brought up wit the DVD and the education.**

Margaret: Who put me onto it? Canterbury Hospital, no… I can’t remember how. I think Dr. A might have mentioned to me about it. And because Canterbury ran it in the morning and it didn’t suit me, Tuesday morning and it didn’t suit me and I rang them to see if they had one in the afternoon. They didn’t, but they referred me to Marickville, they had one in the afternoon.

**Q: So any final words, experiences, words of wisdom or anything?**

Theo: No.

**Q: Billy any final words you want to say? No?**

Billy: That’s ok.

Tom: I think we covered what we could.

Margaret: I think we covered it very well.

**Q: Yes very thorough thank you guys.**

Margaret: We hope we’ve give you some food for thought.

**Q: And while we’re talking about food for thought, dig into the food. I think we filled up all the surveys. We’ve just got a small token of thanks.**

END OF TRANSCRIPT

**Focus Group 7**

**Q: So my name’s C, I’ll be helping to run the focus group I guess.**

I’m Tony.

I’m Rowena.

And I’m Bernard.

**Q: Excellent. Let’s move onto the first question. I’d like you to think of your experiences that you’ve had with the diabetes and chronic kidney disease health services here at the Hospital 3. Based on your experiences what would you say are some of the weaknesses of the diabetes and chronic kidney disease health services here, or areas that could be improved?**

Tony: I don't think I’ve got any complaints. In the olden days before the new hospital I thought the diabetes education unit was a bit out of the way and so on but I don't use it that much because I get very good service from my GP and now that it’s moved here I believe it’s behind security screen that’s in this hospital. So I haven’t any idea whether they still give the same services that I used to but nobody’s got to tell me anything about diabetes education unit, I learnt that through talking to my endocrinologist. But do they still have a shop, can you still go in there to get needles or things like that, I wouldn’t know.

Bernard: I’ve got a very high opinion of them, well I’ve had but I should say I’m only a short way into the system because I don't have dialysis for example. So my main contacts are seeing the specialists which I’d see regularly. So I don't rely much on my GP except for referrals and prescriptions and so on because I use the actual specialists to give me the advice that I need. When I first came in with some of these things I got very good hospital attention so I was very happy with the hospital I have to say, and then the specialists I had then are still the specialists I have now, several years later. So I have a limited experience in that sense but it’s been a very good one.

Rowena: I’ve been labelled with diabetes for 11 years. I’ve been coming here every three months for service and I’m very happy that I receive good service from the doctors and the hospital as well here. It’s been very hard for me to accept it. I was struggling about taking medication because I refuse to take it but I have so many help from the doctor and also complex nurse health services which they always come with me all the time. So they help me for the management. And I’m happy here.

**Q: What about with the chronic kidney disease?**

Rowena: The chronic kidney disease, my kidney doctor, they’re very happy to help me and I’ve waited to have a dialysis so I am so happy with the service.

**Q: So it sounds like everyone’s quite happy with the services they’ve received?**

Bernard: Yes, I just want to add one other point. I was really surprised and very pleased that the doctors, even though they’re specialists, they actually communicated with each other about me, even while I was there. One would ring up the other to make sure the medications...

**Q: So this was when you were seen in the clinic was it?**

Bernard: Yes that’s right. So I was surprised at that. I thought that was excellent.

**Q: So I was going to say it sounds like people find the service very good and some of the reasons I think like you’ve said they communicate well with each other, you’ve also said they gave you a complex care nurse to help you along, especially at the start when you had difficulty accepting the diagnosis and that can be a big thing because it is a big lifestyle change so totally understand that. Are there any other strengths of the health service with the diabetes and chronic kidney disease? I think we’ve established that everyone’s found it good and we’ve mentioned some of the strengths. Any other strengths that people can think of or any other reasons why they find the service good?**

Tony: My endocrinologist, if I can’t get satisfaction from any other doctor and I see plenty of them, I always go to her and she fixes me up. Last year my legs were on fire and my GP just said to me “Go home, put your legs up and lose weight” and I knew there was something wrong with me so I rang Dr DL up and she said “You’d better come and see me” and she took one look at my leg, put her hand on it and was like “Oh!”, like that, and that night I had the nurses coming and giving me massive... I had cellulitis. And I find Dr DL is very good that way that if things go wrong anywhere else in my system she’ll always give advice and so on.

**Q: So she’s happy to look at you as the whole person rather than just dealing with little bits?**

Tony: Yes and Dr BC for instance talking about dialysis, he said “One day you’ll be on dialysis” so a few years ago I had HepB vaccination because of it and little things like that. I find the doctors here very good and they help.

**Q: Anyone got anything else they want to add further in terms of why they find it good or the strengths?**

Bernard: I haven’t been to any of the public clinics and so the experience may turn out to be very different but the private clinics, if that’s what you called them, that Dr BC and Dr Ward, the heart specialist are up in the fourth floor private building.

**Q: Yes but you don’t pay gap though when you go…?**

Bernard: No, that’s the important thing, no I don’t. No.

**Q: So in effect it is like public I guess because you’re just paying Medicare.**

Tony: Yeah.

**Q: There’s no gap is there?**

Tony: Well I’m a pensioner and they seem to have a general rule the specialists in the specialist consulting unit that if you see one of the specialists in the private rooms…

Bernard: Yep, you pay.

Tony: Such as my heart specialist and so on then you do get charged more than…

Bernard: Yeah, whereas because all mine began, all my specialists began because of an incident in the public hospital, I paid Medicare for all of them which was lucky.

**Q: So anyone have any other comments about the strengths or what they like, why they find the service good or anything like that?**

Bernard: Just commenting on that going to the clinics up in the private hospital, even though as we’ve said they run as public, kind of mix up in the private hospital, they’re always courteous, the secretaries are great, they’re always pretty well on time which is very important and they give you time, that is they let you talk about what the problems are and so on and as I said they make extra efforts to try to track things down and so on. So that’s all very pleasing I think and also I have always had good experiences with Dr L as well.

Tony: I find the staff up there are very good and I like to be educated.

**Q: What do you mean by that Tony, that you like to be educated?**

Tony: Well Dr BC takes two of these renal nurses up who man the nurse’s station up there and the first time I encountered them they lay me on their inspection couch to take my blood pressure and I said “Hang on, I’m a diabetic with peripheral vascular problems, you never take a diabetic blood pressure lying down” and “Oh we have to” so I let them, I said “Now take it with me standing or sitting” and they reluctantly did. When I went in to see Dr BC I mentioned it to him and he said “Oh I’d better speak to them hadn’t it?” and ever since then never been asked to lie down and have my blood pressure taken. These are things I’ve learnt from going to the American Diabetes Association’s website which I think is a fantastic educator compared with the Australian Diabetes Association which tells you bugger all.

Bernard: That’s another set of questions, but I’ve found that the Australian Diabetes people, spokespeople and so on, they will give you advice that I would say these days would be regarded as out of date, particularly with respect to carbohydrate. They’ll be still telling you to take small carbohydrate meals, six a day, whatever whereas that’s all been pretty well ruled out by more developed approaches, later approaches, like on the American website and so on and you only know that by going to the web and tracking through not just the official advice but the Q&A sections that follow those. That’s the way I think I’ve become educated.

Tony: I have the Mayo Clinic sending me stuff virtually every week or second week because you can put in what fields you’re interested in, so I put in diabetes, I put in something else and for my wife I put in osteoporosis and then they have a general newsletter and that comes in and it’s good reading. But you try and get that anywhere in Australia, and I don’t care whether it’s… I don’t think the Garvan Institute or Walter & Eliza Hall down in T City have anything like it whereas you know the American websites are absolutely fantastic and it’s all there.

**Q: That’s interesting, thanks for bringing that up. So any other strengths people want to mention? No? Okay. Now next question, is it easy for you guys to get the diabetes and kidney care healthcare that you need? So in terms of locality, cost and affordability and waiting times, is it easy for you to access or to get the diabetes and kidney care that you need?**

Rowena: Yes I’m receiving very easily access with all what I need from my doctors and from the service here. So yeah.

**Q: So in terms of locality is okay, the cost and…?**

Rowena: Costs, yep.

**Q: And the waiting times is all okay?**

Rowena: It is okay, yeah.

Tony: Yeah. I live in Mossman so Hospital 3 is very convenient and my wife and I have often thought if we moved where would we move for access to our doctors. It’s obvious you have to be on one of the train lines that go through St Leonard’s. I have no trouble because when I see any of my specialists, when I saw Dr BC last week he said “Make an appointment for six months” and I made an appointment then and there on the spot for six months and I know, like with Dr DL, if I need her urgently I’ll send an email off to her and her PA and I’ll get a reply within 24 hours. So I have no problems at all. At the moment I have another problem within my waterworks that is being handled by a urologist but I know the urologist and Dr BC are talking to each other. So I’ve got no complaints at all.

Bernard: I’ve got no complaints at all. Obviously being on Medicare suits financially. I live at Warrawee so I just come down the train to St Leonard’s, so that’s convenient. And the timing, sometimes I’d like to get on a little bit earlier but that’s usually not their fault, it’s usually my fault. I get to see them pretty regularly during the year.

**Q: Now another question, and this might seem like it’s coming from left field but it sort of arose from some issues which arose from other places. Have any of you guys ever missed out on a diabetes and chronic kidney disease appointments at all and if so what was the reason?**

Rowena: Yes, many times. The reason because just it’s I don’t want to go to the appointment.

**Q: Was that during the time when you were having trouble adjusting to the diagnosis?**

Rowena: The diagnosis, that’s right, yes. So that’s why now I have a complex coordinator monitoring me all the time and taking me to hospital and drive me around to my doctor appointments.

**Q: So did you say you catch public transport to come here?**

Rowena: Sometime I take myself, I drive or take public transport, yes. It’s easy for me…

**Q: When you take public transport, is it a long…? I mean there’s a train station quite close to here isn’t there?**

Bernard: Yes. It’s all uphill.

Rowena: It’s easy here because I live in North Shore, Berowra it’s easy to catch the train to come to St Leonard’s yeah. The problem is me because sometime my appointment is early and I don’t like to get up early morning and it’s just an excuse that I don’t want to go see the doctor. So now I have that complex nurse that all the time monitoring me so I have to get up and go.

**Q: What about you gentleman, have you ever missed out on any appointments?**

Tony: I might ring up and say “I can’t make it, can you move me?” but I’ve never been scared to come and tell a doctor anything.

Bernard: The same for me. If ever I have missed one it’s usually because I’m travelling or something like that or something else has come up and so I’ll ring up and get another appointment and I’m always delighted to come. Far from being scared, I see them as being a positive benefit for me so that’s’ my attitude.

**Q: The lead on question to this is just say you have both your diabetes and your chronic kidney disease appointments and they clashed on the same morning, which appointment would you go to and why?**

Tony: I can’t see them ever clashing. If I did make an appointment here to see Drew L and over there to see Dr BC and I got home and noticed they’re clashing, I’d immediately ring up one or the other person to move it. But I think it would be fantastic if they were both together on the same day, maybe half an hour or an hour apart sort of thing.

Bernard: Yeah.

**Q: Say if theoretically they did clash…?**

Bernard: So you’re really looking for priority here to us?

**Q: Yes.**

Bernard: I would put the kidney one ahead of the diabetes one.

**Q: Any particular reason?**

Bernard: No, except I’m probably more used to the diabetes and I see the kidney one as more dangerous but I could be wrong.

Tony: I don’t know, it’s a hard thing because before I see Dr BC they take eight litres of blood out of me for a blood test which goes to Dr DL and my GP and Dr BC and so I go to Diana and she will tell me if it’s good results or bad results. I go to Dr BC he tells me the same and my GP tells me the same because I said “I get a copy in the mail anyway and I can see for myself”. So I’ve never even thought about it because it hasn’t happened and I don’t think it’d ever happen.

**Q: Can I put you on the spot and as Bernard aptly said, it is really a question about priorities. So just say they did clash…?**

Tony: Well I’d do the endocrinologist before I did the renal.

**Q: Any particular reason?**

Tony: I don’t know, I think diabetes probably…you know, controlling your kidneys, the problem’s with your kidney more than kidneys are playing havoc with your diabetes.

Rowena: I’d go to kidney.

**Q: Any particular reason?**

Rowena: Because the most important is the kidney now because at the moment I have the problem with the kidney, you know, so all my specialist doctor is focusing about the kidney because one of my specialist doctor, he said I will have a kidney transplant, what’s it called, artificial kidney and they were worried about because I was still young to do it. So I’m focusing with the kidney at the moment and I have appointment every time with the kidney doctors. So I have an appointment for transplants and Dialysis here.

**Q: Sure. There’s actually no right answer to this question, I just want to explore all your priorities. Okay, now as a follow on question to that, what are some of the things that you find frustrating about the services that you get for your diabetes and chronic kidney disease?**

Tony: I don’t find anything. Not frustrating, no.

Bernard: I get a little bit frustrated when I get say a bad reading on one of my kidney printouts and I know there’s nothing I can do about it for several months probably and so there’s that period of time. I guess I could ring Bruce up but I haven’t felt it was that bad yet but when you’ve taken action to reduce a particular reading and then it goes up instead, I find that a bit frustrating but they’ve traced all that in my case down to probably my medications. So they’ve been varying all the medications to try to modify it and I’m still to see if that’s successful. But otherwise it’s fine.

Tony: When you get like my pathology report, they show you the previous times you’ve had tests and so on so you can see how it’s varying and whenever I see Dr BC he says “Everything’s right, it’s within the limits” and even though the pathologist might have it in bold with a note saying that it’s outside the limits, Dr BC is quite happy with it. So I’ve learnt from what he tells me and reading the results that everything is okay so for that reason I probably don’t get frustrated.

**Q: Are there any aspects of the health service that you find frustrating? So say other places people have mentioned things like long waiting times, too many appointments, conflicting advice from different doctors, a lack of holistic care where they’re seeing many specialists and no one’s pulling it all together. Can you identify with any of those?**

Tony: If I get a doctor like that I drop them immediately. For instance, one of the things I have to have is a left knee replaced which I’m putting off because I hear too many people with diabetes getting secondary infections but I went to see a certain specialist in another hospital down the road and because of my kidney problems I can’t take normal anti-inflammatories and through the rheumatology department here a couple of years ago I did a fish oil study which had to do with building up cartilage and R City University found it was a dismal failure but I learnt that it was a fantastic anti-inflammatory for my problems. So I went to see this doctor and he was saying “How much do you take?” and I told him and as he’s talking to me he was scribbling. Blow me down he sends out this report to all my doctors saying that “Over the last five years he’s consumed 30 litres of fish oil and no wonder he’s as fat as he is”.

**Q: Is that what he wrote in the letter?**

Tony: That’s what he put in the letter. I’ve got a copy of it, I’m not saying how I got a copy of it and because of my problems I’m in the physiotherapy department here on an exercise programme and I’ve shown it to the physiotherapist and I said “Look, you’re allowed to laugh, I won’t take offence” and everybody that sees it laughs because of all this ridiculous… And he charged over twice as much as the scheduled fee. I haven’t gone to him; I’ve found a fantastic orthopaedic surgeon here. I mean it’s my choice and I kept telling my previous GP that he refused to give a referral for more than 12 months, “We have to keep the specialists in check”, I said “Hang on, it’s my choice what specialist I go to, it’s not your choice, I get your advice”. So I think I’ve got a fantastic team of specialists and I’ve got no complaints about anyone.

Bernard: Yeah, I’ve got a fantastic team too. Just happens to be almost the same team as Tony’s got. I’ve got a heart specialist who’s different but the others are the same and they are terrific. There’s a bit of trial and error in it all but you get there.

Rowena: For me I have no problem with my specialist doctors and with my GPs, they communicate with each other, yeah.

**Q: So some of these things I mentioned about not being communicative or long waiting times or conflicting advice, not an issue for any of you guys?**

Tony: No.

Rowena: No.

Bernard: No. Both Dr BC and Dr DL are extremely helpful in giving you feedback and talking through any issue and so on, yeah particularly.

**Q: So we’ve spoken about weaknesses and there doesn’t seem to be any, we’ve spoken about frustrating factors, again doesn’t seem to be any, spoken about strengths. What do you find affective about the diabetes and chronic kidney disease health services that you’re receiving?**

Bernard: Well I’m still alive.

Tony: Before Dr DL I had another endocrinologist who I saw at his home and he wanted to concentrate on his juvenile diabetes rather than adults and that’s when I went to the Diabetes Education Unit and asked for a list of all the endocrinologist here at the hospital. I then went to my GP of the day who is three GPs ago - this bloke because he didn’t have much people with diabetes - but I showed him my list and he said “Oh, I don’t know, I haven’t heard… I faintly heard of Dr DL”, so that’s how I happened to go to her. I think again it’s a lot to do with your own choice. Like this other problem I’ve got with my waterworks at the moment, my GP gave me the name of two urologists and with the web you can find out a lot can’t you and so one of them I looked up and he had four lines and the whole lot had to do with prostate cancer whereas the other bloke had quite a lot. It turns out he’s a believer in keyhole surgery and robotic surgery, lectures at Macquarie University Hospital and he’s at the North Shore Private and the Mater and so on and I thought he sounds far better for what I want. Then I find out from Dr BC, “Oh he’s fantastic. When we do kidney transplants we use him for the keyhole surgery” and I thought bloody hell, how big a keyhole to get a kidney through? But you know, it’s up to me to choose. I look to my GPs or whoever for advice, but it’s my decision.

**Q: Do other people have that same attitude?**

Bernard: I had a hernia operation and I went to Newcastle to get the surgeon that I wanted, not because of the advice to me and he did a great job.

**Q: Rowena, would you agree with that attitude what Tony’s been saying?**

Rowena: Yes.

**Q: So have you got anything else you find effective that you want to mention before we move on?**

Bernard: I’ve got limited experience you see because I’ve only gone so far…

**Q: Yes of course but from what you’ve experienced, what do you find effective about it, what aspects?**

Bernard: At Hospital 3?

**Q: Yes and the experiences you’ve had with the specialists here at the Hospital 3.**

Bernard: Well all the specialists have been great here. Dr BC, I think I just hit a gold mine sort of straight off with Dr BC. He’s very caring and gives you the impression that he knows it all without being smart about it. He uses the computer a lot, when you’re talking to him if you mention something that he’s unsure about he gets it up straight away in front of you and then discusses it. You get the impression that he’s immersed in what you’re telling him. So that’s a very positive experience I have there. And Dr DL is cut similarly actually.

Tony: This urologist I went to when he looked at my list of specialists he said “Gosh, Dr DL, you can’t get anybody better than that. She’s the most fantastic communicator” and he’s not even in the public hospital here. And that’s why if I ever got anything done, if I can it will be at North Shore Private because my specialists are here and they can still tend to you.

Bernard: I knew she used to be the, I don’t know what you call it, the endocrinology fellow when she was still climbing the ladder as it were, so she was pretty outstanding even as a younger doctor.

Tony: And I think the whole thing with North Shore, I have a fair idea of what goes on at Hospital 10 because my brother’s there but last year I was invited to a social night they had here because of the fact that Dr DL used me as a specimen and she wasn’t at this, Dr BC was representing the hospital staff but it was a prizegiving for the doctors here. So most of the doctors would’ve been one year out of graduation or something and I was surprised the things that they give prizes for at this hospital. I thought that’s fantastic and I mentioned it to my brother and he had to admit that Hospital 10 doesn’t do half as much as Hospital 3 does. So I think I’m lucky to be on this side of the harbor that I’ve got a good hospital.

Rowena: Yes, North Shore Hospital I’m happy with because of all the service very good and I’m happy with Dr Hocking that she’s very caring and all the doctors here, my eyes doctor, specialist, they’re working with each other. Yes so I’m happy, so I have no comment.

**Q: What impact has attending the diabetes and renal health services made to your health?**

Bernard: Extraordinary because I don’t think I would’ve even been all that aware of how serious things were until I was told, in other words I was functioning at a high level for a while anyway. So it’s been an education process for me which came about because I was involved in these services that are provided here at the hospital.

Tony: When I first came down with diabetes, as I said 25 plus years ago, I don’t think doctors generally were as educated then as they are today about diabetes. I came down with Bell’s Palsy and I saw an ENT bloke who happened to be a school mate of mine and he put me on 15 or 16 prednisone refusing one a day and after a couple of days I started seeing two planes flying in the sky and every pole had two lights on, and this was at Hospital 10, and he lives at Mossman, and he was driving me home and I said “You know that Toyota sign” and you might remember a Toyota sign in Williams Street, it was enormous.

Bernard: Oh the big one, yeah.

Tony: Yeah and all it was was a red blur and he had absolutely no idea what was occurring and the next day I was sitting in the urologist getting needles stuck in my face to see how… And I just mentioned this in passing and he burst out laughing and he said the problem with doctors today is they have no idea what they’re prescribing. He said “I suggest you go and see your GP tomorrow and get a blood test done” and my blood sugars were up at 32 or 33. In those days doctors generally didn’t know that cortisone affected the pancreas and so on, which today they do know. I mentioned it to my brother who happened to be an anaesthetist in cardiothoracic unit, he was involved with the heart transplants and he dashed in to see if the nurses in intensive care and all he knew was because it was in the days of Fiona [Coot] *0:39:23.8 getting her heart transplants, that you got moon face and women grew facial hair and said “You just write the scripts, we have to fix up your messes”. But if that had happened today, warning bells would go out that it’s pre diabetes probably. Then 18 months later I came down with tingling in one foot and it moved up my leg and across my torso and down the other leg over a 12 month period and I was seeing a very good physician at the Mater Hospital and he didn’t want to say anything because he thought he knew what it was but he’d looked up all his books and I wasn’t following what the books said. After it got down to my other foot he decided that I had peripheral diabetic neuropathy. So my brother immediately got me an appointment with a urologist at Hospital 10 and I had a thorough examination and he said “I don’t know why they’ve taken so long to diagnose you. I would’ve picked it” but then the books say you can’t get proof for diabetic neuropathy unless you’ve been a type 2 diabetic for 12 or 13 years. They might’ve changed it these days but that was when I got it. That’s why I put a question 25 question mark the number of years I’ve been a diabetic because nobody knows. So education today for everybody, doesn’t matter if you’re a doctor, a nurse or a mechanic is a lot better than it was 25 years ago.

**Q: So Tony, what impact do you think attending the health services has made on your health?**

Tony: Initially it made me realize how dangerous diabetes can be. Now probably I’d say 50% of my diabetes education is self education by reading Mayo Clinic and American Diabetes Association websites.

**Q: What about yourself Rowena, what impacts has attending the diabetes and chronic kidney disease health services here made to your health?**

Rowena: Well it’s just before I didn’t care about myself you see. I have diabetics and I didn’t care at all about it until I get the diabetes education now and an educator and I learn from them that it’s really very serious and I have to take it seriously. This is my benefit, yeah.

Tony: Diabetes education unit when I first went to it…

**Q: That’s the one here isn’t it?**

Tony: Yes, and I saw a nurse at the time. When I was put onto insulin I bruise very easily around the torso and I started doing my legs, he said “Don’t do that because it dissipates too quickly”, my GP of the day said “He hasn’t taken into account that you only shuffle around the place” so it’s not as if I’m out running and so on and that’s why I’m doing in this physiotherapy department now to get more muscle tone. So I inject myself in my legs. I saw a dietician and she fixed me up on diet. So again with this thing I’m doing physiotherapy department, I saw a dietician two days ago and she was happy virtually with everything I was doing. She said I had too many carbohydrates for breakfast in the morning and to move one of them to the evening meal but other than that she said there’s nothing wrong. So I think the education I got years ago was very good and at the time, last time I went to the diabetes education unit for education they told me because I was doing everything and only come back if I needed them.

Bernard: My diabetes education was mainly in Newcastle and it was extremely good. Went to a two day seminar and you’re supposed to go with partners and most people did and they take you through everything, and that’s where I seriously discovered the impact of carbohydrates on everything. So that education process turned me around in that sense. If I can just make a point back on Dr DL again; I take byetta and it’s to suppress appetite but it also has some control over blood sugar and blood pressure and because I was becoming nauseous after every jab which isn’t much fun when it’s happening every day, I moved from ten units back down to five units, but in the process Dr DL tried to find, there was a new drug out similar to byetta that didn’t give the nausea effect and she explored it with, is it Dr Gad Frank?

**Q: Yes.**

Bernard: She consulted him about it and it has an impact on the kidneys so they didn’t put me on it but if I’d gone to almost anyone else I would say they would’ve just put me straight onto it and it would’ve been more trouble for my kidneys. So I thought that was pretty good.

Tony: Dr DL several years ago now, she always brings it up at these tutorials, put me on something for cholesterol and I’ve forgotten what it is, she remembers. But within a week I’d put on 10 kilos in my legs, they were out like this and I rang her up and told her and she said “Come and see me” and she couldn’t believe it, she said “Stop it immediately”. They’re looking at my beautiful feet these students and so on and she always brings that up that you have to be careful with diabetes patients with medication and monitor them all the time.

Bernard: You do. The trouble I’ve had most of all in my treatments has been that the medications have caused things like swelling of the ankles. I don’t have any of that trouble now because they’ve taken me off the medications that have caused it. And when I used to feel absolutely like I was about to cash in my chips it was due to beta blockers, I could hardly move when I took beta blockers but the GP had put me onto beta blockers. Anyway eventually I was completely taken off beta blockers. So medications can be deadly.

**Q: Definitely. Let’s move onto your experiences of living with and looking after your diabetes and chronic kidney disease. We’ve talked a lot about the services and how happy you are with them, let’s move onto your experiences with living with and looking after and managing your diabetes and chronic kidney disease. So in your opinion what do you need to look after your diabetes and chronic kidney disease well?**

Bernard: I need the medications that I’m on clearly when I’ve now worked out over several years to be levemir to see me through the night and I take byetta twice a day and I usually have one jab of humalog, maybe sometimes two.

**Q: If you don’t mind me asking you, are you on private scripts for some of these?**

Bernard: I guess they’ve all been on private script at some stage. The tradjenta, the levemir, but I think I’m one of those patients that can have tradjenta subsidised and I think the idea is that moves me off, what does it replace tradjenta?

**Q: It will be a insulin sparing sort of effect, similar to the byetta.**

Bernard: Right, well I’m on both byetta and tradjenta but I’m on a reduced dose of byetta. What’s the core of the question?

**Q: So what do you need to look after your diabetes and chronic kidney disease?**

Bernard: Well I need my medications which as I say over time with the specialists we’ve worked out and I need not to do certain things, so I need not to eat carbohydrates. I mean you can’t avoid them completely but I’ve virtually cut carbohydrates out of my diet and I eat lightly, despite my weight I eat lightly. Those are the things that I need to do. I need to do exercise and don’t do enough of it, and that’s usually because I just feel a bit tired worn out and that’s probably the heart. So the things interplay obviously; heart with kidneys with diabetes.

**Q: Does anyone want to mention anything else? So there’s been mentioned medication and diet and stuff like that?**

Tony: Well on medication I suppose being a diabetic you have to have a supply of jelly babies in case you get a hypo but I’ve been taking the steady doses of insulin now, I don’t think they’ve varied for probably five or six years. Dr DL every time I see her just says “Stick on the same dose”.

**Q: So other people in other places have mentioned things like good education, self-motivation, finances, good support from family and health professions and stuff like that. Do any of you guys identify with any of those factors?**

Tony: Well I’d say support from family yes, definitely. I suppose you don’t have to have diabetes or kidney problems to want the best financial support. I think we’ve already spoken about the fact that we all seem to have very good doctors.

Rowena: To me at home the problem managing of my blood glucose, yeah, that’s a big problem with me and my other pressure is medication. Also I need to control my diet because of my sugar level is so high and so I just measure my insulin as well. So I have a communication with Dr Hocking, every time she ring me, I increase the doses in the insulins. Just I need support from my...

**Q: From the professionals, yeah.**

Rowena: Yeah, my insulin…blood glucose yeah at home.

Bernard: The model I work on is really I’ve got to do it for myself.

**Q: So your own motivation?**

Bernard: Yeah and that I’m more likely to get a tongue lashing from my wife than support but that’s support in a way. So if my readings go up she’ll ask and so on.

**Q: That is support in some sense.**

Bernard: It’s a support yeah but it’s that kind of keeping me herded in type support, which I guess is important but other than that she always lays down the law “You have to look after your own diabetes. You have to look after your own health” and that sounds harsh but that’s what she’s like. She believes that very strongly. So it makes you somewhat independent or autonomous in treating it all. If you want to stay alive you do it yourself.

**Q: Other people also mentioned education or being educated as being an important factor.**

Bernard: A lot of it’s self education I’m sure.

Tony: That’s why you go to the diabetes educators here or look up the web.

Bernard: Like Tony, I think a lot of the GPs really don’t know very much about diabetes. You can tell by the way they talk that they’re proceeding on a lot of assumptions that you may have gone past yourself. Once you may have believed them but now you’ve learnt a bit more. I don’t want to sound like a know it all but it’s in our interests to find out and of course a specialist would know all about it but most of the GPs, the ones I’ve come across are not all that knowledgeable about diabetes.

**Q: Other people have also mentioned being able to accept the disease, that they have it and also a sense of hope or something to work towards. Do any of you identify with those factors?**

Tony: I accept I’ve got it and I know I’d probably get better control if I lost weight but I’ve always been this weight and people that know me personally and I say “They want me to lose weight” and they say “But you’ve always been that weight” and being on insulin doesn’t help to lose weight. So you do have these problems. My GP is in a very big medical practice, there’s about 18 doctors in this practice, and after I left the previous one to my present one I went and saw the practice manager who I know over the years, and she said “Leave it with me, I’ll talk to a couple of the nurses” and I’ve now got a very good GP and she is quite helpful with regard to diabetes. But it’s a matter of knowing what is a good educator in that regard because in this practice if one of these TV personality doctors and say “We’ve got whatever her name is, she’s on TV”, I said “No she’s bloody awful. She’s working off a script if you’re on TV…” I said “Give me Graham [Maloof] *0:58:31.5 who’s on TGB on a Sunday night for two hours where people ring him up blind and say ‘Doctor I’m suffering from this’ and he’ll give a diagnosis and he’ll say if he doesn’t know anything about it and suggest they go and see a specialist”. I said “That’s a good educator”. I don’t believe the bloke on Channel 7, whatever his name is or whatever because they worked for a week on a script, that’s not…you know. If you asked them a blind question, they probably wouldn’t know how to answer it. So I think there’s a lot in your doctor, whether they can get the message over to you.

**Q: Do you have anything you want to add Rowena? No? Okay, let’s move on. On the flipside, what things make it hard for you to look after your diabetes and chronic kidney disease well?**

Bernard: Cheesecake. All the nice food that’s around that you can’t eat but other people can eat, that makes it hard. Also it makes you aware at least that you know what you’ve got and what you can’t do.

Tony: Well I’ve learnt that like I take lantus in the morning and then novorapid during the day and I take 12 novorapid in the morning and supposed to be eight in the middle of the day and ten units in the evening but if we have our main meal in the middle of the day then I swap the eight and the then around and I’ve learnt to live with that. But then I like miso soup or something like that, I like to have that at night and I’ll wake up during…and it’s the only time I ever wake because I use a CPAP machine and I’ll just go out to it. Because of the fluid retention I take three lasix morning and evening and the doctors are horrified “You must be up all night” and I said “No I’m not “ because if I don’t use the CPAP machine I am up all night but if I have a hypo during the night that wakes me up good and proper. I get up and go out looking for the jelly babies and usually consume about five of them and wait half an hour, go back to bed and go back to sleep again. But I think your own education matters a lot.

Bernard: UI have a CPAP machine as well and I had Bell’s Palsy as well.

Tony: Seems we’ve got a lot in common.

**Q: Same doctors as well.**

Bernard: Yeah.

**Q: Rowena, do you want to add anything? So what makes it hard for you to look after your diabetes and chronic kidney disease or another way of phrasing this question is what are some barriers to looking after your diabetes or chronic kidney disease well? I think we’ve mentioned diet and the importance of education or knowing what to do.**

Rowena: For me just control about what I eat and my doctor has banned me drinking juice because I was restricted…yeah. I manage at home.

**Q: What I’m getting at is, for example for yourself Rowena you said earlier when you didn’t accept that you had the disease, that would’ve been a barrier.**

Rowena: Yes.

**Q: So other people mentioned things like having a lack of support or not having enough education or having a lot of other illnesses. Other people found having a lower mood or depression or anxiety made it hard for them. Do you guys identify with any of those factors or are there any other factors you guys want to bring up?**

Rowena: It’s very hard for me to manage because I have a depression issue and mentally you know because I don’t really accept that I have a disease. But I have a support from the hospital, from the educational educator people and it’s helped me about to accept it and to manage myself that I can do it for my own need.

Bernard: I was just thinking about that. I’m lucky, I don’t suffer from any depression or any mental things at all, I’m fairly flat and so I just take it all in my stride. Illnesses come along they come along, you just do your best to combat them and you go on for as long as you can. Then one day it will all end and I suppose that’s the way I think about it so I don’t worry about it one way or the other and I don’t look overly to other people to support me except I’ll come to my regular specialist meetings but I don’t contact doctors in between or anything like that. I take my own readings every day though several times, deep track of my blood sugar and I’ll take my blood pressure and I’ll know from that myself, I’ll just sort of diagnose from that myself what’s going on. Of course if something abnormal happened I would seek medical help but other than that it’s just a matter of managing it and getting on with it.

Tony: I tend to agree with Bernard in that regard. Associated with the diabetes, when I was seeing my heart specialist he was very concerned about my arrhythmic heart and I was seeing him every three months or so but it worked out with the vascular physician it all had to do with this peripheral diabetic neuropathy. My heart was changing its rate the whole time to get blood down into my legs and I’d go from 60 something per minute up to 130 a minute, just as if somebody had flipped a switch. Now for instance when I go to see Dr BC and the nurses are checking my blood pressure and my pulse changes they get very worried and I say “Forget about it, it’s nothing to worry about. Bruce knows all about it and it’s all got to do with my legs. My heart bloke only wants to see me two years”. I think a lot of problems to do with diabetes can be solved but it might take time. I’ve got a lot of problems that are traced back to my diabetes and over the years I’ve had diabetic ulcers on my legs and my medical practice was treating them and then Dr DL said “There’s a very good intensive podiatry clinic here for diabetes”.

**Q: Yeah, high risk…**

Tony: Yeah and she said “I suggest you see the chap, you’ll either like him or he won’t and he will either like you or he won’t and the two of them have to match”. So I saw Ian Reid, he treated me and my GP of the day said “Podiatrist, what would he know about leg ulcers?” and I said “Well if you’re in America a doctor isn’t allowed to treat diabetic ulcers, it’s purely in the scope of the podiatrist”, “What?” but he took great note of the dressings that were being used here and he and another doctor and the two nurses went off and did a modern dressing course and ever since then any diabetic ulcers I get are done at the practice, not back here. But you know, so again it is educating people on what to do and I know that with diabetic ulcers the dressings cost a lot of money and even though I’m a pensioner I’m quite happy to pay that rather than going through months and months of treatment and in the last three or four months I’ve taken to wearing stockings which I’ve got on now and I’ve noticed that all the little blisters I’ve had over my legs are disappearing and so on. But again, it’s just pure education and a lot of it is self education.

**Q: Thanks for that. Other people also mentioned conflicting information from different doctors.**

Bernard: No that’s what I said, I think sometimes the GPs don’t know. It’s not so much conflicting as they just don’t know. They’re keen to help you but they don’t know.

Rowena: Sometimes I get frustrate with my GP because sometime when I go to see her and discuss with her and she could not able to answer my questions. She’s sent me to my specialist doctor, you know, and just the conflict of sometime I’m not happy with her.

**Q: I’m just going to backtrack a bit, going back to some of the frustrating aspects. Some people mentioned in other focus groups as a frustration or weakness that there was often poor communication between the hospital and the GP or between different specialties within the hospital. Have any of you guys encountered that at all? Or transfer of investigations, so you might do an investigation for one doctor and then another doctor gives you the same investigations or they can’t find the results. So any of those sort of factors but it’s poor communication within disciplines, either about medical records or investigations and also between the hospital and the GP. Do any of you guys identify with that at all?**

Bernard: The thing I’ve found, sometimes when I come in, say I’ll go to see Dr BC and he won’t have my latest blood results even though I always do them a week in advance and that’s’ because hospital records haven’t you filed them on and then he will ring up and get a particular result. Doctor Ward will do the same, get a particular result. So that’s not so much a breakdown with the doctors however it’s a breakdown with the hospital records filing the things that come in that causes it. So what I do to get around that is if I go and see my GP before I come and see the specialist, I get a copy because I have a copy sent to the GP, get a copy, GP gives me a copy, I bring it along, I give it to Bruce to have a look at if he doesn’t have the results. So that way we work it out but that’s an inefficiency in the system.

**Q: Does anyone else identify with that?**

Tony: I don’t have that difficulty because my pathologist is Hanley Moir and they seem to communicate with Dr BC and others by the internet rather than sending a hard copy. I know in my own medical practice where my GP is, they get it over the internet also but I find it’s not so much with my renal or my endocrinologist but you see other specialists here which aren’t in the specialist consulting rooms but in their own private rooms, such as my cardiologist, they don’t have access to my hospital record. Even though they’re one floor above where Dr BC sees me, it’s foreign and they don’t communicate other than by their own letter about what’s happening. Similarly going back to the physiotherapist I saw two days ago, they’re not using my hospital file, they’ve got their own file and I said “But it’s all in my file”, “Oh we don’t use that”. These days with electronic records I thought it would be easy now for anybody to see in front of a terminal in a hospital and get my medical records. I’ve registered with Medicare and so on and I would imagine if you were in T City you should be able to access my private GP or hospital records because I’ve registered that I’m quite happy for that to happen. But there still seems to be a breakdown in communication between, well even within this hospital.

**Q: Rowena, have you got anything to add to what’s been said?**

Rowena: Well the problem is that because of the lack of communication with my GP, my specialist doctors are very good, I’m happy with them but my GP is not communicating with them. So my specialist doctor sending her a letter, she don’t read the letter until I come, it’s still out there somewhere and I ask her and then that’s it, she reads it to me and that’s why she cannot answer me what the doctor say in the letter.

**Q: Another complaint that’s been mentioned as well with the medical records is that sometimes the medical records may be hard for people in different specialists, just say you see one specialist and then you see another specialist, the other specialist doesn’t know what the other specialist has said because of medical records. Has anyone had similar issues or experienced that?**

Rowena: I experience that with my medical records because my GP, because I have so many specialist doctors and changing my medications and when I go to my GP and she still had the old record there that has already been changed and I get a problem with medication because the doses is different.

**Q: So where does that issue come from? Is that because the hospital isn’t sending it out to your GP or your GP…?**

Rowena: The hospital is sending to my GP but my GP is not really…

**Q: Putting it in her records.**

Rowena: Yes, yeah.

**Q: How about you guys?**

Bernard: I find that all three of the specialists I’ve mentioned all send written reports, letters to my GP and they go on file and my GP’s a bit of a secretive guy so I’ve got to raise the fact myself and then he’ll say “Oh yes, I got a letter from so on”, he usually doesn’t volunteer anything. But that’s a GP problem again I think. I don’t know what it is, whether it’s just they don’t have time or there’s not the same amount of education, I don’t know what it is. But that is an issue but I find the specialists that I deal with, first of all they have a common file on me which is the Hospital 3 file and that will go from my appointments from one place to the next.

**Q: And you find that’s always updated, they know what each…?**

Bernard: No, sometimes they leave off the latest bloods but other than that, they eventually get on and so it’s probably all there but it just sometimes takes a little bit of catching up with. That’s one reason why I make sure I don’t have appointments on the same day because that file has got to have time to move to the next one and so on. So I usually leave them about a week apart and do them on Mondays.

Tony: I carry my file between specialists if I have two on the same day. Many a time I’ve carried it from Dr BC to Dr DL.

Bernard: Well they trust you, they’re not supposed to do that.

Tony: I know but they do. The only problem I ever find is if a specialist prescribes a drug for you and they send off a letter to your GP about what they’ve done, it’s okay, it gets filed in the practice’s computer but then you want a doctor in an emergency and your GP isn’t available so you go to see another GP and you’ve asked for a script for such and such and they say “Why are you taking that, it’s old fashioned and you shouldn’t be taking that” because they haven’t had time to go through all the letters to see what it is. But that’s my only problem and I soon tell them that “No, it’s been prescribed by so and so”.

Bernard: That’s one issue, if you’re going to more than one specialist like that and they’re all fairly strong people, they will think nothing of changing medications and so you’ll be having three different doctors plus your GP possibly all changing your medications. So apart from it being expensive that you’ve got to take that or stop taking medications, you’re taking up the new ones but they aren’t always telling each other. That’s one issue.

**Q: Another issue that’s been raised and this probably is more in Victoria, I don’t know about here, is that some people are finding when they’re seeing many different specialists or many different specialties is that no one has a holistic view of everything and no one’s pulling everything together. So no one really knows what’s going on in the bigger picture, especially when they have multiple medical issues. Is that something that you guys have discovered or can relate to?**

Bernard: Well you’d need a physician wouldn’t you to do that properly, to coordinate everything?

**Q: I mean all of the specialists are physicians…**

Bernard: No, but I mean a specialist physician, what we used to call; that is somebody who is a specialist but he or she has got a very special knowledge of the whole body, not just… They may specialize in something but they also...

**Q: I mean it can be done by any specialty as long as they choose to coordinate the care and choose to know what’s going on for the whole thing. Can anyone identify with that?**

Bernard: Well Dr BC is pretty much like that.

**Q: For you?**

Bernard: Yeah.

Tony: I find that because you’ve got your one hospital file they know what’s going on but then if I go to my heart specialist one floor above Dr BC, he doesn’t know because he’s in the private consulting room, he doesn’t access my hospital file. But he does send reports to Bruce and Diana which get filed in my record but he mightn’t necessarily see reports from them. I think they do send them to him. Other than that I think they talk to each other.

Rowena: My GP has no access in hospital file and it’s very hard for her because she has no access.

**Q: Do you find there’s someone pulling everything together that knows everything about you, all the different medical issues?**

Rowena: It’s my complex care nurse. She knows everything and she forward to her.

**Q: Now let’s move on. In the last 15 minutes or so let’s move onto this question and this can be a bit of a fun one. Imagine you became the director of the hospital and you had the power to change things about the diabetes and chronic kidney disease health services. If we were going to design the perfect health service which would meet all your needs, what would that include? And I guess you might have something to add in this Bernard given that you used to be the vice chancellor or...?**

Bernard: That was different, hospitals are different. Well usually the general rule is that you pull things together as much as possible but yeah, I can see if you’ve got too many patients coming to the same place that creates a new set of problems. So there are a whole series of organisational models you can use and I think different hospitals probably have tried them all at some stage or other but they’ve all got positives and they’ve all got negatives.

**Q: So this is also judging from your experiences as well.**

Bernard: Well it comes down to the attitude of the people. If they want to be there to help you then it’s a good experience. If they don't, and I find a lot of the people on desks and so on, they couldn’t care, you can sit there all day and they’re not going to help you but when you finally get in the doctor will be very positive and helpful and so on. So maybe it’s a function of what their career structures are and how devoted they are to their job. But it’s about commitment and wanting to help people and so on.

Tony: It’s only in the last couple of days I’ve realised it; if you come to any of the outpatients here and the girl was about to ask me today when I went to the clinic one desk for my Medicare card. I couldn’t understand why it’s not recorded in the computer because it appears that all outpatient clinics require your Medicare card whereas when you go to Dr BC or when I saw Dr DL privately down there they know what your Medicare number is, they give you the form to sign and that’s it.

Bernard: Yeah, it’s in their computer.

Tony: It’s just a little irk I’ve got as to why I have to dig into my wallet to get my Medicare card out when no doubt it’s recorded in the computer. My only other criticism, if you’re looking at the hospital as a whole, these people need to get some better food because on Tuesday I had appointments down there at physiotherapy that went up until one o’clock starting at 9:40 and I my wife wasn’t home, she was down in Albury and I thought I might as well have my meal here and I walked right around. Their salads are dripping with mayonnaise and things like that or they’ve got greasy food and so on. There’s nothing there for people that would be on a diet.

**Q: For the record this is Zouki but anyway.**

Tony: Yeah Zouki. And when I’ve mentioned this to Dr BC and others about in the old hospital they used to have individual places and if you wanted a salad you could get a salad made up for you, here it’s all prepared and they said “Zouki’s got a good record, they do a lot of hospitals down in Victoria” and I said “But this is New South Wales”. I suppose it is looking at it as being a diabetic. On Tuesday they had a magnificent tray of pork belly, it looked fantastic but if you’re a diabetic do you want to eat pork belly?

Bernard: Yeah you want to but you can’t.

**Q: Trust me, some health professionals have bought that up about giving people mixed messages. You give them education then the hospital cafeteria serves stuff like that.**

Bernard: That’s right, they don't really understand that they’re part of... They deliver the same kind of fare that they would deliver if they were in a shopping centre.

**Q: Any other factors guys? Other people have mentioned stuff like good education, a combined renal diabetes clinic, cheaper parking, regular pharmacist contact.**

Tony: I think education generally definitely yes. Several years ago I had an aunt dying and it wasn’t until she was in the convalescent home at the retirement village they discovered she was a diabetic. Everybody knew she was going to die and they stopped her having chocolates and so on and my cousin who’s also a doctor hit the roof and told the hospital and the medicine staff “How dare you, let her have her pleasures in her last few days” and they said “But she’s a diabetic, she’s not meant to eat chocolates”, he said “So what, she’s dying”. So I think there has to be better education generally over the medical profession about diabetes.

Bernard: Yes. This is a little bit off but if you fly somewhere they say “Do you want a diabetic meal?”, they don't know what a diabetic meal is because they give you stuff that diabetes shouldn’t be eating. So there is a great need, the catering example, have the caterers better educated about what they can provide. It’s not just people like us, I mean the proportion of people now with diabetes in one form or another is enormous and obesity as you know is going through the roof and it’s all caused by these reasons.

**Q: So anything else guys? Some people talked about a one stop shop where they could see many disciplines under the one roof, diabetes, kidney, educator all in one go. Regular pharmacist contact. Any of those factors you guys would like if you were designing a new health service for diabetes and chronic kidney disease?**

Tony: I don't complain about parking because I have a disability parking permit but I do note that when I come here to do any clinical work like I would imagine these people drove, they would’ve been offered a parking permit for the parking station. I’m given taxi dockets, even though the bus stop’s right here at the door that I catch home I’m offered taxis if I go for a clinical. So I don't think parking is a problem with the North Shore other than if you’re a patient, yes you have to pay and I mean that happens anywhere.

Bernard: It’s a problem for me, the cost because I don't have any disability stickers or anything so I’ve got a card but to bring it down here and park it for the time necessary would just cost too much and then to go home in that traffic. So I come down and back by train.

Tony: Which only costs $2.50.

Bernard: $2.50, that’s right.

Rowena: I have no problem with the car park because I have my nurse come and pick me up and we park the hospital car park in the community centre so it’s easy. I think the most important thing is education.

Bernard: Well it’s a long process over many years and your condition gets worse and you take it more and more seriously and you seek more education.

**Q: So any other ideas about a perfect health service if you were designing a new one, if you were the director of the hospital? And you talk about people being committed to offer good healthcare, anything else?**

Bernard: I know too much about organisation to talk about perfect anything so...

**Q: True but just throw out some ideas, that’s what the purpose of this is.**

Bernard: Well there are all kinds of different models as I say for organising. Do you put the individual patient at the centre of the system, in other words similar to what you’ve got or do you just let the patients come along and make the doctors the centre of the system? Do you put them all in one place or do you decentralise them? Do you put expensive equipment...

**Q: This is from the patient point of view which is why we’re asking you guys.**

Bernard: No, you said if we were running it.

**Q: Yes, but from your experiences though as a patient.**

Tony: Well I wouldn’t change anything.

Bernard: Yeah, it’s pretty positive.

Tony: I mean couple of times I’ve had in the last 18 months I’ve had both cataracts done on my eyes and I had a carpal tunnel done a couple of years ago; being a diabetic I think is fantastic because you’re always first on the operating list. I think it’s a pass to get things done quickly.

Bernard: If you manage to look really bad you can get in on the aircraft first, you know.

Tony: Yeah.

**Q: How about this one stop shop that some people were mentioning about having everything at one place, the kidney doctor, having the educator there, stuff like that. Some people have mentioned that...**

Bernard: Yeah but it becomes a bit like an assembly line. That’s the trouble, everybody goes to the same place. That’s always the trouble with one stop shops.

Tony: But isn’t this hospital a one stop shop?

Bernard: Well in that broad sense it is, yeah.

Tony: I mean they can’t all be within one room can they?

Bernard: No but I think what we’re talking about...yeah.

**Q: So some patients have mentioned that a clinic where they can see the diabetes doctor, the kidney doctor at the same time and an educator at the same time rather than having to go up to the hospital multiple times. What do you guys think about that?**

Tony: If they were on time I think it’s a fantastic idea but usually I don't use outpatients that much but you hear people that do complain about waiting times. People ring up the radio regularly to complain that they’ve waited three or four outpatients to be seen by a doctor, even though you have an appointment.

Bernard: You’re a proponent of the one stop shop?

**Q: No, I’m just mentioning what other people have mentioned. What do you think, you look like you’ve got something to say?**

Bernard: I can see all the problems with that because I mean I worked on a royal commission once and one of the things we proposed was one stop shops for social welfare reasons and so on but no, it didn’t work.

**Q: What happened?**

Bernard: Too many people have come to the same place, queues form, your whole numbers. You mean well but you can’t synchronise it so that people can go from one to the other, it just doesn’t work like that. You end up being in a serious of queues. It would be nice. That’s what I meant by when I started off saying in different models you can have a patient centred model, individual patient centred where that individual patient has all the services gathered round it. it’s extremely expensive and no government’s ever going to support that financially so you’re forced to go to things like one stop shop which is the next stage down and as I say that’s full of problems too to do with queuing and whatever. And you end up going back to a decentralised model but on the same general site, in other words like a hospital here where people do just make their own appointments when it suits them and that works because it works from everybody’s perspective. It’s inconvenient for somebody but it’s going to be inconveniencing the process no matter which way you go so that inconvenience will always come down to the customer. When I was in my active working life and I had a pretty important job, I would come to the doctor and I would have to wait and sometimes I’d say “This is costing me more than that doctor’s being paid for me to wait this time”, while I might wait an hour, hour and a half and that’s expensive but nobody takes account of that whatsoever and I don't think I should be treated any differently from anybody else, that’s not the point that I’m making, it’s just that everybody has to wait and it can be very expensive.

**Q: Rowena, have you got anything you want to add further?**

Rowena: No.

**Q: Okay, before we close up, is there anything you’ve come wanting to say that you haven’t had a chance to say or anything I should’ve asked that I didn’t get to ask that you guys want to mention? Anything important you guys want to mention before we close up in terms of your experiences with diabetes and chronic kidney disease or about the health services?**

Bernard: Erectile dysfunction. That’s a problem that nobody sort of faces very much I think and if you get medication to try to help you never get it on the PBS. It’s always as if it’s a luxury product.

Tony: But again that’s an education... When I saw the urologist the other day he’s prescribed DUODART and he says “Are you sexually active?” and I said “I’m a diabetic”, “Oh”, so he wrote the script. That’s all I had to say. So it was obvious, even though he’d taken my history before and he knew I was a diabetic it didn’t even enter his head.

Bernard: Yeah, but they can’t do anything to help you or whether they can or not they don't. They’ll refer you to some commercial product that may or may not work that you have to pay full price for and even though it’s part of your condition, it’s not recognised as such. It’s seen as an optional extra.

**Q: Any other issues?**

Bernard: Over time that’s been the most important one for me I think, the way in which that’s been treated; very badly, and continues to be.

**Q: Sometimes some specialists don't even raise that, it depends how much time they’ve got.**

Bernard: No that’s right, they won’t, you’ll have to raise it with them. It’s the only thing that gets raised.

**Q: Because it’s quite private, yeah. I didn’t attempt that pun on words but anyway.**

Bernard: That’s alright. And if you do often they’ll do their best to get it off the table ASAP by as I say recommending something commercial or go somewhere else or something. But as I say it’s a genuine part of the condition and it affects something that’s important in life but there’s no sort of acknowledgement of that and there’s no follow through in terms of PBS medication. It’s all private, it’s all commercial. I got that one off my chest.

**Q: Any other issues people want to get off their chest before we close off? I think that’s a no. Okay.**

END OF TRANSCRIPTION

**Focus Group 8**

**Q: Okay, well welcome everyone. So this is a focus group or group discussion. So in essence we’ll be asking you a couple of questions and then we’ll discuss the answers in a group I guess. Now, there are no correct answers. All these questions are based on your experiences and your opinions and in essence, we want to see how we’re doing at the moment in terms of our diabetes chronic kidney disease services. Your experiences and how we can improve on them and I guess, you guys are the most qualified to help us with this, because of course, you’re using the system. So there’s no right answers, there’s no wrong answers either. Feel free to share your experiences and in no way will this – will what you say impact on the care that you provide. In fact, it will probably help to improve it if you’ve got anything, you know, anything to add, to comment on. So can we just quickly go around the room and just comment on how long you’ve been attending the diabetes and chronic kidney disease services for? Maybe we can with Lee in no particular order.**

Lee: Yeah. I was diagnosed in January last year.

**Q: Yep.**

And so I ended up in hospital, at Hospital 4a.

**Q: Yep. And sorry, could I also ask, before you say something maybe just say your name first and then - - -**

Lee: Yeah, my name is Lee.

**Q: Yep. Sorry.**

Lee: And I was diagnosed in January last year, since starting of January and actually I was admitted in Hospital 4a where they found out all my issues.

**Q: Yes.**

Lee: So since then I came out of the hospital, my diabetes was something like 10 or 12 at that time.

**Q: Yes.**

“Lee: And they put me on insulin straight away.

**Q: Yes.**

Lee: Now it’s somewhere around four to – four to seven, and I’m only taking it in the morning. I’m not taking it in the afternoon.

**Q: Okay. Yeah.**

Lee: So through the course of that I’d changed my diet, I’m – I’m regularly - - -

**Q: Yep. So, and how long have you been attending the diabetes and chronic kidney disease services for?**

Lee: At the hospital?

**Q: Yeah, at the hospital.**

Lee: It’d be three months. Once in three months.

**Q: Okay. So that’s the diabetes. What about the chronic kidney disease?**

Lee: Now I’ve just started doing that about – still my second session.

**Q: Okay. Yep. Yep. What about everyone else?**

Ian: Ten years.

**Q: Okay.**

Ian: I had major surgery 10 years ago, triple A, and my immune system went <sound effect>. So now I have kidney problems, I’ve got thyroid, I’ve got the whole box and dice.

**Q: Oh no. Okay. And sorry, and that’s Ian. Yep. And Maz?**

Maz: Yeah, I’ve got more than 10 years here.

**Q: Okay.**

MAz: And also I’ve got a bypass going down.

**Q: Okay. Yep. So you’ve been attending both clinics for over 10 years?**

Maz: Yeah, for both of them.

**Q: Okay. And is that the same with you Ian, both clinics for – yep.**

Angeline: I’ve had diabetes for about 20 years.

**Q: Oh wow. Okay.**

Angeline: I attend diabetic clinic at Hospital 4a and – sorry, my name is Angeline. I attend diabetic clinic at Hospital 4a and kidney and renal clinic here in Hospital 4. Renal clinic has been about three years and Hospital 4a has been about five years. I take insulin four times a day, Novorapid, eight, 12 and 20 in the night and Lantus, 34 in the evening before I go to sleep.

**Q: Okay. Yep.**

Angeline: Other than that I have nothing else to report.

**Q: Well it sounds like you guys have been living with diabetes for quite a long time and chronic kidney disease perhaps, I mean, still a while but perhaps a little bit less, but not as long as the diabetes. Now, you know, we’ve been talking a bit about how long we’ve had these conditions for. I’d like you to think about your experiences living with both of these conditions, both the diabetes and chronic kidney disease, and from your experiences in your opinion, what do you think you need in place to look after your diabetes and chronic kidney disease well?**

Ian: More regularity in my appointments.

**Q: Sorry?**

Ian: Yeah, more regularity in my appointments. For example, in theory I come in every six months. That six months became 11 months, 10 months and I wound up in hospital with fluid in my lungs. If I had the six month appointments it would have been picked up.

**Q: So I’m just wondering whether we should move. We’re quite noisy here. I’m having a bit of trouble. I wonder whether we should try and move rooms.**

It’s because there’s a - - -

**Q: Yeah. Did you – should we try and move rooms? Like we’ve got this room here haven’t we?**

Yeah. Yeah.

**Q: Yeah. We might be a bit easier, because it’s – there’s this noise, so I apologise. If anyone wants to grab some sandwiches before we move.**

I’ll bring them in.

**Q: Yeah, okay, Stella will bring them in. I’m sorry to do this to you but I think it’s getting quite hard to hear everyone. I think it is a bit less.**

It is quieter in here.

**Q: It is quieter isn’t it? Yeah.**

Yeah.

**Q: Okay. Just all grab a seat. Someone can join me on this side if you want. So we were talking about what we needed in place to look after diabetes and chronic kidney disease. I think Ian mentioned, is it more regular appointments or getting to the appointments more regularly or …**

Ian: Well, it used to be six months. Recently it’s become 10 months, 11 months. Because last time, as I said, I wound up in hospital four days, fluid on my lungs, which would have been picked up had I had an appointment three months before.

**Q: Okay. Yep. Yep.**

Ian: So regularity is, you know.

**Q: Yep. Okay. So Ian said regularity. Anyone else got any other things that they think need to be in place for them to look after their diabetes and chronic kidney disease well?**

Maz: No. I think in – in my case, I think it’s already been - - -

**Q: I’m sorry, and that’s Maz. Sorry, that was Ian speaking before and Maz speaking.**

Maz: Yeah.

**Q: I know it’s very hard to remember to say your name, but if – yeah.**

Maz: I’ll try and remember.

**Q: Yes. Sorry Maz. Maz, yes.**

Maz: Okay. I think in my case, everything pretty is quite casual for myself, because sometime if they don’t stand up – I have to stand up on them. So I think mine is quite schedule as per plan. So I – I think I have – I can’t comment on anything on the service for now.

**Q: Yep. But is there anything that, like is there – what things need to be in place for you to look after well? So some people have mentioned, you know, good support from the hospital or regular appointments or education or supportive family or motivation and things like that.**

Maz: Yeah, in my case I get it all that. I get it all.

**Q: Yes. So everything I’ve mentioned, you’ve got it all.**

Maz: Yeah. I think I’m the lucky one probably.

**Q: Okay.**

Maz: Sorry, yeah.

Ian: Don’t worry about it.

**Q: Anyone else got anything they want to add or comment on?**

Lee: I think, yeah, for working people like me, the appointments sometimes a bit difficult like in the middle of the day. That’s a bit hard for us, because as you know, most of the employers won’t let you go. If they let you go you have to have a day off, sort of day off and so – so most of the time I ask for appointment late in the afternoon and sometimes if you – you cannot get them. So that’s an issue for me. Other than that, I think as far as education and support, I think some of it’s alright. Got no issues with that. That’s – that’s my main concern.

**Q: Okay. And Angeline?**

Angeline: I really don’t have much to say. I’ve lived with it for 20 years. The hospital appointments are okay. I think – what can I say. I find having to take insulin four times a day, sometimes I don’t take it in the morning, right because I don’t have time to have breakfast in the morning so I don’t take my breakfast shot, right, and my readings are quite low without taking the shot, you know. So – but with hospital appointments, they’re quite good, you know. I have no complaints about them, but some – sometimes you can go and stay for quite a while before they see you, you know, could - - -

Ian: Usually <laughs> (Maz and Lee laughing to).

Angeline: - - - could speed it up a bit. I don’t know, with one patient they might take five minutes, with another patient they might take half an hour, you know. I – I can’t understand why because we’re all there for the same thing and that’s about the only thing, you know, it’s – it’s the waiting time sometimes. (Lee nods) It’s quite, you know. I have this idea that they think because we don’t go to work we can hang around and have nothing else to do, you know <laughs>(Lee nods). So, but other than that everything is fine, you know.

Ian: Just Ian.

**Q: Yes.**

Ian: On the waiting time, the reason for that is that they make a number of appointments for two o’clock, a number of appointments for 2:30 et cetera and the result is that you – the doctors are back there from lunch or whatever, you end up then having to three, four o’clock.

**Q: So we’ve talked about some weaknesses of the diabetes and chronic kidney disease services. Any other weaknesses that come to mind besides the waiting times?**

Angeline: No. I can’t think of anything.

Maz: Yeah.

Ian: Well, should always be seen by a doctor. Should always be seen by a doctor.

**Q: Yep.**

Ian: This last visit at renal, was actually seen by a nurse because they were one doctor short. That shouldn’t happen. Admittedly it was a straightforward appointment, but you don’t know.

**Q: Anyone else got any other – Angelina, Lee, about weaknesses, things that could be improved?**

Lee: Yeah, there’s some of – there’s a few occasions where I have requested that all my results be forwarded to my GP because he’s – I see him on a regular basis.

**Q: Yes.**

Lee: Like every three months or six months or whenever I want to go and see him, and that didn’t happen, so a bit disappointed from that point of view.

**Q: Sure. Sure.**

Lee: Because I - - -

Angie: I have to support that. It’s Angeline speaking here. I have to support that. My GP hardly ever gets any reports about diabetes or renal clinic, you know, so – and I see him on a regular basis because I go to him for my pressure, you know, and when I ask him have you had results of I went to renal clinic, have you had any – any results from that, he says no, you know. So I would – I would like my GP to be informed a little bit more.

**Q: So it sounds what you – what you guys are saying is a lack of communication - - -**

Ian: Communication.

Yeah.

**Q: - - - between the hospital and the general practitioner. Yeah.**

Angeline: Yeah.

**Q: Does anyone else identify with that as well?**

Ian: Yeah. (lee nods)

**Q: Yeah, Ian. Yep.**

Maz: I don’t have that problem.

**Q: You haven’t got that problem. <laughs>.**

Ian: He doesn’t have any problems.

**Q: Okay. I mean, other people – other weaknesses some other people have mentioned including – well some people have mentioned poor communication between the hospital and the patient, or not enough education, or too many appointments, or even conflicting advice, different doctors saying different things. Do any of you guys identify with any of those?**

Angeline: Angeline here. I think you could have a little bit more – I find diabetes complicated, right, and I think educating people on that, because I get terribly confused sometimes. For instance, right, I’ll give you – I go to bed – before I go to bed I test my blood and it’ll be 10. I take my 34 shot of Lantus, right, and some mornings when I get up it’ll be five, right, which is good, okay. Yet, the next day I might go – before I go to bed I take my – test my blood and it will be 11 and when I get up in the morning it will be 13 and I can’t understand why, you know. After I’ve taken my shot of Lantus why it should be – should be higher. I can’t – I find it terribly confusing and then some days when this – when this happen I think to myself, I’d like to take all my insulin, and I did it once many, many years ago, right, I mean, many, many years ago, I took the whole lot and I threw it down the toilet, because I was so absolutely fed up with it. Since – you know, and I – and I find it, as I said, maybe those people who have diabetes should be educated a little bit more as to the complications with – with diabetes.

Ian: Yeah. Being an engineer, being a logical sort of person, I ask a lot of questions. I think I know more about my condition, particularly the heart side of it, than even some of the doctors here. And in fact, I teach them how to take my blood pressure, that’s how bad it is. But on the business of what you were just saying, a lot of it is not the insulin part, it’s the diet. For example, I’m taking – looking at my situation rather than yours, but I go out to dinner with my son, for example. If we go to Japanese restaurant my blood pressure the next morning will be four and a half, five. However much blood sugar I should say. If I go Chinese, it’ll be 11, 12, 13. So it’s not the condition, it’s what you do with it and if you learn you’ve got the diet side of it as well as the – I don’t have insulin problems, but I think you’ll find that that will be part of it.

Angeline: I eat salad every night, I’m so fed up. I feel like a goat sometimes. I eat - - -

Ian: I’m not criticising you, but just - - -

Angeline: No, no, I’m just telling you, most night – most nights it’s fish salad, otherwise it will be a caesar salad, right, but it’s a salad.

Ian: Any sauces?

Angeline: Any sauces? No sauces. No - - -

Ian: In that case, ask your doctor.

Angeline: No, no, no, because I was told not to – not to eat them and I have to have a salt free diet too, you know. And – and I find – I find it terribly confusing, you know. I – I – when my – when my blood sugars are high I know I mucked around and I write it down in my diary, right, then I - - -

Ian: That’s the time to ask the questions.

Angeline: Yeah. I write it down in my diary, I went out to lunch and I – if I go out to lunch then I have dessert, right, so then I know my blood sugar’s going to be high, so I write it down in the diary, right. But on days when I don’t muck around and – and these things happen, I find it very confusing.

**Q: So, beside the education, has anyone else got any other weaknesses? I think, as I said before, you know, some people mentioned poor communication with patients, or patients feeling like they’re a number, or some doctors just looking at a particular – either the kidney or the diabetes, but not looking at the whole patient. Does anyone identify with any of those?**

Lee: Yeah. Now, I mean, I find it, like Ian said, I ask a lot of questions when I’m around.

**Q: Yes.**

Lee: So I’m pretty good with my diet and – and I get the results as well, so – and then if the question has been asked, I’m pretty, you know, the answer be truthfully and they give you a lot of information. So I didn’t – I didn’t have any issues with that.

**Q: Yep.**

Angeline: I’d like to know side-effects of kidney disease, because of late I’ve had a few odd things happen to me and I don’t know whether it’s the kidneys or the – the diabetes.

**Q: Sure. Sure. Look, maybe we can talk about that after the focus group. Definitely ask your doctor, but I’ll – we can talk about that after we finish this whole group. So remind me once we finish. We can talk about that. Maz, have you got anything you wanted to add or …**

Maz: I used to have your kind of problem. Some doctor say you can eat whatever you want, but in moderation, but I tried moderation but it doesn’t work. It still doesn’t work.

Ian: What’s moderation?

Lee: Yeah.

**Q: So has anyone got any other weaknesses they wanted to add? Any of the ones that I mentioned that anyone can identify with or …**

Ian: Well just on the medication side of it, education is probably a large part of it, but I find that if I see my heart specialist, or my GP, or the renal clinic or whatever, every time I go they’ll change one of my medications up, back, up, back, because I take a lot of medication, and it gets a bit silly, because one’s contradicting the other.

**Ian: Q: Oh as in – do they tell you why they’re doing it, or …**

I tried to get the question. They’ve always got some logical answer, but the next guy you see you’ve got the opposite answer. Communication with medics is a bit of a problem.

**Q: So do you think it’s a problem with communication between the doctors or …**

Ian: Yes, definitely. I was here on Saturday for the physicians exams.

**Q: Yes.**

Ian: And well I mentioned my blood pressure before, and four doctors who are trying to become specialists, each one of them, my blood pressure rate’s 130 to 170 according to them, because they can’t do it properly, and yet they’re qualified doctors. So I think it’s more than the education of the patients, sometimes the education of the doctors too.

**Q: Sure, sure. Anyone else got anything else they wanted to add before we move on? No. Similar sort of question. What do you guys find frustrating about the current diabetes and kidney health care that you receive?**

Angeline: No two doctors tell you the same thing. One doctor will tell you one thing and another doctor, you go and tell him the same thing and he’ll tell you another thing, and they never, never seem to work on the same scale.

**Q: Does anyone else identify with that?**

Ian: Well that's what I said a minute ago.

Lee: Well me – Lee to some extent on that one, because I have – first of all, if you go for appointment you never see the same doctor. It’s always a different one. I’m pretty sure they look at the file before they come and talk to you about it and I don’t know how much time they have because they look at the screen. I said, “No, I have an issue.” So sometimes you have to kind of push your issues to them and like, I went my – not the last, but the appointment before that, and the doctor she was telling me, “You should not be on insulin anymore”, because I get, what do you call it, hypos.

**Q: Yes.**

Which is you know, I go to the – I went swimming one day and the guys had to pull me out of the swimming pool, really. In a flick of a second I was gone. So that was a bit dangerous for me. And then I went to the appointment and they said, “Oh okay we’ll pull you out of insulin because we see it only goes from four to eight.” Maximum is eight. There’s no point. In the morning it’s about four to six, somewhere there, you know. Then I went to the last one expecting me to come out of insulin and when I was talking to this – another doctor and then she said, “No, no, we’ll keep you on for another three months.” My question was to her is, so if I see another doctor next time it will be another three months and another three months. I’m getting scared when I get that low sugar, because I think low sugar to me is no good and if – when I go to the gym I swallow about half a dozen lollies while I’m on the treadmill, while I’m doing a bit of weights. I know that it’s affecting my lifestyle, which I’m taking – they should rectify and they tell me, okay, hang on, you know, “You don’t take those shots. Here’s a tablet you take when it’s low or high or whatever”, you know. So I agree to some extent on that one.

**Q: And how does that make everyone feel when they get this different advice from different doctors?**

Ian: Well that’s when I started asking more questions.

Angeline: Yeah, it’s frustrating. <laughs>.

Lee: Because the appointment is only for 15 minutes. I mean, doesn’t matter how long you want to stay and ask questions, they will wrap it up, because there’s another 20 people still waiting who are getting frustrated.

**Q: Any other weaknesses, or any other frustrating factors besides the conflicting advice? Some people found waiting times an issue, either in a clinic or getting to – or even getting an appointment.**

Ian: All of the above.

Lee: Waiting time is an issue, that is sometimes you have a good day, sometimes you are there for hours.

**Q: Any other frustrating factors that people want to mention?**

Maz: No.

Ang: I can’t think of any.

**Q: Okay. Is it easy for people to get to the diabetes and kidney health care that they need? Is it easy to get the diabetes and health – diabetes and kidney health that – that health care that you need? Like, is it close, is the locality good, is it affordable, are the waiting times okay? I think well, we’ve spoken about the waiting times.**

Ian: Yeah, we have already.

**Q: What about in terms of localities and cost?**

Angeline: You know that parking here is horrific. (Ian agrees and says yeah) I’m not telling you a word of a lie, between today and Wednesday, we will be spending $30 on parking, and that – and that’s the – and that’s the concession rate, you know. It – it’s – it’s horrific that pensioners have to pay that much, that amount of money. Forget about pensioners, anybody having to spend that amount of money, you know, and – and you have to come. There’s no way you can say, well no I don’t want to – oh well you might say, “But I can’t make the appointment this week”, but you’ll have to come next week, so you’re still – parking here I find very expensive and there’s no off road parking either, unless you walk for miles.

Ian: Parking’s a problem. You would know that. You guys all pay for parking don’t you? You don’t get anything free.

**Q: No.**

Ian: See, I ask questions about everything. <laughs>.

**Q: Anyone else got comments? Maz, Lee?**

Maz: I think that the clinics for the diabetes only in the hospital, the main hospital, like Hospital 4 and Hospital 4a. What about like myself, I live in Cranbourne. I need to go to Hospital 4a, which is not so bad, but I would prefer to be nearer than my place. See like, I think we have a Cranbourne integrated or something, but they don’t have that kind of service there. We need to go to the hospital.

**Q: Anyone else got other comments?**

Lee: Lee. I – I totally agree with Hospital 4 parking that kills and last time I came I paid $40, and that’s out of our own pocket.

Ian: Try being in here for four days with a car in the car park.

Lee: Yeah. And then sometimes you get the appointments just like 8:30 in the morning, you know, and then you drive – you’re on the road for a couple of hours.

Angeline: And disabled parking is – they have four spots of disabled parking out there, which is permanently used, right, and – and if you want disabled parking it’s inside the – inside the parking building, right, but you have to pay for that, you know. And I think – and I think they should have – there’s taxi ranks all – all on one side and just four spots for disabled parking. There should be a lot more, you know. I have a spinal condition. I slipped a disc and pinched a nerve and I can’t – I can’t walk. There are some days I just can’t walk, you know. I find it quite unbearable to walk the pain is so intense and parking I find is horrific here.

**Q: Any other weaknesses or frustrating factors that people want to mention before we move on? No. No. Ian’s saying no, Lee’s saying no (lee laughs). Maz or Angeline?**

Maz and Angeline: No.

**Q: No. Okay. Let’s shift tack a bit, but similar sort of topic. We’ve spoken about some of the weaknesses. Based on your experiences, what would you say are the strengths of the diabetes and chronic kidney disease health services?**

<Ian laughs>.

Angeline: I’m a bit stuck there.

Ian: Silence.

Lee: They know all the weakness and everything. I mean, seriously, if you go to the hospital and the staff are good, people are willing to help you if you want help. I have noticed that. One time I tried to shift my appointment at least 15 minutes earlier and they would be generous about that I think too. So caring about patients is good. No doubt about that. They do care. They do care.

Angeline: Yeah. I have to say that when you come – when you go to diabetic clinic or renal clinic, you get good service. They’re – they’re prepared to take time and tell you exactly what’s wrong with you and – and whereas you go to your doctor, he’s not equipped to deal with diabetes or to deal with chronic kidney disease. He’s a – as it says, a general practitioner, right. So you get very good service here, you know, here or Hospital 4a where I go for – for diabetes, you know. Jennifer Wong is fantastic. She’s really good, you know, and so are the other doctors there in the diabetic clinic, you know.

Ian: Actually I’ve found their worse. My GP’s better.

Angeline: Oh do you?

Ian: Yes. For example, I have a thyroid as well as the – and all the time I was coming to the renal clinic they’ve never picked up the connection. My GP recently did a blood test, a thyroid blood test and he matched the thyroxine with the – my thyroid problem, with the kidneys, and put me on B12 injections to give me some more energy, which they wouldn’t have even thought of at the renal clinic. So that’s the opposite.

**Q: Yep. Yep.**

Ian: Good GP in other words.

**Q: Ok, yep, Sorry Ian, just for the purposes of the record could I get you to speak up a bit? I apologise.**

Ian: Oh all right.

**Q: And Maz, anything you wanted to add or agree, disagree?**

Maz: No, it is – it’s pretty good actually the service here. Yeah, just a phone call away, everything, yeah.

**Q: Okay. Related question. What do you guys and girls find effective about the services that you experience for your diabetes and chronic kidney disease?**

Ian: Effective is the knowledge that they’re there if I need them. Sorry I didn’t speak up.

**Q: Yeah. No, no, that’s good. Yep. Anyone else?**

Angeline: That’s right, you know. You know that they’re there when you need them, you know.

**Q: Maz or Lee?**

Maz: Nothing’s wrong there.

Lee: yeah

**Q: Yep. Okay. What impact has attending the diabetes and chronic kidney disease services made on your health?**

Maz: I feel like I’m much more better educated. Yeah, so I know what I should eat and what shouldn’t eat, and what is good for me and what is not, but – no but sometimes I do go overboard, but as my educator said, “Yes you can go overboard, only for the weekend”, which is understandable, you know.

**Q: Yes. You do have to live. Okay.**

Lee: Lee. When I was admitted in the hospital regarding this I remember this diabetic person came. She was just like passing information regards to more about diabetes and all of this. I took her thoughts on board and – and I almost - because she told me I shouldn’t be here and I said, “Why? Why you say that?” and she said, “Because look at you. You don’t look sick. You don’t look anything else.” Go and do some exercise, eat the right food and get – don’t go to insulin, and that piece of information, you know, stuck into me and I did the regular – I changed my diet, I put it upside down and – and doing regular exercise. I’m telling you I really happy with her.

**Q: Wow, that’s great.**

Lee: I should thank her. If I meet her ever again I will thank her.

**Q: Yeah. So there’s a big impact then isn’t there?**

Lee: It’s a very big impact because of what she said to me and I thought about it, you know, took it on board.

Ian: The question is not what it’s done for me, what would I be like if I didn’t have it.

**Q: Yes.**

Ian: So I don’t know really.

**Q: Angeline or Ian, did you want to make any further comments?**

Angeline: I – I – I find in my situation I can’t exercise, right, and they come up with suggestions, but – and I’m a pensioner. I’m a – at the present moment I’m on disability pension, right, and they come up with suggestions that cost money and you can’t do it when you’re on a pension. You simply can’t do it. You can’t. I certainly can’t afford it, you know. They – I don’t know whether they think money grows on trees or whatever, but <laughs> I can’t afford to do the things they tell me that I should do, you know. I – as I said, my diet is – once a week I muck around. Once a week, especially on a Saturday. I – I – I don’t care what happens, my – if you check my diary, a Saturday reading is diabolical, right, and I know I’ve mucked around and I write it down, right, and – but I – I eat the right foods and I try to keep – keep everything in check by my diet, you know, but it doesn’t always work. I would like to be – I’d like to get off insulin. I did once, right, and I lost – and I lost weight and I thought this was great, but then my diabetes was, you know, and I find it – those are the frustrating things.

**Q: Okay. Now we’ve mentioned a couple of weaknesses and frustrating things, like conflicting advice or poor communication with your general practitioner.**

Angeline: Yeah.

**Q: Or parking, or in some instances a lack of education. What – have you guys got any suggestions on how we can address these factors?**

Angeline: It would be nice if you – when you came to clinic you saw the same doctor all the time.

Maz: Is it possible?

Ian: No. <laughs>.

**Q: I’m not sure about that, but anyway, we’re hearing you guys out which is why we’re doing this.**

Angeline: It would be nice because I – I – I – with my diabetes I used to go and see – I used to go private, right and then it complicate – then …

**Q: Don’t worry. Keep on going. That’s fine.**

Angeline: Then it – then it became expensive, so my specialist put me onto the diabetic clinic, you know, but there when you go, as with the renal clinic, you see different doctors every time and it’s as though they don’t know. They read your notes and they work on the notes, you know. It would be lovely if you could see the same doctor every time you went. Well I think it would be nice anyway. <laughs>.

**Q: What do other people think, or any other - - -**

Ian: It doesn’t worry me actually.

**Q: Or any other – has anyone got any suggestions on how we can improve some of the weaknesses and frustrating aspects that we talked about?**

Lee: Lee speaking. As long as, you know, all the doctors tell you something that, you know, you’ve got – not changing, you know, every time you go. Sometimes tells you totally different things to the other doctors. That’s for a patient, for us. Sometimes we go to the clinic and say, “Hang on, who should I listen to, the first one or the second one or the third one or do I do my things on my own then”, you know. So if they can clarify, as I said, they can come almost close to what you want to hear every time that would be good, not like okay, one doctor will say you do this, and then another one, “Don’t worry about it.” Like he says, “You eat in moderation.” What is moderation? You know, you eat whatever you want but eat in moderation. Some say, “No, you don’t eat this because it’s no good for you.” So, you know, it’s a confusing part of it. So if they have – they come and tell us what’s almost close to the same thing I think that would be nice.

**Q: Anyone else got anything they – Ian, or Maz, or …**

Maz: No.

**Q: No. Okay. Let’s shift tack a bit. So we’ve been talking a lot about the services. Now I just want to, I guess moving onto your own experiences and looking after the diabetes and chronic kidney disease yourself. Okay. Now we’re just thinking about that and your own management. In your opinion, what do you guys think that you need in place for you to look after your diabetes and chronic kidney disease well?**

Ian: Well I think I’m looking after it pretty well at the moment, so they haven’t said that I need anything else.

**Q: Yeah, yeah. But what things have you got in place which help you to look after it well?**

Ian: Just my brain that’s all.

**Q: Yourself. So for you it’s all down to yourself, motivation, your own - - -**

Ian: Well, let’s put it this way, I wouldn’t be here. I actually died 10 years ago, twice on the table. I wouldn’t be here if I didn’t have fairly rigid self control.

**Q: Okay. Yep. Yep. And what do – so for you, the support from the hospital and stuff like that is - - -**

Ian: It’s backup.

**Q: Is backup. Okay. So for yourself, the primary thing is your own motivation. What about for other people?**

Lee: Lee speaking. I think the family members have to be educated about this as well because it’s very important. When I have this issue, my older daughter was doing health as well, so she came on board and she started dictating my life. So, you know, if I tell her, you know, sometimes it doesn’t work. She decides what to cook, what shall – I should be eating and what time I should be eating and, you know, all these things. So – which is a great help, you know. If the family members are not – because what they cook, that’s what you eat. And normally if there’s four or five in the family but they don’t really cook what you like, they cook what they like too, so once your tastebud taste something good, you just keep going. It is very important.

Ian: You’re very fortunate. I have to do it myself. (everyone laughs)

Lee: But that was I think, for me, my family members where I am today is because of them. Because I used to go and eat outside and they all stopped it . I was frustrated, but I think after a couple of months I got so used to and I always eat at home now. So that all helps.

**Q: What about other people? Sorry Angeline, you looked like you were about to say something.**

Angeline: No. I – I think when family members know your situation and know about what you’re suffering from, you know, it’s very helpful, you know. We have – our eldest son is – he’s a health fanatic <laughs>, right, so he’s very, very careful about what we eat and especially for my husband, you know, who’s got a heart condition and had a stroke and – and he’s now got kidney dysfunction and – and diabetes. He’s got the works. <laughs>. So, you know, he’s – he’s very good as far as our food is concerned and things like that. So family members knowing about your condition is very helpful.

**Q: Maz?**

Maz: It is all up to me now, yeah. If I want it to be better, it’s up to me. You want it bad, it’s up to me.

**Q: Okay. So the family support isn’t that important for you, or even the support that the hospital provides isn’t that important. It’s more - - -**

Maz: It is important because the hospital really give me the education that I need to do what I need to do. So you say you want to increase it, you increase yourself, depending on your condition. So it’s up to me now.

**Q: But obviously you did need the – at least needed the education there.**

Maz: Definitely we need the hospital.

Ian: Oh yes. Oh yes.

**Q: That’s support where the education – is that correct? Does everyone agree with that?**

Ian: Yes.

Lee: Yes.

Angeline: Yeah.

(Maz also agrees)

**Q: Okay. I mean, other factors people mentioned include things like motivation I guess and support from family and friends, I think we’ve mentioned that, and even being able to afford things and things like that.**

Ian: It’s a big thing on a pension. <laughs>.

**Q: So are all of these factors in place for everyone?**

Maz: In my case, yes.

Lee: Yeah, more or less, yes.

Angeline: Yes.

**Q: Okay. Similar sort of question, changing tack a bit. What things make it hard for you to look after your diabetes and chronic kidney disease well?**

Ian: I just get lazy sometimes <laughs>.

Lorraine: Sorry, I didn’t hear what you said.

Ian: I get lazy.

Lorraine: Oh lazy.

Maz: I think it’s the food we eat. That’s the only thing. Being conscious of food.

Lee: I think it comes to the weekends, you know, because you - - -

Maz: Yeah, that’s right.

Lee: - sort of drift away a little bit, especially - - -

Maz: And then we – of course when sometime you come and visit old friends, we don’t expect them to cater for us, you know. See, if you don’t eat, you feel bad for them. So it’s very hard, very hard, very, very hard.

**Q: I guess unless your friends understand.**

Maz: They understand, but that’s what I said, they cannot cook for us.

Lee: Like it’s the culture, you know, like - - -

**Q: Yeah, I know, yeah.**

Lee: When you go and it – my cousins just expect if you don’t – if they offer a plate of food and you don’t eat, it’s a sign of disrespect and then you get carried away. And then normally I go home and I just jump on my treadmill. (C laughs) But that part they don’t know.

**Q: Yes.**

Angeline: Yeah, I find the weekends are the worst. Oh God. During the week I’m as good as anything, but when it comes to a Saturday and sometimes on a Sunday, Lord, it’s – it’s very frustrating.

**Q: Why is that? Is that because you’re out with friends or do you - - -**

Angeline: No. It’s because at the weekends we normally socialise, right, because everybody else is working and we don’t work, you know, and – and when you’re socialising it’s hard not to take part, you know. <everyone laughs>.

**Q: Yes, of course.**

Angeline: You feel as though you’re offending whoever it is you’re socialising with, right. (Maz and Lee nods)

Ian: Yes. That’s a cultural thing as you said.

Lee: Yeah, it’s the culture.

Angeline: Yeah, you know, and as I said, at the weekends, especially on a Saturday I just – I think to myself, blow it, because you’ve been good the whole of the week – whole of the week, why can’t you be bad on a day – one day a week. (all laughs)

**Q: Okay. Now besides the weekends and I guess the cultural and social factors, is there anything else which makes it hard for you to look after your diabetes and chronic kidney disease well? And some other people have mentioned things like conflicting information, or lack of support from the hospital, or lowered mood, or being very ill, or having a lot of other things going on, things like that. Any other factors which may - - -**

Ian: Not for me. (Ian shakes his head, and so does Lee)

Angeline: I find taking insulin, right, when I’ve started taking insulin I started putting on weight and I – and I really, really got frustrated and I’ve never been able to lose the weight I’ve put on ever since I’ve been on insulin, you know, and the dosages haven’t been less. They’ve always increased, and that’s a thing I find very frustrating.

**Q: Is there anything else anyone else wants to add in terms of barriers which make it hard for you to look after your diabetes and chronic kidney disease?**

Lee: No, not anything.

Ian: Just got to make the effort that’s all.

**Q: My next question was what do you think you – could be done to improve the situation?**

Angeline and Ian laughs

Angeline: As far as I can say it, don’t take it. Don’t take the insulin. <laughs>.

Maz: Still I think still it’s up to us. All that kind of question, it’s still up to us, you know. There’s nothing of the – the clinic or the hospital can do about that.

Lee: I think a major part of us, its discipline you know. That’s what it is. If you do the right thing, eat the right food, I’m pretty sure you can do better. You can do better. In my case, I’m almost out of it, so like I’m pretty sure in the next six months, I am so confident that I won’t be on insulin.

**Q: Wow, that’s awesome. That’s good.**

Ian: The self discipline is the most important, yeah.

Lee: It’s a very – yeah, see I’ve got my routine, I’ve got my gym time, I’ve got my swimming time in which I’ve never changed for no one. I don’t care who – where I have to go, who comes to my place, it’s – in that time I don’t give appointment to anything else but that, you know, it’s a discipline. It’s more of a discipline.

Ian: I used to be a cordon bleu chef by hobby.

**Q: Sorry, you used to be a?**

Ian: A cordon bleu chef. I did all the courses and everything.

**Q: Oh wow, yes.**

Ian: Today I eat very, very plain foods and it annoys hell out of me, but I’ve got to keep the discipline.

**Q: Yes. Yes. Anyone – anything else anyone – anything else anyone else wants to add? Quite a few elses in that question.**

No. (from all)

**Q: No. Okay. Here’s a different one, different question. So have any of you – so have any of you guys ever missed out on a diabetes or chronic kidney disease appointment and what was the reason for missing it?**

Ian: No.

Lee: I did once. I had to travel interstate and I got a calendar – you want me to wait?

**Q: No, it’s okay.**

Lee: Well I got a calendar where my daughter writes all of my appointments. As soon as I get a letter she will – I tell her to write it down, and one of those appointments I forgot about it. I didn’t ring and tell that I would miss it. And when I came back I received a very nasty letter saying you missed your appointment and that you have to go - - -

**Q: Sorry. Sorry guys. Just getting noisy. Okay, yep.**

Lee: So I have to reschedule my appointment and ring this number and all this.

**Q: Yep, so because – that was because of work. How about anyone else who - - -**

Angeline: Yeah, I missed a diabetic clinic appointment once because I – it was when – it was when I fell – not fell over, I don’t know what I did to slip the disc, right.

**Q: Oh no, yeah.**

Angeline: And I was in bed for three months because I couldn’t – I couldn’t move, right, and I couldn’t – I couldn’t sit down, I had to lay down flat and I couldn’t make the diabetic – you know, they said to me they wouldn’t give me an appointment for six months. For six months. And in the end, right, I came to renal clinic and I told them about it and they got in touch with them and blew their brains out and I got an appointment. I didn’t have to wait the six months, you know. They saw me within sort of three months of the renal clinic ringing them up. You know, they penalise you without knowing the circumstances or anything like that.

**Q: Has anyone else had similar experiences?**

Lee: No.

**Q: No. You’ve got – okay. So - - -**

Ian: I never miss appointments.

**Q: Okay. Now, so everyone attends both diabetes and renal; is that correct?**

Lee/Maz: Yeah.

Angeline:Well I do.

Ian: Only just renal.

**Q: Sorry?**

Ian: Just renal.

**Q: Oh okay. So who looks at your diabetes?**

Ian: The GP.

**Q: Okay. I was – oh okay, so your GP looks after your diabetes as well.**

Ian: He’s very good.

**Q: Okay. I was going to say, if – I was going to ask if you had the diabetes and renal clinics clashed and you could only attend one, which one would you go to and what would the reason be?**

Angeline: It hasn’t so far, but I’d go to diabetic than renal.

**Q: And any particular reason?**

Angeline: Because I feel that the diabetes is more important than the renal.

**Q: Yep.**

Maz: Same too.

**Q: Same thing Maz.**

Maz: Yeah.

**Q: Okay, yeah.**

Lee: Lee. I would take my renal because I’m not doing pretty good with that.

**Q: Okay, yeah.**

Lee: But renal I want to know about detail, you know. I don’t want miss that appointment for any reason, so – diabetic, yes, I don’t want to miss that as well, but if I have to pick and choose, renal.

**Q: Now do people feel that they’re getting enough information from the renal clinic? I know we had a whole – a group before and some of them commented that they weren’t getting enough information from the renal or the diabetes doctors. They didn’t know what was going on, they didn’t know what to do to help with their kidneys and that wasn’t communicated. Is that something that people identify with?**

Angeline: I would - - -

(Ian shaking his head)

**Q: No. So Ian’s shaking his head.**

Angeline: Sorry. I would like to know a little bit more what caused chronic kidney disease. I don’t know what – what happened for me to be – to have chronic kidney disease. To this day, if you asked me, I wouldn’t be able to tell you because I don’t know.

**Q: How about yourselves, Maz, Lee?**

Lee: Yeah, they give you an overview, a quick thing, but you know, they give you five, six factors.

**Q: Did you ask them though?**

Lee: I asked questions when I’m sitting in the doctors I think. Sometimes they don’t like me because I ask questions, but that’s what I’m there for. Definitely want to like – would like more information, like a, you know, a session like this or something like that that – how it develops, what can you do. I don’t know, I’m told kidney’s not reversible. But that’s how it is. But yeah, a bit more information will be good because like, I read about diabetic a lot when I – I was diagnosed because I went on internet, I talk to a few people who had that and, you know, you get information out of that people – those people really good. So I’m aware of diabetic conditions, but renal I want to – definitely want to know more.

**Q: Yeah. Maz? Oh sorry.**

Lee: Sending out like an information booklet or something that we can read, you know. Something that can be like diabetic, I know they – they post every three months they post something. I don’t know if you guys get it or not. I do get and they keep sending you like a magazine.

**Q: Oh from the Diabetes Society, yes.**

Lee: Yes. Diabetes Association.

**Q: Yeah, Diabetes - - -**

Angeline: Well I’ve never had it. I’ve had diabetes for 20 years and I’ve never had any information on it. (ian laughs)

**Q: No, this is from the Australian Diabetes Association.**

Lee: Association, yeah.

Angeline: Oh right. Okay.

**Q: So if you’re a member they should be sending you out a magazine.**

Lee: You have to contribute a little amount. That’s a donation, so …

**Q: Maz?**

Maz: I think – I think I went through all the education about renal. I even go to the education about dialysis. So I think I really informed about what happened to me, yeah.

**Q: Yep. Okay. Let’s move on. This is a bit – this question’s a bit fun I guess. So you guys have mentioned some comments about strengths and weaknesses and things that you find effective and things that you’re frustrated about the diabetes and chronic kidney disease services. Now, I want you guys to imagine that you were the director <laughs, Ian also laughs> of the – of Hospital 4 Health and you have the power to change things for the diabetes and chronic kidney disease services. So if we were going to design a perfect health system or service which would meet your needs, what would that look like, what would that include?**

Lee: Lee here - - -

Maz: More doctor probably.(Interviewer laughs)

Lee: Lee here. Like I’ve seen there is TVs, places where you see lot of other things, right, channel seven, channel nine, sometimes they do Fox Sports. I think if in the clinic of diabetic, why can’t they put something about diabetes, while people are sitting and while waiting they have that information, you know, some bit of education for people to do – to have a look at it, to hear that and see it. I think I would do that first. It’s more information about your own disease what you have, like kidney renal, talk about kidney. Put something on telly in the clinic.

**Q: Rather than the - - -**

Lee: Rather than - - -

Angeline: Than entertainment.

Lee:- - - Hi-5 or something like that.

**Q: <laughs>. What does everyone else think on that or any other things they would improve or put in or …**

Maz: I think that’s why I say, maybe more doctor so that we don’t have to waste more time on the waiting room. So really frustrating. So more doctor would be more efficient and would have the same doctor.

Angeline: See the same doctor.

Maz: Yeah.

Angeline: See the same doctor.

**Q: Yep. Anything else?**

Lee: Also if I am director and I have power to do it, if people have appointments I should give them a discount for parking, you know, something, you know, that’s manageable for everybody.

Angeline: Incentives.

Lee: Yeah, that’s manageable, like you know, buying $30 for couple of hours and you park it on the street you get $220 as a fine, so you don’t move your car, which is always a high risk, you know. So something like that which, if you have an appointment, you know, like today I know with the letter said you get $9 for parking. For me, I’m very happy to park but otherwise I would be parking somewhere in the street, somewhere far away and then have to walk down here, you know. Some – some – something to help people, you know.

**Q: Yes. Yes. Anyone else got anything else they would put in? Come on, it’s your chance guys. <laughs>. 57:29___________________**

Lee I suppose you don’t like to voice your frustrations.

Maz: Like you know the service – the service is good, so we won’t touch that anymore, you know, because it’s visible we got the education that we need, everything. So what else to comment? Nothing.

**Q: Angeline?**

Angeline: No, I have nothing really to say.

**Q: So some people have said things like more accessible contact with nurses when they need it, or perhaps education which was more specific for them, or some people mentioned cheaper parking, or some people even mentioned a combined renal diabetes clinic so that they don’t need to attend two appointments but they’ve got one appointment. I mean, what do people think about those things?**

Lee: I strongly agree with that.

**Q: Which one?**

Lee: Have the diabetic and renal. Why can’t we have appointment on the same day, one after the other or something like that? So you don’t – if you’re coming to Hospital 4, you know, with traffic and parking it becomes a bit of hassle, so like, I mean, I talk to you and I said if I can have on 27^th^ I can – I take this session, and then I can go to my appointment. I don’t have to take another day off. So – which is always helpful. Appointments on the same day. You take a day off for that and that’s it. You don’t have to worry about it for three months again.

Ian: I think we do that automatically anyway. I know I do. If I’ve got to come to renal clinic I try and make my appointment with the cardiologist the same day.

Lee: I mean, you are lucky.

Ian: Just try to fit everything in in one - - -

Lee: You can be lucky but you don’t always get it.

**Q: What about – what do other people think?**

Maz: My – my diabetic and renal is not happen to be on the same day, not even at the same hospital, so I don’t have to - - -

Ian – laughs: that makes it difficult

**Q: Where’s your diabetes and where’s your kidney?**

Maz: Hospital 4a for diabetes and renal – Hospital 4 for renal.

**Q: I mean, it’s still the same. It’s the whole – the same network but it’s on different sides.**

Maz: Yeah, it’s only different hospital.

Lee: That’s the question. Is there a renal facility in Hospital 4a where people like us can go for - - -

**Q: For appointments, no, not at the moment. Not at the moment. But this is why we’re holding sessions like this anyway, so this is your chance guys. Anything else? <laughs>. (everyone laughs)**

Lee: Hospital 4a is easier for us, you know.

**Q: Any other things if you were director and you could change things?**

Angeline: Actually having it at your local hospital would be a lot better than having to travel, you know, to do – to – I – I don’t so much agree with having the same appointment for the two clinics, right, but I do think having it going to your local hospital rather than having to travel would be – would be good.

**Q: And with the – when you said you don’t agree with the - - -**

Angeline: Because I think if – if the same – if you came to the same appointment for both diseases, I don’t think you’d be getting as good a service as you do coming separately. That’s what I feel anyway, you know. When you go to diabetic clinic it’s for diabetes. When you come to renal clinic it’s for chronic kidney disease, you know, and you get a good service from both of them, but I think if you combine the two you wouldn’t get that – as such a good – I – I personally don’t think you’d get such a good service.

**Q: Just to act as the devil’s advocate - - -**

Angeline: Because – because, right, the doctors who do diabetes aren’t the same doctors who do renal, so you’d have to be sitting in front of two doctors at one appointment. That’s what I feel, you know.

**Q: I mean, just to act – I mean, you could see both doctors in the same clinic, I guess, but just to act as the devil’s advocate, some people find that particular aspect quite frustrating because one person looks at one part of the body, one person looks at the other part.**

Angeline: Yes.

**Q: And then they don’t communicate and then they don’t feel that they’re getting - - -**

Angeline: Yeah, that’s what I was trying to say, yeah.

**Q: They don’t feel that they’re getting holistic care, so just to act as the devil’s advocate, some people would disagree and say they would rather have everyone look at everything at once rather than having little bits.**

Ian: That’s why you have a specialist.

**Q: So I don’t know what other people think.**

Lee: I think if you come for appointment, the diabetic clinic should be doctor seeing you specialised in diabetic. You know, you can’t have a GP sitting there reading the comments and then talking to you. I mean, anybody can do that. Well, you know, should have specialist who knows about renal part of things, and because like person like me ask all the questions and if I see you cannot give me the answer, I don’t want to see you again.

Angeline: Yeah, but then don’t you think – sorry – sorry for butting in – if you’re looking at a renal specialist for your renal care and a diabetic specialist for your diabetic care, right, they’re not both going to sit in the same room. You’d be wasting more time having one appointment to see two doctors, right, than coming and seeing a doctor in renal clinic and coming and seeing a doctor at diabetic clinic.

Ian: Well, you asked - - -

**Q: What do other people think?**

Ian: You asked if we had the power to change it, that would be one thing. Have one appointment at two o’clock and another one at three o’clock.

Lee: Yeah, should be, yeah. Because I mean, if – if I’m not working, that’s fine. But if you’re working, as I said, you know, you really have to bargain this time off with your employer and say – I mean, if you tell him, “I’ve got three appointments in the same week”, they sort of looking at you funny and they say, “I can’t let you off all the time”, you know. For me, as a working person it suits my lifestyle. So it may be different to you, it may be different to you. I don’t know.

Angeline: Now you’re saying have an appointment at two o’clock and another one at three o’clock. How many times have you come for an appointment at two o’clock and seen your doctor at three o’clock?

Ian: So it would be put back a half an hour or whatever. There’s plenty of patients they could see in the meantime.

Angeline: You’re not going – you’re not – you’ll be wasting more time hanging around.

Ian: You wouldn’t be travelling in twice, that’s the point.

Lee: Paying – paying for the parking, you know. I don’t mind that – I’ve been to so many appointments, but I haven’t been called on time at any stage. It’s always half an hour to two hours sometimes, and that’s how it is. I mean, everybody go through the same process.

Ian: Sure.

**Q: Any other things people want to improve if they had the power to change things?**

Angeline: Well now, the director can’t change – I’m just talking, why must it be insulin that one has to take, diabetes, you know, other than – other than tablets, and tablets have a side-effect too, you know, but you’re talking about a director of services.

**Q: Yeah, no. You can’t change that one unfortunately.**

Angeline: You can’t. Unfortunately you can’t.

**Q: No. No. Any – anything else in terms of if it was a perfect health service?**

Ian: There isn’t. There wouldn’t be a perfect health service. (angeline laughs)

**Q: No, I know. But if you were deciding it though, any – anything else people would want or want to include or …**

No, I think *1:06:07.4.

**Q: Yes, okay.**

*1:06:10.5.

**Q: Yes, okay. Okay. And what about in terms of education or contact with the health service and things like that? Everyone – every – would anyone change anything or they’re pretty much happy with what’s …**

Angeline: Oh I tell you what I’d like to know. Why is it, I take medication that can only be given to me when I come to renal clinic? Why does that have to be that? Why can’t my – why can’t my GP prescribe it and I get the same terms? I take, for instance, right, I take medication called Aranesp

**Q: Yes, yes. Yes.**

Angeline: Right. And I can only get it when I come to renal clinic. I can’t get it any other way, you know.

**Q: Yeah, I – I think that’s - - -**

Angeline: If I get it any other way I have to pay $800 a shot.

**Q: I think that’s because of the specialist medication, so you know, only specialists can prescribe it, I think. That’s a - - -**

Angeline: Yeah, specialists can, but – but - - -

**Q: GP – I don’t think GPs can.**

Angeline: Yeah, I know, but it would be good if he could.

**Q: So that you don’t need to come out; is that right?**

Angeline: But I have to come – I have to come to Hospital 4a to get a prescription filled or come to Hospital 4 to get the prescription filled, you know, and I can only get a prescription from renal clinic.

Maz: Is that so? Is it?

Angeline: Eight hundred dollars a shot.

Maz: I didn’t know that.

Angeline: Well, do you take it?

Maz: I didn’t. I didn’t. I didn’t take that kind of medicine, but I do go for renal but they give me a prescription which my GP can sign for it.

Angeline: No, no, no, this – the GP can’t sign for this one.

Maz: It might be – it might be a different medication probably.

Angeline: Yeah. No, no, because I’m – because I’m – I have very bad - I have this injection and every time I use the injection it’s $800 a shot and I use it every fortnight.

Ian laughs

**Q: Okay. Has anyone got anything else they want to add in terms of if they were the director and they could change things? Anything else?**

Maz: No.

**Q: No. Okay.**

Lee: I would have a tea or coffee machine.

**Q: <laughs>**

<laughter from everyoner>.

Lee: That will help to pass time.

**Q: Okay. So look, obviously we want your help to help us evaluate our current state of play with our services and improve the services. Is there anything else that we’ve missed or that you’ve come wanting to say that you haven’t said? Any other points you want to raise?**

Angeline: Well, the only thing I wanted to know was the side-effects of chronic kidney disease.

**Q: Yep. Okay. Well we can talk about that later, but in terms specifically of related to the actual – your experience and improving the health service, is there anything anyone else wants to say or they came wanting to say that they haven’t had the chance to say?**

Lee: No, I think I’ve pretty much covered what I wanted to - - -

Ian: Covered it I think.

Maz: I think we need to spread out the clinic to other location instead of just a main hospital, yeah, because it’s quite – very hard for someone to travel up and down and everything.

**Q: So you’re talking about access, local access?**

Maz: Yeah, that’s right.

**Q: Okay. Awesome. Anything else anyone wants to add or …**

Lee: No.

**Q: No. Okay. Well why don’t we move onto the surveys. So it’s just one. Now a quick question. So …**

END OF TRANSCRIPT

**Focus Group 9**

**Q: So the questions will be a group discussion questions and it will just be related to your experiences with diabetes and chronic kidney disease. So we’ve already gone around the circle and we’ve spoken about how long we’ve had diabetes for and chronic kidney disease for and also where we’re being followed up. I just want everyone to think about their experiences that they’ve had with the diabetes and chronic kidney disease services here at Hospital 1. Based on your experience what would you say are some weaknesses of the diabetes and chronic kidney disease health services?**

Rolf: Here in the hospital it’s really good, you come and get tested and…

**Q: That was Rolf, sorry if you could just say your name first Rolf.**

Rolf: And I’m quite happy to come here and get that done here. And same with the renal, the renal is very good too, I’m very happy, I come three days a week here, more seven o’clock in the morning, so I’m quite happy and they are really helpful the nurses. So I cannot complain. The only complaint I have is the cold.

**Q: Any other weaknesses or things that could be improved with the diabetes or chronic kidney disease services here at Hospital 1?**

Andrew: I don’t particularly think that they can be any better. Every time I’ve been in this hospital it’s been excellent, like the other gentleman mentioned, their services is good, their attention is good, the staff are good, and I suppose it works both ways. There are some patients who are difficult, but on the whole generally they are excellent. Whether it be diabetes or kidney failure, they are very thorough.

**Q: So some people have mentioned things like the waiting times, issues with parking, things like that, so any weaknesses or things that can be improved here for the diabetes and chronic kidney disease?**

Rolf: Well I had when I was still driving here I paid the ticket for $2 a day, so the lady always went there to the office and bought for a whole month, cost me $18 for the parking and that was very good organised. So I can’t complain about the parking, the only thing is I was in hospital, my blood pressure was down, so they kept me here for a week and my son picked me up on a Sunday morning, we got a parking ticket for $30. And I had still two weeks owing to me for parking, I just paid to take the license off me while my eye sight they reckon is not good enough.

Jeffrey: Just want to say that once the services are available and yes they are quite good, it’s getting the services that appear to be a problem. Obviously time waiting is a thing, three monthly appointments tend to extend out to four five months, because you just can’t get into the clinics. In general terms which is a bad thing, unless there is a problem, unless it’s an urgent problem for which you present earlier or to A&E, the availability of general access isn’t as good as what it could be and that’s something that can’t be obviously controlled. Obviously the numbers are extremely high, the service is in high demand. But it does allow for people to slip a little bit into the cracks especially if they're not monitored at time. If you're on six, 12 monthly visits it’s obviously not a huge problem, but somebody who is being monitored frequently it can be; it can put you back a little bit perhaps.

**Q: What about the waiting time in the clinics when you arrive, is that an issue for anyone?**

Rolf: That is an issue, I’ve been many times my appointment was say 2:30 and a lot of people came after me and practically I was nearly the last person to get in and that was about two hours later. So when you make the appointment do they appoint all the same time the people or how does it work?

**Q: I’m not sure.**

Jeffrey: If you read the actual letter it says they're not appointment times, they're times of arrival and they're actually done en mass. So two people get the same time, [rolf – oh] but they actually specify that they're not appointments, therefore you have no rights to complain. And they ask you to wait, you're asked to accept it… that’s public health.

Rolf: Yeah well with the people coming, they just coming and going in straight away and you stay and wait and wait and wait and wait.

Jeffrey: That’s something you have to ask the clinic.

**Q: Just want to welcome is it CP here? So we’re just talking about weaknesses or the things that the services could improve in terms of the chronic kidney disease and the diabetes, so what are some weaknesses of the current services that are happening. And I think some people have mentioned just the waiting time in terms of accessing the services sometimes, maybe three four months sometimes when you want to get an appointment and also someone just mentioned, well some people mentioned sometimes when you get there you wait a long time; it’s not such an issue for other people. Any other weaknesses that people can think of or things ways it could be improved?** [etan shakes head]

Etan: I thought that now they split the arrival time up, they… I think that they split them up, because sometimes the renal people they tell you you can come, you get a choice, nine, 10 and 11, I usually take the 11 o’clock because less waiting, because I don't like getting up early in the morning anyway. So I find that better. Over the years yes there is a lot of waiting time, but expect that when you come to a hospital, there’s a lot of people and depending how long the consultant is, for how long, sometimes they take a long time depending on the person, sometimes they make a short time. It’s the same as when you go to a doctor or even to a specialist you find that you’ve still got to wait, so depending on how the time on the clinic. The diabetes clinic I find that when you get in there, first thing they're fairly quick by getting your blood sample, so you get your results and they're quick to get back to you if there’s anything. Because I mean one morning I came in my sugar was a bit low and they come in straight away and gave me something and told the doctor. But my three monthly was averaged out, because they do sort of two tests. They're fairly good with that; other than that it’s just a waiting time I suppose. I usually take the later time, because end up less; you come in nine o’clock you could be here for three hours waiting to get in. And again depending whose doctor you get; sometimes you get the same doctor, if you don’t, if you're not as choosy you can take anyone and you get out there real quick.

Jeffrey: Which actually brings up a good point, quite often if you see different doctors who are not familiar with you, (there’s a continuity, it’s quite good because the doctor may perceive and know you), but if there’s not a continuity the doctor reads your last notes and makes independent judgments, therefore there may be changes. Just different things that may happen that they're not consistent if you were seeing the same person.

Etan: Then I found that the people when they were a bit hesitant asking to change stuff, that they went and asked, they’d take me in to where the more senior ones and they do go and ask them apparently. So if there’s any change that’s regular, it’s normally you go and see them and nothing’s changed in your results, they let you move on. It’s just that they find there’s something they're not happy with, they go and usually ask a more senior person. So I’ve been in the system over 30 years, diabetes I’ve only been for five years and I found first time I had to go every month, but after that I’m now doing yearly, so it’s starting to be a bit under control.

**Q: Anyone else got any other weaknesses they want to mention about the diabetes and kidney disease services?**

CP: Yeah all mentioning regarding the waiting time in the hospital, I’ve been on dialysis doing peritoneal dialysis for the past four years and I had an infection two years back and twice my operation was postponed. And one day it was for morning till late evening I had to wait and they put me into starvation without any water or food nothing and towards the last six o’clock they come and tell me today is not possible, you’ve been postponed until some other day. And the next date also was cancelled and so the inconvenience cost and the problems were increasing because I’m coming all the way from Hospital 7 and have to stay in the hospital the whole day and nothing is done and go back in the evening and again wait for the next appointment and things like that. So that was the issue once and one more issue I will tell you is last year I broke my hand and the paramedics put me into Hospital 4a and since they don’t have the dialysis facilities over there I was asked to come back here to Hospital 1 which took me a couple of hours to reach here and after I came here I was in the stretcher hailing with pain for about three four hours. And finally it was so bad I had to shout and ask for help and one of the nurses came and told me it won’t take too long, the doctors will come. I said you give me some pain killers at least. And then after about one hour the doctor came and they put me in a room and they gave me some pain killers and things, so the waiting time and the certain places now the facilities also create a lot of problems.

**Q: What about the actual, so is your renal disease and diabetes looked after here at Hospital 1 CP or?**

CP: For major operations I come here and the other things are Hospital 7.

**Q: So in terms of who looks after your diabetes?**

CP: I don’t have diabetes, I’m type 2.

**Q: So your type 2 diabetes who looks after that?**

CP: Nobody.

**Q: Just your GP?**

CP: Yeah GP and my what do you call it the kidney specialist.

**Q: And the kidney specialist where do you see him?**

CP: Hospital 7.

**Q: Do you see him there privately or in the public clinic?**

CP: Public clinic.

**Q: So anyone else got any other weaknesses they want to mention?**

Rolf: About what he just mentioned about stay there the whole day; when you ask the sister she get you some sandwiches and a drink when the diabetic clinic and you stay longer than you have to, you ask the sister and say oh you feel you need something to eat and they get you some sandwiches and a drink.

CP: No that’s for your diabetes, but the day you going for the operation you're not supposed to eat or drink anything.

Rolf: Yeah that’s a different story.

CP: Yeah my case was that.

**Q: Anyone else got anything else they want to mention, Glenice did you want to mention?**

Glenice: Well I haven’t said anything because I’ve been full of praise for Hospital 1 and they’ve treated up very very nicely and well and sometimes you get shuffled in sometimes and you think oh ok. But they’ve treated us like grandparents.

Geoff: But the thing is if we have an appointment and we come in on an appointment time, we only have to wait about five minutes and the two nurses that are looking after us take us and they do what they have to do and we go off. We’ve never had to sit here and wait for hours, we’ve had excellent service.

Rolf laughs

Rolf: You are lucky.

They’ve looked after extremely well.

Glenice: I’m full of praise for them.

**Q: Now a similar sort of question, what are some of the things that you find frustrating about the diabetes and chronic kidney disease health services here?**

CP: And when you think about for example Hospital 1 is going to give you a donor, going to give you a kidney and then you think about your operation and whether that will reject or whether in later stages whether you have an infection, that’s all these kind of things are frustrating, because the problems, some people like to stay on dialysis, because they want to avoid all these kind of things.

**Q: Sure, but what do people find frustrating about the actual health service experience, what aspects are frustrating? I think some people have mentioned waiting times and things like that?**

Rolf: Waiting when you come here in the mornings and you sit here for hours. Sometimes next on the 31^st^ I have the renal department, renal thing, that is three o’clock so I don't go home, I stay here, otherwise I have my dialysis, so I wait here, sometimes come home about five o’clock at night, that day is frustrating yeah. I come, I have my renal, then I have something to eat in the day and wait till my appointment is. I go to my appointment and that’s why I have to wait and sometimes I come home about, I come here seven o’clock in the morning and I come home five o’clock at night. So that is frustrating.

**Q: Anyone else got anything else they want to add about frustrating?**

Andrew: Well I’m Andrew and listening to the people here, all these problems seem to be coming from Hospital 1. Now this gentleman here Etan will tell you that we’re at Hospital 1a and we don’t have the waiting problems. Our doctor comes on a Friday, the renal doctor comes…

**Q: So you get reviewed at Hospital 1a, not at Hospital 1 is that right?**

Andrew: Yeah that’s right, so the problems that we are listening to now generally attributed to Hospital 1 and we don't have that problem of sitting in renal clinics and diabetes clinics. We have the doctor come there on Friday and he takes his patients in and they're very quick. This gentleman will correct me if I’m wrong; they're very quick. I mean I have my appointment at quarter past one, that’s when my dialysis finishes at one o’clock and quarter past one he takes me in, he looks at me and I’m home by 25 to two. So the problem seems to be coming from Hospital 1 and not; well I speak for Hospital 1a and what I can say about Hospital 1a. And the only time I come here is when I had a transplant I had to come to this clinic here and even then Dr. Manahan was pretty quick, I didn’t have any problems. But listening to the people I can understand that they might have a wait here.

**Q: And your diabetes who looks after that or your GP?**

Andrew: Well my GP looks after my diabetes, but always the renal doctor helps as well, so he looks at both at once.

Etan: I’ll just say that I’ve done both, I do Hospital 1 and Hospital 1a, when I used to be at Hospital 1a it’s a lot quicker, because the clinic is smaller, whereas when you're at Hospital 1 it’s a huge clinic, there’s a lot of people coming in all the time, there’s a lot more doctors, where at Hospital 1a there’s only the two doctors, where Hospital 1 there’s up to five or six. So the clinic is much larger and yeah that’s all, I find it just a large clinic thing. I found that they have two clinics, because when I had a renal transplant, they moved me to a Wednesday afternoon clinic, which was a quicker clinic, but that was because I had a transplant. And so they moved a lot quicker and it was easier to get in. But when it was just normal kidney it was a longer wait by the time doctors see you, but when you went in transplant, which there’s certain things they were looking at when I first come, so the clinic was quicker. I find the biggest problem when you're in the hospital is when you're inside it and when you get moved around, unless they move you to a renal ward, you have issues on the wards, because a lot of the wards don’t understand the issues. When I had the hip operation here I was in the orthopedic; eventually they had to move me to the renal to get the proper sort of care, because they started to worry more about the kidney than they were worried about the…

**Q: So Etan you're hemo dialysis or peritoneal dialysis?**

Etan: Hemo, I wouldn’t do anything else. I’ve done it twice, I did it 17 years ago here at Hospital 1, I originally got transferred out of here to get a transplant because originally they didn’t do living related transplants back in my day, so back nearly 18 years ago, they transferred me out to the Hospital 4. So I spent time at Hospital 4.

Glenice: A while to get here.

Etan: To get to this, I get to move around and I’ve been in here and they were transferred, if I had a triple by pass and they transferred me out of here to Epworth because I needed to get it done real quickly, so I found the service had been great, just they have an argument with me, discussion over what their better options are at the time. So same with the transplant, I had it as the clinic goes, I’d been coming to the clinic; originally I was outside, Dr. Stambe came back into the hospital and he bought me in with him. Originally 30 years ago I had a kidney operation and I find more issues when you're in the hospital with the nursing because they seem to be busy.

**Q: So it’s more when you were an inpatient rather than an outpatient?**

Etan: Yeah outpatients I find it’s fairly… Alright I find - inpatients if you need to go somewhere and you're desperate you're calling for a nurse, unless there’s someone… which is great my wife used to be around so she used to take me out, but I find that there’s a waiting time, but I expect that with a larger clinic and I get that even at my own GP I find that even when I go to the GP you make an appointment with him at five, you could be up to an hour before you get to see him. So other than that, most of the services I’ve been in have been fairly good. I find that it doesn’t bother me which clinic I go to, I get to see a doctor and if there’s further issues they seem to go out for a minute and talk to more senior people.

**Q: So anyone else got any other frustrating, things that they find frustrating about the diabetes and chronic kidney disease health services?**

Jeffrey: Just one of the problems if you're seen frequently is the frustration in multiple appointments because different clinics are run on different days. This week I’ll be here at Hospital 1 three days a week, next week I’ll be here three days a week. Diabetes, renal, whatever, it’s unfortunate, but the clinics just don’t run on the same days and unfortunately your tests don’t run on the same days either, they have a lot of trouble. It’s just frustrating coming backwards and forwards if it’s a long trip. But again something that really is beyond control.

Etan: Talking about that I do have an issue, because the diabetes clinic is on a Wednesday morning, my issue is I have dialysis on Wednesday morning, so I have to change for that day to an afternoon.

Jeffrey: Do you change your clinic, the diabetes or the renal?

Etan: The renal, you can’t change the diabetes, it’s only on the one day. I tried to get an appointment, it’s on a Wednesday morning and I just happen to be doing, so I’ve got to go and tell Hospital 1a to get me changed. But I find it is right, because renal has two different days.

Rolf: They’ve got Mondays and Wednesdays.

Etan: And also Hospital 1a has got Friday, so you’ve got a choice, where the diabetes you only get the one day which is sometimes not convenient. If they had another day would be really good, I never thought about that yeah, after you’ve mentioned it, yes it is very frustrating, especially if you're a renal patient and you’ve got the same day and you just cannot, because I rang up asking do you have another day and they just don’t seem to have another day or *0:25:17.5 which is an issue. I never thought about that, yeah that’s frustrating, I’m only coming once a year, but when I was coming more regularly I guess it would have frustrated me because being on the machine on the Wednesday morning you're just going to need to change it and it throws your day out. Yeah renal has several days where diabetes only has the one day, Wednesday morning.

**Q: Any other frustrating factors, besides you mentioned having too many appointments?**

Jeffrey: Only that sometimes your lifestyle is very much changed by the manner in which you're treated. Renal dialysis is a probably much easier thing, because it’s three days a week is it?

Yeses.

Jeffrey: Whereas it’s a set time and you're going to a set place, whereas peritoneal dialysis because it’s seven nights a week overnight…

**Q: Are you peritoneal Jeffrey?**

Jeffrey: It does use so many hours you have to adjust. If you have appointments in the morning it means that you actually have to start your dialysis a lot earlier so that you finish early so that you can get to your appointment early. If you’ve got a late appointment it’s perhaps not a problem, but it means that your life is basically guided by what you do at night. And it depends on the regime you're on; if you happen to be on a long regime it takes a large part of your time.

Glenice: We hang on end of therapy, now we’re free.

Geoff: If you don’t you can’t get off the machine. I can just raise one point, just listening to everybody talking, we have a hospital not far from us, the Hospital 1b, which is part of Hospital 1, the only thing wrong with the place, it only had hemo dialysis. I went in there to find out information about the amount of gear we’ve got to dispose with and they did not know what peritoneal dialysis was.

Glenice: [overtalk].

Geoff: Now there’s no set up for the Hospital 1b, though it’s Hospital 1, just extended the hospital, just put a new outpatient in, nursing station, but they’ve only got hemo and my wife is not on that. We only go, we do the peritoneal dialysis at night, which at this stage works well for us, but allows us to go out if we need to during the day.

**Q: So any other points that people find frustrating or a weakness? Other people have mentioned that they get inappropriate education or not enough education, especially on diet or other people have also mentioned that there’s a lack of holistic care. Like they see different specialties and each specialty is just worried about their own little thing and there’s…**

[Jeffrey makes scrunched up face, looks like he is not sure – is this of the question?]

Etan: Is that because they're more afraid of interfering?

**Q: Well no I’m just saying these are just points that other people have raised, that they see different specialties and everyone is just worried about their own specialty without considering the whole patient. Does anyone identify with any of those and some other people have mentioned poor communication as well between doctors and patients. So the doctors don’t explain enough, they say something and the patient just doesn’t know what's going on. Does anyone identify with any of those frustrating factors or [overtalk]?**

Rolf: I have my dietician comes to my when I’m on dialysis and quite very helpful.

**Q: Whereabouts are you dialysed Rolf?**

Rolf: Here at the renal department. And they supply me with some drink. I have some trouble eating, so I don't get enough protein or something what you say and they gave me a big box drinks like if I need a drink, I forgot the name what it is. And they are very helpful there, they come and tell you that for six months they come, when I want more drinks I just ring up and everything gets organised. He sent me that home, I got no trouble with that. And sometimes it’s another thing frustrating is in the moment I come here with ambulance and in the mornings ambulance always on time, but in the afternoon when I’m finished dialysis sometimes you have to wait two hours to get a lift home. That is frustrating too.

**Q: Any other frustrating factors? Any other points I mentioned, can anyone relate to those? So not enough education about diabetes chronic kidney disease or not enough advice about diet or poor communication between the doctors and the patients or just no one pulling everything together and seeing different doctors and everyone just worries about their own little bit and no one actually seems to know what the whole picture is or no one seems to be pulling everything together.**

Etan: I don't find that with the renal and the diabetes, they seem to work fairly well and the renal people always check that out. I did find out with other departments, I find that other departments in this place had that same issue, the issues where the doctors are just concerned about their certain areas, but that’s only the stuff I’ve had to do with. But not if we’re only relating to diabetes and renal, no I found them very…

Jeffrey: I tend to agree with Etan that diabetes and renal tend to be very coordinated, but they are very wary of other units. I know that some of my treatments have been delayed, because renal won’t interfere with transplant; transplant are not very quick in getting back to renal, but they don’t act on it. But they don’t want to tread on anybody else’s toes, because it’s not their specialty basically. It’s not a case of not wanting to cover everything, it’s a case of being wary of medications they're not familiar with or regimes that they're just not happy with. It does resolve eventually, but it’s a time consuming thing, because there has to be discussion, there has to be consultation.

Rolf: Before was my renal doctor Mr. Septile he was the boss from the whole clinic and he always advised me or told me what's wrong and so. He retired and now there is another young doctor there…. and every time you get a different doctor when you come here and some of them are alright and some of them they say oh come back in so and so…. You sit there for two hours and then you go in there for not even five minutes, oh you're alright, you're alright. So you haven’t even got time to ask them some questions, they want to get rid of you. So that is… it was good when I had my own doctor there.

**Q: Does anyone relate to what Rolf is saying?**

Etan: The only thing I can say relating about doctors.. what you were saying about doctors.. I just got brought in three months ago in to the emergency department and I got taken to intensive care, I know that there they're not allowed to interfere, the renal people, but when I got into emergency department, the renal people had to come down, because intensive care unit thought I was having a heart attack rather than a blood poisoning whatever they call it. When the renal people came down they picked it really quickly what the issue was, removed the permacat. They removed it and then I started to get better and I found the two units they're yes when you're in emergency they look after you, where the renal got stepped in, but when I got transferred into intensive care for a day, the renal people weren’t allowed in. Whereas once they established it was more renal and they moved me to the renal and then they started to recover. But if renal people hadn’t come down in emergency and picked the issue, I think I’d still be thinking it was a heart attack rather than being a poison. So it’s really good to get them to come in. There is a point where they don’t always see eye to eye, I don’t know why because I was in the emergency department with the emergency doctors and then they finally asked the renal people to come down and have a look at it.

**Q: Some people have mentioned as a weakness poor communication between doctors, different specialties and even between the hospital and the GP, so the GP doesn’t get letters or the communication is not great. Does anyone notice between units?**

Rolf: That is true and I had cancer taken out on four places, so I had three surgeons working on me on the same time and it was alright and then they put me in hospital here while my blood pressure was not high enough so they put me a week here in hospital. The renal doctor and the plastic doctor came and had a look at it, I didn’t even have time, he was so busy, to ask him if everything is alright and he said oh it’s alright, yeah just had a quick look. I have one here, I had one here, got one up here, one on the back [pointing to body] and so I was yesterday here and had yeah was yesterday and had another appointment with the plastic doctor. And they took the stitches out and the doctor just came and had a look, told the sister to take the stitches out, that’s all what I’d seen. I asked the sister and she said oh you’d be alright.

**Q: So is it easy for people to get their diabetes and kidney health care that you need? So is it easy for you to access the diabetes and kidney health care that you need in terms of locality, cost and affordability and waiting times? I think we’ve spoken a bit about the waiting time, what about locality and cost and affordability? Do people find it easy to access the diabetes and kidney health care that they need?**

Rolf: There is the cost and I don’t live far from here I live in Albert Park. For me it’s quite convenient, bit of a waiting time, but otherwise.

Andrew: I don't have this problem with the diabetes and kidney coming to the same place, because my diabetes is looked after by my GP so I don’t have that problem. That’s eliminated from Hospital 1, and there is no cost involved if you are on public. I always am covered by private Medibank, but the public service looks after you on diabetes, you have no choice, you can’t go to a private hospital. Sorry if you have renal problems you cannot go to a private hospital. The only private hospital there is is in Malvern to look after your renal and it is basically a public hospital. They haven’t got the facilities to operate, the only people that have it is Hospital 4 and Hospital 1, so this sort of thing hinders you from going anywhere. So you can’t go private if you're renal. It’s not possible, but diabetes you can go private which I do privately with my GP and I don’t have the problem of coming to Hospital 1 for diabetes and waiting in queues or times or whatever, I don’t have that. I just deal with or my GP looks after my diabetes and as I said I come here for my renal problems.

**Q: And that’s quite accessible?**

Andrew: Yeah I come here, I get an appointment at 9:30 or 10 and it’s within half an hour doctors see me, so I have still not come across a problem.

**Q: Etan and Jeffrey or CP or Glenice, any other comments?**

Etan: No problems contacting the renal people since I moved to Hospital 1a, it’s easy to get onto doctors, whereas the diabetic clinic I find because it’s only on the one day, when I try to get an appointment or something like that, it’s very hard to get onto them. I technically don’t know who they are because I see them once a year, I did see them earlier, I used to know who they were, but once I’ve been extended out to yearly, unless I get a message or something from the people, I technically forget, I get a notice, very hard to contact anybody if you really don’t know. I normally go through the renal people to get in to see the diabetic, because renal seems to be easy for me, I’ve just been with them such a long time and I know and you can get in a lot quicker. I only moved to Hospital 1a from here because I needed to do other things that I needed to do physiotherapy and I found that it was easier to get rehab much quicker to do at Hospital 1a and Hospital 1a did both. Otherwise I would still be at Hospital 1, but I just moved there, because I needed to have other issues and which Hospital 1 doesn’t cater for.

**Q: Jeffrey or CP or Glenice?**

Jeffrey: Only that the cost/accessibility doesn’t seem to be a problem in general terms, you can access the services even if you need to do it through the emergency department, so it’s not a huge issue. Costs a little different, routine things covered, but if they play with your medications and that, that can become very very expensive over a short period of time. And also as far as dialysis goes which is a rather new experience is becoming perhaps not as cost effective as what they originally said. There are a lot of things that are hidden costs that aren’t seen which is annoying; if you knew them up front perhaps you would be better prepared, but once you started you can’t very well reverse it.

Glenice: I’ve just got a question, it’s actually more to do with the carer side of things, as it is Geoff is my sole carer and he has to do quite a bit of manual work. I’d never be able to manage by myself and it does concern me a little that he doesn’t conk out. I mean he’s ok and he’s fit, but I think don’t fall over or anything, because you’ve got to change all circumstances. And I try to have a bit of faith about things, but he’s a doer and won’t listen to what I say.

**Q: Well it’s great that you’ve got a good carer.**

Geoff: I’ll just add to it that we don’t come here to the diabetic clinic, we have our own doctor at the National Institute for Integrated Medicine in Hawthorn, only because we’ve known him for many many years and what he’s done with my wife’s diabetes, first thing he said, I’ll make sure you don't take insulin needles, is exactly what he’s achieved. So he does take insulin through a form of a little tablet called Januvia but she does not take any insulin pump or insulin needles at all. And he’s kept that under very good control.

**Q: Now I think some people said sometimes appointments clash, so has anyone ever missed out on a diabetes or chronic kidney disease appointment and if so why?**

Nos.

No I changed the thing around.

Geoff nods

Etan: It does clash, but I can’t do…

Jeffrey: Yes I’ve missed appointments, because there’ve been two made on the same day and basically you can’t be in two places at once.

**Q: So if someone had to pick between diabetes and chronic kidney disease appointment which one would you pick and why?**

Jeffrey: Well the only reason I had to pick it, because I can’t change the diabetes and it’s easy enough to change the renal, they're very flexible, so I’ve had to change it, but that happened to me this year, because the Wednesday I rang up and asked them for a different day and they just don't have a different day.

CP: For me I don’t have a problem with diabetes, but I have a problem with the kidney failure, so if I have any other appointment with the GP or anywhere else I give preference to my renal issues first. So at that point I see to it later or I’ll ask them to give me a different time on the same day, but a different time.

**Q: We mentioned a couple of weaknesses and frustrating factors about the diabetes and chronic kidney disease services like waiting times and things like that and I think that’s probably the main issue that people brought up I think and there was some other issues as well. What do people think can be done to improve on these weaknesses?**

Rolf: There’s not much what we can do.

Jeffrey: Unless the government come to the party [Geoff says yes] with more money, basically there’s not a lot you can, unless the clinics are funded to include more positions for the clinics. It’s alright to have four or five doctors in a clinic but unless you can increase that number to compensate for the number of patients that are being seen, your waiting times aren’t going to go down. And your waiting time in terms of appointments is going to also be extended. I mean they can only see so many people in a clinic. Either that or you go to an alternative, you look for something outside the system as in you have the option to go privately, an expensive option. But public wise if you tie it into the public system then you don’t have a lot of options.

Rolf: Or another thing is maybe when you have your dialysis and the doctor comes down to talk to you while you have your dialysis. You know what I mean? When you're dialysing and like we have the [points to etan and Andrew] trouble with the diabetic, I got the same problem what you got, got on a Wednesday, and sometimes the nurses let me off half an hour earlier that I just make the last appointment from the diabetics. And so maybe when a doctor comes down when you have your dialysis and tells you what you want to know.

Etan: I’d just say different clinic times that’s the only issue that I have…

Rolf: Different clinic yeah.

Etan: More than one time would allow that’s for the diabetics, like I said the renal has several clinics and you choose. I didn’t know that Hospital 1a was going, originally I was at Hospital 1 and you get a choice between Mondays and Wednesday which one is a morning and one is an afternoon, where the diabetics again is only just a morning and you don’t get a choice.

Rolf: Unless they change the time for afternoon for diabetic.

Etan: Yeah unless they make another time, but I find yeah it much easier to change the renal time, but then they're used to that, but I think a lot to do with when you want to move around in the clinics.

Rolf: Yeah it’s the only thing you can do anyway.

**Q: Now we’ve talked a lot about the weaknesses and frustrating factors, I think some people did say they were quite happy, so what are the strengths of the diabetes and chronic kidney disease services here at Hospital 1?**

Geoff: I’ll just speak up, one little point, we have a nurse up here in the renal section…

**Q: Sorry Geoff do you mind if we get Glenice if Glenice wants to…**

Geoff: I’ll speak on her behalf, that particular nurse rings us every two or three weeks to check to see how we’re going. Even our next appointment is not August.

Glenice: Well they used to follow up which is good.

Geoff: It’s the follow up that we’re getting makes you feel you're not being lost out in the system.

Glenice: You get a schedule full of dates, here’s the diary and it’s hard when you’ve got to keep all things in perspective. I find that these two doctors have been doing this part of it, I can’t fault them, they’ve been very very thorough. And I’m not an examiner, I’m just a learner.

**Q: Anyone else got any other comments they want to say about strengths?**

Rolf: Not really.

Etan: Nursing staff seem to be very understanding and understand where they're going and seem to be doing very well. I find nursing very able to and if they can’t they seem to ask someone more senior. So I find there’s a lot of knowledge in the departments and especially when they're needling you I’m finding if the one doesn’t, someone else will come over to help him out. Someone more senior gets it done. I remember when they first started out handling me it was a frustration then, yeah they asked more senior people, *0:51:08.6 and I find yeah doctors have been fairly, the renal doctors have been fairly good, but I’ve been with them for such a long time, they seem to know me. Again diabetic clinic, I have yeah I only see them once a year.

Jeffrey: Accessibility to renal seems to be more prominent than diabetic basically, no matter what aspect of renal they seem to have better I don’t know perhaps more reliant to be in contact.

**Q: Andrew or CP or Rolf, anything you want to add to strengths?**

Andrew: Like I said before, I haven’t had much to do with the diabetes clinic at all here at Hospital 1, but when it comes to renal, which we have not spoken much about, they are excellent. I have no problems with the renal doctors, I have been in Hospital 1 a few times over my kidney transplant and they're excellent. The nurses here are fine, the doctors are fine, the only thing I find is that every three months they change their doctors around and they send them to a different campus and then you get to meet new doctors. So it’s not a problem, but they take over your case and there are a few doctors involved. I had a problem when I had my transplant, I had chronic diarrhea, they couldn’t work out what was wrong with me and they put me in isolation for a week. And they found that the tablets they were prescribing were wrong and every doctor that came to see me changed the tablet [jeff nodding and smiling like he understands], every three days they were changing the tablets and I don’t know whether there was consultation or not, but they were changing. So I was going to my chemist every three days getting new tablets, so I ended up with this chronic diarrhea till they worked it out. So that’s the only problem I had with Hospital 1 and which wasn’t a problem I suppose, but the doctors are excellent and the nurses are very good, no problems at all.

Rolf: I can’t think of anything else in the moment.

CP: In Hospital 1 I have no doubts because the problems the doctors are good, the nurses are good.

**Q: So you receive peritoneal dialysis or hemo dialysis, sorry I forgot?**

CP: PD.

**Q: And then your PD care is looked after here at Hospital 1?**

CP: Yeah but I have a doctor in Hospital 7, so still coming every three months here, they send me over there. So in case if I have any problem and issues in between these three months, I contact my coordinator. So they give me advice what has to be done and what is good for you and things like that, so I just follow the instruction and so far there was no problem, except once I had a, what do you call, infection, till then everything seems to be ok. And I used to tell them if you obviously don’t call me, if I do have any problems I will call you or if I have issues I will come straight to the hospital and get in touch with you.

**Q: Now what do you guys find really effective about the diabetes and chronic kidney disease services here at Hospital 1?**

Etan: What I found effective when I was on renal and they bought me here and I had shingles, they rang me to tell me to change the tablets and once I got the results where I didn’t have to chase it up…

**Q: So this was after the transplant was it?**

Etan: No this is just about last year I had shingles, so I got transferred into here because I was doing isolation and what they were working out was what tablets I had to take to help out and I found out that once they took the test, I went home, the next day they rang me to tell me what to do, so they did follow up and I found that very, which I didn’t have to go looking for, they did the tests and rang me back and told me to take, withdraw a couple of certain tablets, because of what I was having. Even though when I got here they weren’t sure, but once they did all the tests they got back to us real quick and advised me on what to do and what not to do. So I didn’t have to come in which was really, I thought was great for them. I was in isolation, I got transferred out of Hospital 1a back to here for a couple of weeks while the shingles were being treated. Once I got that I was sent back. But I found that good that they rang us up and followed through the tests and gave me a ring.

**Q: How about anyone else?**

Rolf: A fortnight ago when I was finished with the dialysis, the sister said I don’t let you go, I said why, they said oh your blood pressure is down. So they rang up the doctor, the doctor came down and he said right away we have to leave you here, you can’t go home. So they had a bed right away for me upstairs on the second floor and I stayed there one night until he said oh maybe you can go home and it didn’t work out. It was about four or five doctors in the night came to me to have a look at me and so I stayed another night. And he say tomorrow you go home, I stay another night, so it was a week where I was in hospital. And I must say they looked after me very well in that time, the sister came every five minutes and had a look at me and took the blood pressure and it was, my blood pressure is sometimes very low anyway, but they couldn’t find out what's wrong with me. That’s what they say, I ask them and they said what's wrong and they said oh the next day that was Saturday, he said you can go home on Saturday and she took my blood tests for the sugar, the sugar was down. So I had to stay another night.

**Q: So I guess what you're saying is they provided effective care and I guess when there was something wrong they looked after you?**

Rolf: Yeah, yeah.

**Q: How about anyone else, anyone else got anything they want to mention in terms of what they find particularly effective about the diabetes or kidney disease care that they get here at Hospital 1?**

Jeffrey: As long as they're controlling the problem I guess it’s judgmental if your visits are becoming longer and longer apart they must be doing something right.

**Q: Related question, what impact has attending the diabetes renal health services made to your health?**

Andrew: Well I don’t know about others, but it does impact on your health the long run, whether we like it or not it gets you down. Coming for dialysis three days a week is not a joke [CP and etan nod], you got to sit, be strapped into that machine for five hours at a time and it’s not pleasant. Sometimes it has come to my mind sometimes whether I should just stop and forget about it all, it’s a bit frustrating, you can’t do things, there are things you want to do that you cannot do, but then you have to put up with it. The transplant might come quicker than you think, it is a surprise when it does come, which happened to me and that’s the frustrating part that we have to be strapped to that machine for five hours at a time, for three days. It takes a lot of your life away. It’s not pleasant by any means.

Rolf: Yeah that’s true, I have to find that myself, I like to go overseas again to Germany and I’ve organised everything and asked how much I have to pay. You see when I go to Germany I would have to pay for the dialysis and they wanted 500 euros a day. Who can afford that?

Etan: That’s a lot.

Rolf: Here you can go in holidays, when you make all the arrangements you can stay in another hospital and have your dialysis there, or say you want to go to Adelaide or somewhere. This is frustrating, it’s not easy.

**Q: What impact has attending the health service made to your health, has it made it better or worse?**

Etan: Just something I probably would…. to make it better would be to talk to somebody. I go to other units where they have a psychologist talking to me about other issues that I’ve had to deal with, cancer and stuff like that, where they’ve got units where they talk where you're on the machine, renal, the only time I talk to a psychologist was when I was having a transplant because it was a living related one. And that was back when they sent me and I thought that was very good when I was transferred out of here to Hospital 4 where they had a chat to you, the psychologists and all that had a chat to you, to tell you all the issues that you’d be dealing with and what you had to deal with. Whereas as Andrew was saying, is yeah one of the frustrating things about being on the machine is yeah you are frustrated and I get the same issue, being tied down to a machine three days a week and…

**Q: So are there any psychological services here where they get a psychologist to help talk to you about adjusting? Do they have that here or?**

Andrew: You only have to ask and they get someone to your bedside straight away. At Hospital 1a, even here the dieticians are prompt, the psychology people are prompt, so they have all the services, you can access, there is no problem at all. I found that it’s available here and at Hospital 1a.

**Q: Was it automatic?**

Etan: No I found it’s not automatic, you can ask for it, but it’s not automatic in the renal section, I’ve been there for a long time, where it was automatic when I went to the cancer unit, it was just automatic, it was part of the issues straight away. Where you have to deal with, because you're dealing with something about death, where I found because it was my wife, I found that in the renal the only time when it was automatic was when I had the transplant 17 years ago, I find I can ask. I find that whether I like or not, the dieticians are automatic, I’ve no idea why, I’ve never asked for the dietician, they seem to think that I need one. Not that I really, where as a psychologist there’s not; there is, I’m not saying there isn’t there, I didn’t ask, it’s not automatic. I think something people just don’t take for as Andrew was saying, take for granted. It is hard to go through those weeks on the machine and then work out, it does frustrate me, because I’ve got to get up every morning and go three days a week especially since…

Andrew: Like the gentleman over there said (points to rolf) if you want to go on holiday it’s not cheap, you know everybody says yes you can go on holiday, you all know that, but it’s a very expensive item. I just book myself into Hawaii next month for a week and it’s $750 a time for dialysis and it’s not covered by any Medibank or anything here, they don't cover you [Rolf says oh yeah], you can be in Medibank or anything here they don’t cover you. You can be in Medibank Private, you can be in anything, but you’ve got to pay your way through when you go overseas. So for the people who cannot afford it [rolf nods] I can understand that, it’s a very costly; doesn’t matter where you go in the world you can access dialysis, but at the price.

Rolf: England is free.

Andrew: Yes because they have the understanding between Australia and England and New Zealand, there are countries that will give it to you but…

**Q: And I think Jeffrey you said before about with PD can be frustrating because you have to organise your life around…**

Jeffrey: Well conversely as opposed to not being offered, I was actually in the short term offered everything. Cut a long story short, long term renal disease, but…

**Q: Sorry are you talking about the psychology service?**

Jeffrey: Psychology well basically I had long term renal disease, but how do I explain this quickly, basically the cancer hit suddenly, appeared out of nowhere. Now I’ve never seen an oncologist at Hospital 1, I’ve been treated by the renal unit the whole time, sorry renal and urology and I was given the option.

**Q: Sorry you had a kidney cancer or?**

Jeffrey: Long story short, heart transplant, I caught the diabetes because of high dose steroids, I use immunosuppressants and caught cancer. Cancer unfortunately took out the kidneys, literally they took them out. And hence the dialysis. So basically my problems are all iatrogenic, none of them are natural, and the renal unit have been more than, sorry the transplant unit and the renal unit have been more than happy to supply within I think the diagnosis of the cancer which they made it was less than a week and I saw a psychiatrist, a social worker and had cancer removed, which isn’t bad timing for any public service.

**Q: I guess maybe because that was cancer related perhaps.**

Etan: I find with cancer they did it straight away. I’m not saying that renal don’t do them, as soon as they get cancer I got that straight, well it was my wife had cancer, straight away we were seeing someone, whereas I’ve been with the renal department for nearly 30 years and I had a transplant and back like I said 17, 18, 20 years ago they had to transfer me out of here, because they just didn’t do it, but straight away when I went there, had straight away psychology, because it was a transplant in those days, related. Where I find since I’ve been back and I haven’t had any, I had 17 years, not on a machine, I just got back the last two years, but I haven’t had anything to do. I know the service is there, I know you can get it and same as I’ve no idea, I don’t ask for the, who am I thinking of, the dietician, I don't ask for them, I get that anyway. They ask me why she comes and sees me, I even went in there, I’ll go because the service is there, but I don’t really, what they're going to tell me is the same old thing and I’m not going to, I’m not too fussed about diet.

**Q: So in your opinion what do you need to look after your diabetes and chronic kidney disease well?**

Etan: Exercise.

Rolf: What can you do?

**Q: So for example some people mentioned education or support from family or support from hospitals or being motivated, so what in your opinion what do you need or what needs to be in place for you to look after your diabetes and chronic kidney disease well?**

Etan: Just making sure we’re going to do the right things.

**Q: So motivation really?**

Etan: Yeah I’ve lost…

Geoff: Access to current information usually through the nursing staff.

**Q: Anything else?**

Andrew: You got to know your limits with diabetes, you know it’s a terrible disease to have, doesn’t matter who’s got it and your diet is the most important, so if you can control your diet and ensure; that’s what I do, I control my diet a lot and my intake and balance it and it seems alright. My diabetes ranges between six and eight and I used to have it up 16 and 17 and now it’s six and eight, so the doctors say it’s pretty well controlled, just leave it.

**Q: So what you're saying is self awareness and awareness of the disease really?**

Andrew: Correct, you must be aware what it does to you and most of the time it’s your food and drink intake. So if you can control that I suppose. It’s hard, it’s very hard.

**Q: And I guess you're also saying in response, self responsibility as well really.**

Andrew: It’s again like going on holiday, you can’t have beer and scotch and everything else you think, you have to have limitations and self control I suppose. (all laugh, expect CP)

**Q: Anyone got anything else they want to add in?**

Rolf: I got another trouble. I got lung disease, I got scarred lung. So I have breathing trouble, so I stand up and go here in the seat, I’m already started pumping and they cannot find out where it comes from.

**Q: So any other comments in terms of what people need to be in place for them to look after their diabetes and chronic kidney disease?**

Glenice: I think it’s a good idea to keep an eye on your blood sugar levels, that’s important, I take mine once every day and it goes out sometimes, but that’s to do with me. If I have another piece of chocolate. That’s my fault, but if you know yourself, you know your body, your limitations as Andrew said.

Geoff: And just to add to that a little bit, we got a result about two weeks ago from our doctor and he took a blood test and it showed the toxic levels in her body are dropping, because of the peritoneal dialysis is causing the toxic levels to drop, because she’s on peritoneal.

**Q: Sorry Geoff if you don’t mind if we can leave it to Glenice sorry, because we’ve already spoken on the phone.**

Glenice: No it’s just that I think, it takes you a little while to get used to it, but I think it’s working and that gives you hope. And go to bed with a good knowledge, you might wake up in the morning.

**Q: Anyone else got anything else they want to add?**

Jeffrey: The availability needs to be there for renal dialysis, because you…

**Q: The access to health care.**

Jeffrey: Well the access to the equipment which isn’t always the hospital.

**Q: CP anything you want to add?**

CP: I have to keep an eye on my high blood pressure and exercise. And medication and you have to follow the doctor’s advice.

**Q: Related question, what things make it hard for you to look after your diabetes and chronic kidney disease well?**

Rolf: Taking the measurements for your blood. You got all fingers full of holes. That is a bit… I take it three times a day, sometimes I have to pressure mark that I can even get blood out of it.

**Q: Anyone got anything else they want to add? What things make it hard for you to look after your diabetes and chronic kidney disease well? So some people have mentioned having many illnesses make it hard or just being dialysed and you're tired after or depression.**

Etan: Most thing that keeps me from looking after my diabetes - my hips - I've had two hip operations and I haven’t been able to move as well as I’d like to be able to move. It slows me down from doing things I need to do, exercises, moving around getting things. It’s mainly the other diseases that stop me from doing what I need to do. Finding it hard to; not move as well as I used to, since I had my last.

Jeffrey: Sometimes the psychological effects can be somewhat depressing because they're not things that will go away, basically chronic.

Glenice: Sometimes you won’t admit it.

**Q: Anyone else?**

Andrew: No I think it’s pretty good for me.

**Q: So other people mentioned lack of family support or lack of finances or confusing information given by doctors, things like that. Do any of you guys identify with any of those factors?**

Rolf: Not really.

Etan: I have due to, I do have family, right now I’m going through a hard time, I did have support from my wife, she was looking after everything I was doing. I have had to change my way of doing things, she was looking after everything, tablets, appointments and everything else, having to learn to do it myself, so that’s also making what I don’t have right now is the support I used to have three months ago where everything was done for me. She was very supportive looking after me, but since she’s gone I’ve had to change the way I’m doing things. Kids have been great, but again they're not with you, but having to learn to deal with everything on my own is a big change and it’s one of the things I’m going through right now, having to change the way the family is dealing with things, which I didn’t have before. So over the years it’s been great when you needed a cup of tea or something, you would have got it there if you weren’t feeling well, there was someone in the house and now it’s a bit harder, there’s no one in there. Even to make a phone call to get someone to come is a bit of a; yeah just a matter of a phone call, whereas before there was <inaudible>. It’s a bit harder.

**Q: And related to that sort of support, some other people have mentioned the expense of actually getting council support if they need it. That can be a barrier as well, actually getting a care in, because that’s expensive. I don’t know whether any of you guys can identify with that.**

Etan: I can identify with council, because councils aren’t that expensive, because my mother, cared for my mother, I had council in. But the council goes to certain level, when you go to the next level and you go privately, yes it’s very expensive, because when you go outside the council system, councils only cost about $4, $5 and they’ll come, whereas because my mum had some sort of, they transferred into a private system, where it’s a lot more expensive to get someone in, it’s not cheap, they're nearly $30 an hour and you can’t have them for more. Whereas when the council lady comes, again they're limited to what they can do. Even I found that the hospital again was a lot more help with coming out to see us, they sent the district nurse out which isn’t that expensive. Because while I was sick the district nursing which is really good; didn’t realise that because the hospital organised that and once the hospital organised that, I found that that was a lot cheaper than private. District nursing is not as much money, I think it’s about $30 a month or something, not that expensive compared to private organisations where they book you for the hour. And the nurse every day was there, every day changing, didn’t matter what day it was they come and change the dressing and all that. Yes there is support out there, but I don’t think people know what it’s about. I think people need to be told there’s other things out there, like councils and district nurse who will do the same thing as council and as cheap, whereas private companies we have to deal with because there’s a limitation, they're very expensive, they're nearly $200 a month.

**Q: Anyone else got anything else they want to add in terms of barriers that make it hard for them to look after their diabetes and chronic kidney disease?**

Rolf: My son and his wife lives with me and we try to get some help from the council, but we can’t get it with my son living there and he is a worker, goes to work at day. [Etan nods]

**Q: So you guys can’t get it because your son is with you or you can?**

Rolf: He lives with us yes.

Etan: That’s right, council will not come out if there’s someone; my mother the same thing, when I was sick, when I had to move in mum’s for three months, she’s got all this facility to walk around, council won’t come out. That’s why we had to go private, because when you're there they won’t come out. Whereas if it’s a private place you pay the money.

**Q: I might skip to the next question because this is one of the main questions of the whole discussion. Moving back to the health services provided for the management of your diabetes and chronic kidney disease, all of you made some comments about the strengths and weaknesses and things like that. Now we’d like you to make some recommendations as people who have experienced the services and you’ve mentioned your likes and dislikes. We’ll like you guys to make some recommendations. So imagine for example you became director of the hospital and you had the power to change things about the diabetes and chronic kidney disease health services. If we were going to design a perfect health service which will meet all your needs, what would that include? [repeating question].**

Rolf: Well money in the first place, otherwise…

**Q: But more specifically.**

Jeffrey: Continuity of care, it would be nice to be able to see the same, if not the same person, at least the same two or three people consistently over a long period of time.

Glenice: I’m happy with what we’re getting, I haven’t been in and out of hospitals a lot through my life thank god, but I was just surprised how different they are now. They were just so quite kind and respectful, that was the thing for Geoff and I. I think you need a bit of substance sometimes, all our family are interstate, so we don’t really see them much. We’re having new issues at the moment with Centrelink like everybody else does and we just feel frustrated.

Etan: If you want to die quickly go to Centrelink it’ll kill you.

Glenice: We feel frustrated at the moment, I hope <inaudible> god, god will look after please.

**Q: So Centrelink you find frustrating, but the actual hospital itself is good, so you wouldn’t make any changes. Anyone else if you were to design a perfect health service what things would you do?**

Etan: I just make it flexible time again with diabetes clinic, because that’s where I’m finding frustration, able to get different times to see people in the end. Trying to work if you enlarge the system you enlarge everyone else at the same time, which is being more flexible and more contact, able to contact the same person. So able to ring the hospital and get onto someone yeah.

**Q: Anyone else got any other comments or suggestions? Just to give you some ideas what other people have said, so other people have said things like better education or cheaper parking or better continuity of care or seeing the same doctor. Some people have mentioned a combined diabetes and renal clinic, so they knock all the appointments for the diabetes and renal in the one day. Other people have mentioned services coming out of them when they're getting hemo dialysis, things like that. So what other factors will people want if we’re going to design a perfect health service which will meet all your needs in terms of diabetes and chronic kidney disease care?**

All shake heads

Andrew: Parking is a universal problem, not a health problem. I don’t think no matter how much you mention parking I don’t think anything can be done about it [etan laughs]. Look at the airport what they're doing with the parking there, it’s a money spinner and I suppose they do it because it generates money for the hospital or the whatever. So it won’t come down.

Etan: Since I’ve left Hospital 1 parking, Hospital 1a is real easy, Hospital 1a just happens to have a wonderful system and you get a card and you get a special area, so it’s not an issue for me. It’s a bit of a cost, but it’s not as much, whereas Hospital 1…

Rolf: $7 for a pensioner to park.

Etan: For a whole week, I get it for $20.

Rolf: No not for the week, just a couple…

Etan: I find Hospital 1a parking I pay a monthly fee of $24 and it lets me in for all the time, any time I like, whereas I don’t find that at Hospital 1. It is very hard to park, but again it’s just the area. No matter how big, I know they're putting extra car parking spots and the more they put in it’s harder to get, so I can’t see. I do park down there, it is very expensive. I usually park outside, as long as I get in before 10, as long as you get your appointments after 10 and before three o’clock you ride out the, because…

Rolf: Well when you're a renal person and you come here for service, you pay your ticket for $2, so is $6 a week.

Etan: I used to come in the old days you used to park out the back there.

Rolf: And you have your parking in the back from Hospital 1 there is a big car park just for disabled car park.

**Q: Any other things that people want to mention?**

Jeffrey: If you're tied into a hospital it’s easier if they have supplementary, you have Hospital 1a, Hospital 1bs, but they're all eastern. If you come from the west, access is strenuous and limited basically. Hospital 1 offer nothing across the river basically and it’s unfortunate, but again reality.

**Q: Someone else has mentioned if they can localise care they would like that.**

Jeffrey: Well if you're coming in for an appointment it’s a long way to come for a 10, 15 minute appointment, but again options.

**Q: Any other factors, what about any of the other things that I mentioned, do any identify with them, like having their diabetes and kidney disease appointment on the same day.**

Rolf: Yeah that should be…

**Q: Or even in the same clinic or having people come out to them when they have hemo dialysis, so having services come out, so when they're dialysing, things like that? Anyone think those would be a good idea?**

Rolf: That should be changed yes, that should be come in one day or another day when you don’t dialyse. The best is say in the afternoon, like the renal got twice a week and when you dialysis, so the renal make the appointment in the afternoon. And that is quite convenient, well after you finish renal you go down and have a coffee or something and then go up and see the doctor. I am a diabetic like you said there’s a problem. See we come, myself I’ve been happy to come in the morning seven o’clock, I go home about 12, then I still got something from the day. When you go in the afternoon, first you get all the traffic in the afternoon when you go home, you get all the working traffic, it’s inconvenient, that’s frustrating to go through that bloomin traffic.

**Q: So guys anything else if you were going to design a perfect health service which would meet all your needs, anything else that you would include in it? So mentioned some stuff, anything else?**

Rolf: What can you do?

Etan: The only thing is the 24 hour hotline to ring someone and the nurse on call so can help you with issues, rather than coming in, making a quick call in the middle of the night. I find most times people get sick in the middle of the night and you try to get onto somebody about anything, you have to wait usually the next day. So just an opinion, something on 24 hour call, like a nurse or something. You’ve got that anyway if you're a private Medibank or something, you can ring them.

Geoff: The renal unit do offer that.

Etan: Renal, as I said asking for assistance, I always found during the day most of the time, after midnight you’ve got a bit of an issue.

Jeffrey: No you just ring the ward, I’ve had no issues, because the machines operate overnight and that’s when our problems occur, you ring the ward and generally if they can’t…

Geoff: There’s a special number goes straight into the nurse’s station, I think it’s 5000. If I touched the end of my wife’s catheter the first instruction they give us is ring and bring her straight in and we’ll attend to you straight away and remove the catheter and put a new one on. And they stress that point, every time we’ve been in here for training, that we have that number listed, it’s straight into the second floor nurse’s station and bingo, we just ring them and come straight in and they’ll attend to you. That’s the service we; we haven’t had to use it, but it’s there.

**Q: Andrew, CP anything you want to add if we’re going to design a perfect health system? So imagine if you became the director, we’re going to design a perfect health service which would meet all your needs, what would it include?**

Andrew: I was sick one day and we rang up the ambulance, they brought me in and I stayed overnight in the emergency and four o’clock in the morning they find a bed for me. That service was alright, I mean after all you have so many patients to look after it, you cannot complain so long you got a bed and be inside.

CP: See it’s very difficult, because we are dealing with a public hospital, in a public hospital there is no guarantee of anything. The people come from the public and everybody gets the same treatment. You can be, god almighty, but you’ll get the same treatment and in our case if you come as a patient who is receiving dialysis, they look after you straight away. You come to the emergency, doesn’t matter what time you come and you say you're on dialysis and they take you in straight away. I have always had the good fortune of being taken in straight away and that’s what they tell you, that if you're a dialysis patient and you come, if you're a kidney patient you come to emergency they treat you very well.

**Q: So it sounds like you’ve got nothing else you would add, you're quite satisfied with the current system?**

CP: No I don’t think, because like I said before we are dealing with a public hospital and there are certain things we have to put up with.

**Q: We are looking to improve, so that’s why we’re holding this.**

Rolf: I got by with insurance, I’m with HBA. I don't get any special service here for having the private insurance.

CP: You won’t, like I said it’s a public hospital, they can’t give you any special treatment.

Jeffrey: Don’t know whether it’s relevant or not, but access to information from the hospital can be a problem. The simplicity in accessing medical records; a very poor example recently is I went to a public hospital who actually wouldn’t treat me because I was a transplant patient at Hospital 1 having dialysis. And the problem was actually renal and the access to the information wasn’t readily available.

**Q: From Hospital 1 yes.**

Jeffrey: Yes basically it was put you in an ambulance and send you to Hospital 1, which I found absolutely ridiculous, because it wasn’t an issue that needed to be treated here basically. But the fact that the matter was is one hospital guard their own patients and two the information is very slow in transferring, especially if you have a long medical history physicians tend to not want to treat you until they know everything. And that can make it very awkward. Whether or not Hospital 1 have; not sure how their system works for retrieving medical records, but it’s obviously a long protracted process.

**Q: Anyone else got anything they want to add, CP you haven’t been saying much recently?**

CP: Yeah once I had a problem with my machine, it was not going to the next, so they record the number which is on the machine and one stage they told me your pro card is full.

**Q: Your?**

CP: That card which I insert in, the pro card is full, so that’s why it’s not going to the next, so I didn’t know what to do and then I came here and I met my coordinator and I told him this is the problem I had last night and the answer was the pro card is full, so you have to delete all these things and just empty all that and then continue. That was ok, so the facilities you get here in this country is great, you won't get all these kind of facilities anywhere else, whether dialysis, in Italy you come straight away…

**Q: Your country as in another country you won’t get all this?**

CP: Yeah you won’t get all these facilities in other countries, like New Zealand or India or any other place you go on a holiday and if you have an infection or if you have some problems and you have to spend your own money and things like that. We come to the hospital and you tell them there’s a problem and immediately they look into your problem.

**Q: Okay last Question..**

Etan: There is an issue, it doesn’t exactly relate, it does relate between the hospitals, between Hospital 1 and Hospital 1a, especially x-rays and I only found that out because it’s not to do with the renal it’s to do with the, I had a set of x-rays done at Hospital 1a, but because Hospital 1a is a private unit they have trouble accessing the x-rays at Hospital 1 getting Hospital 1a x-rays and stuff. So I found that very frustrating that I had to go through another set of x-rays to get done here, because I’d already done one a week earlier and I just couldn’t get it, because it’s a private. Yeah that would help save to me it’s cost, because it’s a private unit and they don’t seem to have access to it. And only noticed that again renal it’s much easier for me to get things done at Hospital 1a because it’s just closer, but when they wanted to, when I come here to Hospital 1 I just couldn’t access it. I think the same issue they had at the Hospital 1b, even though they're all supposed to be the one unit accessing to their files, I find that’s one thing I’d like to get fixed, only because they keep telling me it’s a private unit that does x-rays at Hospital 1a. Because it’s private it doesn’t link into the system, where if you get the x-rays done here, anyone can see, all your doctors can see it. Whereas if you're Hospital 1a you can only see Hospital 1a stuff, I couldn’t understand. Again that frustrates me, because being one unit, yeah.

**Q: One hospital yeah.**

Etan: Yeah but again it’s x-rays and it’s different issues just relating to that, because it wasn’t to do with renal, it’s to do with something else, but it’s part of the… It was just easier to get things done at Hospital 1a that was all.

**Q: Very last question, is there anything else that you’ve come wanting to say that you haven’t or anything that we’ve missed asking you guys? About the diabetes or kidney health services or experiences, is there anything else that people have come wanting to say that they haven’t had a chance to say or that I should have asked you and I haven’t asked you?**

Rolf: No not really, I can’t remember.

**Q: Jeffrey or Glenice or Geoff or Etan or Andrew?**

Geoff: We have a group called Baxter that supplies us with our supplies and they regularly ring us up and what do we need and we got you down for so many boxes, etc, etc. As I’m getting a load this Thursday, but they follow up quickly, they follow up and we get the supplies without any hassle and the drug comes and loads them all out in my carport and I take them and put them upstairs as I need them. But I’ve got to say that Baxter they're on the ball, they’ve given us very very good supply.

Glenice: Good quality control.

Geoff: We had an issue with a couple of the bags in the early stages and I raised it with them and sent the bags back, the bags were crimped too far and the fluid wouldn’t come out, so I sent them back and obviously they’ve sent me a letter saying thank you, they’ve looked into it and they’ve found what the problem was and we haven’t had that issue ever since.

Glenice: It’s handy to have engineering experience.

Geoff: But they are very very good at keeping in touch, same with the hospital here, we get a regular call from the nurse up on the second floor, Jackie Ellis, she keeps in touch with us, how are we going, how’s the reading, how’s the drainage, etc, etc. We don’t see her till actually August now. We were here in May, so it’s otherwise we’re getting the results that we didn’t know about, <inaudible> getting the results of blood tests.

**Q: Glenice anything you want to add?**

Glenice: I said it all, I get a little bit concerned, because if anything does happen to Geoff what do I do, because I’m going to be stranded? I feel what happens if the place expires.. I can’t get out of this, I’m strapped in. So these little worries go through your mind at times, so I had thoughts of… if anything… oh dear I have to go back down and get it off.

Geoff: We found the secrets how to get around it believe you me, but I must admit the staff here in going through the training book of what you do for your machine I picked up on a few little things that weren’t printed in the handbook and by following those few little things it made a hell of a difference and stopped us, pardon my saying so, bloody machine waking us up at night going beep, beep, beep, beep. And I realised what it was. And since then we’ve had peaceful sleep.

**Q: The other Jeffrey.**

Jeffrey: Question if I can, why the correlation between diabetes and renal?

**Q: I’ll answer that after when we switch it off. Anyone else got anything else they want to add about the whole discussion that we’ve had or any further points about the quality of the diabetes and renal health care that they’ve had or experiences that they want to share? Etan, Andrew.**

Etan: Just the seating occasionally just needs some maintenance on the seats that we sit on and the chairs, be there for hours. I found one tear once or twice, but mainly been fairly good, I found I had to change the knobs around, because it went up and one went down instead of gone up. So that’s more maintenance, but other than that yeah fine. Get different ideas of seats. I know they try all different things to make things as comfortable as possible, try all sorts of stuff, but I think it’s hard.

**Q: CP or Rolf?**

CP: No I’m fine.

Rolf :I don’t have much trouble with diabetics, so I don’t know anymore.

END OF TRANSCRIPT

**Focus Group 10**

Joe: So I’m Joe.

**Q: My name’s C. I’m running the focus group.**

Joe: Oh sorry

Enrique: My name is Enrique.

Mark: I’m Mark.

Chee: Chee

**Q: And today you’re Chee. <laugh> Awesome. So look, maybe we’ll just start with a very simple question first. I’d like you to think about the experiences that you’ve had with the diabetes and chronic kidney disease services here at Hospital 2. I know most of you guys are on – actually how many of you guys are on haemodialysis or peritoneal dialysis?**

Enrique: I am peritoneal.

**Q: Peritoneal. Everyone else is haemodialysis; is that right?**

Mark: Yeah.

**Q: So obviously you have some experiences with especially the kidney health services here at Hospital 2. So thinking about those experiences, what are some of the strengths of the services that you’ve received here at Hospital 2?**

Joe: My experiences have been very little in discussions with the health service itself, except for the actual dialysis treatment that I receive, and that is fine, but I do have some problems with the machinery that’s being used and so forth.

**Q: Any strengths? So when you say your experiences with the other services are limited, so you’re - - -**

Joe: Well, how far do you go? I mean to say, the service is talking about neuropathy for my feet. I have ulcers on my feet. My general wellbeing is being looked after by Dr. G.

**Q: So with the ulcers on your feet, you see the diabetes foot service here at Hospital 2?**

Joe: Yes, at the level five in the medical centre.

**Q: So just talking about all those services that you have received and the health that you’ve received, what are some of the strengths?**

Joe: Well the strengths are that I’ve got somewhere to come in one place that they can do it all for me, and I can often have appointments directly after dialysis, which because of my limitation to getting around, I’m not mobile by myself, that’s a big asset to me. Just recently I’ve had to use the dental service here as well. So I’ve got no complaints on those issues. To me they’re all a strength because they’re a group together.

**Q: And with the diabetes foot service, do you see a diabetologist, diabetes specialist, through that or is it just the podiatrist?**

Joe: Originally I was seeing the diabetic foot clinic at HOSPITAL 12 Hospital, but now they’ve moved an equivalent branch to that in the medical centre here in Hospital 2, so that’s where I attend now. I’ve got an appointment next week actually.

**Q: And who sees you there, just the podiatrist or does the diabetologist?**

Joe: The podiatrist.

**Q: Does the diabetes doctor see you there as well or is it mainly the podiatrist?**

Joe: No, I don’t see a diabetic doctor anymore, because my diabetes is basically self-controlled and my GP and with dialysis, they analyse my sugars and levels. Once a month they take a full blood count and if there’s any main problems they instruct me what to do, what medication to go onto. They change my Aranesp levels or iron levels and all that sort of thing. So that’s all been done through the hospital system.

**Q: What about everyone else though, in terms of the strengths of the diabetes and chronic kidney disease services here at Hospital 2? Thinking about your experiences, what are some of the strengths?**

Mark: I think my main area of the hospital has been in the dialysis services, and I think one of the biggest strengths is the depth of knowledge of the people there, the people there to help you. If one person doesn’t have the answer, then certainly they will go and ask someone else and there is that depth of knowledge.

**Q: Are we talking about the nurses?**

Mark: Well both.

**Q: Both nurses and doctors.**

Mark: Both the nurses and the doctors. I have also had some dealings with the chronic pain team. There’s someone I can go and call on if, you know. And it starts from the nurses right through the whole system.

**Q: Excellent.**

Chee: I was transferred to Hospital 2 since I discover I have to do the dialysis treatment. I had the basic diabetes for 30 years, when I was working in Bangkok, and then I transferred to Hong Kong. They declare I have diabetes and must go for the treatment of dialysis. So I did it in Hong Kong for six months and then I contacted Dr George in Hong Kong Hospital and I move back here, come back to Australia. I am an Australian citizen. So when I come back here, I go directly to the dialysis. I been here for a year and a half. They never mention my diabetes history. Actually I thought – what do you call – history and past to Dr George, right, but there’s nobody concerned my diabetes and I stopped taking the diabetes tablets. I always used to take diabetes tablets for three years and then it get worse to be dialysis. I don’t know the reason. Then when I have the dialysis treatment, if I have problem here, problem there, they will send me to like ultrasound, but if I have no problem there’s no – I don’t know whether I have problem or not. My diabetes still on. But I never been seeing any diabetes doctor so far.

**Q: What are some of the strengths of the services here for the dialysis? Anything that you find particularly good?**

Chee: Because they do that dialysis, they have to do the pin, needle.

**Q: Yes, a fistula.**

Chee: And then once they broken my fistula and then I have to go to operation, and this was six months now. After six months I think my fistula have something, here. I have a bubble now.

Joe: A bubble?

Mark: Oh they all do that.

Chee: Yeah. But usually you are here, where the needle is. See. Yeah, I know.

Joe: See, the camel humps.

Chee: But my fistula was very good. I done it in Hong Kong see, the private. When I come here they said it was stuck here. This was stuck, so they suggest me to go for fistula treatment. That six months now. How come from here? I don’t know. So I asked the nurse and asked Dr George just two days ago. He said, “This is normal.” But I saw every patient on the needle side. So I have to propose that go to see the ultrasound again. Not they propose to me.

**Q: So you obviously received health care elsewhere in Hong Kong and Bangkok. How does the health care here at Hospital 2 compare to Hong Kong or Bangkok?**

Chee: In Hong Kong I have to pay <laugh>. It’s extremely expensive. It cost me $750 a week.

**Q: Oh wow. For dialysis?**

Chee: For dialysis. Private. We are private hospital. You can go to the public, but the public you have to queue. Take time. So I done it six months there and my son suggest to me come back to Australia. So I give up everything to come. <laugh> I left Australia for 15 years.

**Q: So do you think the health care in Hong Kong is better or here at Hospital 2 is better or worse compared to Hong Kong?**

Chee: The service I think here is good. I no complain with the service. But here you have to ask what happen this, what happen that. Unless there’s a serious problem, then they will tell you, otherwise nobody will. The nurses only do their job. And I see Dr George every three months, you know. You see a doctor how long?

Joe: I find that if your nurse is not familiar to what your problem is, there’s always a doctor around that will attend. I mean, the fact that he’s got to travel from 4 North to the medical centre, it’s the same doctor so you’ve got to wait, but the doctors always seem to be able to investigate, you know. I’ve had several issues, when I passed out in the chair, and they’ve pressed the button and I’ve had 20 doctors around me within a minute sort of thing, because my blood pressure drops considerably during the course. So in that sense, I find that the treatment is very instant and very quick to respond to the medical needs. So I commend all of them for that. I mean, every patient is different and every nurse is different. They all have a different technique of putting in, they all have a different reason why they do it like that. So we have to accept what they say at the time. They’re looking after us and we accept what they say. Medical reasons we require a doctor. A nurse cannot diagnose the problem. She can only visually see what’s going on.

Chee: The nurse here, they are all different scale. I’m lucky, I have very good nurses, very punctual. Other nurse, I need the local. Because I was aware for needles since I was baby. I never take needles.

**Q: So you said you like the service better here.**

Chee: Yeah.

**Q: Why do you like it better here compared to Hong Kong? In what way is it better?**

Chee: Hong Kong is only beginning. At Hong Kong they don’t have local. They put the liquid. The cold liquid, and then put the needle. You aware of it. I have to say, in here the doctor come routinely every Tuesday or sometimes once a week, but the different doctor look after different patient. So mine is Dr George. So Dr George is a very busy man. He just comes to say hello and if you don’t have problem to ask him, there’s no reply.<laugh> And then my GP cannot tell me anything. I have a GP. He knows I am in the dialysis in the Hospital 2. He never gave me any suggestion or anything or send me to a diabetes specialist, and myself, I want tablets, and I don’t go to the doctor, give me one tablet to cure me. I don’t ask, so I was living like this.

**Q: How about the rest of you guys, what were some of the strengths in your experiences? What are some of the strengths of the services that you’ve experienced here for the diabetes and chronic kidney disease?**

Enrique: The hospital is excellent. I’ve had three operations here and everything excellent. The diabetes, I never in the hospital for diabetes. I got in the medical centres, they got a doctor. I showed you, I give you the name.

**Q: Dr Chan, yeah. He’s still part of the hospital.**

Enrique: The thing I find is I saw him every three months and every time nobody tell me nothing. So I think somebody have to follow more the patient. For three months, I just check myself and maybe the GP, but the specialist, I don’t see him for three months. This is wrong. Otherwise people here is excellent. The service in the hospital is excellent.

Chee: Yes, what you described is absolutely correct. The service and the treatment, this is I think one of the best hospital, Hospital 2. I didn’t go to other hospital, but I feel I’m very satisfied. But one thing is, I have joined the kidney transplant plan, and to join this plan you got to pass through all the tests, and I done this test eight months and still no find. That is what you say. They are, sometimes when there’s too many patient or, I don’t understand. I wait for eight months to be complete and still at last I still have some transaction not complete yet. Dr George tell me. I don’t know what is it. He didn’t say. They leave you alone. We have to go for ultrasound, go for heart checking, for 12, 10, different check testing, and I think I did pass all, but after eight months, no news and then I go to ask. He said to me, “You still have some test.”

Joe: I was just checking with Enrique, he was saying that he sees his doctor every three months. Is that your endocrinologist, your sugar doctor?

Enrique: Yeah. Yeah. Sugar doctor.

Joe: If you see them in an office, in their rooms, they only make their appointment for you every three months because they do a long-term sugar level. That’s what they do their readings from. They don’t need to see you unless you’ve got a problem, unless something happens. Like I was on holidays when I became a dialysis patient, and I was taking four injections a day for insulin for my diabetes, and all of a sudden I was hypoing every time. Every time I was dropping down I was falling, so I rang my doctor and I said, “What’s going on?” and she said, “Look, just regulate it. Drop your insulin level and see how you go. See me when you come back.”

**Q: So you rang your GP was it?**

Joe: Yeah, I was away in Coffs Harbour and I rang my, not GP, but my endocrinologist.

**Q: So who’s your endocrinologist again?**

Joe: It was Dr Ann Conway.

**Q: Oh it was. Yep.**

Joe: And she said to me, “See me when you get back.” So I went back, because I was on holidays, I was eating ice cream and this and that, things I shouldn’t eat, but my sugar levels were never going up. They were always dropping. So when I came back I saw her and she said, “Oh well, everything’s okay. We’ll decrease the amount of insulin.” In the meantime, I had to see Dr. G, because I had a urinal tract infection and when I went to see him, who is my kidney doctor now, he went off his brain because she should have picked up the fact that my kidneys had completely stopped working and functioning, and that’s why my insulin wasn’t controlling anything, and that’s when, that same week, he put me on peritoneal dialysis. So, you know, if you’ve got a problem it’s up to you to contact the doctors and then they will decide what’s going on, but you don’t wait for them to come to you. As my heart doctor said, he said, “I’m not going to come looking for your problems. You got a problem, you come and see me.”

Enrique: The problem is that diabetes go up and down every day or whatever, and if I got that problem they go too high or too low, I need somebody to tell me what to do. It’s not that they don't talk to me. Take it to units or put three more units. Driving, be careful. Don’t go out if you go more than five. But I need somebody to tell me, “Look, you’re this problem.” You go up in the diabetes, you go up in the sugar, because you’re eating too much, or whatever the reason. It’s nothing to do with the service. The people come when they have to come and everything, but I think they need a closer control.

Joe: Do you do peritoneal at home or in the hospital?

Enrique: At home.

Joe: That’s why. There’s nobody around you to help you.

Enrique: So I die waiting for somebody to help me.

Joe:	Yeah, but the fact is, in the hospital service we’re talking about the hospital service in itself, there’s always a doctor here. They do your bloods every day.

Enrique: Yeah, but I talking about diabetes.

Joe: Yeah, I’m talking about diabetes too.

Enrique: I no talk about that.

Joe: Every day I’m here they take a reading of the sugar level. It’s passed onto the doctor whether the potassium’s high or low, but at home you haven’t got that facility, you know, they’re not going to call into your house every five minutes. Whereas here, there is a team in the hospital which look after you automatically.

Enrique: I’m sorry, but I still have an active life. I driving, I in the street, I not in the hospital. I need somebody to tell me what to do.

Chee: I know what you mention. They send them for home dialysis. They should have a routine checking on him, not just leave him at home, you know, and he just live for instruction or direction.

Joe: There is a social welfare team here that can organise a nurse to come home to you if you’d just talk to them about that. They can organise a nurse that will come home once a week to check your levels and advise you what to do, because when I had my ulcers in my foot they were coming home and changing my dressing twice a week. So you just need to talk to your doctor, get the social team, and they will organise a nurse to come to your house for you. You just need to know where to put your fingers to do what. I mean, I’ve had a lot of experience. In the last three years I’ve been in hospital for nearly 17 months and I’ve had a lot of problems in a lot of different areas. Anyway, I’ll shut up. I’ve been talking too much.

**Q: So Mark, I’m observing you listening. If you’ve got anything to comment on what they’ve said, feel free, but otherwise if you can also make some comments on the original question on the strengths.**

Mark: I’ll start with the strength and some of the things that have been said that are the strengths. Each of these specialists have a team of interns et cetera, so you may not see the specialist but you’ll see part of their team and that information certainly gets back, you know, it feeds back and forth. You mightn’t physically see your doctor, but you’ll see part of, you know, which is a strength in this sort of system. I see a private endocrinologist for my diabetes and yes, I can ring him up any time I like, but he’s the only one I can see.

**Q: I’m sorry, who do you see again?**

Mark: I see a Dr Borkman at Eastwood.

**Q: That’s right, yes.**

Mark: And that’s fine. If I need something in between I can ring him up, but here it is this – I’m just not putting it in the right words – but it’s this team environment where there’s so many people to call. You can call on this home service team. I home dialyse and a nurse comes out to me once a month, but I have a hotline number and I can ring them up 24/7 if I’ve got a problem, either a physical problem with the machine or dialysing or something else that I think is related. I can contact them any time. That’s part of this service, if you want to use that description, that is part of the hospital. We can only come from a big group situation.

**Q: Have you got anything further to add from what they were talking?**

Mark: Not really. I think it’s been covered pretty well. I think we all at times need advice, and I think Joe hit it on the head. You just need to know where to find it. It is there.

Joe: They just need to know how to access it. But you need someone to tell you.

Mark: Yeah, and you can sit there and say, “I don’t know what to do”, but you know.

Enrique: I think we need Joe on the front desk.

Joe: No. It’s not that.

Mark: Get Joe’s phone number.

Joe: I don’t mean to be offensive. I wasn’t criticising you.

Enrique: No, no, it’s not offensive. It’s all right.

Joe: But I’m just saying, you know, when you leave hospital and you go home, the whole world changes again. Here, I was in hospital just three months, I got out a month ago, you have nurses coming and bringing you medication when they want. They come and wash you, they come and change you, they do everything for you. They bring you food, they change your bed. When you go home, who’s going to do it for you? You’ve got to do it all. You’ve got to remember to do all these things. They come and wake you up in the middle of the night and give you your insulin or your tablets. When you go home, I go to sleep, I forget. I don’t take it all the time. You’re not responsive to it, and you can’t stay in hospital, but I just find this is a five star hotel with water views in every room and service everywhere you want.

Enrique: And beautiful nurses too. <laughter>

**Q: Now this is a similar question. What do you find particularly effective about the diabetes and chronic kidney disease health services here at Hospital 2? If you’ve got nothing further to add, that’s also okay as well, because I know we talked about strengths, and this is what you find particularly effective.**

Enrique: I can’t talk too much, because I just depend from my private doctor. I don’t use the hospital.

**Q: But you use the dialysis here though don’t you?**

Enrique: The dialysis from the other hospital.

Chee: HOSPITAL 12.

Enrique: The HOSPITAL 12. They from there.

**Q: So peritoneal dialysis though, who do you see for peritoneal dialysis? Is it here or in HOSPITAL 12?**

Enrique: No, in HOSPITAL 12. HOSPITAL 12 all the time. Because they got the classes there to teach how to use the machines and everything. So from there on should depend on them.

**Q: So whenever you need help for peritoneal dialysis you contact ..?**

Enrique: HOSPITAL 12.

**Q: HOSPITAL 12.**

Mark: Got to remember that the two units are co-joined.

**Q: Yes, exactly.**

Mark: Right. So it’s really all part of the same. We talk about Hospital 2 and HOSPITAL 12, but with dialysis anyway it is a joined system.

**Q: Yes, they are.**

Mark: So we’re talking about the same thing.

**Q: Yes, we are.**

Mark: Just at a different site.

Enrique: I didn’t know.

Mark: Yeah. They’re joined together. They do the training at HOSPITAL 12, but it’s still the same doctors, it’s under the same control.

**Q: So you can still speak Enrique if you want. So what do you find particularly effective?**

Mark: For me, because I’m at home now, I find the home service, the ability to talk to someone at any time, this contact nurse that you’ve got, you know, who will come and visit you if you want, or answer your questions, or sort things out. I find I haven’t had to use it much, touch wood, but the fact that it’s there and you know that even at two o’clock in the morning if you have a problem cannulating you can ring up an on-call nurse to guide you and to give you some advice.

Enrique: I come from a third world country and I can tell you that with this disease, I was dead.

**Q: Sorry, if …**

Enrique: I was supposed to be dead. So that’s the difference they do this country to the people. I am very disappointed that not many people come to this meeting. I don’t know why people are not interested in this.

Joe: I find effective in coming to the hospital and the service that we get, because initially when we were first introduced to what dialysis was and given the option of having peritoneal dialysis or haemodialysis, it was explained to us if you do haemodialysis you’ll meet people and friends and everything else. You do peritoneal dialysis, it’s on your own. The advantage I found with my peritoneal that I had for 11 months, was that I was able to travel anywhere I want to. I used to be able to do peritoneal dialysis in the car, in my boat fishing. As long as I had hygienic equipment with me and be able to wash my hands, I was able to do it wherever I want. I put together my own kit with all my solutions and everything that I need, with paper towels and everything in. I was nearly going to market it. And I found that it was easy to move around. I’d ring up the Baxter team and they would deliver the number of boxes and the equipment I would need to whatever hotel I was going to. No problem when I got there, it was always there. I carried enough in my car with me travelling, but it was a lonesome thing. At home when you’re sitting in that chair, because you’re doing it four times a day seven days a week, and it only takes an hour and a half, but still, it’s the preparation. You’ve got to sit down there and do that and it sort of restricts your movement through the course of the day. It’s not as if you can, oh I’ve got to come back and do dialysis. You can’t go out for six hours. You’ve got to come back and do your peritoneal. However, with haemodialysis I found that you come here and the service is all here for you. Everybody knows what they’re doing. I don’t like the machine that I’m on, because I’m on a series 5000 machine and I find it a problem, because this a training hospital. Hospital 2 is a training hospital, and unlike the medical centre, here in 4 North, there’s a lot of student nurses learning the system and so they’re constantly standing there explaining and the machine is very sensitive and annoys the crap out of me to be honest. But there’s a lot of friends. I come here, it’s effective because there’s friends when I come here, you see the same group of people, you have a chat and everything else. They bring you a sandwich and cup of tea and everything and it’s just nice to come. I find that it doesn’t bother me that I’ve got to get up to come here because it’s my life. If I don’t come, that’s the end of it, you know. People say to me, “Oh, are you still doing dialysis?” and I don’t know how many times I’ve said to them, “Dialysis is the rest of my life. I can’t live without it.” And as you say, in your country over there, many places, I was told by the Filipino nurses here that in the Philippines they give you the tube in the beginning of the week and you use the same tube over and over and over, whereas here, every day it’s a fresh sterilised system. So this country does offer that. Although the government says that we’re behind and here and there and everything else, we are far ahead than even America, because in America it costs a lot of money. If I travelled outside of Australia, I can’t plan to go overseas because it would cost me, it’s about $1,000 per visit to have dialysis done. That’s $3,000 a week to travel. Like, I can go to England, that’s fine.

**Q: Chee, do you want to say something?**

Chee: They always push us to go for the home dialysis, because they can leave the seat for other patient, but I will explain to them, I’m single. I live alone. And I am in fear of needles. I can’t do it, you see. And I know once I go in the dialysis treatment, like Joe said, it’s life. That’s why I was delayed one year to do dialysis. They told me for three and a half years ago, I do the fistula first and wait for one year and then I say, “I can’t stand it.” I had to surrender to dialysis. The home dialysis, I mean, one time my nurse offer me the, what do you call?

Joe: Buttonhole.

Chee: Buttonhole, yeah. Buttonhole. Because I don’t want to see. Later on I go to bed. So I done it for four months. No, no, I done it for nearly two months, buttonhole, and suddenly they announced there’s an infection. No good, so they cancel. I just enjoy my buttonhole for six, seven, eight weeks. I was very good. Because the buttonhole is only one line, two hole regularly. You can do it by yourself later on. Then they stop me. I told Dr George I am doing the buttonhole and he said, “Good.” Then after six weeks, seven weeks, yeah it stopped. They said this is something not good. To do this you must do it by yourself and if you agree to do it by yourself, they can allow, but how can I do it by myself from the beginning? They should give me a chance. We got a patient, what his name?

Joe: Jack.

Chee: Not Jack. The other one. He is doing his buttonhole in the hospital. He’s still doing it. Why they said I have to go home to do? I have to go home. And that’s another patient was still be here for one year in the hospital.

**Q: Thanks for that. Let’s move on. This is a similar sort of question. Perhaps some of you guys might have answered it already. What impact has attending the diabetes and chronic kidney disease services made to your health? What impact has attending the services made to your health?**

Enrique: It changed my life because as soon as they find out that it was diabetes, everything go down. So from then on my life change completely. You have to check what you eat, what you drink, what you use dialysis, everything changed.

**Q: So I’ll rephrase the question. What impact has attending the diabetes and kidney services made on your health? So is it a good impact or bad impact? Has it improved your health or made it worse or kept you alive?**

Mark: Certainly, yes, it’s kept me alive and yes, it’s improved my health dramatically. It’s taken a long time, but without going into other complications, it’s certainly made a huge difference to my ability to get around to do things.

**Q: What about everyone else?**

Joe: I find that, yeah, it’s my life. I’ve had to understand and accept the fact it is my life that I’m sort of looking after by doing dialysis. It’s a great thing, but it has impacted on me in other areas. I can’t take off the required fluid that I need to because my blood pressure keeps dropping. I have very bad low blood pressure. It’s even dropped down to 68. And anyway, that’s another story, but from my point of view I can’t do anything else. I’ve got a lot of other pain, a lot of other problems, heart problems and so forth, but as far as the dialysis is concerned, it is keeping me alive, but my whole day is just, when I go home I just sleep, because I pass out. I don’t see the change that it’s making in me, except the fact it’s keeping me alive. I have not passed urine for three years. Everything I eat or drink in fluid content remains in my system and I find it hard that I’m on a restricted level of fluid. It’s a problem that I have personally, but as far as the reason why we’re here, is to keep us alive and that we’re all in the same boat. I mean, but it is hard on your family at home, you know. They’ve got to wear the consequences. You know, can’t plan to go out on Saturday night anymore because I come home from dialysis and I’m wiped out. It wipes you out. That’s how I find it anyway.

Chee: Dialysis can give us, for a weak person they can give you feel alive again. But for me, I was a healthy person and I was weak when I have the problem, you know. When people first got the disease of the kidney, they have a bulky body, because they have too many water. Dialysis take out your food. So as soon as I took this I become slimmer, but every day I cannot do anything. I’ve had to give up all my hobbies because when I come in the morning for prepare dialysis, my blood pressure is 180, 190 and after finish I drop down to 120, 110 and then I feel dizzy, so I have to go home.

**Q: Feel dizzy?**

Chee: Yeah, dizzy. I have to go home. I can’t go out because you just feel you need a bed. So I have to give up many hobbies. I can’t go for my social life, my business. Everything I stop. I become like a isolating person, because I don’t want to see other people when I feel dizzy.

Joe: It does impact.

Chee: And every second day I have to go for the dialysis to get your life back. The other thing is I can breathe again. I can have one day after dialysis, feel good. That’s only the second day. So one week probably, for dialysis person, we have three days normal, but the other three days you can’t do much.

**Q: For four days.**

Chee: So dialysis really keep me alive, that’s like you say Joe, but it doesn’t give you pleasures.

Mark: I know this is a little bit off the subject, but in relation to that, home dialysis is great because you can do it around other things. I did it last night at seven o’clock at night because I’d been out for dinner, and slept till one o’clock, got off the machine and went to bed.

Enrique: What sort of dialysis you have?

Mark: Haemo.

Enrique: Hey?

Mark: Haemo. I put my own cannulas in, set the machine up. It’s all sitting at home. There’s a big water filter on the side. It takes up a whole room. I got a delivery yesterday of supplies of 84 boxes. And that’s the difference, you know. That’s why I say for me it’s improved my life.

Joe: I would love to do home dialysis in that sense.

Mark: I realise it’s not for everyone, and that’s why I can say, when you say it’s keeping you alive, yes it’s changed my life and it is keeping me alive, but now with the next step, which for me was the home dialysis, not everyone can do it. It’s certainly given me my life back. I run my own business. I work when I need to work and I fit the dialysis in in between. I worked on Friday night. I got home at 4:30 in the morning. I got straight on the machine and slept till lunch time.

**Q: On the machine?**

Mark: Yeah. Well, in the chair, but I just fall asleep. I was tired, I’d worked all night, but then I had my afternoon. You can’t do that here. You’ve got to come when they tell you. And maybe that’s why I see it a little bit differently.

**Q: Let’s move on. So we’ve talked a lot about the strengths. Let’s move onto weaknesses or things that could be improved. So let’s move onto your experiences and what would you say are some of the weaknesses of the diabetes and chronic kidney disease health services?**

Mark: Well, I’ll speak first. I don’t want this sort of thing to sound critical, because it is a very good service. But anything can be improved.

**Q: Yeah, of course, and that’s why we’re here.**

Mark: And Joe touched on it. Yes, this is a training hospital and even in the home training centre, everybody does it differently. There’s no sort of standard way of doing all this stuff. Even the home trainers, there’s four home trainers, and every one of them tells you something different. The same, but different.

**Q: Does that become an issue?**

Mark: Well it does, because nurse A doesn’t know what nurse B did because they did it differently to nurse C. It’s more in the actual delivery area that I’m talking about. I’m not talking about the doctors, I’m talking about the actual delivery of the dialysis. And, you know, nurse B comes along but nurse A has done it differently, so they aren’t quite sure where they’re up to or what they’ve done or whatever. You know what I mean? There’s no sort of standard about how this service is delivered. And it goes back to nurses are trained in different hospitals and they’re taught in different ways, but I would have thought in a situation like this that they would have had some sort of protocols and standards on how things are done.

Joe: Exactly.

Mark: Like I said, I don’t want it to sound negative because they’re still a damn good service.

**Q: Yeah, no, no, no, that’s fine.**

Mark: Do you understand what I’m saying?

**Q: Yes. I think so. So can you give an example?**

Mark: I’ll go to the home training, because that’s where I’ve been most recently. One nurse will tell you you’ve got to do this, this and this and then another comes along and says, “No, you do it this way. When you set the machine up, no, you put that hose on before you put that tube on. You put that on before that one.” But everyone does it differently.

**Q: So it leaves you a bit confused?**

Mark: Well it does till you sort of work out for yourself how to do it. And I encountered it here. I was in hospital when I came here for some time, even though I self-cannulated, and I watched them. The machine is set up differently, this is done differently, that’s done. And if you’re trying to learn, that’s a bit hard. And even if you’re not, I’ve noticed where, you know, nurse A’s had to go and see nurse B because they’d started and where are you up to, what have you done, whereas if it was a standard you’d see exactly where it’s all up to. Make sense?

**Q: Yeah, yeah, yeah.**

Mark: But again, I’ll stress, it’s a fantastic service, but it can be improved.

**Q: Any other weaknesses?**

Joe: Just adding to what Mark said, and as I touched on it before, it is a training hospital and as I said, every patient is different to cannulise and every nurse is different the way she does cannulise. Example one was yesterday morning when I dialysed, the nurse come along, new nurse, first day here, and senior nurse is trying to train her the machine, and I’m a series 5000 machine, not the normal machine, because I’m doing a survey over a long term. Supposed to improve everything for you but I don’t see that. It’s a very sensitive machine and it takes a long time to set up. But to cut a long story short, I normally have both needles going up my arm when they cannulise and yesterday the nurse said, “No. Put one down and one up” and she left it for this new nurse to cannulise me. Now, I don’t take local so I suffered the pain that comes with putting the needles in, however they crossed over the needles and when they tape it up and everything else, you’ve got one needle up and one needle down and you’re putting pressure on one to stop when they de-cannulise you, and you’re putting pressure on that other needle and it’s killing you. Nurse A will come along and the biggest problem I find, once again not criticising what they’re doing for us, but you come here, you’ve got to wait till 7:30 to go in. But then all the nurses come and they’ve got to do a – I don’t know what they’re handing over in there because when we come in the morning we’re the first group. So there’s nothing to hand over, but they make us wait and wait and wait. They’re here doing a job, they’ve got to be here, but we come here and sometimes it’s not pleasant to have to come and wait. And the longer they delay you, the worse it is, and then they start talking for half an hour. At least cannulise you, put them on first, then do their talk, you know, because it’s just common sense. Everybody complains. You stand in the hallway or the waiting room, everybody complains about the same thing. People I talk to, they complain because you’ve got to stand there and wait. Now I’m not talking about the lack of nurses sometimes. Yesterday there was only two nurses cannulating nine people in there. I can understand that. It’s the time wasted in between. It’s just the time wasted in between trying to cannulise, you know. And they had a system at HOSPITAL 12 that any four hour patients went on after the six hour patient or the five hour patient, then the four hour patient. Here, no. They put a four hour patient on first and a five hour patient’s got to wait. The system at HOSPITAL 12 was a bit better than here.

**Q: You’re nodding vigorously Chee.**

Chee: Well I agree with what Joe’s saying. I’ve been to 4 North. You come there at 7:30 and they still not let you in.

Joe: Yeah, eight o’clock they put you on.

Chee: That’s all right then. I only take three weeks and then I say I want to transfer to Satellite.

Joe: Yeah, Satellite they open the door at 6:30.

Chee: And Satellite start at 6:30. Sometimes nurse come early, we just go in early. Sometimes 6:15 we can go in. We set up our own chair, everything and we’ve got enough time to go home early, not like the 4 North. Because in 4 North all the senior I think. So everybody’s the boss. When I come 7:30 sharp they still wait. Go to the sitting room.

Joe: Yeah, the waiting room.

Chee: I have that experience, yeah. But never happen in the Satellite dialysis. Satellite dialysis like home. Everybody come home.

**Q: Any other weaknesses people want to mention? So in other places they mentioned things like waiting times, too many appointments, conflicting advice, or from the doctors? Can any of you guys identify with that?**

Joe: I’ve got no problem with the doctors, but as I just said, the waiting time was what I was trying to indicate that that’s the biggest problem. Everybody complains about the same thing, and there’s no protocol, as Mark said, in regards to the system. They’re all different, you know. We have one senior nurse here, I’m not going to mention names, she’s a stick in the mud. She’ll make you wait behind that door right to the last minute and even beyond, and you come here when she’s not on duty and the other nurses will let you in and be a bit tolerant. I mean, when we go in, we were taught at HOSPITAL 12 to set up your table, to get your tapes ready, to open your things so that the nurse could come from one patient to the next and it’s all ready to cannulate. Here, there’s one nurse, “Don’t touch that. You’re not allowed to do that”, and then another nurse. So I ask of a morning, “Who’s my nurse?” to find out what I can do, because I’m not going to get into trouble if I’ve got the other nurse sort of thing.

Chee: The difference now in Satellite, they train us to set the machine, check the chair. They don’t do anything. That’s good. We can learn.

Joe: Yeah, go in and do it.

Chee: But in 4 North, you cannot set the machine.

Enrique: I can’t make any opinion, because I do it at home.

**Q: I know. You do peritoneal don’t you?**

Enrique: Yeah.

**Q: So other people have mentioned also waiting times for appointments to see doctors. Some patients also complained of poor communication by the doctor to the patient or between doctors, or some people also complained about inappropriate education or lack of education. Can anyone identify with any of those factors?**

Chee: Maybe Joe can have no problem with the doctors because he probably is serious patient. So they must see you first. But for us, you make a problem, wait three months or two months, you know. Like I have tried, I went to the fourth floor, fifth floor, the doctor I see him once and then I said, “Can I see you, appointment at another time?” He said, “Three months.”

Mark: Maybe I’m a little lucky. I’m fortunate I don’t have to see the doctor all that often. But again, I can contact him any time through his secretary. I can ring him up and he’ll always ring me back or get her to ring me back, you know, if I tell her the problem, whatever, if it’s something simple. This is Dr. J.

**Q: Dr. J, yeah. And you see him publicly here?**

Mark: Yeah, I see him publicly. But I can ring up his secretary, Magdalena, and say, “Look, you know, I’ve seen him for a month, I’ve got this problem”, he’ll ring me back.

Joe: Is that Dr G?

Mark: No, Dr. J. He’ll ring me or get her to ring me back and say, “This is what you need”, you know what I mean. Use her as a mediator to save his time. But he’s always contactable.

Chee: Dr. J, is he Chinese?

Mark: No, he’s Indian.

Chee: Oh Indian.

Mark: Yeah, young Indian man. Very good. But on the diabetes side, I see a private endocrinologist. Same thing. I can ring him up.

**Q: Enrique, have you got anything to add to identify with any of those weaknesses?**

Enrique: No, because as I say, I don’t come to the hospital. Don’t do nothing. So I just do it at home.

**Q: So your diabetes and kidney disease, who looks after those?**

Chee: How long you been dialysis?

**Q: So Enrique - - -**

Enrique: I’m sorry?

**Q: Who looks after your diabetes and kidney disease?**

Enrique: Dr [Whitlam] *0:53:50.8.

**Q: Dr Whitlam.**

Enrique: *0:53:53.5 Whitlam.

**Q: And where do you see him?**

Enrique: Every month.

**Q: Where, where, where?**

Enrique: I see him here before in the medical centre.

**Q: Sorry guys, can we …**

Enrique: I saw him from the medical centre, but now I move houses so I saw him in Kempsey.

**Q: And then your diabetes, again sorry?**

Enrique: Diabetes, the doctor I tell you is here in the medical centre.

**Q: Dr T isn’t it?**

Enrique: Dr T.

**Q: So when you see any of them, did you have to wait a long time to see them or anything like that, or any issues?**

Enrique: Dr T is every three months, but Dr Whitlam is every month. I do a blood test before I see him every month.

**Q: And there’s no issues with waiting in the waiting room for a long time or anything like that?**

Enrique: No, no. No.

**Q: What about education or not enough education or inappropriate education, where like, you know, they’re not giving education in a digestible fashion? Like there were some people from another hospital sort of complained that someone just gave them a – and said, “Here read this” and it was like a massive book and the language, they found it hard to read. Any of you guys have an issue with that with regards to education, with regards to your kidney disease or dialysis or diabetes or anything like that?**

Enrique: On HOSPITAL 12 they have four people there. Any one of the four people can tell you exactly the same. They do the same direction to the things and they are on the phone all the time. So they are excellent there.

**Q: Oh, do you want to ..?**

Joe: I was going to say something, but it’s slipped me now. I forgot what the question was actually.

**Q: That’s okay. When it comes back you can mention it. Now, the other thing was, some people also complained that they have too many appointments, they were seeing too many doctors and that each doctor sort of worried about their own bit and no one was pulling it all together. There was a lack of holistic care. And sometimes when they asked one doctor about a certain question, they say, “It’s not my problem. See someone else”, or they might ask the dialysis nurses the issue and they say, “Oh, see your GP” or something like that. Does anyone identify with those weaknesses? No. No one.**

Mark: I can think of instances with other people. It hasn’t happened to me. But I can understand, you know, you say about see your GP, see this, see that. I don’t see that as a weakness, I see that as if it’s the nurses, they’re here to help you with your dialysis, not with the cut on your finger or the fact you’ve got a headache or whatever, whatever, and I can see that sometimes they get snowballed with, you know, someone comes in with dialysis and they bring their whole bag of tricks with them of problems. But I suppose I find my renal specialist sort of pulls all this together. I see the dietician, I’ve been going to the same clinic, but he’s the one who pulls it all together and gets all the information from these people and then explains to me what it all was, you know.

**Q: How about for other people, do they have someone, a certain health professional who pulls everything together like Mark was talking about?**

Joe: Yes. Prior to meeting Dr G, through the years I picked up 12 different specialists. When I went to Dr G he said I had far too many and it was best if I disposed of a few. So he worked out which ones and I think I dropped four, and he said he would look after everything. So I see him on a regular basis, at times six weeks apart or two months apart, but the thing is he’s sort of co-ordinating looking after that. However, along the way now I’ve picked up heart specialists and everything that I didn’t have before and these people are essential to my wellbeing. I’ve got to see them. Tomorrow I’m dialysing in the morning and at two o’clock I’ve got to be at Burwood to see the heart specialist. So this is happening constantly, all the time with me. I’ve got double appointments every day. I’ve got diaries and calendars blown up full size so I can list the number of appointments. This week, today coming here, is the sixth appointment I’ve seen in the three days.

**Q: What do you guys find frustrating about the diabetes and chronic disease health services?**

Enrique: With the service there’s nothing. Diabetes is frustrating. But the service is good.

**Q: Yes, and the care is okay, yep. Anyone else? I’m talking about the care and the services.**

Joe: The service is great, it’s the individuals that carry the service that cause the problems. I mean, what we’ve got offered in front of all of us by doctors and that, but every person is different, and this is what I find the biggest problem. Nurses can make your day or they can break it, because I don’t know how they come at you and approach. I know we’re just another patient to them and that’s how they’re taught because they have to. They’ve got compassion in some ways, but the biggest problem is they all treat you differently, and that’s what I find is a big difference, you know. And I said earlier that we come here, yeah, we talk to our friends all the same but you can’t talk to the nurses in that same format. You don’t have that. They’re doing their job and that’s what they do.

**Q: Just in the interest of time, I might move on. Let’s move onto thinking about your experiences now with living with the disease; so diabetes and chronic kidney disease. In your opinion, what do you need or what do you have in place to look after your diabetes and chronic kidney disease well?**

Enrique: Well, we depend on the doctors to help us to work out with everything. We check with the doctors to see what to eat or what to drink or what sort of medicine you take.

**Q: So you’re saying support from health professionals?**

Enrique: Yeah, exactly.

**Q: Anything else?**

Chee: We rely on the nurses to look after us, but there is no education or any guideline to teach us how to keep your dialysis systems. And if we don’t give enquiry, the patient don’t make the enquiry, there’s no response to you. But the good thing is the hospital, once you have any enquiry, they will respond right away. They send you to the doctor and the doctor arrange you other procedures. But if you’re a quiet person and you don’t understand, sometimes there’s no response for you. They will not come and say, “How is your visit? What happened?” They don’t come to ask you this.

**Q: So what you’re saying is you need to have a certain understanding of your disease and also have a certain level of assertiveness as well.**

Chee: Education.

**Q: Is that right?**

Chee: Yeah, exactly.

Joe: Can you say the question again please?

**Q: So the question is, in your opinion what do you need or what do you have in place to look after your diabetes and chronic kidney disease well? So I think Enrique has mentioned support from health professionals. Chee has sort of mentioned, you know, perhaps knowing something about your disease and be willing to ask. Other people mentioned things like good education or having motivation or having support from family. Some people even mentioned having finances. So what is important for you guys to look after your diabetes and chronic kidney disease well?**

Joe: I find – gone from me again. I lost the question again. I don’t understand that. No, I can’t relate to the question again.

**Q: Do you want me to say the question again?**

Joe: Yeah, please.

**Q: So in your opinion, what do you need or what do you have in place to look after your diabetes and chronic kidney disease well? So what’s important?**

Joe: What’s important to me is the fact that we come here to dialyse, because that is the only thing that we can do. There’s nothing else that’s going to prepare us or treat us for the rest of our life as dialysis will, and without it, we’ve got nothing and with it, we’ve got life but we’ve also got some disappointments in life, as I said earlier. After dialysis I’m totally wiped out. I can’t do anything. I go home and I sleep. It affects the family, because we can’t plan to do things from one day to the next because it depends how I feel. I’m dependant because I can’t drive and everything else. I’m depending on everybody else to do things for me and it’s terribly frustrating. And that’s all I can say about it.

**Q: So other people did mention things like family support or being able to adjust to the disease.**

Mark: I think support is critical. I’m very lucky. I have a very good wife who’s very helpful and very supportive. I think what most of us – well, from what I can pick up from hearing even myself – is a better understanding of what’s going on, you know, some sort of training of some sort so we can understand why you go home feeling like rubbish. I think most people can accept things if they know what’s going on.

**Q: So you’re saying that you probably need more education or more training. More explanation.**

Mark: I think explanation in simple terms that you can understand, and I think that’s important to know what’s going on with yourself.

**Q: Of course.**

Mark: Both with diabetes and with the kidney disease. Enrique was saying his blood sugars go up and down, you know, why? Unless there’s some education, you know, I’m probably a bit lucky. I’ve got a thing call the internet and I read a lot and spend a lot of time sitting on that machine with my computer in front of me. But not everyone does or even understands. Some of it’s all gobbledygook. But some simple education as to why things are happening helps you to understand and I think that that is something that is lacking. It’s, you know, this has happened, okay, well what’s the outcome of that? What do I need to do to minimise that? Does that make sense?

**Q: Yep, yep, yep. I guess linked in with that is having some sense of motivation as well to look after your own health, which is important. Not everyone has that.**

Mark: But external motivation helps. People to motivate you makes a big difference.

Joe: I do get a lot of family support, you know, I’ve got four children and a great wife too and I feel the frustration because I’m putting the pressure on them, depending on them to do things for me. Fortunately Mark can drive and everything else and he does his dialysis at home, so he’s got a bit more freedom. I’ve got to get to the hospital and get home and my wife at the moment is sick. I’m depending on taxis or community transport and it makes me frustrated that I can’t just get in the car and drive myself here and do what I’ve got to do. I would love to do home dialysis, but because of my vision they said I can’t have a machine. Okay, fair enough. And I’m not a candidate for a kidney transplant because I’ve got too many other problems in the system. But I can’t say enough, you know. A lot of people out there are unaware of what dialysis is. The number of kids saying to me when they talk to me, friends and family, “Are you still going to dialysis?”, you know. I’ve told you a hundred times that I’m doing it for the rest of my life. It’s not, I get a cold and it goes away in a couple of weeks’ time. People out there don’t understand what dialysis is. There’s a lot of people in the community that need to understand, you know, and when you’ve got several other problems, to carry dialysis is a problem. It’s a life threatening problem, and you’ve got other problems, you know, in my case I’ve got heart and a lot of other things. It’s difficult to address one, you know. People have got to understand that you moods change in a lot of ways, and I’m a perfect candidate for that happening. And Mark and I shared a room in hospital and he’s seen a lot of way that I behaved under certain pain and stress and everything caused a lot of problems. So I don’t know how you resolve that issue. I don’t know. Because I understand why my blood pressure drops because I’ve got to take out too much fluid. I understand why I’ve got the fluid because I drink too much, but you know, I’ve got no control. I came to see the dietician to help me and she sent me away and she gave me a list of things and this is what you do, this is what you don’t do. I need help. In the hospital you get it. At home I don’t get it, in the sense, you know, because your family are not always there with you. They go to work unfortunately.

**Q: Let me flip the question the other way. What things make it harder for you to look after your diabetes and chronic kidney disease well? What things make it harder for you to look after your diabetes and chronic kidney disease?**

Joe: I’ve got one word for it. Willpower. Strength.

Mark: Yes, you’re right, it is very difficult, fluid restriction, sugar restrictions, you know, certain food restrictions because of potassium and sodium and all the stuff that’s in it. It is very restrictive, and it’s all related around food, let’s face it, because that’s what causes the problems. Fluid and the wrong foods, you know. And it gets back to education, but it is difficult. It is a struggle all the time. You go out to dinner, oh well I can’t have that, I can’t have that, I can’t have that, I can’t have that. Bring me out a dry biscuit and a cup of tea please, you know. Oh make it half a cup of tea, I can’t have that much.

Joe: No sugar.

Mark: That’s right, no sugar. And only light milk, okay. We laugh, but I think we all relate to exactly what I’m saying, you know. It is willpower and that is what’s difficult. It is very difficult when it’s stinking hot and you’re thirsty and you have one mouthful and you’re dying to pour a bit more down. And with the diabetes it’s the same. Every now and then you let your hair go. You have to. Even my endocrinologist said, you know, “You’ve got to let your hair down every now and then.” It might take a little bit of rebalancing, but if you don’t, you know. Don’t go crazy, but if you go out, have a nice meal, have a dessert, whatever, whatever. I don’t drink anymore, but I used to, but have a glass of wine or something. Yes, it’s going to take a bit of rebalancing after it, but you don’t do it every week, you know.

**Q: Anything else you want to mention? So other people have mentioned things like lack of support or having trouble adjusting to the illness.**

Mark: Yeah, well that’s all willpower.

Joe: That’s willpower.

Mark: You’ve got to do it for yourself. No one can do it for you.

**Q: Other people have mentioned a loss of identity as well, because like, you know, they’ve lost their hobbies, they’ve lost their job and stuff like that.**

Joe: In the loss of hobbies and so forth, look I’m a cabinetmaker of 44 years and when I don’t dialyse every second day, as Chee said here, we got three days or four days that we’re good. So I go in my garage, I go round my pool and do different things. I have those things so I use them to my advantage to keep me occupied, otherwise I’d go completely crazy. My community service, car service, has a social outing as well, and I’ve booked on three coming up very shortly to go on social drives with them. We’re going to Ettalong and we’re going to different places, you know. So you look forward to those things. Unfortunately, last one I was meant to go on, that morning I fell over and broke my leg and finished up in hospital for three months. So I haven’t gone on one yet. But they’re the things that we’ve got to look forward to. Without them we’d be totally lost. So if you are interested enough to try to get away from your problem and put it behind you on certain days that you can, then it’s an advantage to you. You’ve got to be in mind to do those things, but it’s willpower when you go out there and you go to lunch and you sit down, you know. I unfortunately have a problem where my diabetes is bad, but my potassium level is worse <laughter>, and when I went to the dietician only two weeks ago with my wife – we sat there for an hour and a half – she said, “Forget the diabetes diet and just concentrate on your potassium. That’s more concerning to you because of your kidney situation.” I asked Sarah before, like I had a sandwich, I shouldn’t have. I didn’t eat the crust because it holds the grain. Potassium, you can’t have brown bread, you’ve got to have white bread. Diabetes, you can’t have white bread, you’ve got to have brown bread. <laughter> It’s so confusing. You can’t have vegetables. You can’t have fruit with potassium. But I’m just at a loss. I give up. I am seriously, in the past, have thought of my own life. That’s something I openly say here, and I can understand why a lot of people who are weaker do what they do, you know. It’s the same with children bashing. I can understand, people who are not strong – I’ve had four children and I can understand it, but you’ve got to have the will to understand what that child is also going through, you know. So it’s all come back to willpower and managing. Nobody else is going to do it for you and there’s no place you can go that they will do it for you. You can’t just walk into a hostel and say, “Listen, look after my weight and my diet for me.” I came into the world at 11 and a half pound. I came in big and I’m going out big. In the meantime, I’ve tried many things.

**Q: Other people have mentioned depression as being a barrier or making it hard for people with diabetes and chronic kidney disease. Some people also mentioned the illness itself, like you know, coming off the dialysis feeling washed out, or just having a lot of other illnesses, which impact on their ability to care for themselves. Can anyone identify with any of those?**

Enrique: Depression. Is another thing you want to do you can, the older you coming the worse they coming. The family to a certain point help you. To me, myself, I divorced, so problem the family go together with the sickness, push you down, way down.

Mark: Very much so. You feel like a burden on your family, you know. Oh I can’t go. My wife wants to go up to Queensland and see her brother for a week, so I send her. I can’t go, because you’ve got to dialyse. And you can arrange it. I realise that. But it’s not something you can do on the spur of the moment. These are examples. My wife went out for dinner last night. I couldn’t go because I had to dialyse, you know. So you do feel that these things are a burden. And one of them is the ability to support your family, you know. Their lifestyle is restricted because of your problems, and for that you do get very depressed and you get very upset and you get very down and you feel very frustrated and a burden on people, and it’s not necessarily your condition but it’s how it’s impacting on others.

Joe: That’s right. What Mark said is perfectly right, because I find my biggest problem at the moment, or since I’ve become a dialysis patient, is I’m unable to be in the same room with people who are talking, friends of mine who, they’re not doing anything wrong, it’s just I see it like that. They talk about going overseas and going on holidays and doing this and I look at my poor wife who has always wanted to go back and see where she was born, and she’s going to have to wait till I die because I can’t go with her and she won’t go without me. So, you know, that’s the burden and the pressures that one feels that we put on people. See, we carry the problem in the disease, but it’s the person behind us that wears the pain. That’s how I see it.

Chee: I live alone because I don’t want to burden my family. I don’t want to burden my friends. That’s why I live alone. I leave them alone so I can do myself. I don’t want to give them any problem, worries, you know. Even on dialysis they go, “What is dialysis?” But another way I lost a lot of hobbies like I said to you. I knew I never stopped this, stopped that. I eat everything. I do what I like since I start dialysis. I want my life. I don’t want to be just stay home, sulk, you know, boring and depressed. So dialysis person from the beginning, I think they’re all depressed. So firstly I also like that. Now I change. The second day off, when I take my meal I take everything what I like. I don’t just follow the list of what you should eat or what you should not eat. That is too much depressed, make you fall down, down, down. So thinking positive is the best way I think.

Enrique: I think that everything else here is depression, because you feel frustrated. You feel frustrated you can’t have – to go to Queensland because you had carry all the bags and the machine. So that the worst part. That keeps you alive. They don’t kill you but it don’t let you live free as a person.

**Q: Now it sounds, from what is emerging from everyone, is that the psychological aspect of the disease seems to be a really big factor.**

Enrique: It is.

**Q: Do you guys feel that you get enough support in that area from your health professionals or from the service, or could more support be provided? Like, did they adequately prepare you for it, did they talk about it?**

Joe: Nobody’s offered it to you. In all the discussions that we had, even leading up to introducing us to dialysis, nobody talked about psychologically what it would do to you. Nobody talked about the depression that sets in. I’ve been dialysing now for five and a half years. When I was at HOSPITAL 12, four friends that I knew, young and old, committed suicide by not attending dialysis because of their depression state, the way it affected them. And time goes by quickly, you know, but the thing is, it stays with you. You can’t get away from it Glen, you know. I’m here today because I wanted to be here. I was asked but I said yes I wanted to be here. But I’ve got to come tomorrow for dialysis. If I wanted to or I didn’t want to, I’ve got to be here, you know, because if I’m a responsible person to anybody else, I come because of that, not because of myself.

Mark: I think there is some issues, and it’s not just for the patient, it’s quite often for the carers and for their family. They’re the ones that don’t understand and they also get depressed and frustrated because they can’t do, you know, they feel obliged like Joe’s wife won’t go overseas because she won’t leave him. They feel obliged to change their life and put their interests on hold because of you, and then that comes back to you because you feel horrible about it. But there probably is and probably, as we say, if we investigate hard enough, there may be some sort of support for carers. I don’t know.

Enrique: Only last week, Dr Whitlam, the doctor that see me once a month, offer me a psychologic help.

Mark: Take it.

Enrique: So I already ask him to make an appointment for me, because as I say, that cost my divorce. My wife to support.

**Q: Do you guys think it should be offered upfront, some sort of psychological support?**

Joe: Sorry that I’m always first. I don’t know how they can offer you that support upfront on the individual. They’ve only just met you. They’re educating you, preparing you what you’re going to go through with dialysis, but they don’t know how your strengths are in regards to it. But it seems to me, I thought it was me. I’m not unique, but it seems to me everybody in this room has a similar thought of what I’m feeling and what I’m going through for the same reason. We’re all here for the same reason, whether we do it at home or in the hospital. We’re all doing it for the same thing, because that’s our life in our hands. But it grows around you. You know, my wife says to me now, you know, we’ve lost a lot of friends because of my condition, because I’ve been moody or I get moody, you know. People don’t understand what you feel or what you’re going through. Not even you. You’re an educator or you know, and you hear a lot of comments, but it’s got to happen to you before you can fully understand, and that’s the hard thing about an educator or a nurse, because they’re doing their job, they get paid to do what they’re doing and they come in and they’ll stick the needle in you, they’ll watch the machine and make sure you go home okay, but that’s all it is.

**Q: Sorry, can I just pause for a second? Now, do people have to go at one sharp or can we go about five or ten minutes?**

Mark: I’ve got all afternoon.

**Q: Because I know we’re sort of discussing some important stuff. I’ve got two more questions, ones that are particularly important. Do people mind if we go over by five or ten minutes?**

Joe: No. I’m not looking at the clock.

**Q: That’s good. Continue. Sorry Joe. Just in case people were - - -**

Joe: No, well that’s basically what I had to say, you know. It’s just awkward, you know. I’ll give you a perfect example. When my mother died I had tears for my mother’s death. My brother-in-law did as well, but he never carried the same understanding until his mother passed away, God bless them. Then he saw and he experienced exactly what – and most people who lose somebody close feel that way, so you have to experience the pain. We were talking before about a bloke, Jack, that does dialysis in the what’s a name, and he’s a – I don’t know what he is – he’s always got pain. If you’ve got pain, his is twice as bad. He thinks it’s like a competition, a contest, of who’s got the worst pain. It’s not. We all carry a different burden, you know. We all experience different things. I mean, I think I’ve lost my manhood because of it as well, you know, incapable of responding to other needs, if you understand what I’m saying.

**Q: Yeah, no I do. I do.**

Joe: It’s hard in every direction, whichever way you look, but you have to go there, you know.

Chee: On the dialysis patient, what we need is try to get independent. That’s why I move out. See, I want to let myself don’t rely on anybody. I can live by myself. I can handle myself, you see. You’ve got to learn how to be independent. Jack we mentioned, he like a baby because he always rely on everything, rely on people come to calm down him, you know, people come to touch him, talk to him, then he calm down, otherwise he’s always whinging. The character is very important. We must learn how to be ourselves and then we feel happy then. Don’t just follow the routine, you do this, do that. No need. Just do the right thing what I think is right to me. That’s it. Life still can be better than before. And don’t put the pain on other people. So nobody knows. If I see friends I never talk I am dialysis. But even you mention that, they don’t know. They wouldn’t know that is so serious. So that’s why I don’t want to see friends so often, because once you met them they always ask you, “How come, what wrong with you? How come we never see you?” I don’t mention. Even you mention, they don’t know what is that pain for all the time?”

Mark: Yeah, you do get sick of telling people that dialysis is over and over and over. You go back to the shortcomings et cetera, back to the question bit, and not that we weren’t on it, but you know, something that sort of just bounced off me. It’s interesting when I had diabetes I thought that was debilitating and certainly, you know, life changing. Once my kidneys went, diabetes is nothing.

Joe: There’s no pain with diabetes.

Mark: That’s right. It’s just all willpower and take your medication and check your sugars. I thought that was bad enough, but once, you know, this is just – I’m still trying to run a business. I still run a business. I’m frustrated I can’t do what I used to do in the business. I’ve got to pay people, but you know. And you’ve really got to plan yourself. You can’t be an instantaneous person or a spur of the moment person. Everything you do has to be planned ahead, worked toward. Even going out, you’ve got to plan that that’s a free time and work around it, you know. I need to do this work, I’ve got to get in the office, so I’ve got to work around that, you know. That’s not your priority any longer. Your priority is your dialysis and your world and your life and everything you do has to revolve around that.

Joe: So you run a Clayton’s business, only there three days a week.

Mark: I run it from home. I run it when I can.

Joe: I closed my factory because of that.

Mark: I was sitting in the office at three o’clock in the morning sometimes to catch up.

Joe: There’s so many things I could talk about, but anyway.

Chee: We lost the strength see.

Mark: So, you know, one of the difficulties is it’s a total change of lifestyle. Not just for you, but for anybody who is close to you, be it wife, children, friends, whatever.

Joe: I just wanted to go back to what Chee was saying about the family and pain and that. I’ve got seven grandsons and I’ve introduced them all to my lumps, because when I’ve got short sleeves on they see them, and I’ve taught them from young that there is a battery in there and if they touch this one, my arm goes like that. If they touch the other one they turn it off, and they sneak up on me and that’s what they do, you know, they’ll touch it so they’re not offended by it. But my mother-in-law, I’ve got to wear a long sleeved shirt all the time because she can’t stand to see that. It’s not offensive and there’s no sort of guard that you can probably wear to cover it, but you know, is there anything that can be done with them to get rid of them? I don’t know. Is there surgery, because they’re just getting bigger and bigger and bigger, and you know, we carry a bracelet, an identity bracelet for what we are. We’re a unique group.

Chee: Home dialysis, they don’t have the …

Mark: No, you just get a little one on that. So you have two hard lumps.

Joe: Is that all?

Mark: They’re hard as hard as hard, because you go in there every time. I’ve been using those for 18 months.

Chee: Home dialysis don’t have those thing.

Joe: Mine pulsate. Look, watch my finger.

Mark: So do mine but they don’t swell up because you’re punching lots of holes in there. That's the same hole I’ve been using for 18 months. It’s hard as a rock. Little hard lump.

Chee: After two years I probably get it too. Now I not have it.

Joe: Oh well I want yours.

Mark: Yeah, but you’re starting to get the track from all the holes see. You don’t have to worry about all the holes do you? You’ve just got the tube you’ve got to carry, which is bad enough. You’ve got to worry about infection and this and that.

Joe: I think this session is good to be able to express ourselves and hear other people who’ve got similar problems to what I’m carrying, you know, I think that’s the best thing that’s come out of today for me, that there are other people out there that carry the same thoughts and have the same problems, and there’s people like yourselves that are interested enough to want to do something about it.

Mark: Something like this is great, you know, where people can just talk and hear what other people are saying and say, “Yeah, I can relate to that, but you’re not on your own buddy”, you know.

**Q: Like a support group.**

Joe: Or an alcoholic group, you know, an AA. People go and sit in their groups and talk. There should be more of them for people so people do one, because this is where people find out about the real problem. The educator up the top, she’ll talk and tell you everything that’s on paper and everything else, but she can’t experience it, you know.

**Q: I’ve got two more questions. One is very important. Look, I’ll just ask both of them and hopefully, I guess, we’re not that stretched for time so we can go a bit over so that’s okay. The first question I have was, how easy is it for you to access or to get the diabetes and kidney health care that you need? So what I’m talking about is the ease of accessibility to contact who you need to to look after your diabetes and chronic kidney disease, in terms of cost, locality and waiting times.**

Mark: I think it’s partly covered. Actually getting to see a specialist in any situation can be difficult if you need them in a hurry, because they’re always booked out. But for me, my specialist is great. I can contact them any time I like. I’ve got nurse support for the dialysis. I’ve got technical support for my machine and it’s all free. There’s no cost involved. Even my private specialist, I’m bulk billed.

Enrique: Any time I have any problems, if I can’t do it with this hospital or HOSPITAL 12, they cost me nothing, and they act straight away. So no problems in there.

Joe: Over a long term, you know your GP very well. I’ve been going to the same bloke for 40 years. You come to the hospital, you’ve got full support. A telephone call and nine out of ten times you can ring triple oh and get assistance very urgently. There’s always somebody around that can make that call for you.

Chee: *1:34:42.3 the same * but I am new. I’m only here two years.

Mark: And he’s touched on something that we’ve missed, sorry, in health care. Accident emergency are great. If you go in there and tell them you’re a dialysis patient here <clicks fingers>.

Joe: You don’t wait. Tell them you’ve got chest pain and they’ll take you straight in.

Mark: Same sort of thing, but if you tell them they know you’re here for a reason, you know, something related to your problem. They look after you very well.

**Q: Sorry Chee, what were you saying?**

Chee: Some of the patient, I just don’t like. They always use the facility and it’s not that serious. The benefit is too good, that’s why some people are greedy.

Joe: He’s a lonely man mate.

**Q: What about in terms of travel and parking? Transport and parking.**

Joe: Well I’ve got great transport. My wife brings me and community transport. I was reading a notice today downstairs on the board, they’ve put up a plaque, that as from tomorrow the 29^th^ there’s a shuttle bus that will drive patients from the car park up to the hospital or take them to the various points. There’s nine points.

Mark: There’s been one on and off for a while.

Joe: Well it’s starting tomorrow apparently. They’ve set up a shelter in the car park across the road. So if you can drive yourself here now. It used to be a hell of a walk for me, especially to walk from that car park to come here and the street parking is limited because you get here at seven o’clock in the morning for dialysis and it’s all parked out, and it’s the nurses that are staying here.

Chee: They have shuttle bus.

Joe: You can’t park in any street. My wife come to visit me. I was here for three months and she’d park up around the corner and get a bus back. But any hospital is restricted. It’s not just here. They’d have a bit of a car park like we have in the shopping centres over there, you know.

Mark: You’re right. The amount of people that are here and have cars. It is an issue parking.

**Q: The roof is falling down.**

Joe: No, he heard us.

Mark: It’s the same at every – okay, okay I’m sorry. I think people who use hospitals accept the fact that it’s always going to be like that. There’s not much you can do without many, many millions of dollars, which the system doesn’t have and certainly has better things to do than build multi storey car parks with their funds. But it’s always difficult here. But HOSPITAL 12 was the same. Lucky they had some dedicated parking for dialysis patients that you could use.

Joe: Yes, that’s close at hand.

Mark: At no cost. Here it’s not. If you go in the car park here and you tell them you’re going to dialysis they let you in for nothing, but it’s still – I’ve driven around the car park and can’t find a spot in it anyway. So yes, it’s an issue. When I was in hospital my wife did the same as Joe’s. She’d park miles away and walk. As for transport, I drive, my wife drives. I live ten minutes away.

Enrique: I drive.

**Q: You drive Enrique?**

Enrique: Yeah.

Chee: I drive. Just what I think, the parking, now I just heard they will have the transport.

Mark: Everyone has that problem, but you know.

**Q: Let’s move along. This is the most important question I guess, and you can sort of see when I ask it, you can see we’re sort of working up to this. So moving back to health services again and thinking about your experiences with the disease, imagine you became the director of the hospital and you had the power to change things about the diabetes and chronic kidney disease health services. If we were going to design the perfect health service which would meet all your needs, what would it include? So I’ll just repeat the question. Imagine you became director of the hospital and you had the power to change things about the diabetes and kidney disease health services. If we were going to design a perfect system or health service which will meet all your needs, what would it include?**

Mark: In that question, you are assuming that funds aren’t an issue?

**Q: Well realistic funding.**

Joe: That’s what I exactly thought.

Mark: I would have said an individual nurse for each patient who looks after them every time, understands them, knows the ins and outs and the intricacies of a person, as Joe has raised a number of times, how everyone’s different, and some sort of education for carers and patients.

**Q: So better education.**

Mark: That’s realistic, the other one isn’t.

**Q: We were talking about the psychological issues, but would you - - -**

Mark: Well that’s part of the education.

**Q: Yeah, education, even screening for them. Did they screen for you guys?**

Mark: No. That’s part of the education. Those issues would be less if you were better educated. They would relieve some of those stresses and some of those issues with education.

**Q: And I think one of you guys mentioned perhaps having some sort of support group or social group for the patients to come and talk.**

Mark: Yeah. A forum like this is great.

Joe: Based on the forum, it’s only small, there’s only four patients in here talking, if I had the opportunity to set it up, the layout of the areas where we dialyse is tremendous. I feel that the nursing staff need to be questioned on a serious note, on a forum type base, where they say to you what each patient is doing to them, because what they’re feeling at the time, they come in cranky, they treat you bad. They come in happy they treat you good. They’ve got to come in in the right frame of mind to understand the patients are the ones that are in the suffering point. You know, they stick the needle in, we feel it, not them, and they’ve got to understand that as well. They’ve got to have a little bit more compassion.

**Q: So you’re saying the nurses should also be in a forum with the patients.**

Joe: A forum by themselves.

Mark: To help understand these sort of issues.

Joe: To understand. Not with us, not having patients in the room, but just nursing and a head person to question the nurses, what do they want from the patients to make their life better. That’s just one point. The other point is that, as Mark said - - -

**Q: Can you explain that a bit further? I’m not sure whether I’m understanding what you’re - - -**

Joe: Well, what I’m saying is, we all in here, and we’re speaking how high the service is of the place, what we’re getting out of it, but we’re also airing what we feel is wrong with it, and the people that we’re really talking about is not the system, but the individual person that’s treating us. That’s what I find. So many people have different ideas and methods of doing things, we don’t understand them. So I would have the nurses tell me the different problems that they are getting from the patients.

**Q: We have spoken to health professionals as well, the nurses.**

Joe: Well that’s one area that I would what’s a name. Mark said have one on one nurse. I think one nurse for two patients if you’ve got the staff to do it would be sufficient.

**Q: Or what about the same nurse?**

Mark: It was more the same nurse every time.

**Q: So there’s continuity of care. Is that what you’re talking about Mark?**

Mark: Yeah, that’s right. Yeah, so an understanding of the individual needs - - -

Joe: That’s right.

Mark: - - - of your two patients or whatever. Even three patients, but with the same nurse.

Joe: Yeah, same nurse.

Chee: Five patient, one nurse.

Mark: Whatever the ratio, but the same nurse.

**Q: So just like what you guys have for doctors at the moment, where you generally have the same doctor.**

Joe: Well, no, they change on a monthly basis.

**Q: Oh I’m talking about the specialists though.**

Mark: Yeah, yeah, we’ve got the same specialist.

Joe: Yes, that’s right. Your specialist knows what you’re doing. And the thing is with me, because I’m on a special chair, I’m in the same spot every day, but in every case wherever I’ve been at HOSPITAL 12 or dialysis here or in the what’s a name, they change your position, they change your nurse. So you don’t know where you are when you go into the room. You’ve got to walk around and have a look. That’s your area, even though it has two people using it morning and night, but that’s your chair. That’s your nurse and she might stay with you for two months, you know, but at least she gets to know and you get to know what to expect from her. Because I sometimes I walk in here and I know I’ve had a bit more extra to drink and I get scared because they’re going to scream at me when I come in. They talk to you like, you know, it’s wrong. There’s another thing. You’re on dialysis and you want to go to the toilet, you know, and they say, “I’ll bring you a bed pan.” No, I don’t want a bed pan. They’re reluctant to take you off. You can’t sometimes control it. “You hold it. You hold it”, they say. On two occasions I’ve soiled myself in this hospital because they don’t respond to your needs, you know. If you’re incontinent, you can’t control it and they need to understand what the patient is going through. That’s my biggest – everything else, I understand why I’m doing this. I understand the service is here for us and what we get is all for free, and that is great, but it’s the attitude of the individual. It’s like anywhere. You go into a shop and you stand up to a cash register girl, to a checkout chick, and one is good to you and one is really nasty. Nothing changes in any situation, be it employment in a factory or whatever, but that’s my comment, to make the understanding, to get a bond between patient and nurse.

Chee: To have one permanent nurse to look after five patients, that is good, but in the hospital here they like to give training for every nurse, so that’s why sometimes this nurse comes to you, sometimes the other nurse comes to you. You cannot have your satisfy nurses. That’s why every time I come to hospital I got a feeling today’s execution.

Mark: I know why it happens, but the question was if you were the director.

**Q: Yes, exactly.**

Mark: And it was a perfect world, and the funds weren’t limited.

**Q: Yeah. Any other things people would want in the system? So I think we’ve mentioned continuity of care, at least with the dialysis nurses, we mentioned better education. Part of it is also screening, at least giving patients - - -**

Mark: Psychologically.

**Q: Psychologically at least giving them an idea of what can happen, the toll that it can take and maybe a screening for that process we spoke about. What else have we spoken about? I think those were the main ones that we spoke about so far. Anything else people want to bring up?**

Enrique: I think the psychologic part is very important. Advise the reason for everything, because that's how we feel and how we do it with the family and how we do it with the friends and how we do it with ourselves.

**Q: I guess also maybe education of nurses as well maybe. Sorry Joe.**

Joe: Just picking on what Enrique was just saying, yes, psychologically. It’s very hard to be educated in certain things. I, in six weeks here, had a psychiatrist visiting me while I was dialysing and she would stay with me for half an hour talking to me about my issues and my problems and everything. It all sunk in. She’s been away now for six weeks. I haven’t seen her. But it’s not like you take a tablet when you’ve got pain in your hip, you take a panadeine and it takes the pain away. Those problems always stay with you and no matter how much people talk to you, it doesn’t change a lot. I don’t see what they’re doing to help me other than I just open my heart to them, tell them my life story and then, well what have they done for me, you know. I just can’t see the help that they give. But we need to be educated in certain things and prepared. Like to say to Enrique, well when you do peritoneal dialysis you’ll have nobody at home to help you, you’ll do it yourself, we’ll show you how to do it and then that’s it, you know. The poor man’s questioning he’s got no help, so he needs to be educated. You could have a nurse come home. You could have somebody helping once a week to control his diabetes. It’s just education for the individual to find out what’s available to them. I rest my case.

Mark: I do realise that all these specialists we see are under pressure because they’ve got so many patients, and going back to if you’re the boss of the hospital or whatever, certainly look at specialist numbers and trying to lighten their workload somewhat so they can spend a bit more individual time with people.

**Q: Did you guys feel that the specialist that you go to spend enough time with you guys?**

Mark: I think that they spend as much as they can. Well my bloke does. But I do realise he’s also under pressure, you know.

**Q: But did you guys get adequate education and explanation from them, or would you prefer more?**

Joe: I questioned, as you would have noticed me. I said I had the name Mouth of the South. But I do question them. It’s like taking your car to the mechanic, if you don’t tell him what’s wrong with it he won’t know what to fix. So when I walk into the room I sit with my doctor, he knows me and we’ve got a good bond, so I can talk to him freely about certain things and everything. I feel comfortable seeing my doctor.

Mark: I would agree. I can speak to my doctor and ask questions. When you’re in a hospital and they’re coming and doing their rounds, you can see they’re under pressure. They’ve got to see so many people over so much time. They’ve got this to do, you know. They are working very hard and in a way you’ve got to empathise with them too, but it can be a bit frustrating if you feel as though they’re sort of rushing past you, or you’re okay today, see you tomorrow, you know. But there are reasons, you know.

Joe: And private hospitals are worse than public hospitals for that. I had private health for 40-odd years and got rid of it just recently because I was paying $290 a month and every time I went in to have surgery - - -

Mark: You had to pay more.

Joe: - - - it’d cost me nearly $6,000 out of pocket just to have a hip replacement.

**Q: Anything else?**

Joe: No, that’s all. Just to say that cost is a big involvement in a lot of other areas as well and I had to get rid of it. It was costing me too much.

**Q: Is the service the same for the private versus public?**

Joe: Look, I’ve got the same doctor here, I’ve had eight operations on my right hip and two on the left hip and the same doctor that performed in the Mater Hospital at North R City in a private hospital is based in this hospital and performed my procedures as well.

**Q: So other people have mentioned other places that they would like perhaps some sort of diabetes service while they’re dialysing or maybe a dietician coming to them while they’re dialysing or podiatrist.**

Joe: Well that happens.

Mark: If you ask it happens.

Joe: It happens. They come.

**Q: They come. Now this is a regular thing or other people have mentioned that - - -**

Mark: No, I don’t think there’s anything regularly attached to the dialysis service where they come around, but if you speak to the Registrar, you know, I’ve had the dietician come and talk to me while I’m on dialysis. I haven’t asked about the other services because I see an external diabetes type person and an external podiatrist.

Joe: The services in while you’re dialysing do happen here in this hospital. I mean, if you’re a diabetic, podiatry comes round once a month and checks your feet and does all those things automatically.

**Q: Here?**

Joe: Yes.

**Q: Does it happen at the Satellite services?**

Joe: Yes, the same. They’re in the same building.

**Q: But do they automatically, or only if you ask?**

Joe: No, automatically the podiatry. If you’re a diabetic, podiatry come around and check your feet.

**Q: So that’s here.**

Mark: And is that if you’re in hospital or just - - -.

Joe: No, at dialysis. They come once a month.

**Q: In the dialysis that’s here in the hospital.**

Joe: In the 4 North. They also do it over there because they’re in the same building. They’re just two floors above.

Chee: Nobody come down. I never see any.

Joe: Well, are you listed as a diabetic with them?

Chee: I don’t know.

Joe: Well they won’t come to you.

Chee: I gave all the paper to Dr George.

**Q: Maybe you should tell them then.**

Joe: That’s what I say. It’s just communicating.

Chee: But I don’t feel anything wrong with me. See I am a patient 35 years ago declare diabetes centre, 35 years ago.

Joe: Have you got a diabetic card and everything?

Chee: But I never went. I never went, so I don’t care. That’s my attitude.

Joe: Well see, that’s why they don’t come to you because you’re not listed.

Chee: That’s right. But my paper record, I have paper hand over to Dr George, but he said to me as I told you, I’m doubt of your kidney problem, you come from dialysis, so probably he knows, understand my records. I was diabetes patient.

Joe: One other service that we’re getting here, a bit of help, is from the church. Like there’s pastors that come around when you’re a patient in hospital. They come round and they visit you. The Father that I’ve seen has befriended me and he comes twice a week every week to see me while I’m dialysing and he’ll sit with me, have a cup of coffee, give me that holy communion and he makes me feel good. We’re friends, you know.

**Q: That’s nice.**

Joe: He knows I’ve got a lot of problems, so that’s what has happened. But the thing is, you know, if you ask or require a dietician to come and see you, psychiatry to come and see you, whatever, they don’t come automatically. They’ve got to be booked. They’ve got rooms here as well that they’re seeing a lot of patients.

**Q: So in the ideal service would you make them automatic?**

Joe: I think for people that require it.

**Q: With diabetes.**

Joe: Yes, it should be automatic, once a week or once every two weeks they come and visit you because when I was seeing a doctor trying to lose weight, I did it by visiting my doctor five minutes every day and I was just about to show you – not every day, once a week – and I visited him and we just sat and talked. He said, “What did you have for dinner last night?” and different things, and I lost seven and a half stone in eight months.

**Q: Wow.**

Joe: And I lost that contact and so things just go back. You lose it you know. And that’s what I looked like. So it is help you need. You need to be able to attend to somebody. It’s like an alcoholic.

Chee: You lose seven and a half kilo.

**Q: Stone, not kilo.**

Mark: Stone. That’s about 15, 20 kilos.

Chee: Twenty kilo.

**Q: So other people also mentioned a combined diabetes chronic kidney disease health service. What do people think about that?**

Joe: I don’t think that’s necessary. You know, because you can be a diabetic patient and you go and you just have your medication. You check once a month or whatever.

**Q: So they’ve got the same doctor and the same service looking after both. All the services are combined so you don’t need to see separate doctors or separate things.**

Joe: But that’s what happens here now.

Mark: That’s a bit hard on the doctors because most of them specialise in an area and it would be very hard to be an endocrinologist and a renal specialist.

**Q: Or even having a place where you see both together.**

Mark: Oh okay. I understand what you’re saying. If it could be co-ordinated, it’d just mean one less visit.

**Q: I guess you’ve got, Martin’s looking after both for you.**

Joe: Dr G’s looking after everything. I don’t have to go and visit. I visit him and he’s controlling both, but they automatically do it. See, these gentlemen do it at home. They’d have the same available capacity to do it.

**Q: So in effect, you’re having both together.**

Joe: Yeah, that’s right.

**Q: What do other people think, Mark or - - -**

Mark: No, there is an advantage because if they’re working together they know exactly what’s going on, they’re talking together about things. Joe said you don’t have the same availability. Well I do, but I do it myself. I do my own blood tests and you know, I self adjust my own medication and my endocrinologist only wants to see me once a year, just because I’m able to do it. But that’s not for everybody. I’m talking about me. Not everyone can do that.

Joe: That’s right.

**Q: Did you want to say anything?**

Enrique: No, I think the services together is better. Even you think everybody have access to the record and every doctor with the records from everyone.

**Q: Thanks Enrique. And Chee, any comments?**

Chee: I have the same comment of them. I think diabetes, when we have diabetes, the first beginning, because your diabetes, because your kidney, that’s the second section. So once your diabetes come to dialysis, your diabetes is not much concern I think.

Joe: That’s right.

**Q: Just looking at the other complications of diabetes, the eyes, the feet, stuff like that and - - -**

Chee: Yeah, but I’m not serious, so that’s why I never ask.

Joe: Oh it can be.

Chee: I don’t have anything broke or anything serious, so I never ask for diabetes care. But actually, if somebody is telling me to see the diabetes - - -

**Q: It can be serious, yeah.**

Joe: Well it is, because my diabetes has created my kidney failure. I’ve now got neuropathy in both legs, so constantly pins and needles. I lay down I lose the circulation. I’m legally blind in both eyes. I can’t see your face from here. I can see you’re sitting there.

Chee: Oh you lose a handsome face.

Joe: But eventually it can go to your fingers. I just had teeth out and I was a very big concern to the doctor about my teeth because of my diabetes and different things. So there’s a lot of issues that it leads to eventually, and I asked my renal doctor, “What will kill me first, my diabetes or my dialysis?” and he said, “Your diabetes.”

**Q: Well we do need to close up I think because the rooms at 1:30. We’re going to hand out a survey, but while we’re handing out the survey, has anyone got any last comments they want to make? Feel free to make it. I’ve still got the recording on, but we do need to hand out the surveys because we do need to vacate by 1:30. Has anyone got any last comments they want to say?**

Joe: Well I just want to thank youse for having this focus group. It’s made me feel a lot better - - -

**Q: That’s good, yeah.**

Joe: - - - leaving here, understanding what other patients’ problems are, and I think if a bigger group was together it would take a lot more time and it couldn’t be done like this. Small groups make it much easier to control and to get through to you what you’ve got to do, you know.

END OF TRANSCRIPT

**Focus Group 11**

**Q: I’m not sure that everyone’s met everyone, but maybe if we can quickly go around the circle and say who you are. So my name’s C, I’ll be facilitating the focus group.**

Joseph: My name is Joseph, I’m here for the focus group and I’m a dialysis patient with diabetes as well, so I’m looking forward to this.

John: My name is John, I’m a diabetic patient, not yet on dialysis, but in readiness for it.

Sharon: My name is Sharon, I am a dialysis patient and I haven’t really been diagnosed with diabetes, but I’ve been told I have to transplant, that the steroids will probably push me into type two diabetes.

Ioane: My name is Ioane, I’m on dialysis and used to be a diabetic, but two years now no longer on any medication.

Denise: My name is Denise, I’ve been a diabetic for many years and I’ve only been on dialysis since December last year.

Janine: I’m Janine, I’ve been diabetic for 45 years, I was PD for three years and been hemo patient for two years and it’s been quite hectic.

**Q: So how many people on hemo dialysis and how many people on peritoneal dialysis? So who’s on hemo dialysis?**

Speaker: Me.

Speaker: Hemo.

Denise: I’m on Home D.

**Q: Have we got any peritoneal?**

Janine: Yes I was, I used to be yes.

Ioane: Before.

**Q: And your wait…?**

John: Yes I’ve been prepared for it, but not yet.

**Q: Maybe I’ll just move on to the first question then, I’d like you to think about the experiences that you’ve had with the diabetes and chronic kidney disease services here at the Hospital 3. So what would you say are some of the strengths of the diabetes and chronic kidney disease health services here at Hospital 3?**

Joseph: Well with the diabetes I was introduced to Dr. Fank about a year after I had, I was diagnosed with diabetes through my GP and Dr. Fank has been excellent as far as I’m concerned and he had brought me to a good level. And then I had a bit of a problem with the finance, my finance portfolio and I invested wrongly and I went down and lost everything. And then I got rid of my insulin and everything and moved away from any medications for about 18 months and that then brought on the kidney problems. The kidney problems when I was diagnosed with a kidney problem I was non plus, because I had never ever been in a need for drastic medical help, but I was then introduced to the dialysis coordinator who changed the whole aspect of my feelings for dialysis and everything. And then she’s the one who brought out everything to me and that made me much more different and from then on I’ve had kidney disease for the last four years. It took me that time to get on a level playing field with the whole set up and now it has come to a point that I can handle it so well thanks to their help and medication and everything. So I am very very happy with the outcome.

Sharon: I’m still at a very bad place in my head, because the cause of my kidney disease, as I said I’m not diagnosed as a diabetic was amyloidosis. And in a way that saved my life, because it was a symptom they picked up before I then had to have the bone marrow transplant. But of course I was then left with kidney, very poorly functioning kidneys after the amyloidosis was in remission. And I’ve been handled extremely well through the renal physicians of this hospital over a period of 10 years and I was prepared for dialysis by having a fistula done in September of 2011. But after discussions with my doctors, didn’t actually start the dialysis, because I didn’t want to, so I was on conservative treatment with drugs, but I’m also on the transplant list and I’m fortunate enough to have a live donor. So things have not been as good for my head space after that, because I’ve been going through the list of pre transplant list as has my donor and I didn’t start dialysis until I had to do the last two tests, which required a contrast dye and that was going to send my kidneys into complete collapse. And my head space is bad, because one of those tests, the CT scan picked up other issues that I needed to have. I needed to have two surgical interventions, so I’m still on the transplant list, but I’m also seeing the psychiatrist at the moment, because I’m finding dialysis incredibly challenging. I started in March and the challenge about it is the lack of control. I feel I have no control over my life and the nurses are amazing. I’m attending Ward 6A which is the acute dialysis, I have briefly been to the satellite centre. When you get to the negative things, I’ve got a few comments around that, but the positives have been the nurses really try to help you through anything. I’ve had a few complications where after surgery for example I ended up with fluid. Instead of being thrown out into the cyclical thing with all the other doctors, most of the things that I’ve had to follow through on have been able to be handled by the fact that I’m going to dialysis. So when people need extra bloods they can take them out of the machine. If I need to see the dietician I don’t have to wait for appointments, because the dietician visits the ward. And you can request to see the RMOs that are changing, so you can actually request to see a doctor. Like something actually went wrong with the healing of one of my wounds and that was managed while, because I’m in the acute dialysis ward. So those are the positive things.

**Q: So just the regular medical contact, the support that they're giving, including the support with your head space, adjusting to dialysis?**

Sharon: Well the nurses are unable to do that, but what's happened, I’ve been referred back through the transplant clinic and I’m now seeing the psychiatrist through the transplant clinic once a fortnight. So it’s more the; one of the things with all of this is logistics and you're trying to balance so many different things, but to me it’s been a real boon to be handled, I’m being handled in the acute ward. I think if I was anywhere else it would be, I think I’d go under and I know it sounds ridiculous, but in terms of parking, this sounds really stupid, but I’m sure people will identify, parking here is almost impossible. Until I started dialysis it was suggested I apply for a disabled parking sticker. And the fact that through my GP I was entitled to that, means that coming to dialysis has got about 50% of the stress removed. If I had to come here three times a week without that parking sticker I’d just be off the planet.

**Q: Now we’ll get to the negatives later, we’re concentrating on the positives or the strengths at the moment.**

Sharon: Well that is a positive, having the sticker.

**Q: Definitely, now anything else people want to mention or any experiences in terms of the strengths of the services here at Hospital 3?**

Janine: I believe that they do prepare you very well before you go onto dialysis. You have all the seminars and pre transplants and all this. I mean they really, really inform you, it’s very informative and they prepare you in a very good way. In your head you sort of know what to expect and it’s also very good to speak to people who are on dialysis already to find out how they feel and what's expected. Now a lot of people have come and spoken to me and I feel like I’ve helped quite a few people relax about it and feel better about it. That’s the main positives. I’ve got a couple of negatives. I’ve also got, my sister wants to give me a kidney. Being on peritoneal dialysis and being diabetic that’s all glucose in their fluid. I put on 17 kilos ok. I’ve lost 15 since I’ve been on hemo, but now I’ve got to lose that extra five which I can’t lose and I can’t get that kidney until I’ve lost it. I’ve done all the work ups, I’ve passed all the work ups, the transplant work ups and it’s just so depressing. But I have got articles, I phoned about them doing transplants on bigger people and that the success rate isn’t that much different than a normal person who is not overweight. And it’s just getting for me now getting quite desperate; because I’ve been diabetic for so many years my arteries and my vessels are calcifying. So I can’t have access, I’ve had all these attempts which haven’t worked. This is about the fifth or sixth family care and this one is giving trouble at the moment. And because this one went septic, I’ve got septicemia, it’s blocked, they couldn’t put another one in, so it’s blocked everything. So to put a fistula in this one I have to go for another angio to see whether they can or not, because if this is blocked, how is the fistula going to work? So I’ve got all these problems at the moment, but my Dr. W is rooting for me to go onto the emergency transplant list, because Dr. Q explained that even though I’m a bit overweight, he doesn’t want to take my sister’s kidney, because he doesn’t want her to lose a kidney and me to lose a kidney, because with the live donor you can’t take as much tissue as you can from a deceased donor. So he was quite happy to put me on the deceased donor list, but now it’s become urgent because of my access, access has become a big problem. So that’s my story.

**Q: Are you ok, it sounds like it’s taken a toll?**

Janine: Look it’s taken its toll on me, because it’s all very recent, it’s all you know, on Friday I was really, I’m normally a very positive person, but on Friday it really got to me. I thought I was <inaudible>. Not going to make it.

Sharon: That’s what I’m like all the time.

Janine: I’m normally, I’m not like this.

Sharon: Me too.

**Q: Would you like some tissues or?**

Sharon: What Janine is describing as far as head space that’s where I’m at; I’m not normally like this, but the reason I was referred to the psychiatrist is because I just cry all the time now at the drop of a hat. If anything at all goes wrong in dialysis, which happens all the time, you just end up crying.

Janine: That was the first time I really broken down ever. I’ve never had to see anybody about it, because I’m quite positive, you take every day as it comes, because that’s the way it is. I laugh and this is the way you carry on. If you want to live you’ve got every reason to live. I’ve got kids and I’ve got a husband and I’ve got you know my whole life ahead of me, I’ve just turned 50. But I mean I just feel for everybody I think. Dialysis patients are the strongest people I know, because nobody realises what we actually go through. You know fixing up your fistula because it’s not working every five minutes, in and out of radiology, in and out you know, it takes a toll on people sometimes and I really can relate to… I’ve got a really good friend who does home dialysis and she’s also been through them all, and we got the same level, we can understand each other. I don’t think many of the nurses and the doctors actually understand what we actually go through.

Joseph: I think even when Janine and Sharon were saying that, it reminds me of the time when I was diagnosed and I went into the kidney part of it, I was in a set up where I felt that’s the end of it and I told my children and my wife too that I won’t be coming back. But then the way it was handled here it made me feel positive and it made me feel different and that had brought it out a long way and I feel it so much more better now than what it was then. And even…

Janine: The staff here are like guardian angels. If it wasn’t for most of the people, especially Debbie, Debbie is my life line.

Sharon: Debbie is the fistula…

Janine: Debbie yes she’s the fistula access nurse, she’s fantastic, she is absolutely my knight in shining armour.

Joseph: Because I called the dialysis nurses the life givers. That’s…

Denise: I agree the support is great here. I remember before I had to go in, I went to a seminar and you could take anyone with you that you wanted to and because I’m not married and not having children, I took two cousins. And it was a real eye opener for both of them of what this was a full day and they were so pleased that they actually went with me, because they found out what actually happens and what goes on. And then when I started dialysis those two especially one of them, she came in, she was here for a couple of weeks with me and the other one came in for a day, because she works. But they found out the support was good and I do home dialysis now; I started that in February. I started dialysis in November last year, I felt so sick and I said to the renal physician I’m not ready to die yet. She said you're not going to die on my patch don’t worry and it’s amazing the support when I started, how much better you do feel and you lose all that fluid. I got that I couldn’t walk very much because of the *0:17:20.

Janine: The PD was bad especially for diabetics, it’s not ideal to put a diabetic onto peritoneal dialysis. I just feel towards the end I got peritonitis three times and it wasn’t from me not being clean enough or washing my hands enough, it was all from internal. And that’s one of the worst things anybody can go through is having peritonitis. And all that weight I put on, I mean I really thought I wasn’t going to make it, because initially before they put me onto peritoneal dialysis, they tried to get access and they couldn’t, so they put me onto peritoneal dialysis. And they didn’t hear from me for three years because my machine never beeped, I never had a problem, whatever. And in those three years I just packed on weight and they did my line change every six, every three months or whenever they did it and it just got to a stage where I couldn’t do it anymore. And I had to go onto hemo and hemo really got rid of most of the fluid and I started losing weight and it’s just excess now. That’s the problem.

Sharon: Did you want to move on to the negatives or other people got positives? That’s what I mean, I’ve got a whole lot of negative stuff as well.

**Q: Any other strengths people want to mention? We will definitely get some negatives in another 10 minutes or so.**

Denise: The only other thing I was going to say is when I started in SDC on the R City dialysis centre, they had the nurses down there are great, they're on call 24/7 and you can ring them any time and I suppose I do home dialysis so I find that that’s better, because I’m not tied to a particular day or particular time. I found now that I’m starting to do it at an afternoon and you go into the night, because I was sitting there during the day and looking at all the beautiful sun out there and thinking I can’t get out there, I can’t get away from it. So I switched to the afternoon and it made it a lot better.

**Q: Ioane anything you want to add in terms of the strengths?**

Ioane: They all very good to me.

**Q: What about in terms of strengths of the chronic kidney disease services here? So I think we’ve mentioned that they're supportive, I think that’s what everyone seems to appreciate is the support they're getting.**

Janine: It is, it’s good, the support is fantastic, really it is. Everybody cares and that makes a big difference.

Sharon: There’s a difference in the support which we’ll get to later on with reference from my perspective of the transplant situation versus the diabetes and dialysis situation. And diabetes and dialysis situation, the information provided is amazingly good, the coordination is very important is also very good. For example between the satellite centre and ward 6A the acute ward, the NUM nursing unit managers are constantly in touch with each other. And if they’ve got a spare chair they arrange between the two of them if someone is able to check, they’ll phone people up. We’ve got people who are on set shifts and occasionally they want to do something special, like go to a wedding or something like that, so through the NUM you can nearly always organise to come, they will try and accommodate you. So that sort of thing is very very positive through the dialysis side of things. But when we get to the transplant I’ve got a few issues with that.

**Q: What about from the diabetes side? We talked a lot about the kidney side, any comments about the diabetes side in terms of strengths?**

John: I’ve been dreading the dialysis situation and I’ve been avoiding all issues on it and scared stiff about going on a dialysis. And ever since I’ve had the fistula put in I’ve been worried stiff about the day when I will be going on dialysis. So I’ve been taking care of my diet, especially my wife is particularly keen on ensuring that I eat the right foods and all sorts of things to ensure, but I had a kidney and I found that they saw a tumour in my kidney, so they removed one kidney. So I only have one kidney and then shortly afterwards they found that I had another cancer in my lung, so they lopped off one third of my right lung as well. So I’m a two cancer patient survivor. And on top of that I’ve got my cardiac situation is not all that good, so I’ve been seeing my cardiologist and he has been sending me back and forth for angiograms. So I’m in that kind of situation at the moment and every time I see Dr. *[Stagenburg]0:23:00 she tells me oh you're GFR is still 15 and…

**Q: So Dr. [Stagenburg] is your diabetes?**

John: Nephrologist, renal specialist at the moment and she looks after me very well actually, she is very good. So was Dr. *[Shen]0:23:20 before her. And I’ve got many other doctors outside this hospital looking after me as well. So I’ve been ever so grateful that the dialysis date is being postponed.

Janine: Enjoy it, go away on holiday, enjoy it.

John: That’s what I was told, I was told that I could go away on holidays and I’ve just come back from overseas and hoping that I will be able to make another holiday some time soon, but you never know.

Sharon: Actually that’s one positive, as part of the kidney work, sorry it’s back to kidneys rather than diabetes, as part of the kidney work up having a CT scan, that actually picked up possible renal carcinoma with me as well. So which wouldn’t have been picked up, which led to an nephrectomy, the right kidney removed. And it was stage one, fortunately encapsulated, so I also had a very large splenic aneurism picked up and so without the screening that’s been done for the kidney disease, the cancer probably would have got me later. So that’s a very big positive.

**Q: Really quickly any strengths or positive things about the diabetes services? We talked a lot about the chronic kidney disease services and I know that’s probably the main thing at the moment for most of you because of…**

Janine: Well it’s the diabetes that actually caused the…

**Q: But I know now because the predominant thing is the chronic kidney disease for a lot of you, any strengths about the diabetes services?**

Joseph: Yes I could elaborate on that a bit. With my diabetes when I was diagnosed and I was introduced to Dr. Fank we started off quite well actually because he explained to me, then I was introduced to insulin, because I was taking too many tablets for diabetes and Dr. Fank handled me right along and he introduced me to the regime that I should follow which I did. But I was still not sure of things, then he moved me into a diabetes assessment as well and we did the whole assessment on myself at the clinic in the North Shore Hospital. And then I found out if was to follow those things and put my bit of contribution into it, I can enjoy a far better lifestyle rather than depending on the medical side of it. And I had a good chat with Dr. Fank on that and he then explained to me yes do this and let’s take it one step at a time. And it took me at least about a good six, seven years to get this into some sort of program with myself. And I think I contributed a hell of a lot to it and today my readings are between five and seven at all times. And only two insulin units of 23 units in the morning and 23 in the night. And during the day it suffices, because it’s a 60/40 thing and Dr. Fank has been wonderful with the thing. But the only part that I like to mention is and I think we’re going to that shortly is that at dialysis we don’t have sufficient diabetic input or investigation through the nursing staff. That’s about the only thing I have.

**Q: We’ll get into that later.**

Sharon: A negative on diabetes?

**Q: We will get into that, so any other positives or strengths about the diabetes services that people want to mention?**

Denise: So I would say there used to be the diabetic cottage, I don’t know when it’s out of the new hospital, I’m not quite sure, but they were marvelous, because I’ve been a diabetic for 30 years and they were really good, 20 years sorry the diabetes and they’ve been very supportive from down in the cottage.

**Q: Were they the nurse educators?**

Denise: Yeah the nurse and the dieticians from there too.

Janine: From the diabetic side yes, also the NDSS is very good as well. You get your blood test machine and all that kind of thing for a reasonable price and you get your needles for nothing. I’ve had diabetes for 45 years, if I got it these days I would have been smiling. 45 years ago my mum was boiling my syringes and my needles, I had blunt needles. I didn’t have any disposables or anything like that, no blood testing machines, no nothing.

**Q: So you have type one?**

Janine: Type one yeah, so I’m actually surprised after 45 years I’m still standing here. There’s lots of people who have lost limbs and gone blind and considering the length, it’s quite amazing for me.

**Q: What impact has attending the diabetes and renal health services made to your health?**

Joseph: Excellent. Excellent, I’ll repeat it.

**Q: So it’s made a good impact to your health?**

Joseph: Very good impact.

John: And it has controlled my diabetes and like Joseph, my readings have been very good, consistently good, just taking my medications.

Sharon: My diabetic experience has been completely negative, because I’ve obviously if I had got diabetes had it under control by the diet that I eat myself. And by having one reading on a glucose tolerance test, I’m now labeled diabetic on the hospital records, which is presumably why I was invited here today. And that’s had a very negative effect for me, because I’ve been put in hospital for other things and immediately labeled as diabetic with a diabetic dietary thing, which has created huge problems, because renal patients don’t have any appetite anyway, and then they give you this totally inedible food. So I just went off my head last time, slammed the lid down on the top of the stuff, said I’m not eating this junk and unless you let me go down to the cafeteria and buy something I can eat, I’m checking myself out. So that at the admin level, but the other negative that’s occurred is that this information has now gone through to my GP who I’ve been seeing for 30 years and who as part of her practice handles diabetic control, chronic diabetics and renal. She said Sharon I don’t know what I’m supposed to do, you're not diabetic, what am I supposed to do, give you pills for something that doesn’t even show up on your blood tests. So there is this real mish mash and it’ll come up again when we get to the kidney stuff of admin tying into clinical services. I don't know about elsewhere, but in this hospital it’s a bloody disaster.

**Q: I’ll have to explore that a little bit later. Now what impact has attending the diabetes and chronic kidney disease health services melding together made to your health, anyone else want to add anything?**

Janine: Been fantastic, I wouldn’t be here if it wasn’t for them.

Denise: Yes I have no problem with it, but then again I’ve seen my, both of the doctors privately here. And the only problem is that when I saw the dietician down in the SDC, the diet that they give you for the renal…

**Q: Sorry SDC is?**

Denise: R City dialysis centre.

**Q: Where is that, is that here at Hospital 3?**

Rosie: Yes it’s community health centre.

Sharon: It’s the satellite centre.

Denise: The problem is that sometimes those two diets they clash with one another.

Janine: They do definitely.

Denise: Yeah.

**Q: And no one tells you what a good in between is?**

Denise: Well no, I said to the dietician, look you know what you're doing is sort of clashing with the diabetic diet and she said oh no it’s not and I thought well it is really.

**Q: So that’s probably a negative. Ioane have you got anything you want to mention about what impact has it made on your health attending the diabetes and chronic kidney disease services, good impact, bad impact?**

Ioane: I think it’s quite good with dialysis, two and a half years now. Since I had hemo dialysis, suddenly my blood test come up by dialysis specialist, John, you're not allowed to inject yourself anymore. Why? No more diabetic. No more. I mean might be the dialysis cleaned all...

Janine: I think so maybe.

Ioane: I have been diabetic for 35 years. Amazing.

Sharon: Just one last thing, I didn’t mention the overall impact, the overall impact on me at this stage is extremely negative, because I was on a; it was mainly my creatinine that they were using as a measure and it was staying, after my bone marrow transplant it was 180 and then over the years it just crept up. And I was using conservative treatment, Resonium, *0:33:36, *, etc. Having to start dialysis because of the other test I was doing, my overall health is definitely down, because I was pretty good before, I was tired but very good. Now after dialysis I’m incredibly drained, I’ve got no energy, it’s been impacted by the fact that I had the nephrectomy and I’m also therefore anaemic, but I can’t be given blood products, because I’ve got to match with a live donor, so I’m just trying to get past on *0:34:13. So the way I feel compared to the way I felt previously is terrible.

**Q: I don’t know whether that’s just as a result of the progression of the disease and everything that’s happened, rather than actually the health services, the health care that you're getting, I’m not sure whether…**

Sharon: It’s the impact of the hemo dialysis, because I’ve just had my two days off in a row and I’m feeling pretty good today, but when I only have one day and the dialysis I just feel terrible the whole time.

**Q: I definitely understand what you're saying, I know definitely after hemo a lot of people feel washed out, I think that’s general, but I guess I’m just wondering whether that’s just you did require hemo dialysis just because of the way unfortunately the disease has progressed or …?**

Sharon: No not really because I was down to 9% kidney function with both kidneys when I was on all the other medication. I mean now it’s less obviously because I’ve had a kidney removed, but it can’t get much less than 9% in terms of and being controlled by medication.

**Q: Let’s switch topic, now that we’ve started discussing some of the negatives, I think Sharon’s mentioned some, you guys mentioned some, the clash between the diabetes diet and the renal diet and no one has put them together for you. So let’s talk about some of the negatives, so based on your experiences what would you say are some of the weaknesses of the diabetes and chronic kidney disease health services?**

Joseph: As I briefly mentioned the positives, with the negative side of it, I think in the dialysis centre where I do dialysis, down in the community health centre, the nurses are all ofay with the set up of dialysis and chronic kidney disease, but when it comes to diabetes they only look at the testing your blood glucose level and say 11 o’clock in the morning if you're in the morning shift. Now I would actually look at getting a little more information where we can, with them to be able to talk about diabetes, diet and all that with the nursing staff, because they are the ones, the front line staff with us you see. And also visit of the diabetes registrar or the diabetes doctor to the dialysis centre occasionally to ask us how we are. Like the renal people do you see and then combine it together, because…

**Q: So that’s what you would like is it?**

Joseph: I would love to see that, because I am really, I control it quite well myself, but then medical help is always…

**Q: Where do you dialyse again Joseph?**

Joseph: Down in the community health centre in the North Shore Hospital, satellite section.

**Q: And the diabetes contact that you get at the moment is not that much, you just see them once every three months or four months?**

Joseph: No only when I see my specialist Dr. Fank is at the time when I’d be able to do all of it and my local doctor, but once again a specialist is always best anyway. And Dr. Fank has been the person who has been in charge of me for so long, so…

**Q: Any other points people want to bring up?**

Janine: Joseph’s got a point about the diabetic side of dialysis at the dialysis unit. I see Sam Hawkins every three or four months for my diabetes and my HbA1c once he’s given and all the rest is all hunky dory and all the rest, but when your toes start going numb and things like that, you need to see somebody about it and you need to try and keep on top of it. Because my toes are numb half way up my feet, so I’m worried am I going to loose my feet, am I going to you know? And I think dialysis has also got a lot to do with making your diabetes actually quite worse. That’s how I feel.

**Q: And did you talk to the dialysis nurses about it?**

Janine: No I don't speak to the dialysis nurses about my toes going numb no I don’t. I don’t, but I mean yeah are too busy, they're putting people on and off and running around, it’s a bit difficult for them. But maybe it is a good idea that a diabetics doctor comes around and checks up on the kidney patients. I think that’s a good idea.

**Q: Any other negatives people want to mention or weaknesses?**

Denise: It probably would be very helpful for the doctors or the registrar to come and visit, because the renal people they do a round once a week. It might be better if the diabetic ones or the endocrine ones came once a week, if there was more consultation between them yeah.

Ioane: Every time at the dialysis before tea time they had the nurses come and check your blood and then that’s about it, they put down your reading, what's?

Janine: What does it help anything, you're right, doesn’t help anything?

Ioane: Where’s that going to go? That’s all they do and they feed you with wrong food and…

Janine: They do.

Ioane: And sandwiches.

Janine: The sandwiches have got cheese, tomato, all the things you're not allowed to have.

Ioane: Potassium, there’s cheese and tomato there. And I complain over there about the sandwiches not very nice sandwiches, they say take your own if you don’t want.

Joseph: That’s an interesting point too, because what as patients we seem to be, we seem to get the information that chronic kidney disease starts off with diabetes and that more or less activates chronic kidney disease and that’s why I’ve done my own research on that and found that even though if there is not enough assistance when you're on a dialysis and with chronic kidney disease, then you can always find a bit more information from outside and then take it with you to your specialist and then get the information you need there. But if the nurses or the staff in there and if a registrar comes around and then discusses a bit with you, say for example about the numbness of toes and things, the nurses don’t get into it, because it’s not chronic kidney disease.

Janine: Part of their job no.

Joseph: You see it’s diabetes, so there is that little bit of a difference there.

**Q: So they just worry about the chronic kidney disease and that’s it, is that you're saying?**

Joseph: Exactly you see, because they will hold a fistula and then say it’s working beautifully, but they wouldn’t hold your toes, is it numb, because they don’t want to go into that. So you can’t blame them as such, that’s why as a patient I sort of take that into my control and make sure that I’ll take it with me to my specialist.

Sharon: Just following through on that, because I’m in 6A obviously there’s a different mix, the acute ward, there’s a different mix of patients, there’s a lot of people who actually aren’t diabetic, but part of the model I think, the service delivery model is they are absolutely on the edge of being understaffed. If any individual appointed nurse has time off, they’ve got to try and get people in from agencies. And they seem to be very fortunate around this area in that there seems one agency in particular that does specialise in dialysis nurses. But another thing that I’ve noticed, which ties into the model as well is that over time you get to know all of the nurses personally. And when they are looking after you they are looking after you, but they are so on the edge with terms of what's required of them during a shift that if they're not looking after you on that day, you can say oh hello so and so and they’ll nod and say hi, but they won’t necessarily talk to you, because on the specific day that they're working in their shift, they are looking after particular people. And they can’t afford to in terms of time to actually talk to other people just even to have a chit chat. So I do think that’s part of the service delivery model and I do think it’s part of them being on the edge of being under staffed. And so that’s probably impacting on these things of not being able to discuss things that might be to do with something else that’s wrong with you. So I’m as you can tell from today a fairly assertive person, so like Joseph I keep very big tabs on where things are up to, so that if I need something done, the nurse that’s looking after me on that day, I say hey remember today I have to have such and such. Because what the nurses are looking after is what they have to do for your kidney disease on that day and it might be for example you need your iron supplement or your Aranesp. They say to me are you due anything today and I always know, but then they check it on the paper as well. As I said that’s part of the service delivery model; I don’t know how you break from that, it’s a very hospital focused model.

**Q: Could I ask you what do you think would happen to you if you weren’t as assertive?**

Sharon: I think some things would get missed and if I was coming from a diabetic background, I wouldn’t know what to ask for in terms of additional services, whereas the things I’ve asked for have been part of the medical model, where I’ve been an inpatient and I’ve actually had dialysis a few times as an inpatient. So I’m following through on a dressing, a particular dressing or medication or something like that, which is to do with the chronic kidney disease, not to do with diabetes.

**Q: Does everyone agree with Sharon? What would people think would have happened; obviously most of you guys are pretty empowered about your health and you know what you're meant to be getting and what you're not meant to be getting and perhaps most of you guys will probably, if you know you need something, you will say it. What would you guys think would happen to you if A you didn’t know what you needed and B you didn’t ask what you needed either?**

Janine: Six feet under.

**Q: Do people agree with what Sharon’s been saying?**

Sharon: I actually think in 6A, which is as I said chronic, some of the people who are in there are quite elderly and some of them are coming from nursing homes. They always get what they need, because the nurses know them, they’ve been going for years and whatever. So I don't necessarily think people will miss out, but if you need something a little bit extra you’ve got to be able to remind people.

**Q: What does everyone else think?**

Janine: You’ve got to have all your faculties, if you haven’t then mistakes are made and I’ve seen it and you’ve got to know what's happening around you and be quite aware.

John: For a long time my whole body was itching from top to toe and I didn’t know what was wrong with me and it was Dr. [Stagenburg] who determined I should have *[Nupentine]0:45:58 as a medication and that has stopped a bit. But I still scratch all over my body, do the rest of you have the same?

Janine: I’m starting to scratch already. [laughter]

John: Is that a diabetic problem or a kidney problem?

Janine: It’s a diabetic and kidney problem.

Sharon: It’s a kidney problem.

**Q: Kidney because of the uremia, if it’s not cleaning the waste, it might mean that you require; look I don’t want to scare you, but it might mean that your time might be approaching the need for dialysis, I think once you dialyse that does help.**

Sharon: Don’t let that scare you too much.

Janine: When you dialyse it actually dries you out, it actually dries your skin out, it also becomes quite itchy, you’ve got to keep on putting creams and whatever. And also I don't know if it’s phosphate or potassium if it’s too high makes you itch.

John: Potassium oh gosh.

**Q: We can talk about this later with me John, any other weaknesses people want to mention?**

Sharon: This may not be a weakness; everyone mentioned with reference to Debbie the fistula specialist nurse practitioner, she has a tiny little screening echoscope thing which she takes around with her. And it’s one of the, *0:47:22 I think, but it seems to be really bottom of the range and I believe that she needs access to the next step up, not the full diagnostic, but because I’ve had a couple of times where there’s been issues with cannulation and at one stage on the basis of her just having this tiny screening machine, I had to be sent across to Dr. Q who’s got a $40,000 machine only to find out that there wasn’t anything wrong. Now it wasn’t just her machine, there was a whole lot of other things, but to me that particular machine needs to be upgraded; I think that could really help in terms of making her job a lot easier, because she herself is excellent, but I think her equipment needs to be upgraded.

**Q: Any other weaknesses we want to mention?**

Janine: Coming back to the transplant issue, I think there may be too many people involved. There’s just too many people involved to say yes or no, because I was told by Dr. Q’s partner yes I can go ahead with the transplant. Three weeks later Dr. Q didn’t even know who I was or who and he says no, so they referred me…

**Q: So the same doctor said yes and no?**

Janine: His partner said yes and he said no and then I made an appointment to go see him, because I wanted to know why he said no, because he doesn’t even know me. My name has been bandied around, but nobody knows me, but they're going according to BMR which was created by insurance companies, which is not a text book for everybody. Everybody is built differently, everybody has got different bone structures, everybody has got different make ups and they're going by an average on something that was not even a medical thing really, which really upsets me. It just gets to me, you get told, you get left hanging in the air not knowing what's happening, because there are just too many people involved and nobody is getting back to you and telling you yes you're on the list, no you're not on the list. You get letters sent from your renal specialist to your GP saying we want you now on the urgent transplant list and then nobody gets back to you to say you're on the list now. I mean and I've got no one to speak to, who do I phone and ask? Am I on, am I not on? Nobody gets back to you and that’s my problem.

Sharon: I also have issues, I don’t know whether it’s part of your study, but with the transplant situation as well and it is to do with uncertainty. I know that they can’t tell you absolutely until you've passed all the tests and the reason it’s so successful now is they're so careful about what they do. But you do get kicked from pillar to post; the transplant clinic is run once a week through staff station four just behind there on Wednesdays and it seems to be that they’ve got people visiting who are still waiting to be transplanted or either on the list or off the list, you're not quite sure at any one. But they also need to follow up on the people that have been transplanted, so they seem to be attending the same clinics, so it’s always just mayhem. Everybody is always running late, there are so many people involved and they don’t seem to have a clinical nurse practitioner as a recipient coordinator at the moment. They’ve got a donor coordinator who seems to be doing both jobs and it’s just a mess in terms of feedback. You can’t get any feedback in terms of delays and dates and especially if you're in a live donor situation, you're affecting other people’s lives.

Janine: exactly

Sharon: And I’m in a real guilt situation because I’m affecting two other people’s lives in terms of when I get a date or some sort of idea of a date. And then as Janine said, like one person’s telling you there’s no problem and it’s ok and then they're saying well we’re not sure, because we still haven’t got this particular information back. So for example when I had my doctor Justin Vass a nephrologist, took out my right kidney and I had to be confirmed with no cancer obviously, I’d gone to see him post operatively, he said to me everything is fine, stage one encapsulated, I’m recommending you stay on the list and go ahead and then I go to see the clinic and I’m told well until we’re told back that it’s insitu and encapsulated, we can’t give you any extra information. And yet the clinic was the one who told me it was encapsulated and insitu before I got to see Dr. Vass. So there’s all this and now all the tests that I've done to pass are all 12 months old.

Janine: Yeah me too.

Sharon: And so now I’m starting to have to look at doing them again.

**Q: And it’s like the communication is not getting through is it?**

Sharon: The logistics are a disaster.

**Q: The communication is not quite getting through?**

Janine: No.

Sharon: The medical side of it seems to be very good, but the communications and the making dates meld and whatever is a disaster and you keep getting conflicting information and it ends up just taking more and more time and that then impacts back into a whole lot of guilt and whatever in your own situation. Like I’m in a situation now please John there’s a lot of people who are fantastic on dialysis, don’t let us scare you, but I am really in a dilemma at the moment in that if I don't get a date for the; my date for the transplant was May and then these other issues came up, if I don't get a date in October now, because of my donor’s circumstances, I’ve got to wait until next year. And therefore my head space, that’s why I’m seeing the psychiatrist and I have a lot of issues. I get cramps when I’m in the dialysis chair, so people come screaming around to pull me out of the chair with needles in my arms to try and get the cramps to stop and I have fistula problems and whatever. And a lot of this would be eased if you; you’ve got no control of what's happening in the transplant issue. And so it’s logistics side of it is a disaster.

Janine: I was saying to Dr. W the other day that a month in everybody else’s life is nothing, but a month in our lives means a lot and it just gets put off and put off and put off. My sister offered me this kidney over a year ago and nothing… It’s like an emotional roller coaster, you never know where you are and it’s just really really soul destroying sometimes, but you’ve got to get up and move on and take it on the chin.

Sharon: I wonder if there might be a difference in this between people who have potential live donors and people who are on the deceased donor list too? I think the issues that we’re talking about or me in particular is because of the live donor situation. If I was on a deceased donor list I don’t think I’d be quite as worried, because you just know you have to wait and it may not come up for seven years or something. But it’s a roller coaster if you’ve got a live donor, plus I’m 64, my potential donor is 66, so I’m now worrying also about age as well.

**Q: Let’s move on a bit, but similar sort of question, what do you find particularly frustrating about the diabetes and chronic kidney disease health services? I think we have explored some issues already, any other points people want to raise in terms of what you find frustrating about the diabetes and chronic kidney disease health services? Just to help you guys along a bit I just might mention some other weaknesses or frustrating points that people mentioned from other places. Some people mentioned a lack of appropriate education, other people mentioned no holistic care, so people just worried about a particular part of their body and not everything, some people mentioned poor communication between health professionals or with themselves. Other people mentioned long waiting times or too many appointments. Do any of you guys identify with any of those that were mentioned?**

Janine: Well we’ve got no real free time, because we’re on dialysis three times a week and on your free days in between you're seeing doctors and there’s just no free time available to us. And I prefer to do dialysis in the morning, because I can’t get home after dialysis nine o’clock at night and feed my husband. He can’t got to bed at nine o’clock with a full stomach. So that’s my reason why I do it in the mornings, but the Tuesday and the Thursday I’m seeing GPs and going for angiograms and having fistulas fixed or permacats fixed and it’s just all over the place, it’s just really… And also this transplant clinic on a Wednesday morning I do dialysis on a Wednesday morning. So I’ve got to go and do dialysis in the afternoon and then they still make you late for dialysis, because they're running late, it’s just a nightmare, it’s all a nightmare. I’m not talking about the dialysis as a whole, it’s your lifeline, it keeps you alive and that’s the way I look at it. It’s frustrating and all the rest, but that’s just part of life and you’ve just got to get up in the morning and get on with it. It’s just like I’d love to go away on holiday sometimes, but it’s impossible for me.

Joseph: On that issue I’m on the transplant list, this is donor transplant list and my specialist for that part of it is Dr. Stella *[MgGinn]0:58:01 and I have a lot of questions for her and I go in and I’ve done several tests and she follows it through and I follow it through and we keep going and I’ve just finished seven other tests again to keep the thing at a level. And the diabetes is the same, the kidney side of it is the same with Dr. [Khaled], so when I get back to the dialysis centre, then when I’m on dialysis, if I need to ask a question I approach the nurses, but I find that they don’t go into any discussion about any points that you have, because I notice that they have one way of the dialysis treatment. And I find it very hard to move them away from that and ask a question and say what if you do this, this and this, can it happen and they straight away pull back and say… And they don't say anything, but they go back to the normal routine you see. And then if you have something; I’ve asked about diabetes information as well in a few things and they wouldn’t elaborate anything on that side of it and they would say take it to your specialist when you go and see your specialist. So I think that is where as far as we as patients are concerned, if you are able to get a lot more information and discussions with the nurses, we feel a little more comfortable, because they're with you almost your full period, because I as an individual when I finish dialysis I can run from here to T City and back I feel so good, because I go to work and I work till about seven in the night. I finish dialysis at two and I work till about seven in the night in finance, that’s where I work and then I work on my off days as well and then on my weekends I work in the garden, I do garden work and handyman work and keep my whole body moving at all times. And I feel good, so I don’t; but when I come into dialysis I come ready with pointers that I want to discuss, but then I find that I can’t discuss it with the nurses, because they don’t venture out of their normal range. That’s the only thing I feel that would be interesting if they can help.

Sharon: In terms of the frustration thing, shift times is another issue that’s potentially very frustrating, because people have different; like I’m actually as I said I couldn’t cope with, although I’m fairly young and with it, I couldn’t cope with home dialysis, I live by myself. I have an upper arm fistula and there’s no way I would be able to put needles and things into my own arm, so I am stuck with either satellite or ward 6A. Ward 6A has worked out well for me, because I have back problems as well, but in terms of the actual shift times, sometimes you're forced into a situation where they're trying to push you into what's available. And obviously that’s what they have to do, because these dialysis chairs cost a fortune to run individually. And I consider myself lucky now, because I’m on a Tuesday, Thursday, Saturday afternoon shift and that actually fits in with my lifestyle and I try and keep those. But sometimes Satellite centre for example try and keep those for people who are working, because then they only have to take two half days off a week. So depending on, I think there needs to be recognition, because I had a lot of problems to start with, that different people have completely different not requirements, but preferred times in term of shift times. And if you can’t work out the best shift time for you and stay on that, that’s a major issue as well. So there needs to be some leeway or some ability to discuss with either the NUM or one of the coordinators helping you with the NUMs, because the NUMs just want to run their clinics, that’s what they just want to run the clinics. So we’re lucky here that there’s a couple, a coordinator, her name’s Lucy, who seems to be the bridge between the NUMs and the patients. So shift times can be a real frustration. The other thing of course is I’m very used to travelling and as Janine said, I try and keep my Sundays and Mondays with nothing on, sort of like a weekend. I’ve convinced myself I go to work, even though I’m retired, that I go to work for those other three days, because the days that are left usually are full of other medical appointments or even if you want to get your hair cut, anything at all, you need to jam it into those couple of days. So you don’t have any time to yourself. I found on the positive side of that though, that because I’m coming here to 6A, a lot of my medical appointments are here. So other than the transplant clinic which is on a Wednesday, therefore I’ve got Tuesday, Wednesday and Thursday, most of the doctors will try and fit in, and even this clinic now fits in, I’m going straight from here to dialysis, so it saves me a trip.

**Q: Does everyone find that it’s easy to access the diabetes and kidney services that they need to access?**

Janine: Yes.

Sharon: Yes.

Joseph: Yes.

John: Yes.

**Q: Let’s change tack a bit, let’s move on to your experiences with living with and looking after your diabetes and chronic kidney disease. What things do you need in place to look after your diabetes and chronic kidney disease well?**

Sharon: I don’t really need anything, because I’m not on home dialysis and I have an extremely good GP that I’ve been seeing for 30 years, understands all my health problems. And I feel because I am fairly assertive, that if I need to chase up on anything, all I have to do is ask my doctors.

**Q: Anyone else?**

Denise: I’m pretty well set up at home, because I do home dialysis and I live by myself. And I found that I’ve got to have double of everything, two next to me, because it’s hard to get off the machine and go and get anything that I need. So I had a neighbour across the road who was good, but she sold and moved to a retirement village, but I’ve got a lady next door who will come in any time anyway and help me out. But yeah because they provide everything for you at home and all the solutions and everything you need, the needles. The only thing I had to do was I had to get a chair that I could lay back on and that was mainly, and I put a special wardrobe in for the stock.

**Q: So just to clarify, when I’m asking a question I don't mean that need is not in place, what I mean is what things do you need that are in place or may not be in place, what do you need to look after your diabetes and chronic kidney disease well? So some people have mentioned education, support from family, support from health professionals, being motivated, being more educated, knowing what's going on and things like that. So what do people here think?**

Joseph: I think it would be interesting if you had a phone access to either a nurse or an intern or a doctor of some sort, just nurse, phone access, because if you have an emergency you can quickly get onto the phone and say look this is my problem, what should I do? And then you’ll be advised as to what it would <inaudible> and have to do straight to emergency as emergency can take a longer time. I find that’s about the only thing I can think of.

**Q: So do people identify with any of the factors I mentioned? So education or having support from family or friends? So what do personally find is important that you need or that you find important to look after your diabetes and chronic kidney disease well? So I’m not mentioning things that you're lacking, that need to be in place, I’m talking about what do you find important to look after your diabetes and chronic kidney disease well?**

Janine: I feel the public needs to be sometimes more aware of diabetes and what's required. If anything goes wrong, if you pass out or if you have a hypo or something like that, I think people should be aware that you're not falling around drunk or whatever, it’s a, I mean people should be wearing their diabetes bracelets and things.

Ioane: The diabetic side of things I think you need a dietician like once a month to advise you, remind you what to eat, because I thought my side is the food that causing all this diabetes, because when I was 30 years old I eat a lot, drinking a lot, didn’t look after myself. And I am diabetic too, I took, so I think if you need someone professional like dietician once a month to give you a call and remind you what to eat, because sometimes you lost a bit and eating the wrong food. But about the dialysis the nurses they help you with the dialysis.

**Q: So what you're saying is good support from the health professionals. So what do other people think, is support from family important or education important or having being educated important or having motivation important?**

Sharon: I admire Denise that she lives alone and is able to do the home dialysis. I would probably be able to manage home dialysis if I didn’t live alone, because I’ve never married. If I did; I rely a lot on my sister, when we do things together. Normally I’m completely self reliant, but for hospital admissions and stuff she will come and stay with me. But you can’t expect someone to come and stay with you forever, but I feel if I had someone at home I would be able to manage home dialysis, but that’s what prevents me from doing home dialysis.

**Q: Anyone else so in terms of what’s important?**

John: I tend to agree to Ioane that a dietician’s advice would be very helpful. As I was saying I think food is an extremely important element in a diabetic patient’s lifestyle and if I was reliant on some help I would look to a dietician to advise me the kind of breakfast I should have, the kind of lunch and dinner I should have that controls my sugar level. I had several fainting spells, some very dangerous ones which resulted in an ambulance being called. So I think it is important that one does not get into a hypo situation by eating lollies or having lollies on person and what to do when you get funny sensations. You're walking and then suddenly you feel wobbly, what do you do? Things like that. You need to be properly advised and like Joseph said I wish I had somebody I can ring up and say oh I feel like this now, right now, what do I do? You might say go and drink a glass of orange juice or something like that. Yes I think a dietician; I went to see a dietician a couple of years ago and he gave me a list prepared by the government of Queensland, unfortunately that was nonsensical. They told me that I can’t eat butter, I should eat margarine, but we all know margarine is no good for you, yet he has advised me to take margarine. And he told me no coffee and I can’t live without coffee. He says no chocolate, something that I love and for you to say no you shouldn’t have chocolates, I didn’t respect that fellow’s advice at all. But he did say the type of fruits I should eat, so I have stopped eating oranges and bananas and those that are high in potassium.

Sharon: Just on this dietician thing and you might even be able to clarify this for people, because this information didn’t actually come from the dietician, but because I was, but it’s been raised a couple of times, because I was on a conservative treatment and one of my drugs was Resonium for control of potassium, when I actually started dialysis and I’ve basically internalised most of the things to do with the low potassium diet, because I’ve been doing it for 10 years, you don’t really have to cut things out completely as long as you're only eating small quantities. I didn’t have a lot of trouble with that, because another renal problem is lack of appetite. But with reference to potassium and all the sandwiches and things that people have been talking about, I’ve been advised by the nurses, not the dietician, that when you are on hemo dialysis, one of the things that is actually controlled through the hemo dialysis process is potassium and in fact I was taken off Resonium. So you still have to be careful, but it seems that people are saying, that they’ve still got in their head that they’ve got to be super super careful with potassium and that’s not the message I’ve had once I started hemo.

**Q: If it’s medical advice we can talk about after, I’m just noticing in interest of time and some people need to leave. If it’s medical advice we can talk about it when we’re finished if that’s ok Sharon.**

Sharon: Yeah but that was just with reference to the dietician, the dietician did give me that advice about diet with reference to potassium.

**Q: What things make it hard for you to look after your diabetes and chronic kidney disease well?**

Joseph: To me it was a change of lifestyle, because my lifestyle was so different. I used to have client meetings, client dinners, client lunches and everything and so many different types of foods, so it’s a complete, completely different lifestyle now as far as that is concerned and I’ve followed that very very hard, but I follow it now and I feel much more comfortable since then.

**Q: Did it take you a while to adjust?**

Joseph: Took me a long time, took me at least five years. Five years and constant contact with my medical team and even with the doctors, the specialists, the nurses and all of that and asking a lot of questions and finally got down to it and now it’s fine I don’t have any issues as far as that’s concerned. So that’s one thing that one has to get completely used to it, as John was saying chocolates and all those type of things, they all go out in stages if you want to have a comfortable lifestyle and that’s what I’ve done.

**Q: Does anyone else want to mention what makes it hard for you to look after your diabetes and chronic kidney disease well? I think Sharon you mentioned and Janine mentioned also the getting into my head space makes it hard as well and associated factors, the mood affects things? So people have mentioned that definitely as well, other people have mentioned just having a whole, just the disease itself makes it hard, feeling washed out after dialysis or just having a whole lot of other illnesses gives them a lack of energy or conflicting information from doctors or lack of support either from doctors or from family or things like that. Do any of you guys identify with any of those factors?**

Sharon: All of the above and I also agree with what Joseph has just said, although I don't have family and children, I’m heavily involved in community, though I’m retired I’m heavily involved in community and for example two of the three things I’m involved in, I can no longer go to the meetings and whatever that are associated with that, because they're on when I have to have dialysis. So I’ve had to pick and choose, choosing the shift that I’m doing, I’ve had to pick and choose which things to drop out of and then the things that I’ve stayed in, I find it very difficult in terms of my energy levels to participate to the extent I did before. For example if I chair certain things which I do, I still feel the responsibility to do that, but I’m absolutely exhausted and then once again with me it’s the head space thing. I am probably a lot more negative than a lot of other people. Because people tend to want to live on for family and graduations and children and grandchildren, I say to them, and that’s part of the reason they’ve got me going to the psychiatrist I think, because I don’t have those things to look forward on to, I can’t get my head around the fact that if I have to be on dialysis for another 10 years or 15 years, if I’m in the same head space I’m in now, I don’t want to be here. So I think that’s party why they’ve got me seeing the psychiatrist as well.

**Q: What do other people think or what do people find makes it hard for them to look after their diabetes and chronic kidney disease?**

Denise: You did have to make a difference to your lifestyle, especially after you go on dialysis, I only retired last year because of the kidney I was advised to, and I only had six months off. I think I finished in March and I started the dialysis the end of November. So I really didn’t, I’m sorry I hadn’t done it 12 months before to do some other things, because I got so sick in that time and yet I did have one trip away to central Australia. I didn’t enjoy it, because I didn’t have the energy to keep going with it, now I think I can do it, but I can’t do it, because being tied to a dialysis machine will make it difficult to go away. I think you can do it, but it’s probably difficult to organise dialysis in different places.

**Q: Other people mentioned having to organise their lives around dialysis, do you identify?**

Denise: Yeah had to and that’s why I’m glad that I do it at home, because now I can pick and choose what days I do it and I often do it every second day, but every couple of weeks or so I will have two days off together, because I find I need those two days off to recuperate. And to do things really.

**Q: Ioane and John have you got anything you want to mention?**

Ioane: Most important thing for me is that travelling, because before I went to dialysis I used to drive to my homeland twice a year back home, 20 whole years now in Australia, used to travel twice a year.

**Q: Yes back to Samoa yeah.**

Ioane: Now on PD I take a pallet of fluid.

**Q: Are you on PD or hemo now?**

Ioane: Hemo.

**Q: But when you did PD yes?**

Ioane: I used to take a pallet, just open on plane and also I carry 50 kilos on a plane to just take my own stuff, but not anymore now. My last visit was 2010, so going three years now.

**Q: So you can’t travel overseas because of the hemo yes.**

Ioane: Yeah supposed to go do that tomorrow, but because Dr. *1:20:55 stopped me from going, just waiting for my operation, so…

**Q: John have you got anything you want to mention? As in what things make it hard for you to look after your diabetes and chronic kidney disease?**

John: That’s the major problem, fortunately my wife looks after me, boils all the vegetables and throws away the water, I mean a lot of patients tell me that food is a major factor in their lives, that tasteless food. I wish some specialist in diabetics would invent foods that are tasty. I mean I’m used to having good curries, but now everything is bland and no salt and… But anyway I’ve been tolerating it, because they say it’s good for me. What I miss most is fruit actually. I was also given Resonium, I was due for my lung operation when they found out my potassium level was very high, so they had to keep postponing week by week until my potassium levels came down. So it is complicated actually, you lead a life full of restrictions. Other than that I pray to god I still have a life.

**Q: Going to move on now for the interest of time and thanks to everyone for sharing so honestly. I’ve given us 20 minutes for this last question and I know we’re ending up soon, but I’d love you to concentrate on this question, because this is really why we’re here. So this is the main question, it’s a bit fun as well, but you will see when you hear this question you’ll see why I’ve been asking all the other questions and where this is all heading. Now imagine if you became the director of the Hospital 3 and you had the power to change things about the diabetes and chronic kidney disease health services. If we were going to design a perfect health service which met all your needs, what would that include? I’d love you guys to concentrate and brainstorm, flash ideas around, especially from what we’ve been discussing. We’ve discussed a lot of weaknesses, things which could be improved, so let’s open the floor up for comments.**

John: I’d like to ensure that there are many kidneys available for transplant patients, including one for me. And I would ensure that there are as many dieticians as possible employed by the hospital taking care of a certain number of patients, so they can monitor the progress of the diets in each of these patients. And I believe that if the patient eats the right kind of food he will enjoy life better and the necessity to go for numerous pills and having insulin at a later stage can be avoided.

**Q: How about individualised advice? I think it’s sort of mentioned that some people got very general advice and they didn’t know what to do in certain situations, would that be helpful as well if they individualised and tailored advice?**

John: Yes absolutely.

Sharon: It ties in with your question, because to me the major improvement is at the staffing level, mainly nurses with the various units, but not just the day to day nurses, the more, a lot more clinical nurse coordinators. So that what they actually do is they're the ones that are then able to look at you as an individual, so there’s coordinators that are aware and they do things like pull in the dieticians and tell the actual clinic that there’s an issue with you individually. But they’re also overloaded, that although that the skeleton of what's needed is there, there’s not the capacity for them to take it so that you are being looked at as an individual case the way your GP is able to do it. They're aware of your history and tying it into the other services.

**Q: So just to get some idea and I don’t want to be putting words in your mouth, but are you sort of talking about someone that especially once you reach the complexity of health that a lot of you guys have, like an individual person who will help coordinate your care and know what’s going on and help coordinate it, so ok you've got this appointment, you’ve got that appointment and stuff like that, is that what you're talking about?**

Sharon: Not necessarily at the appointment level, at the treatment level. And that’s why I think the clinical nurse coordinators do depending on what specialty they're working in, would be the best people for the job, because they understand…

**Q: So like a clinical case, like a nurse…?**

Sharon: A clinical case manager almost yeah and I think the coordinators are able to do that, because they also then are able to feed in for example through your clinics to chase up on the registrar or to chase up on a dietician to come and give you individual information. So if those people are available, the Allied Health professionals and whatever are actually available; you still need someone to get them to you.

Joseph: I would also like to see that the nurses get some sort of customer relations training, so that then they can also handle the different people when they come in for their dialysis and if they're diabetic and all of that. They’ll be able to handle them better, because sometimes the nurses do lose a bit of patience, because they find it difficult to be able to get their way of thinking into the way the patient should think. So if I was in a situation where I can bring that in, I would and then also introduce a system where the patients, one or two patients go into a seminar with the nurses, so that they can do a lot of their input as well and help the nursing and medical staff to see it from both sides of the picture. And then offer employee of the month and patient of the month incentive in some form of the other medically to boost that feeling, because sometimes see we are individuals in with a debilitating illness and when you have diabetes and chronic kidney disease, it’s actually like closing the door on your life. So you go in there, you're with this in your mind, but then you realise suddenly the nurses are there to do a job. And when I was put into training with SDC, I was under the NUM on that section, when I finished off I did a big write up about the NUM who trained me. And I said you get trainers of horses and animals that are paid millions of dollars, but this person trained me to look after my life and I really took in a lot from that. And I thought when I gave him the write up after I did it he said I only did my job, I said yes but you did a wonderful job. And I said you should be told that and that’s what I’d like to see, where we can tell the nurses look we are doing this, we are the people who are sick and we want to see what we can discuss and come out of it. So I think I would bring a lot like that where there is customer relations and a lot of information to be able to boost our feelings, because some of our patients and I’ve come a long way and I help a lot of my patients in dialysis, I go down and talk with them and we have a lot of jokes and all that and I try to boost their feelings. So I’d like to see that where I can do a lot of input into getting our, as patients getting our lifestyle up and up a bit.

**Q: So just to make sure, I understand your second point, so you're putting in some incentives for the staff to improve morale and I guess also to improve the service offering and also some facility for peer support perhaps.**

Joseph: Exactly.

**Q: For you; for people who have gone through something to help others. And was your first point a wider scope of practise, so that the dialysis nurses are also trained in perhaps a bit of diabetes or things like that is what you're saying?**

Joseph: That’s right and just to elaborate on that, when I was in hospital and like John being introduced into dialysis, I was scared stiff until I had the visit from the NUM at the time of the dialysis centre. And she spent one and a half hours with me and went through everything, listened to me with everything I said for about half an hour or so and then she spoke about it and made me feel good about it. And that changed my whole lifestyle and I ended up where I had told my family that I’m not coming back, that’s the end of me, I ended up changing completely and today I am so happy because of that. But I nominated that NUM as the Australian nurse of the year and she came in the first ten, because of the nomination. It was so good, it made so much difference. So I thought this is interesting, because this is what I’d like to see with the staff, so that the patients can feel a lot better. Because it may not be the word C A N C E R cancer, but it is a debilitating illness. And recently I did a write up for a flyer for the newsletter in the thing and I got myself with the photo next to the machine and I said till death do us part. And it was a hit, because it; and I feel that’s what I want to see, I’d like to do it and I’m going to do it, even if I get a transplant and I’ve gone, I’m not going completely, I’ll be back there, I want to see this happen.

**Q: What do other people think in terms of improving the service or designing a perfect health service, what would you guys include?**

Denise: Because I know with the renal service for the home dialysis patients, they have a nurse on call 24/7 and you can ring them any time, same with the technicians and they're very good and they make home visits every three months. It would be good if there was some sort of service like that available for the diabetics side, where you could ring a nurse or someone for advice or just help to talk about things yes.

**Q: I think there actually is for the diabetes, if you're linked in with a… Who do you see for your diabetes?**

Denise: Sally Duke, but I see her over in the rooms not here, I’ve only seen her once, because I used to see Dr. Steel, but he’s retired.

**Q: Any other things that people would…?**

Ioane: I think John say it all, but when I was at the community centre dialysis, short staffed, nurses on, sometimes about two nurses. It’s not on and if someone is sick on a machine they ban it sometimes.

**Q: I think before someone mentioned about better education especially around diet, someone also mentioned whether diabetes doctors could come out to the dialysis centre. Is that something…?**

Denise: Yes I think I mentioned that before.

**Q: Is that something you would like to…?**

Denise: Yeah it would be very good for the patients also to have someone to talk to, but also to review them over there as well. I know when I went to the home dialysis, I didn’t have my own renal physician, it was the Dr. *[Sh name]1:34:24 that looks after those, but I still had my own, I saw her at times as well.

**Q: So we’ve mentioned case managing, we’ve mentioned incentives, wider maybe better upgrading of skills with some of the dialysis nurses, we spoke about maybe the diabetes team coming out to satellite service.**

Sharon: Staffing.

**Q: Yes staffing, I’ll just mentioned what other people mentioned.**

Sharon: I’ve got one more too. The way, once again this is sort of a resource, but it’s more of an equipment issue, because of the number of chairs that are available and the fact that you’ve got shifts and the fact that some people are on four hours dialysis, some people are on five hours dialysis whatever, you're given a; I would change this somehow, I don’t know how I’d change it, but you're given a time to turn up in. For me it’s between two and 2:30, but what happens then and it’s only reasonable is because it’s an afternoon shift, people are coming off the morning shift, so if something’s gone wrong, people may or may not be running late for a particular chair and also they put the people onto the machine first to have the longest time. So I’m a four hour person, if I turn up at two o’clock which is what you're supposed to do, I can be sitting waiting and getting all upset and everything for anything up to an hour. That’s unusual, but usually between half an hour and 45 minutes to actually end up on a chair and that’s because of the availability and whatever within it. And coming off is the same thing, you’ve got a limited number of staff who are able to take you off the machines and people are coming off and often because of the way they’ve gone on, a whole group of people are coming off at the same time. So you're sitting there with your machine dinging and you're unable to come off the machine and that’s really really stressful as well. So if there was some way of making that a thing that flowed better in terms of little time slots or something like that on the basis that things could still go wrong, that would make a huge difference to me psychologically. So I think if they could think around the logistics of those situations, I’d be looking at whether something could be done about that.

**Q: Now you mentioned about waiting times for dialysis, has anyone ever had problems in waiting times to see their doctor?**

Sharon: Oh god yes, depends on where you see them.

Joseph: I haven’t had any problems.

**Q: So no one?**

Denise: Not really.

**Q: But you have Sharon.**

Sharon: Specialists.

**Q: What situation was that, was that with the transplant unit?**

Sharon: The transplant clinic and obviously if you’ve had a specific procedure done as I have done with two like they're doctors who are, obviously I think they're VMOs, so they’ve got their own full practises, plus they're feeding into the hospital. So post operatively if you’ve got other things done, trying to get the appointments, it’ll be a month or six weeks after you’ve been discharged that you've got to wait to get the appointment. And if you're seeing someone privately rather than, well the waiting time, I’m seeing Dr. *[Stilham]1:37:52.3 again through the transplant clinic, that’s difficult enough. If I wanted to see her now that Yvonne is on maternity leave through the community health centre as a new patient it’s like three or four months wait.

**Q: Now I’ll mention what some other people mentioned to see whether you can relate to this. So other people have mentioned better education, cheaper parking, some people mentioned combining the diabetes and chronic kidney disease health services. Other people have mentioned regular nurse educator contact. Do any of you guys think any of those ideas were good?**

Joseph: I think that combining diabetes and chronic kidney disease is excellent, it’ll be excellent if it can be done like that and have medical staff that can discuss both sides of it evenly, because when you have diabetes and you're having chronic kidney disease, it means a hell of a lot to one’s life and the changes. So that combination will be wonderful I agree.

**Q: So what do other people think? Some people mentioned a combined clinic or something like that.**

Sharon: The only issue I have with that combination is that you wouldn’t want to de-emphasise the chronic kidney disease that’s not caused by diabetes and there’s a lot of people who have got chronic kidney disease that are not diabetic. And on the other issues that you raised, if the parking wasn’t sorted out, the parking is now Wilsons Parking h ere, and although you have access to, if you don’t have a disabled parking sticker, because you're here for hours and hours and hours and hours, you do have access through the social workers to a ticket that’s a weekly ticket and it’s only $7.70. But you have to find a place in the parking station. Now it’s ok if you’ve got the disabled sticker, because there’s quite a few disabled parking spaces around and so as long as you can grab one of those as they turn over you're ok. But having the $7.70 ticket is not really much help, because there’s no parking spaces until you get to the roof and then you're coming out at 8:30 at night and wandering up through four floors of a deserted parking station. So I think it’s a huge impact both financially and when you're absolutely exhausted at the end of the day to be able to get to your car fairly easily.

Joseph: I’ve just got two sort of to cut in on that, as far as we are concerned throughout the community health centre, we get free parking.

Sharon: If it’s available. If it’s there.

Joseph: No we get free parking, we have a thing that is issued.

Denise: But you can’t always find a park.

Sharon: But you can’t get a park. I was there…

Joseph: But we can park anywhere, we don’t have to park in the car park, we can park on the street there, we can park in underneath the community health centre building and it’s the enlargement of that. I just put that on the dashboard and that entitles me to…

Sharon: I spent three to four weeks at the satellite centre and they only had two of those left, those parking exemption tickets left and nine times out of 10 you could not get in, if you're afternoon shift you could not get a park underneath the building, had to try and get one in the street. And they were the ones who encouraged me to get the disabled parking sticker, because then they could have their one of two things back.

Joseph: I agree, the parking does, they only have 15 because there are 15 people on each shift at the community health centre, but once it increases, which they're hoping it’s going to be increased to something like 40, then it will be different.

Denise: Because that car park it covers not only the satellite, it covers everyone coming through that building.

**Q: So has anyone got anything else they want to mention, especially when we talk about better education or the combined service or parking? What do people think about the better education or combined service or parking? What do other people think?**

Denise: There would be better education or more education for I suppose ongoing education would be of benefit to the patients yes.

Sharon: As you come up the transplant clinic and that is better logistical coordination. I find most of the medical staff wonderful, I find the logistics of getting else done almost impossible.

**Q: So other people mentioned better communication between all, better medical record some people mentioned. John or Ioane have you got anything you want to mention about any of the topics we mentioned, so parking, education, combined service?**

Ioane: I don’t know, that’s your staff, nurses, I think that’s the main thing in the community, there’s only about two people there. The other shift come in, even this sitting outside, because only two nurses.

**Q: John?**

John: I think better education, I’d say education is the key to understanding always. I’m slowly beginning to appreciate how efficiently the dialysis system is being run by this particular hospital and the satellite stations and there is one in Lynnfield that I might go and register myself there. I have already been there and they’ve been very welcoming of me and I’m less scared of dialysis than I was before. Through the lady called Knagge, what's her name? There is a lady here called K N A G G E, I think that’s the name of the lady. She was very helpful to me, she explained in great detail what happens at one of those dialysis centres and how you are assisted in the peritoneal dialysis and as well as the hemo dialysis. So I think more education is warranted, people will have piece of mind at the end of it all.

**Q: In some other places they’ve got a podiatrist and dieticians that come out to people with dialysis and nurse educators as well, do people find that useful?**

Sharon: Yep.

Joseph: Excellent, I in fact have had the dietician and she comes out, now she comes out more often than before, because we have come up with good plans and I appreciate that very much and I look forward to it and I advise any of the other patients if they wish to get some details, to follow suit.

**Q: This is regularly though, regularly.**

Joseph: Yeah.

Sharon: I think the podiatry is a good idea as well, because especially as people get older, it’s very hard to reach down to cut your toe nails and whatever anyway and if that’s complicated by diabetes or something else, it’s a major issue.

**Q: And I just wanted to see what John, Denise and Ioane, what would you guys think of a combined service or a combined clinic with renal and diabetes? Would you guys think it’s a good idea, bad idea or indifferent?**

Denise: I think it probably would be a good idea yes. Just be more communication between the specialists yes, that would be good.

John: I’d welcome that if at all there is a facility like that, I’d like to go and come and attend focus meetings like this if there is any future. I learnt a lot by attending this particular meeting today.

**Q: That’s good to know.**

Sharon: I just want to reiterate though with reference to not being a diabetes as a cause, that if it moves too far towards a combined service I’d be very concerned that people who are non diabetic, kidney disease would end up on the periphery.

**Q: I think diabetes is the, I do see your point, diabetes is the commonest cause of chronic kidney disease now, but definitely do see your point. Has anyone got anything else they wanted to mention, any burning points that they came wanting to say but they haven’t managed to say or any other questions that I should have asked you guys that I didn’t and you guys want to mention something before we close off?**

Sharon: Just very quickly and it’s just emphasising what was said previously about travelling, if your life has been, you’ve been looking forward to doing lots of travelling and/or you have been doing lots of travelling, there should be information available on what… I mean there’s a very small amount of information available that you can for example have a guest chair somewhere else, but there’s not any real information about how to go about doing that and whether you’d be putting people out to help you try and organise it or whether you have to do it yourself. And that’s been a huge detriment in my lifestyle as well just like you.

Denise: I do know that there is a service available, because I know a girl who has dialysis at Prince Alfred and she goes on several trips with a dialysis group. It’s not particularly for Prince Alfred, I forget now, you can bring it up on the internet as dialysis travel, and you can register through them as well.

Sharon: You can but it’s not easy.

Denise: She seems to go a lot of trips, she’s just been to Italy and back on hers.

**Q: Talking about travel, I know we haven’t discussed this at all, does anyone find any trouble travelling to their dialysis places or to the Hospital 3 or any people find trouble with cost of travelling or parking and stuff like that?**

Joseph: No I don’t.

**Q: Or waiting times and stuff like that?**

John: No.

Sharon: While ever we can drive ourselves or someone bring you in, I don’t see it as a problem, but otherwise we’re going to end up dependant on ambulances.

**Q: If no one’s got anything further to add we’ll close up. We’ve just got some quick surveys to fill out.**

END OF TRANSCRIPT

**Focus Group 12**

**Q: Okay, do you want to maybe pop it further, maybe point it that, no this way? So that the speakers, yeah, that way perhaps. Okay, well thank you everyone for coming, welcome. So look, this is a focus group discussion, so essentially what happens is I’m going to ask a couple of questions and then you guys can answer and it will start a discussion, there’s no right answers at all. We are looking for your opinions really, and your experiences, so there’s no right, no wrong answers and we want you guys to be as honest as possible. As we said before, all the information you say will be de-identified so it can’t be traced back to you, especially now that you’ve got <laughs>, you’ve got all the fake names. So anything you say will not impact on the quality of care that the doctors directly give you, but as in it won’t result in any negative things. But, this project, the purpose of this project is to improve healthcare, so any information that you do give in the longer term, although de-identified, may contribute to improvement of healthcare overall.**

**Now I guess the other, other quick sort of things, I guess just when we are running a focus group, just in terms of laying down ground rules, try, I mean I know most of you, I know this won’t be a problem with this group, but sometimes people do get excited and, and sort of jump in and cut in. If we can try and avoid that, that would be good, so let everyone finish what they have to say, and remember, you know, you are all experts in your own sense because that’s why we are asking you because you’ve got experience, you’ve received the care, you know what you’ve received, so we really want to hear from you. So without further ado, let’s skip to the first question.**

**Q: Obviously there’s different experiences here with the care. So now let’s quickly think about your experiences that you have with the diabetes and chronic kidney diseases services, here at Hospital 4 which could include dialysis, could include clinics, stuff like that. But based on your experiences, what would you say are some of the strengths of the diabetes and chronic kidney disease health services that you’ve experienced?**

Marg: You want to know medically?

**Q: So some of the strengths, so what do you think is good about the services, the strengths?**

Joe: Well in my case, I’ve got onto insulin in 2006 because I had a stroke and it was recommended that I switch from tablets to insulin and I’ve had to take two types of insulin…

**Q: Yes.**

Joe: NovoRapid and Lantus.

**Q: Yes.**

Joe: sometimes worked and sometimes it didn’t.

**Q: Yes, yeah, but what, okay, what are some, so from your experiences with the services, so with the dialysis, going to the clinics, what are, what are some things that, what are some weaknesses of the services that you’ve experienced? Things that could be improved, weaknesses?**

John: Well the first thing with the clinics is how long it takes, even though you booked in at a certain time, it can take up to three hours to get through your appointment.

**Q: Is this the diabetes or the kidney disease, or both?**

John: It was the diabetes, except for the last time, there’s, with the, they’ve got a research project going on so there was extra staff.

**Q: Okay.**

John: But normally there’s not enough staff and it must be they’ve got too many patients because you, you have a fixed time to come in…

**Q: Yes.**

John: And they tell you don’t come beforehand, but then it can take up to three hours, as I say, to have your appointment.

**Q: Yeah, what about the kidney disease clinic?**

John: Oh, I get that through privately, so here in the hospital with Fiona Brown.

**Q: Okay, so that’s quite…**

John: She does a great job, and she’s got her fingers on the pulse, because that’s one of the strengths of this hospital here is that all the facilities, with one exception I’ve found out, are here, so it’s easier to get referred to the other people to treat you.

**Q: Yes. Anyone else can comment on that?**

Nitz: Yes.

**Q: Does anyone agree with John that have come to the public clinics and found that they’ve been waiting a long time?**

Nitz I agree with John, it’s Nitz, I agree with John, with the kidney problem – I had renal failure, I find out too that it’s a big waiting list, long time before they see you. Say for example, like when you go to the clinic on Monday, it’s packed with people and you waited and you waited and you waited. The earliest you can see is about two hours after the set appointment that they give you. You get different doctors most of the time, of course they’ve got different opinions… (Lorraine nods)

**Q: yes.**

NItz: What I don’t like is some stage they get a blood test with you, you got for a blood test and like in different pathology, because it’s very far for me to go to the Southern Health pathology all the time for blood test, so I when I go to Dorevitch, they have to ask for the result of it and the problem is sometimes they can’t get it, or sometimes the Southern doctor will say “this is not the test I wanted from you. I want another one.” So they just finished looking at you, you just had a blood test a week ago, he want you to have another blood test the next day. I mean, can’t you see that it’s supposed to be, they can send test through to same doctor who is familiar with you? Like different doctors come and see you, and you get a different opinion and they give you a different medication. Another doctor come along and he say “no, this medication is a bit stronger for you, or lesser for you. I would like to change that into this one.” How many times I changed my medication like that too? (Lorraine nods) Sometimes it doesn’t give you enough time to settle, like you get nauseous because this sort of medication is a bit strong. They said they base it from the particular blood test they have recently from you, so wouldn’t it be nice to just a few good, you know, permanent doctors that they can see the same patient all the time? Like it’s more familiar of you than, it’s nice to have some new doctors sometimes, that they test one opinion, that sort of things, but I don’t fancy the idea where you’ve just had a blood test this week and I have to go and have a blood test again the following day because it’s not the same. You get exhausted. (laughs and so does Lorraine) It’s not only that blood test sometimes you go through, you’ve got different things that you get, ultrasound, you get this. Like how many times do you go to the hospital and all this too, you try to cooperate, but you can only take as much for yourself to go through it all these tests and all those things that you have to go through.

John: But if you are in hospital, you would be having a blood test every day.

NItz: Oh yes, I did try that one too. When you wake up you have a blood test before you have anything else <laughs>. It’s not easy, but you know, with us, like I, I am single and I only look after myself, so driving from here all the time to there, you know what I mean? Driving from, from your place to here and you go home in the peak hour in the afternoon, sometimes you get nauseous along the way, it’s exhausting.

**Q: Mm, so has, does anyone else identify with Nitz? So Nitz talked about how she’s seeing different doctors and some are saying, give differing opinions, is that right?**

NItz: Yes.

**Q: In the, in the public clinic. Does anyone else identify with that?**

Marg: In my case I think it’s very convenient, it’s quite close to where I live. I live in a retirement village, and the unit is just about ten minutes drive from my place, and any blood tests are all done in the same place, while I’m dialysing, they take the blood and send it in for tests, so I don’t have to go to different places.

**Q: Who looks after your kidneys again, Marg?**

Marg: I’ve got Dr [Cohan] (Frankston private)

**Q: Oh, so it is..?**

Marg: No, Dr Cohan looks after my diabetes in Frankston.

**Q: Yes.**

Marg: And there are three doctors who come twice a month into the integrated centre, and we go there and the doctors see us. So it’s all…

**Q: For the kidneys?**

Marg In one place.

**Q: Yeah, yeah. Do you have any issues with different doctors saying different things, or waiting there?**

Marg: No, I haven’t, so far I haven’t had that problem.

**Q: Okay.**

Marg: And it’s very convenient because in the last few weeks I’ve been very short of breath, so they were able to phone the doctor straight away and find out what they are to do and increase the tablet, the corresponding tablet.

**Q: So basically where you have dialysis they also review the, review haemodialysis?**

Marg: Yeah.

**Q: Okay.**

Marg: So I think a few more centres like that for, for you know, for renal or for diabetes, will be very good because I started off at The Alfred, then I went to Caulfield and then to Sandringham, and then finally they got me in at Cranbourne, because that’s where I live. So if they had units scattered around, it would be much easier for the patients.

**Q: Does anyone else identify with Nitz in terms of..?**

Lorraine: Even with me, though I, even though I come from Austin, I dialyse at Hospital 4b, Hospital 4b, so all my blood tests I don’t go, Austin Hospital will write to them for my renal, for my transplant stuff, but all the other blood tests are being done in Hospital 4b, and it comes here to Hospital 4.

**Q: Yes, yeah.**

Lorraine: And they will look at my iron levels, and I haven’t gone anywhere else. Even when I had an infection in December and I was hospitalised for a long time in Austin, and when I was sent home from Austin, every time I went to dialyse, they would do the, Hospital 4b, even though ti was – they would do the blood tests and look at my CPR to see whether the infection was settling down, or going up. So it’s been quite…

**Q: So it sounds like it might be different for the haemodialysis patients?**

Lorraine: Yes, because…

**Q: John, I think you are receiving peritoneal, do you have the same issues as Nitz, about the waiting time? About the different, seeing different doctors, different opinion?**

John: Yes, I was going to say that that’s a real problem, the different doctors. They don’t seem to know, I suppose they are rushed, know what’s happened the previous clinic time, and I wonder at the same time, most of them seem to re-write the information, it’s up there on the screen, but they handwrite it again. All your past details on, on the sheet, and I think that takes up a lot of time.

**Q: Is that, that’s for the diabetes clinic is it, or for the renal clinic?**

John: Dia-…

Nitz: I was talking about the renal.

John: No, I’m talking about the diabetes.

**Q: Okay.**

NItz: Oh.

**Q: But they are both public clinics I guess?**

John: Yes, yes they are. Here, at this hospital..

**Q: Yes, yes, and so you see different doctors as well like Nitz, and do they..?**

John: Every clinic you, you see different doctors. So depending how frequently you are coming, it’s always a different doctor.

**Q: And do they give you different advice and stuff like that?**

John: Yes. There is a confusion there about the advice, and I seem to think it is in the training, that they come from different institutions, and it may be what this hospital ought to do it have a regular training session for all the new doctors coming through just for things that can be different, because they can be contradictory at times.

**Q: How about yourself Joe? You’re, you’re seen in the public clinics for diabetes and kidney?**

Joe: Mostly yeah, yeah.

**Q: Yeah, what have your experiences been? Similar to John and Nitz, or different?**

Joe: Well I mean in the case of renal, it’s the time factor. You’ve got an appointment at two o’clock and you see, see the doctor at 4:30. You’ve wasted two and a half hours, while you could have done something better with your time. Then this time around five minutes to see the doctor, who have a different view of the doctor you saw the last time, and you don’t feel any different, but from the results they’ve got from blood tests, they can only tell you what they see. Two doctors won’t have the same opinion, oh no, [as many models, same opinion]*0:17:29.6 and so it’s a bit confusing in a sense if you are the patient to know what is the real reason of them telling you all this. Yeah, and sometimes I find it confusing myself, because I didn’t know whether this was correct or not sufficient enough to, to diagnose what I have got or, because I’ve got multiple things. I’ve had a by-pass, I’ve had two strokes, I have renal problems and I’ve got hypertension and so on and so forth, so with all of those I’m taking medication and they probably overlap with some of the other medications. It’s confusing, do I need to take all of the medications that I’m taking or not? Yeah.

John: I would agree with that, that when you have the different doctors there, they come from their speciality and the patient isn’t really treated holistically, most of us have got lots more diseases than the particular one we are being treated for, but the doctors that are in a speciality, they only treat that one. I think that’s the major failing of the whole system is that they don’t treat you holistically, they need to connect you up because probably along the track, all the diseases relate to the same general core. So if they are just talking about down the end of the line, it may not refer to the things that are more important, and that’s the main thing I notice in the clinic, that they just treat you individually for that problem.

**Q: Does anyone else identify with John, what John’s saying about a lack of holistic care? It sounds like what you are saying is that each person you see is just worried about their little bit and that’s about it, is that right John?**

John: They only worry about their one little bit, and they don’t seem to know about the other little bits. I know they must have some training in it, but their specialities take them along that path. Now…

**Q: When you say they don’t know about the little bit, is it as in they haven’t received correspondence from the other specialists that are looking after other bits, or they don’t know anything? Is it the knowledge base, or when you say they don’t know anything about the other things that are going on, is that like they don’t know about what’s going on in your, in your body, or more they don’t know what’s going on in general about..?**

John: In anybody’s body, because they are not interested, they are a specialty in that area, and they can’t possibly look after all these. Whereas if you go to a specialist outside, they generally have an awareness of all the factors involved in your disease and your treatment is holistic. The tablets you take, whether they clash with other ones, the type of treatment…

**Q: Are you comparing your experience, because you see, you see Fiona Brown privately, are you comparing your private care with the care that you get in the diabetes clinic, are you?**

John: No, what I was illustrating is that’s, some people do it, the outside specialists, the private specialists, does the whole lot as part of the termination and the treatment, whereas the clinics are specifically for that individual field, and you can get contradictory information, you can get people giving you different tablets or different doses of tablets, or different amounts of tablets. I find that a real problem, because they are not getting at the key. I go there, I have lots of things, a triple by-pass, I have nerve problems in my feet and hands, I have eye problems and all these things, and I’m just recovering from a bowel operation, so I need all the things looked at rather than just one individually. They all need to be done together, so I notice in the correspondence it says “combined clinics”. I think it needs to be a bit more than that. You need to look at the types of diseases that are associated, and they need to be considered at the one time when you go to the clinic. (Lorraine says MM and nods)

**Q: Does anyone else identify with John, or agree with John?**

NItz: Yeah.

Lorraine: Yeah.

Marg: Yeah.

Nitz: Yeah. It’s different when you go private practice, because they are more focused onto your own whole, how it’s all linked. When you go to, it’s private, I don’t know, that’s what I feel sometimes that you like being in a processing thing, they can just see this, this, this, this “this is what you’ve got to do, you’re blood pressure is just this, this and…” If I ask the doctor, like sometimes I ask, because my kidney still works a bit, sometimes they say “oh you’ve got 17% working”, sometimes they say “oh, 17% is working”, and you ask the doctor, “how many percent is still the kidney works?” “Oh, don’t worry about that, because that’s why it fails because you’re diabetic, isn’t it?” You can’t hint like that. I said to him “I would like to know.”

Marg: Yeah, I was told the same thing.

NItz: You should know, because that’s why we’re here for, what improves – “oh no, don’t think about that”, you can’t, so I sit longer and ask more questions because I’m not satisfied of being, you know, we might be sick, but you know, not stupid, being pushed around, considering I’ve been waiting for him for about, over two hours and a half and you’re the last one waiting there, doesn’t have any more patients in the waiting room when you get out, so why rush you like that? That’s what I ask, especially sometimes you find some doctors like that.

John: Yes, some doctors shouldn’t be doctors.

NItz:Yeah.

Marg: Why do they shunt you off like that once you’ve got renal failure?

Nitz <Laughs>.

Marg: And you start dialysing, and I came, I came here to get that blood test to see how much my kidneys are working, because I’m still going to the toilet.

NItz: Mm.

Marg: And they turned around “don’t, don’t worry about it. Your kidneys are gone already.”

NITz:: Mm.

Marg: You know?

Lorraine: I got that too.

Marg: They just wouldn’t tell me how much, is it 5%, is it less than 5%, or all, or..? I would like to know.

NItz: And yet some doctors will tell you.

Q: Mm.

NITz: “Oh, your kidneys are still working but 17% of it. Oh, it’s a bit better.” You are used to that sort of thing, and then somebody, someone you just met, he just push you like that, that’s why you are there for, because you would like to know, if you fully gone or what, or something. You know, that sometimes it comes up and down, but, and then also constances you wouldn’t push a person because you don’t want him to talk to him about what you don’t want to talk to him. As a patient you would like to know what you feel sometimes, how come you feel nauseously like this? You feel this like this? How come you not good like this after you do this and that? (Lorraine nods) You would like, as a patient you would like also to experience what’s good into your well-being. (Lorraine nods)

John: Do they give you any booklets or anything when you come to the clinic?

NItz: Yeah, I get all, everything.

John: Because some of the, the booklets say that that nausea is just a part of, of the disease.

NItz: Yeah, it’s because, because a part of the medication, it’s a part of this and that.

John: Oh no, the disease itself.

NItz: Yeah, the, the disease itself, we are aware of that, but the question is, sometimes it depends on the medication (John nods) that they give you, if it’s a bit stronger, like I get given Lasix 50, and one doctor jumped me to Lasix 500, well, that really melt my head, so that sort of thing.

John: It’s the whiskey you put in with the tablet.

NItz: <Laughs> You wish sometimes <laughs> (so does Lorraine and Marg(.

**Q: So I guess what seems to be coming is that a weakness seems to be a lack of someone exactly knowing what everything, sorry I’ll rephrase that, a lack of, a lack of holistic care? Like there’s not one person that seems to know what’s going on with everything, is that right?**

John: Yes, that’s what’s been said, but some doctors are different and they don’t impart knowledge. They seem to think it’s, it’s too precious that they keep it. I’d say that’s one of the major problems of the clinics, is the information feedback to the patient is not good enough.

**Q: Yes. Does everyone else identify with that as well?**

NItz: Yes.

Lorraine: Yeah, Marg: yeah.

Joe: Yeah.

John: And there’s one, two other things, there’s not enough doctors and not enough staff. There’s not enough time.

**Q: Yes, do you agree with that as well Joe? Like I know, I think Lorraine…**

Nitz.

**Q: Nitz and Marg have just agreed with John’s statement, do you agree with that statement as well Joe? What John’s just said?**

Joe: More or less, yeah.

**Q: Yeah, now I think…**

Joe: When you consider that you’ve got an appointment for the renal clinic, you normally expect to see a doctor whose practice is regarding that speciality. You may, you may see what’s on the screen as part of your whole history, but they are not connected to it because that’s not their specialty. They may know about it and if you ask them they may be able to tell you, but I don’t think they feel obliged to go into all your history when you are there to see them for a special purpose. It’s just renal or whatever it is.

**Q: Now some of you guys, I think John touched on this, some of you guys see, see someone privately, either for your kidney or diabetes and then see someone, and then see the public clinic either for the diabetes or kidney disease, some of you do both, see both. Do you, if you compare the private versus the public care, what’s the difference?**

Lorraine: That’s what I was about to say, with the private care, I mean you go there, like, like for me to, when I went to, my diabetes, when I was first diagnosed with diabetes and the first thing the doctor, when I, they sent me to Hospital 4a and I saw a doctor at Hospital 4a, and he said to me to get onto to the, he put me on a tablet and I was working at that time, I was working as a machine operator, I was getting off course and I thought, my husband “it’s your body getting used to the tablet, you’ve got to eat and you’ve got to do this.” But then I went and saw this doctor privately and he said “your diabetes is not that bad, it’s, it is diabetes but it’s not real, why don’t you stop the tablet and start some exercise? Keep the tablet in case it goes very high, but start some exercise.” So education-wise I didn’t know that, if this doctor didn’t tell me that, and I started doing the exercise, I started, I mean I know a lot of, now can’t do it at this, but I was quite young when I was diagnosed, I was 30 years, like almost 35 something, I could have done the exercises. He didn’t educate me, “just take the tablet”, that was it. So when you pay money and you go and see the doctor he educated me in that line and I’m still, I’m still, I’m on dialysis now, and I’m still on diet control.

**Q: That’s excellent.**

Lorraine: Because I have learned how to eat my food, I’ve learned how to, my, my carbs, I, he showed me where to go and get books, which I didn’t know because I don’t have medical knowledge. I only studied up to Grade 8 in school and I learned all that, so now I know, I mean potatoes and all this carbohydrates, you know, after a certain time you don’t, I mean you have to eat them too, but…

**Q: Yeah.**

Lorraine: You also have to exercise with the carbohydrate, because otherwise it turns into sugar. A lot of people, if you don’t pay the money, go private, the hospitals don’t, which is wrong because some people don’t have the money to go private.

**Q: Yeah.**

Lorraine: Pensioners can’t afford to go private.

**Q: Yeah, thanks for that Lorraine.**

Marg: I really, Lorraine, it’s education is very, very important because my mother and her whole family had diabetes. Now I’ve got it and a brother and a sister have got it, and now my children, two of them, one has already been diagnosed and he’s 40, no he’s 54 and my youngest daughter is 43. She’s on the borderline and he is now trying with diet to keep the diabetes away. But I don’t think that his doctor is dealing with it correctly, because I think he should be going to a specialist and getting all the information that he, you know, can have some sort of educator that he can keep regular contact with.

**Q: So, so Lorraine, going back to the public versus private, Lorraine mentioned one thing she found when she went privately she got more education, those people who, who have experienced public and private, what other difference have you noticed?**

John: Oh, I don’t think some of these things can be that sweeping, like there’s a group in this hospital where, Penny Paton runs it, where they do have very good education services. They get somebody from every aspect of the dialysis, or kidney failure and they give a little talk.

**Q: Sure, sure.**

John: For half a day.

**Q: Sure, besides education, I guess, I think Lorraine’s sort of talking more about education at the cons-, at the consultant’s, consulting level…**

Lorraine: Consulting doctors.

**Q: I think you are talking about a broad education with the peritoneal dialysis.**

John: No, it’s very specific, very targeted.

**Q: Yeah, yeah.**

John: They have short sessions going through. They have somebody from that department give the talk, and then they ask questions from, and they have previous patients coming along.

**Q: Yes.**

John: So it’s very targeted.

**Q: That’s for peritoneal dialysis, isn’t it? Is that with the education for dialysis or..?**

John: No, it’s for…

Lorraine: No, that’s for seminars.

**Q: Seminars?**

Lorraine: That is for people that are going through, even I, before, when…

**Q: Before going through dialysis?**

Lorraine: You go, dialysis, or even now, because if you are on the trans-, you can go for seminars.

John: No, it, it’s not only dialysis it’s, it’s the whole works.

Lorraine: Whole, yes.

John: And she runs it, as far as that’s, she’s called an education officer. (32:32)

**Q: Yes, so this is for the kidney department is it, or..?**

John: Well, they are located next to the PD.

**Q: Okay, so I think, yes, they could be…**

John: Just, just, just here, yeah.

**Q: Yeah, yeah, yeah, so does anyone notice any differences between the public care versus the private care that they get? Like when they are seeing a doctor? Like in terms of clinical consultations, is there any difference between private versus public that you guys have experienced?**

John: Well the main thing is the private people have more time. The hospital system is gradually getting crowded in that there’s just so many patients to treat with so few staff. (Lorraine nods)

Lorraine: Yeah.

NItz: You notice that with the specialists, they, they are more, of course they are more thorough on you, you know? They go further and like, when you look at the diabetic doctor in a private, they don’t just look at your blood sugar level or how it goes, but he look at your eye, he’ll make sure all the organs in the body that are affected, he refers you to the eyes, to like to know the result of your eyes every now and then. (Lorraine mm nods) He would like to see your, your foot. He would like to know about the, running with this and that, he always up to, into that. If your sugar is coming high, he’ll always make sure he would see you in about the next two, three weeks to make sure it is, sometimes he’ll even give you a call on how you go with this.(Lorraine nods) I got a bit blinded by now I don’t have that private doctor, because I used to have a public one, which he just kept going on and on and on, my eyes is already infected, infested of cataracts and he doesn’t even think about it, know about it. He just want me to refer to the doctor that he knows. Well this one, when I go and transfer to different doctors, he looked at it I was nearly blinded for all I know my eyes is that bad. My foot is that bad as I can hardly even move my, my toes. So he, he…

John: Do you go to your GP at all?

Nitz: I, I get the GP too. What can he do to you?

Nitz: Your GP’s…

John: He…

Nitz: I changed my GP because one they give me before, I go to this person and he just kept on, he, I asked him once “can you give me diabetic education?” He said “oh, you don’t have to go to diabetic educator, just listen to me.” Just keep on stuffing you with tablets and tablets and tablets. I mean, every physician I would see - it shows a doctor from the doctor doctor, just want to give the consultation and stuff you with this, without even fixing or looking at the background. You get some doctors who are very terrible. Unfortunately, some of them that’s move interstate or somewhere, so you have to look for another one again, so that’s what I found, when my diabetic goes like that. I, I, forget myself to look after myself, because I used to be very busy working. I worked seven times, seven days a week, night shift, and I didn’t listen to my health well. I, I just take the medication that he gives me all the time.

John: What I think’s happened is that diabetes and kidney disease has just grown and grown.

**Q:** Mm.

Nitz: It has.

John: And it’s left the doctors behind, because most GPs and even specialists in this hospital are unaware of diabetes and dialysis, the extent of it.

**Q**: Mm, mm.

John: The treatment of it, because often when you come here, I go to about six specialists within the hospital because of the bits and pieces, and I suppose most of us are like that, and the, the nurses, the doctors in the other wards are relatively ignorant of what happens over this end. It, it’s not uppermost in their minds.

**Q: So are you saying lack of communication, or just ignorance?**

John: No, lack of knowledge, their own education.

Marg: Yeah.

Lorraine: Lack of, yes.

Marg: Lack of knowledge.

Nitz: It could have been avoided. All this, too much organs failures and everything if they, in the very beginning before diabetic was being look, nowadays it’s not bad you get a lot of diabetic educators now that the doctor push you into it and you get all this dietician, you get all this things for diabetic care that the government has introduced.

John: Yes, but still not outside the immediate field.

Nitz: Oh yeah.

John: Like I’ve been in respite care and you get on your dialysis of an evening and you’d have half the nurses in the hospital be coming across because they hadn’t seen it, they hadn’t done it in, there are only a few hospitals that do the whole lot and the, the nurses don’t get the grounding in these two, so even here, they are relatively unaware of it, and that’s why they have the PD nurses coming out to the different wards.

**Q: So besides what Nitz has mentioned, has anyone noticed any other differences between private care versus public care?**

John: The cost is massive.

Lorraine Yes.

John: And there’s no con-…

Lorraine: That’s what I said, some people can’t afford to go.

John: There’s no control on the costs of, one specialist might be $70, this one’s 120 or 200.

**Q: How about quality of care? If you see them privately, do they, do you think they tend to look at your more holistically than if you, if you went publicly? Is that a..?**

John: Some of them do.

**Q: Some of them do?**

John: Yeah, again, it’s, they get locked into a specialty and that’s the only thing. I think in the field of diabetes and dialysis it’s passed them by. As they’ve grown up and they are training, they’ve not been treating many patients, or any patients, in this areas, so they really need some educational, I don’t know what you call it, but there is a thing where people give you courses.

**Q: Marg, what do you think? You receive both public care and private care. I think private for your diabetes, is that right, or public..?**

Marg: Yes, I think privately is much better because…

**Q: In what sense?**

Marg: They seem to be more involved with what is wrong with you, and the questions and the time they take to check your feet and your eyes. (nitz nods) I sort of take the report from each doctor to the renal specialist and then, because he can then, if I’ve got some prescription that doesn’t suit my ailments, then he can take all, take this. I don’t know, I just, I just, where I live I have to travel. I go to eye specialist, I go to the heart specialist, because I’ve got a pace-maker as well.

**Q: Yes.**

Marg: So I’ve got a heart specialist and I’ve got the diabetes specialist, and of course the renal specialist comes to the clinic.

**Q: So would it be fair to say that most people think that if they go private, they might get better quality. I don’t know whether it’s a fair statement, but they might get better quality care and they might at least get someone who is more willing to look at them as a whole person versus just a single part of their body, would that be a..?**

John: Definitely.

Marg: Yeah, actually the GP just looks at you and…

Nitz: Yeah.

Marg: You just take this tablet. He really does not know what he is doing, that’s for sure.

(everyone agrees except Joe)

Lorrinae: Especially…

Marg: My GP, I only just go to her now to get a prescription, that’s all. I don’t tell her anything else. I keep the other ailments to the specialists.

Lorraine: Some GPs are also worried when you say you are on dialysis, because they haven’t learned much of, of kidney problems. So like even to give you an antibiotic, my GP, something “oh, I wonder if I’m doing the right thing?” Because he’s concerned about the, so he says “oh, can you make an appointment and see your specialist?”

**Q: So what are other weaknesses and frustrating aspects of the diabetes and chronic kidney disease services? Has anyone else got any other weaknesses they want to bring up? I think things that we’ve mentioned are waiting times, the lack of holistic care (Marg nods) that we’ve mentioned, what else have we mentioned? Sometimes we get, we see different doctors and we get differing opinions (Lorraine nods). Any other weaknesses people want to mention?**

Nitz: Well, I think as much as doing something. The thing is, they should give more subsidised, if you go see a private, like, specialist. (Lorraine nods) Like me, I’m under pension at the moment, if I have to see a medical doctor, I have to see an eye specialist, it’s not, it’s not only 70, it’s about 100 now. When you see your eye specialist again, because you haven’t seen him for, for about a year or two, he will start charging you, the first look alone it’s about, not less than 250, $270. I mean how much, you know? Between the dentist if you want to go see a dentist, if you get – everything, it adds up.

**Q: Okay, yeah. If you go privately that is?**

Lorraine: If you go, and even psychologists.

Nitz: Yeah.

Lorraine: Psychologist is not in the, like you, when you go, when you start dialysis, when I started dialysis I was quite a, I was quite scared and I needed someone to talk to and when I was in the hospital, when they put, when they started me they put, they had to put a *0:42:02.2 to start me off, because I had to go on dialy-, then the hospital they have someone to talk to me, but at home, there was no one, and then I needed home help because I’ve also got a back injury, and my husband live at home. So they said to “go to your council.” So when I went, my kids are married and when I went to the council they said “you’ve got to pay $40”, that’s fine, I mean because he’s still working I didn’t have a problem with that, I paid the $40 and I got, but some people can’t afford $20, or even $10. How are these people going to..? I was just thinking, when you, when you are down, at that time you need someone to talk to.

Margaret: Oh yeah, yeah. (nods)

Lorraine: You don’t have home help, what is the next thing that people think? They say about suicide and all that, I mean if I didn’t have, I would have, because I have always been a person who it’s my fault. I was always a person who was very, I had to keep my house clean. I have been brought up by my father like that, and everything had to be done, couldn’t keep, so I had to teach myself, and I went and saw psy-, a psychiatrist on my own money to get myself so that “oh, don’t worry about that, your life is more important, take a rest.” But those kind of things they need to have somebody to talk to these people who…

Nitz:: Government should have a thing.

Lorraine: Somebody.

NItz: Unless a person had, is more, you know, assistance if they need it. Diabetic alone cost a bit of money.

Lorraine: Yes, yes.

Nitz: Then they tell you to go to a, plus, if you’ve got a problem with your heart as well. So how many people do you have to see? How many specialists do you have to see? Plus the support to go with it, dialysis, you need psychologist at the same time, you need some home help, you need some, all this. You can’t move all that without nothing, even if everything is cheaply done. Especially with us who doesn’t have any relatives here in this country, you have nowhere to turn around so much, because you are embarrassed to ask your friend to do things for you, you know? After all, we’ve been working for what, 28 years of our lives? Like *0:44:18.9 know that we are like this. Sometimes I come to my stage at the time, why did, what did I do myself before? I give up everything and this and that to come here, I worked like, my guts out, it’s just unfortunate that I got sick suddenly and I got disabled like this. This is not my plan, I planned to work until I was about 65, unfortunately it just, got just like that. So, you know, being, you are a person who has been working so hard and you are so independent for a long time, you so proud to go there and people begging like that, it’s not you, so they should think about that as well, what we need, you know? They got social worker, you should help a bit, but you can only ask as much I think assistance as they can give you.

**Q: Let’s, sorry to shift gear a bit, let’s think, let’s think again about the public services that you guys attend, the diabetes and chronic kidney disease, what aspects of these services do you find frustrating?**

John: Availability, lack of days and hours, like PD it’s only two, you know, an odd hour in the afternoon and not at weekends. Unfortunately, most of your problems occur at night, or at the weekends, even with your PD, if something goes wrong you need to ring Florida or somewhere like that. It’s free, no problem there, but you are talking to someone who doesn’t even know what language you speak, do you speak English over there? So the availability of hours. I think that when patients come in here for a long time, they are treated like a piece of the furniture. People don’t seem to realise the needs of somebody that’s, if you were locked in this room day after day after day, month after month, it has a psychological effect on you. You mentioned psychiatrists, I went to one, or was sent to one, and the charge for just an hour was $500.

Lorraine: Yeah. (nods)

John: You know, how many times can you go to somebody if you might need it every day, or every week? There ought to be more dialysis PDs and haemodialysis sites around the state and around the hospital. I think…

Lorriane: The psychiatrist I think in the hospital here, even in the dialysis unit, someone to come and talk, some hospitals.

John: Some hospital only have like…

Lorraine: Some hospitals, the one that I’m doing, they don’t have any.

John: They only have haemo- and not PD, or none at all. So if you go to there, because of all the other things you’ve got, and in the long run I was bleeding last year for about, a number of months, and they couldn’t find what was wrong. It ended up the people at St Vincent’s found out where it was because they could get an extended, extended thing that could go up and find where it was in there.

**Q: Sure, yeah.**

John: The facilities aren’t good enough, hours, actual people and machines.

**Q: Okay, any other frustrating aspects people want to raise? Joe, anything you want to..? You haven’t said much.**

Joe: No, I agree with that. I’ve got appointments that differ in length of time and periods when I see the specialist, so I know that if I have a two o’clock appointment and don’t get seen until four o’clock…

**Q: The waiting, so the waiting times really bug you?**

Joe: One can understand…

John: We don’t have the time, so…

Joe: Understand, because there’s that many people in hospital as opposed to going to a surgery, or GP, they can evaluate their patients in a different way than in a hospital. So sometimes one just has to accept it, that’s the nature of the business, you are treating somebody and then you’ve got an emergency and you have to go to that instead of the next patient.

**Q: Yeah, any other frustrating aspects you want to mention, Marg, Lorraine?**

Marg: The travel, going interstate is another problem.

Lorraine: Yes.

Marg: I don’t have the….. I can go to the Gold Coast and live for three or four months even if the Robina Hospital can fit me in for dialysis. It’s the luck of the draw more or less.

**Q**: Yeah.

Marg: If you want dialysis there, so something has got to be done, Australia-wide, to move about.

**Q: Yeah, yeah. Okay, what about just focusing on the current, those health service at Hospital 4, the public ones…**

Marg: Yeah.

**Q: Any other frustrating aspects?**

Nitz: That’s all there is for me, and the parking, the cost of parking costs a fortune <laughs>.

Lorraine: The parking, yes, of course, yes.

NItz: If you have to come back here three times a week, I tell you what, it drives you up the wall. Sometimes you waiting here for well over two, three hours, that’s all. (laughs, and animated)

**Q: Does everyone agree about parking?**

Lorraine: Parking.

NItz: Well, it costs you…

John: It cost me $800 just to, my wife coming in while I was sick, you know, this is over the month. If you have to come several times a day, or keep on coming every day, it does mount up.

**Q: Yeah.**

Nitz: Otherwise if I have to come here every day, my God, you won’t believe, I hardly eat after that <laughs>.

**Q: Okay, well we’ve mentioned a couple of weaknesses and frustrating factors, what do you guys think can be done to improve on these frustrating and weaknesses?**

John: How many hours you got?

<Laughs>.Lorraine and Marg and Nitz

Lorraine: Even the chronic…

*0:50:24.2 <laughs>.

John: No, I, I thought this was serious so…

**Q: No, we are serious, so…**

John: Yeah, no.

Marg: Lack of coordination I think, you know?

Lorraine: But, sorry.

Marg: Taking all into account, the frustrations, somebody’s got to…

Nitz: What about a wheelchair?

Marg: Somebody’s got to be at the doctor and, what?

**Q: Well that’s one of the reasons we are holding sessions like this, so who wants to start first? What can we do?**

Lorraine: What about if they're for people, like chronic kidney disease people and people who always have to come to hospital for check-ups like this, giving them a pass where they pay a minimum on the car park, like a $2 card that, not to misuse of course, but maybe $2 or $3 something like that, if that can be arranged?

Nitz: We are lucky to get $9 off that sometimes, but the thing is we asked them if they can just limit the waiting period for so long. Why we only have to see the renal failure *0:51:22.2 they open only every Monday, right? And you have to wait for about three months, or two months for the next appointment and when you arrive there you waiting for about two hours, three hours. Why can’t they not cut it into half, like see some people on Wednesday or something, see some people on Monday? The waiting period will just be about an hour, hour and a half. You have to admit you are all talking about sick people here. If you are sick, I don’t think you can wait so long sometimes, if you are not feeling very well you waited there for about two hours and a half…

Q: Mm.

Nitz: You already hung it up, this time, that time….. at that time I get hypo, I don’t understand why, I get confused with something, I get nauseous, that like when he was doing a consultation with me, the sandwich passed through, I don’t know sandwich I just grab it and ate it and the orange juice, because I can’t reach the canteen any more. This is not bullshit, this is true, for that waiting. If they can go by half, why put in one go the same thing and then let the, you are talking about patients here who are all sick. They are not these people who are just asking for consultation, you’ve got a headache, or – these people have got renal failure and got diabetes at the same time, and sometimes you’re sick, you are nauseous, and sometimes their body is not used to dialysing, like me in the beginning. I felt so bloated, you know, like – you hang around there, you don’t know what to do any more sometimes. People are so tired, you getting like want to pass out, and then you’ve waiting for two hours and a half, then you have to drive all the way home in a very traffic hour, it’s too much. Cut it into half, both an hour and a half would be, I don’t mind an hour and a half, would be nice, not just over two hours all the time, two hours and a half.

**Q: What does everyone else think about any other ways we can..?**

John: Well as I said, I thought this was serious so I sat down and…

**Q: Yeah, no, no, we are.**

John: Looked at the things and I knew I was unusual because I have a lot of thing wrong and I was here for a long time, whereas normally you are in and out. I had a problem that I was bleeding, so everything was leaching out, so I just, bring me in, I got sick, come in, and that raises another point. The delays in Emergency are incredible for somebody that’s really sick. You are not going to die there probably, if you are lucky. It is too long for kidney and diabetes patients. If you are sick you’ve got to come through the system, now they treat you at the triage, but then they’ve got to get somebody, the PD department isn’t on in most of the hours, so you’ve got to get the sister from the ward, and that’s not easy and often not convenient, so that’s an aside, if there’s big delay then something out to be done.

**Q: Yeah.**

John: More than the government’s doing now.

**Q: Okay.**

John: But when you come in, you are just alone and then you just sit there. There’s religious services, but often nobody contacts you. I’ve been for weeks and nobody, and I want somebody from religious services to come, but it’s only every so often that a priest or a lay worker comes. There doesn’t seem to be any coordination amongst everybody. Here you are in a bed for weeks and months, a trolley doesn’t come around with the little things that you need, toothpaste and things like that, they ought to be a trolley service like a number of, some of the other hospitals have where, it comes around once a week or something like that with chewing gum and chocolates, anything you might want to get, toothpaste, letter-writing gear and all that. But anyway, I don’t want to bore everybody, but I’ve got a stack of things here. The only thing I can suggest is I photocopy it and give it to you rather than go through.

**Q: Okay.**

John: Unless you some of them to go through?

**Q: I mean maybe just raise the salient points very quickly?**

John: Yes, well that’s, what I’ve done is, I just put the questions that you put in the original letter.

**Q: Yeah.**

John: We mentioned the need to, take the treatment of the patient as a whole. We need more public education, so that the community and the medical people in the community are aware of these diseases if they are specialists. I’d say the same thing for the heart or something like that.

Q: Mm.

John: There needs to be more consistency amongst staff, they give you conflicting advice, or different advice. Some of them want to change your medication, some of them oughtn’t to be here, like in the clinic, you come along and there’s one nurse that takes you aside and tells you all the homeopathic things you ought to do or have, or where you go to buy sandals, things like that, when she’s got you trapped in a room when she’s doing just your blood test and things like that. More funding and more staffing has to be said, because that’s the basic problems in all hospitals. We need to make sure that there’s more awareness of kidney disease and dialysis and diabetes amongst the other wards, because, and that raised another thing, the number of bed changes. You might have five from the Emergency until you eventually get a bed ward. Now the system is being fiddled, they’ve got to get you out, so you only move one bed around, or two beds…

**Q: yes.**

John: Something needs to be done about that, and knowing that you are sick, it really knocks you around. Even simple things, when you’ve got those two things, the kidney and the diabetes, things can be difficult to do. So the design of tablets and bottles, the things to press through, if you’ve got nerve damage it’s very hard to do that, so a redesign of the tablets and the bottles, the lids, the actual dialysis machines, the connections to the bag, your fingers slip off and you rip your nails off. They need just a mechanical design, a designer would do in half an hour.

**Q: Yeah, yeah, okay, yeah.**

John: The problem of all your other conditions comes up that there ought to be some mechanism that’s stamped on the top “he’s got this, that and the other thing, and these are the things you have to watch out for.”

**Q: Have you got much more for this specific question on how we can improve, what we can do about some of the weaknesses?**

John: Well I was trying to get rid of all of them in the one.

**Q: No, that’s okay. We’ve got other questions as well so…**

John: Yes, yes, I understand that.

**Q: So, has anyone else got anything they want to add in terms of what we can do to improve on some of the weaknesses that we were talking about? Does anyone else want to add anything, Joe, Marg, Lorraine?** No ? **If not I’ll move on, does anyone else want to..?**

Joe: No.

**Q: What are some of the strengths, we’ve talked about the weaknesses, what are some of the strengths?**

John: Well the strengths are the people that you’ve got in this hospital, and all the hospitals are probably the same, they are fantastic. They are caring and concerned, they go mostly beyond limits, they do extra things for you.

Q: Mm.

John: But the system tends to constrain that, that there aren’t enough hours, the continual cycling, cycling of the groups, you know, you don’t have the same nurse for more than a day <phone rings>.

**Q: Sorry everyone.**

John: Alright, take it.

**Q: No, it’s okay.**

John: So you are explaining your conditions and all the things, with our diseases like we’ve got here, we end up telling the nurses mainly what the doses, what the things are, and even then, that’s great and everybody’s happy, the system then gets that you’ve got tablets that you need to have with a meal. So the breakfast or whatever it is comes now and half an hour or an hour later the tablets come, and so if you wait your breakfast is cold…

Lorraine: Mm.

John: Things like that, and that’s more funding, more staffing.

**Q: Yeah, so any other strengths anyone else want to mention?**

Loraaine: Yeah, I agree with that too. When I was in hospital I had that problem.

John: Yes, well I…

Lorraine: I had to have my medication with food, my phophsate binder , but it never came with, I had to wait for them because the medicine cupboard’s locked. (Nitz agrees)

**Q: Any other strengths people want to mention about the, the public system for diabetes and chronic kidney disease?**

Marg: Well, I’ve got very good treatment here and there, the nurses are fantastic, they’ve got time to listen to you and, you know, there are others who come and help if you are in strife. No, that unit is pretty good to me.

Nitz: So far I’m happy with the PD section, the nurses, they are more willing and helpful all the time. To me, that’s all there is.

**Q: MM: Joe, any strengths you want to mention?**

Joe: No, I’m happy with the treatment I get.

**Q: Okay, what do you guys find effective about it, about the public system in terms of the diabetes and chronic kidney disease?**

John: It keeps you alive.

Nitz: Mm.

Marg: I, I can’t say anything about the hospital system because I haven’t been in hospital very much. The last time was about four years ago.

**Q: No, we are more talking the out-patient system, clinics, dialysis…**

Marg: Yeah, so far I’ve been alright thanks.

**Q: Okay.**

Nitz: Yeah.

Lorraine: Yeah, they are very nice at the dialysis, yeah, all the nurses are well-trained and they help you out and everything.

**Q: And Nitz?**

Nitz: We, we, I noticed when I get hospitalised before in the section, in the dialysis, they don’t have enough beds <laughs>. How many times I was eating because I was checking out. I was eating outside of the room because some people has to come in. I felt sorry for them, but that’s what I noticed that the, you know, the, the waiting for more beds at one stage, they have to rush the man next to me in the hotel upstairs because the nurse just said “we don’t have room for you, because this one come in.” I don’t feel that very nice, somebody wake me up at three o’clock in the morning, like that, tell me like that <laughs>, you know? Like, you are not well, you know, because of the rushing all the time I could understand they needed a bed, that’s what I’ve noticed. There’s no bed, even just one out, one up one go, one up one go, so…

John: That does raise, that hotel is a strength for this hospital, that…

**Q: Oh, the MediHotel?**

John: Yeah, that you are available to have things done to you and you are not clogging up the system, that you can go somewhere without having to go home, or out to the country, or wherever it is.

**Q: Yeah, any other strengths or effective things that people find about the public system here with the diabetes or chronic kidney disease? Anyone else got anything further they want to add? No?**

Lorraine: I’m alright.

Marg: The diabetic side I don’t know very well.

**Q: Okay. What things, okay, what impact has attending the diabetes and renal health services here, made on your health?**

Nitz: A lot. I used to be petrified. I don’t want to know about it.

**Q:** Mm.

NItz: They’re very consistent to hammering it in my head that I needed this, I needed this, I needed this, because I’m too petrified. Just mention the doctor before, that he will have to do dialysis, I drop, you wouldn’t finish the conversation, because I broke down, I am that petrified.(Lorraine nods.. Hmmm) I think you need more staff to really go one on one sometimes, for those people who find it very hard, to slowly discuss it and break it into pieces. Yeah, the impact of that education at the first, before you go for dialysis and education that they give you about the transplant and all the stuff, it’s quite helpful. But I think sometimes for those people who find it very difficult to take, yeah, they get social workers to attend you, they get all these things, but I think personal one on one. Because some doctors sometimes say different, the way they deliver it to you, like, one doctor said “that’s it, you’ve got to have dialysis, if you don’t want it, you’re just gonna die.” One person says “how do you know you are going to cope with the needle? There’s no needle in it.” So your mind is like, if they did more education, just for some people. Some people who find it, into a denial state for a long, long time, but you need something, some particular person to break it up professionally, talk to her and to make her fully understand, that will be easier, because for me it’s a bit of a shock to my system. I just willing to die before instead of going this, because just the look of it. I had a friend who’s there, I seen her on haemodialysis and even then, even if they asked me to go there and observe him and see him what he is doing and everything is so happy and all this, this,…. I can’t take it at the time. For how many months I’ve been breaking up, breaking up, breaking up.

Marg: Yes, I think that’s very important that education just before you start dialysing. (Nitz and Lorraine nods)

Nitz: Mm.

Marg: Because I just, my doctor told me that, I had Dr Perry at that time, and he just told me…

**Q: Are you guys getting an education? Are you guys happy with the education that you were getting though before dialysis?**

Lorraine: Mm.

Nitz: Mm.

**Q: Joe, is everyone happy?**

John: That should be repeated every couple of years.

Q: Mm.

Lorraine: Yeah, what she said was too, like…

John: All the developments.

**Q: So everyone’s happy with the education they get on dialysis?**

Lorraine: The education is good, but, but, before dialysis.

**Q: Yes, before dialysis?**

Marg: Yeah, before, yeah.

Loraine: But when the doctor tell you that you have to go on dialysis, you still don’t accept it. You know, some people don’t hear it, like I didn’t accept it too, I thought it’s going to be another…

Nitz: Tell me about it <laughs>.

Lorraine: While before he’s going to tell me. You are going to put it off your mind. At that time, it’s true, when the doctor says it sometimes it’s…

**Q: Yes.**

Lorraine: So you need to have somebody there.

Nitz: If not that nurse (laugh).

Marg: Yeah, I think that have a sort of a class, you know, where they can bring you in and tell you how they will dialyse, what they are going to do. I was just picked up and taken to the Caulfield Hospital, and…

**Q: But there is a class here though isn’t there? They do do a class.**

NItz: Yes, it does have a class.

**Q: There is a class here?**

Marg: They don’t have one in Frankston.

**Q: Oh you don’t?**

Lorraine: I’ve been to the class.

John: There’s a lady that runs it.

**Q: Yeah, I think there’s a class here at Hospital 4, isn’t there?**

Joe: Yes, there is.

NItz: There is a class. This is a class discussion, what I’m talking is something for those people who find it very difficult to take.

**Q: Yeah, who are scared, yes. They need more one on one time.**

Nitz: They should make a bit of extra effort to make them understand, and to break it slowly, not just…

**Q: Okay, yes, okay, okay, so, I understand. If we can go back to the question, sorry, apologies, if we can go back to the question - so what impact has attending the public service here in terms of diabetes and chronic kidney disease made on your health? Joe you haven’t said much.**

Joe: I haven’t had any problems with that.

**Q: So what impact? Has it been good impact, bad impact, improved your health?**

Joe: Good, yeah. Ninety nine percent, it’s good.

John: I would have been dead years ago.

**Q: Okay, John yeah. And Marg or Lorraine?**

Marg: I’ve no problems with the treatment.

**Q: So has it been a good impact, or..?**

Marg: Yeah, I feeling in myself, I feel much better because I got into it at a very bad time. My husband was ill and he was on the last stages and my kidneys were going, and everything was happening together so the family were, oh, it was just one big mess at that time. But now I have settled down and it’s much better.

**Q: Okay, let’s…**

NItz: Excuse me…

**Q: Yes?**

NItz: Do you do, what sort of peritoneal do you do at home? Overnight or the bags?

John: Both.

NItz: Both?

John: Both, manual bag and automatic machine.

Nitz: Mm, you don’t have, you only use one bag during the day, or..? How many bags do you use during the day?

John: One on the manual and that’s two litres, one 6 litre and one 2.5 litre on the machine.

NItz: Because I’ve been, I’ve been asking for a machine and they said “yeah, we’ll put you into a waiting list”, and I meet some people I go with…

**Q: Sorry, can we, we can talk about this later, sorry, just for the interests of time.**

Nitz: Alright.

**Q: We need to keep on moving, I apologise, but you are more than free to continue this conversation, you can ask me questions if you want after. Now let’s change tack a bit and think about the experience, all your experiences that you’ve had living and looking after your diabetes and chronic kidney disease. So let’s just start thinking about all your experiences and I know for some of your guys it’s been quite a long time. In your opinion, what do you need to look after your diabetes and chronic kidney disease well? So what needs to be in place for you to look after your diabetes and chronic kidney disease well?**

John: More staff and more facilities.

Joe: And more specific, because if you generalise, two people are not the same in the same, example, if your diabetes is handled by tablets…

**Q: Yes.**

Joe: How do you not get onto insulin? What is that progression whether you like it or not, from tablets to insulin? (Lorraine nods) Now if you have that information given to you up front, you can span, how can I say? You can make arrangement in a way that it won’t impact you too much when you go onto insulin, because with insulin you have to take your meals at certain times, your dosage at a certain time and it is very frustrating when you can’t, people saying “come in here at three o’clock” and wait two hours to be seen and you know you have to get out your meal at five o’clock. (Lorraine nods)

**Q: Mm.**

Joe: To get that done you need to be out of here by at least one o’clock, so you have to try and span your arrangements in a way that you don’t impact yourself to the extent that’s detrimental to what you have. But that’s all a matter of what happens on the day, or at the time, and it’s not always foreseen. You can be an hour, you can be two hours, but that’s not something either you as a patient, or the doctor can foresee, because what, other interferences between the two.

**Q: Sorry, I’m not sure whether you guys understood the question, what I’m asking. I guess what I’m asking is, what do you need in place to look after your diabetes and chronic kidney disease? For example, some people said “we need support from family, we need support from the hospital, we need to be educated. We need to be motivated…”**

Lorraine: Yes, that’s what I was going to say, more education.(Marg: mmm Nods)

**Q: So that’s what I’m sort of asking. So what do you need in place to look after your diabetes and chronic kidney disease well?**

Lorraine: More education, even on TV, I mean they always show things about cancer on TV, nothing about diabetes. They show on newspaper obese and, but most of the people, some people are not even obese they got diabetes, just because, so just show it on, like if the government can do something like that, just show something, you know, where people can learn a bit more and more education, even in schools. Kids, kids nowadays eat more junk than…

**Q: Yeah, but, but what do you personally need in place to look after your diabetes and chronic kidney disease well?**

Nitz: It’s the help, care, looking after yourself. They should show, having diabetes and all the concerns about the effect of the diet and that’s what they come up with exercises. The thing is I would like to have someone who will show us how to improve as well out well-being (Lorraine nods), especially the circulation problem of how to handle that one, we don’t have that same topic being talked to us because seems to be that some people doesn’t know. I mean you get this, you get the numbness of your hands, your feet and everything. With the weather, hot weather comes, what’s the best aid for you to take, be able to surpass that, to be able to make yourself manageable. To be able to get all the stiffness, even if, you know, you can’t really exercise so much or walk so much when you’ve got diabetes, because it hurts in the long run. You get exhausted easily and you get tired easily, so something like that. Care of how to improve your body, improve your strength, to improve these things. To be able to handle, because that seems not too much of it.

**Q: What do other people think? What do you personally need to look after your diabetes and chronic kidney disease?**

Marg: I think it’s plan with yourself.

Q: With…

Marg: You’ve got to plan, in the morning you know you’ve got to do certain things. You’ve got work it out as to where your meals are going to come in. Now I’ve got to have a needle even at lunch-time…

**Q: So you need to be organised, is that what you are saying?**

Marg: Yes, and be in a position to take that injection at that lunch-time and see that you get your needle in at that time, even if you have to, I get my groceries tomorrow, or I’ll, you know, or the next time I come shopping, but you have to plan your day, because it’s very important to have the correct meals at the correct time and the correct injection at the correct time.

**Q: What do other people think? What do you personally need in place to look after your diabetes and chronic kidney disease well?**

Marg: Sorry, the other thing is, supermarkets having correct food. You’ve got to sit down and read every label and every bottle and every packet to make sure that they are having the correct food. Should be something simpler than that. (Lorraine smiles)

**Q: Yes you are laughing Lorrraine.**

Lorraine: And also educating yourself, educating yourself and making sure, like if you are going somewhere and you are not sure what time you are going to come home, having some jelly-beans in your handbag.

Marg: Yeah.

Lorraine: Having a sandwich or something in your handbag, so if you do feel that your sugar, that’s what happens to me, I don’t get high sugar I always get low sugar. So I quickly sit down and I take a jelly-bean, because, and then eat some, with the jelly-bean you eat a carbohydrate something as well, because that’s lifts it up straight away. So everybody being educated that this is not…

**Q: Yeah, so being educated, being organised…**

Lorraine: Educated, and you educate yourself as well.

**Q: And being organised, yeah.**

Lorraine: Yes, doctors letting, go to the doctor and the doctor he said “yeah, yeah, yeah, this, this, this.” But you listen to them and you read it yourself, because it’s your body.

**Q: Yeah.**

Lorraine: It’s your body that you’ve got to look after, and the doctor…

**Q: So being motivated you are saying?**

Lorraine: Motivated, you can’t expect a nurse or a doctor to be with you 24 hours a day, so you know, you quickly take it and motivate yourself.

**Q: Yeah, what does everyone else here, what do you think John, or Joe? What do you personally need in place to look after your diabetes and chronic kidney disease well?**

John: Well, I think one of the important things is that you need social workers more aware of the problem for diabetes and kidney disease. Some of the social workers here are, don’t know, like what facilities do the councils offer? And you’d think that they’d at least know in this local area, council’s, what, can you get somebody to help you clean your windows? Can you help somebody showering? And there doesn’t seem to be a coordinating body from the district nursing to the council, they all give bits and pieces. Nobody seems to know what it is, and it only takes one phone call by the social worker to ring the local councils, find out, get the girl, the district nursing girl, to tell that. That would relieve a lot of your problems and worries.

**Q: Yeah.**

John: Because there is lots of things you do need help with. Some are just minor, and some probably couldn’t ever be catered for, like you couldn’t have people running around doing things for you, but the social workers should be aware of that if they are any good.

**Q: Yeah, yeah, anyone else? What they personally need to look after, personally need to be in place to look after their diabetes or chronic kidney disease?**

Nitz: Well first of all you’ve got to positive in life.

Lorraine: Mm.

Nitz: I don’t take things seriously any more, like I used to worry so much, “yeah, okay.” Being diabetic you’ve got to have jelly-beans and have all this, your water, you’ve got all this crap in your bag, having a kidney, you’ve also got your kidney bags, you’ve got one dialysing bag. Everywhere you go, you get invited somewhere, I go, with my social life sometimes I go visit friends on the other side of town. For my, you know, being, if you don’t think positively all the time, if you think about the illness all the time, if you worry so much all the time it will drop you really down. For me, it’s being, thinking about the positive…

**Q: So having a positive attitude? (Marg and Lorraine agrees)**

Nitz: Oh yeah.

Lorraine: Mm.

Nitz: It helps a lot, not because...

**Q: So Marg and Lorraine are agreeing with you.**

Nitz: “Well I’m going shopping and have a bit of a coffee or something”, like that, a bit of a latte, talk to friends, you know? (Lorraine nods) It’s something that you keep on doing for yourself, you fit in your food, your life-, some of your lifestyle at the same time, but not to mention, don’t put yourself right down. Don’t worry so much…

Marg: Yeah.

Nitz: This and that, just go things at the time.

**Q: What does everyone else think? Joe, you haven’t said much.**

JOe: I’m quite happy with what I have got in hand for myself. I can’t talk about…

**Q: And what, what are those important things that you’ve got in hand which help you?**

Joe: Well I, I know the importance of not taking my medication on time, having meals at regular hours and that sort of thing. And so that’s a safeguard as far as I’m concerned, towards my welfare and that’s the main thing.

**Q: Yeah.**

Joe: Because you can look after yourself, but as I’ve been told, diabetes affects a lot of oneself, kidneys, sight, urine, everything else. So if you progressively look after the things you have to do, that is going to be a big advantage in controlling what you’ve got.

**Q: Yeah, so you are basically saying you’ve got everything in place yourself?**

Joe: Yeah.

**Q: And you are making sure it’s…**

Joe: I have to make sure that I’ve got them in place.

**Q: You have to make sure, you have to make sure, okay. Have you got anything further to add John before we move on?**

John: I was just thinking that, in terms of the patient, the hospital could become more patient-friendly in the design of the basic equipment. You go, one test I had was there’s a huge machine, X-ray machine the size of this room…

**Q: Yeah.**

John: And it sort of comes down on you. It’s the one looking at the radioactive blood, so it comes down and you are cramped with this monstrous machine over you and you get very claustrophobic, but there’s nobody there.

Nitz: Really?

John: So the two problems…

NItz: It does seem a bit…

John: Yeah, well, I, I called out and I called out.

Nitz: “Are we there yet?” <Laughs>.

John: The, you oughtn’t to have patients left alone with nobody to contact, or even a button to press and the machine itself should be adjusted, you are there for hours, so even just photographs on the ceiling, or up on the machine when it’s down on you, ought to be there.

**Q: Yeah. John, a lot of your answers have been focused on the services and the system, but what about any other factors, personally, that you think need to be in place for you to look after your diabetes and chronic kidney disease well?**

John: Well, I, I mentioned about Emergency. You ought to be able, if you come, you are going to get poisoned, so you, you, you contact the hospital immediately, but then you are going to spend hours in Emergency. There ought to be a direct line to the ward or something…

**Q: Sure, sure, sure.**

John: Where you can come in and be assessed straight away.

**Q: But what about, what do you need day to day to look after your diabetes and chronic kidney disease well? I know you were sort of talking about a lot of situations here and there.**

John: Well that’s what your days are, the situations you worry about, the next thing, or what could go wrong? Like the problems that can go wrong in dialysis in PD, you get a book of them. There are so many, there are hundreds, so they fill out your day.

**Q: So we’ve mentioned a couple of things which you need to look after your diabetes and chronic kidney disease well. Are all of these factors in place? So education, I don’t know…**

John: Availability of hours, they are not there.

**Q: Availability, they are not there. Are they all there, or are some lacking?**

Joe: I haven’t gone onto dialysis. I can’t answer that question.

Nitz: They are all there.

Lorraine: Yes.

**Q: John, it sounds like you are saying there are a lot of things that are lacking, is that right?**

John: Yes, yes. Whether they are important or just minor.

**Q: Marg, or Nitz:?**

Marg: There seem to be more problems in the public system than in the private, isn’t it?

Q: Mm.

Marg: But then not all of us can afford private, so…

Q: Mmm.

Marg: Maybe as Lorraine said, government can step in and help the others to get their…

**Q: Yeah, one of the main purposes of this is we are trying to improve the public, so…**

Marg: Yeah.

**Q: Okay. Now what, what things make it hard for you to look after your diabetes and chronic kidney disease well?**

Nitz: Well one thing is sometimes it’s, like for me, it varies subject to you feel like eating sometimes by yourself, you know? Like, I’m sort of person who’s not really a loner, that stays at home just by myself. I want to go out. I’m more happy to be with, surrounded and everything. Sometimes if you are doing kidney dialysis, peritoneal at home, four times a day, you don’t have much time for that. After you’ve finished washing your hands, doing your kidney and this and that, putting in your books and doing some house-keeping, going back again and do that sort of thing. Just about four hours with those. If you’ve got like an appointment, something like this, you have to try to fit yourself into that. The problem sometimes is, when I ask this, the lack in the hospital, the lack of, the time wasted waiting, you know? Things could be just better if we can spread our time, because if you are doing dialysis and at the same time doing insulin, it’s not easy to juggle both of them.

Q: Mm.

Nitz: You have to be set in your times sometimes. Sometimes I don’t think so much about it, I don’t think seriously, but you’ve got to be aware that you have a time to do insulin, you have a time to do dialysis, and a time to wait for this hospital and travelling time that you have to go from here to there. It’s that sort of thing that really annoys me, the long waiting.

**Q: So everything’s so time-consuming?**

Nitz: Yeah.

Marg Yeah, and it involves a lot of organising.

Lorraine: Mm.

Marg: Yourself and especially if you are alone.

Lorraine: It does, yeah.

Q: Yes.

Marg: You’ve got to see to so many things.

Lorraine: You need to.

Nitz: Living alone yourself if more than enough, let alone your house-keeping to go with that at the same time, you know? So many activities on the outside because you still have to see your GP, you still have to see your chemist, you still have to do your blood test, you have to do this, and you have to see the diabetic educator sometimes. You have to see your dietician, you have to, so much…

**Q: So just looking after your health takes a lot of..?**

NITz: Oh yeah, a lot of, yeah, yeah.

**Q: Very time-consuming?**

NItz: Yeah, yeah.

**Q: Too many appointments?**

NItz: Oh yes, you have to have a diary to fit in everything.

John: Well…

Joe: I do have a diary.

Lorraine: Me too.

Joe: It got so many.

John: I, I’ve had weeks with nine appointments in it, or days with three appointments in.

Nitz: Yeah, yeah.

John: And if they are not in the same location you’ve got to travel around T City.

Q: Yeah.

John: It’s, it’s a real problem the number of appointments and the ease of getting to them.

**Q: So what else makes it hard for you to look after your diabetes and chronic kidney disease? What factors act as barriers I guess to looking after your diabetes and chronic kidney disease?**

Lorraine: Lack of educa-, as usual lack of, you know…

**Q: Lack of? Lack of education?**

Lorraine: If you don’t know, if you don’t know what you are doing. If you don’t know.

**Q: Yeah. So some people have mentioned that, conflicting information, or lack of support, or having a lot of other illnesses, or being sick, does anyone identify with any of those?**

John: Well, one of the problems is your body’s slowly failing with these diseases and it makes it very hard to do lots of things.

Lorraine: Things, yeah.

John: What’s simple for some people, it becomes hard for you to turn on a tap, that can be difficult. I have trouble picking up things, the nerves have gone, so you don’t know where your fingers are, and if you can’t see your hands, you don’t know where they are, or your feet.

**Q: Yes. Any other factors? So someone mentioned conflicting information, does that make it hard for you, or..?**

Nitz: I think the council need to keep an eye on your health as well. Home help, even if they can just assign about two or three hours in a week or something to help us.

Lorraine: They do, they do, but you’ve got to pay for it.

Nitz: Well, oh you have got to pay for it, yeah, yeah.

Lorraine: That’s what I’m saying, that’s what I just said before.

NItz: And sometimes it depends whether you are entitled to it too.

Marg: I have one hour help every week from the council to clean, cleaning and, you know, doing the toilets and changing bed linen and all that stuff.

NItz: I’m not getting full disability yet, so if I think I get provided by the government to get full disability I can get some help, a lot, not at the moment, you know, just giving like, just giving like the, just getting your medication free that’s all I have, and I find it very difficult.

John: There is one area that there probably needs to do more work on, and that’s the person that cares for you. If you’ve got somebody caring for you, the carer…

Lorraine: Yeah.

John: Where do they find out what facilities are available from the council, or the government, or the hospital, the nursing service?

Marg: Yeah, even for the personal carer.

Lorraine: And the question to that, when I went to get home help, when I had my kidney out, they said “that wasn’t much one of kid-…” The first question they asked me was “have you got children?” “Yes, I’ve got three children, but they are married, they are working. They can’t come.”

Marg: They have a life.

Lorraine: Yeah. I said “they can’t come home to clean my house when they got…”

John: Got their own families to look after.

Lorraine: “Their own families.”

John: Yeah.

Lorraine: I mean not that they don’t come and do it, but you can’t expect them to give up work. Then they said “okay”, then I had to pay, I couldn’t afford to pay so my husband took three months, because I had a bit of, it was six weeks that they asked me not to drive, so I couldn’t drive to dialysis, but then I had complications, so they asked me not to drive another, so he had to take his long service leave. So they keep asking about your child, “why don’t your children help you?” But I mean your kids, they are married, they got their children, they, they bring their babies and come, it’s even worse sometimes, because there are children running up and down. They house is even in a worse mess, not that, I love to have my grandchildren, but I’m saying they are coming to clean a house, so there’s no…

Nitz: Yeah, I used to a long of things…

Lorraine: So this is…

NItz: But when you start moving, your body doesn’t follow <laughs> after fifteen minutes..(laughs).. that’s it.

Lorraine: Now I do, yeah. So now I do my own, like a try, one day I do this and the next day I don’t do everything at once.

Q: Yeah.

Lorraine: And on the weekends, my daughter, or my daughter, they, when they come to visit they will change the bed linen, and <laughs> so, because I’ve got a back injury as well, which I can’t…

Nitz: To have a bit of a home help, honestly, changing…

Marg: So you don’t have council help? You don’t get council..?

Lorraine: I have to pay. I have to pay for the hours, here but I’m not working.

Marg:: Yeah, I pay too, yeah.

Lorraine: So when I have to do it, my husband stay, it’s very hard $40. I know it’s $40 a week, but it adds up, it becomes hard.

Marg: No, but my, my council is only $6.

Lorraine: Yeah, but you must be on a pension. I’m not on a pension, so I get different rates.

NItz, Marg: Yeah, yeah.

Marg: Not on a pension, like us.

John: Yes.

NItz : Until I get 65, they wouldn’t give you that.

Lorraine: Pensioners’ rates are different to…

Marg: Oh.

**Q: So if I’m hearing you correctly, what, what you are saying is the actual disease itself makes it hard to look after…**

Marg: Yeah.

Lorraine: Oh yeah, yeah.

Nitz: Yeah.

**Q: Plus also perhaps a lack of finances and a lack of access to home help?**

John: That’s very important, yeah.

Q: Yeah.

Marg: Because you are even exhausted very quickly, and in my case I just do a little bit and then I sit down, and then I do a little bit more and I sit down.

Q: Yeah.

Marg: Because I get tired and I can’t. **1:31____________________________________**

Lorraine: Especially the days you come and you do dialysis, you can’t, all you want to do is sit down. You don’t, you’re drained out.

Marg: Yeah.

Loraine: You just can’t, and then you start getting cramps sometimes, taking too much fluid out of you.

Marg: Yes, awful.

Lorraine: So you can’t really do much.

Marg: No.

**Q: Any other factors or barriers that make it hard to look after your diabetes or chronic kidney disease well? Some people mentioned lowered mood.**

Lorraine: To look after it more what I do is on the weekends, me and my husband will cook for the whole week, because when I come off dialysis-, so we both get together and we cook and we freeze it.

John: Oh, that’s a good idea.

Lorraine: So when we, when I come after dialysis and when he come after work, food’s there, we don’t have to worry about food. And so those little things…

Marg: Yeah.

Lorraine: Like if you do on the weekend, even if it’s raining we still wash the clothes on the weekend, and we put it on the airer, and if there is sun I put it out. So we both do it together on the Saturday morning, so I don’t get much tired and he helps me out too, so which I can’t do vacuuming because of my back, so he does it, and with the bed linens sometimes we have arguments because I want it this way and he, so to stop the arguments my daughter-in-law will come.

Marg: You’ve got the advantage of having help.

Lorraine: :Yeah, so my daughter-in-law…

Marg: says something

Lorraine: Or daughter will come up, come home and they will do the bed linen, because he wants to put the, it’s men, men have a different way of doing this. So it’s just, it’s…

John: ?I would have got a fix up?.

Lorraine: So that’s how you got that, I have started doing that just to make it easy for me, from your question, to make it easy to look after my, my health.

John: That’s one area where having a get-together with people talking about how they are coping with all the problems, you know, say an annual conference or something, annual patient get-together might be good.

**Q: Helpful?**

John: In the education side.

**Q: Does everyone agree with that?**

Marg: Mm.

Lorraine: It still does get me down sometimes, because I come from Sri Lanka and I did a lot of catering, and I love to do that. I used to be, I think you are from Sri Lanka too, and I used to do catering at home, which I loved. I loved doing it, but I had to stop doing it because I can’t do it anymore. But now and again, I mean I can just go and make something, cook some rice and curry.

John: She goes out, she doesn’t cater at home.

Lorraine: I can go and do a little bit for myself, but like things like, there are hard things that, you know, what [string hoppers] is…

Marg: Yeah.

Lorraine: Which I do myself.

Margaret: I go and buy them <laughs>.

Lorraine: It’s a hard thing because you’ve got to squeeze it.

Marg: It’s a long process.

Lorraine: Yes, it’s a long process and those little things, when I felt down before, if I, if I have an argument, or if I felt a little bit down, my therapy was getting something out, baking something, cooking something, but I can’t do that now. I can’t do those squeezing and stuff like that.

Marg: It’s cheap enough to go and buy <laughs>.

Lorraine: To buy, but it was something that I have always said I will never do. I will make my own, but now, I mean there are places where you can go and buy them, not that you can’t buy, but it makes you feel really, like I’m 55 years old. So I’m thinking “I’m 55 and I should be able, I am old, I’m not saying I’m young, but my Mum, when she was 60 years old she was still making that.”

Nitz: : Yeah.

Lorraine: So it made you feel, makes you feel down, but then I’m very, I, I’m a Christian, I’m very involved in my church group things, so quickly I’ll go and call someone as soon as I get these funny thoughts in my head that I feel, “oh no, I want to dash everything. I want to…” Because I can’t do something and I settle down soon. So those are little things that I’ve educated myself to do, call someone, call a friend, call somebody from the church and talk, but there are times that I’ll go to the kitchen and only last month, after I had my second kidney taken out too, I took all the string hopper things that you make those things, because that made me feel a little bit, every time I look at it, so I put them all in a, in a box and it’s in the shed now.

Marg: Mm. (laughs>

Lorraine: We don’t want to just put it out where nobody knows how to use it, waiting for some person who comes from Sri Lanka who wants to use them, they can use them.

Marg: And not even your children want to learn how to do it anyhow, so…

Lorraine: No, my kids wouldn’t even know how to do it <laughs>.

Nitz: I learned from my doctor, she is the best Sri Lankan doctor. She moved to Queensland. Her advice to me at the beginning when I start not coping well with myself, especially, you know, the frustration of not even being able to do things that you used to be able to do. I used to be like that too, very fussy inside my house. We used to have a two-storey house, and I want it very nice and neat. I, I am very fussy with everything because I work. I get somebody to clean my house and I want my house to be always spotlessly clean. Bed linen should be changed at least once a week and I want make all this in a pair and fold this. The doctor told me “you have to learn how to accept that there’s certain things that you can’t move any more. If you take off the fussiness from your head, and just do what you can and be happy with what you have. Put yourself, transfer yourself into frustration or boredom into something that you enjoy the most. Because you like socialising”, so I opened Facebook.

Marg: <Laughs>.

Nitz: I try to talk to my nieces and my nephews all over the world. I post my, somebody to make me a Skype that I don’t know how to do it, but I want to open a Skype and off I go. That, that boredom, that loneliness that you have in yourself, talking to your nieces, to your nephews, all of them, and it’s nice that, they know that you are sick, they try to cheer you up and they tell you the good stories nowadays, so I think that brightens my day.

**Q: yes.**

Nitz: Every now and then I arrange with a girlfriend to see, we have a bit of a coffee or anything like that in the shopping centre. I used to have a friend, I’d get, ring some of the friends who would, I used to be like a psychiatrist who listened to everybody’s problem, but I come to the stage that I don’t want to do that anymore now. Just tell me the good thing and I’ll be with you, <Clem and Lorraine laughs> don’t tell me about your problem with your husband, because you know, you should sort that out yourself at home, and I just only go there and I find that…

Lorraine: That helps.

NItz: If you don’t have stress any more.

Q: Yes.

NItz: You don’t worry so much about stress, you don’t have any, thinking about something in your head all night about “oh, I should have done this one. I should have done…” I don’t have that thing any more, and I try to fix up my circulation by doing these exercise and get massage and everything. I’ve noticed that my body has started to start getting better, my face is getting brighter and I start to cope things in life, is here. That’s how I think you do it.

**Q: Yes.**

Nitz: Because if you, I used to, I used to socialise a lot before, I used to have barbecues, I used to have lunch with them, dinner with them, this and that, and I always invited friends because they liked my cooking of this and this. Even my son sometimes told me “we should have bought up a restaurant”, because I feel the whole classmates, the whole troops of friends, and I got this friend that goes through my house and he said, but I stop all that because I, I, I started to realise that my body can’t take it anymore.

**Q: So what you are trying to, what I’m starting to hear is that there is some issues with, I guess, adjusting to the loss of independent?**

Marg: Adjusting, yeah.

Nitz: Yes.

**Q: That comes with going into end stage kidney disease with the diabetes.**

Marg: Mmmm

Lorraine: Yeah.

John: Yeah.

Marg: And you’ve got to find out what will take your mind off the disease.

Nitz Mm.

Marg: And do some sort of relaxation.

Lorraine: That’s it.

Marg: I like cooking, knitting.

Lorraine: Yeah.

Marg: Crocheting, so…

**Q: So that prevents you from having a low mood, I guess?**

Lorraine: Yeah.

Marg: And if I’m really feeling bad I pick up the phone and talk to one of my children.

Loraine: That’s what I, yes.

Marg: So that helps a lot.

**Q: So does, does, if, if you weren’t able to get out of that, and you did have a low mood, would that affect, that would probably in turn affect your diabetes and chronic kidney disease?**

Marg: Mm.

Lorraine: Because your stress can cause it more.

Q: Yeah.

Nitz: If you are more stresses, you are more worried all the time, you know, your sugar level come up. You can never control it no matter who much good you are with dieting and with exercising.

**Q: Sorry Joe, I hope this hasn’t scared you all this?**

Joe: No, no.

**Q: Have you got anything to add? I know you haven’t said much.**

Joe: Not really.

**Q: Nothing to add?**

Joe: It’s all information.

**Q: All information.**

Lorraine: Dialysis doesn’t hurt at all, the ones they are doing, it doesn’t hurt, once the fistula is done.

Joe: Yeah, I’m having the peritoneal one done.

Lorraine: You are having the peritoneal, yeah, it doesn’t hurt, it just, when you come home sometimes you feel a bit tired, that’s…

**Q: Let’s…**

Nitz: I was so scared about the needle. It scares me, the needle.

**Q: Let’s move on to the last, last question…**

John: I just, those two things, they’ve brought up something that I’ve thought about before, is that the physiotherapists, you need the physiotherapy but they seem to have a lot more control over you than the normal doctor. I think the physios have too much final say, for example, when you are coming out of the hospital, they are the ones that really decide your release date. I think there should be some sort of course given to the physios here on diabetes and dialysis, kidney disease, so that they are aware that when they come and give you all these exercises, some of them will kill you, like your sugars can go down very rapidly with some exercises and you can go into hypo… (Lorraine: Mmmmm)

**Q: Shock, yes.**

John: And if there’s no trained nurse, which there isn’t usually in the physio rooms, you can die.

**Q: Yeah.**

John: The other thing is, it’s very hard accepting that you are losing your facilities, because you lose them more quickly than…

**Q: When you say facilities, you mean your ability to do things?**

John: Yes, yes, physical things.

**Q: Yes.**

John: And, and thinking as well. The ward, or the PD department, ought to have a psychiatrist that is aware of the problems you are having in this sort of environment so that you can come and talk to them, and they understand what you are talking about. Just, just even bump you up.

**Q: Well I <interruption>**

Stella: It’s been two hours now, I think you might have to go, because somebody needs to, your transport has a meeting soon.

Marg: Oh okay, yeah.

Stella: So we’ll probably have to finish it up, but I’ll just…

Lorraine: My son’s in hospital, so he’s gone for an operation and we have to pick him up at six o’clock.

**Q: Okay, look this will, this last one will only take ten more minutes. So now that you mentioned that this, this question is the last question we’ve got, it’s a bit more fun. So obviously you’ve commented on things that you’ve liked and disliked and things like that, imagine if you were the director of Southern Health, and had the power to change things, if we were going to design a perfect health service which would meet all your needs, what would that include?**

Nitz: I think we need about two hours for that story <laughs>. You want to know? I’ve got lots of things to say.

**Q: Well in ten minutes, we need to finish this in ten minutes.**

Lorraine: I think each chronic department not only diabetes and dialysis, but even people with chemo and all that, that they should have more doctors when they come to the clinics.

Q: Yes.

Lorraine: Like this gentleman can say, like, you know? Because sometimes you come and they’ve got a nine o’clock appointment, I have gone for a nine o’clock appointment, and there are ten people who’ve got nine o’clock appointment.

**Q: Sure, sure, yeah. Anything else? So we are talking about how could we improve the chronic, diabetes and chronic kidney disease services? We are redesigning the whole system, what will we include?**

Nitz: Educate the kids.

Loraine: Education.

NItz: Early.

**Q: So educate, prevention.**

Nitz: Educate the school.

Lorraine: Prevent.

**Q: Anything else?**

John: In that, you ought to bring the kids from school in and see the operation.

**Q: Okay, so education, prevention, anything else?**

Lorraine: The people itself getting more education, the person who is going through the sickness getting more…

**Q: Yeah, okay, anything else?**

NItz: More facilities.

Lorraine: Facilities.

Nitz: For, for the people.

Lorraine: And diabetes…

NItz: You can just get diabetic nurses and educators in one state, in one particular place and they have to book for this and that, they should have been roaming around for those people who can’t go out, especially for those disabled people who have got the difficulty of going out, that have appointments and this and that, they should have some people, nurses or anything to visit, home visits and push more awareness.

**Q: Okay, anything else? Some people…**

Lorraine: More dialysis centres.

**Q: Okay.**

Lorraine: Because I was, I started dialysis at Austin and from Narre Warren to Austin in a long way. I had to do it over a year until I got…

**Q: So more local care?**

Lorraine: More local care.

**Q: Anything else? Joe, nothing you want to add or improve?**

Joe: No, no, I’m alright.

**Q: John?**

John: Too many little bits and pieces, but you’ve covered the main one.

**Q: Some people mentioned, do you want to add anything Marg in terms of, if we were designing a new system for the, for the diabetes and chronic kidney disease, what would you include in it?**

Marg: I would say that more units closer to each other to help people, because travelling to and from dialysis is not, not a nice thing, because you are tired afterwards.

**Q: Okay, yeah.**

Marg: If you are waiting for transport, ambulances take a long time to come and pick you up and if you are driving yourself you have to make sure that you are capable of driving. Sometimes your blood pressure has gone down and…

**Q: So closer, I think closer?**

Marg: Yes.

**Q: So some people also mentioned cheaper parking, or some people even mentioned a combined renals/diabetes clinics, so that they can see everything in one go.**

Marg: Yeah.

**Q: What do every-, what do people think about those things?**

Lorraine: Yes.

Nitz: That’s a good idea.

Lorraine: Yes, that’s very good, yeah.

**Q: Which one? The…**

Lorraine:The combined.

Nitz/Marg: Combined dialysis clinic.

Marg: Dialysis and…

**Q: Diabetes and renal?**

Marg: Diabetes, yeah.

**Q: Anything else people would..?**

John: I would just say though, if you have a combined clinic, you don’t want to freeze out the private consultants. Just having the one in the clinic, that’s not the answer you need if they wish private ones as well.

**Q: Mm, anything else people want to add?**

Nitz: Why can’t the public hospital get the doctors, like the private doctors, to stay focussed for some patients that are more needy? More needed?

**Q: That’s why we are doing this <laughs>.**

Nitz: That’s what I’ve been saying, why rush? Like why put it into, like it’s like a processing plant, first one person, then, you can’t really see properly what’s the problem. They just rush like that all the time.

Lorraine: Sorry ladies and gentlemen, I’ve got to go.

Joe: I have to go too.

Nice to meet with you.

Nice to meet you too, yeah.

Nice to see you. Hope it goes well for you.

Thank you, yeah.

**Q: Okay, has anyone else got anything to add, or we’ll finish up here?**

NItz: Sometimes the things that you needed like I’m waiting for that machine, that machine for quite a while and I haven’t got it. I would like, you know, being, being ill like that, sometimes you feel like taking some, during the day, you want your day to be more available, because sometimes for me I’d like to take some courses, like some side courses to learn more.

John: A machine’s not the answer to that because you are locked to the machine for eight hours.

Nitz: I don’t mind that when I go to bed.

**Q: Okay, let’s, why don’t we..? I think we probably have to, why don’t we..? I think we’ll just stop. I think we’ll just stop now.**

Alright.

**Q: Let’s finish up now.**

END OF TRANSCRIPT
