# Supplementary material for: The Perspectives of Patients on Health-Care for Co-Morbid Diabetes and Chronic Kidney Disease: A Qualitative Study
Source: PLoS One. 2016 Jan 5;11(1):e0146615. doi: 10.1371/journal.pone.0146615 (PMC4701448; doi:10.1371/journal.pone.0146615)
Supplement: S1 Table — (DOCX) [file pone.0146615.s002.docx]

**Supplementary Materials**

**S1 Table: Initial Questions inventory for focus groups and semi-structured interviews**

| 1 | Think about the experiences that you/the person you have cared for have had with the diabetes and CKD services. Based on your experience, what would you say are weaknesses? |
| --- | --- |
| 2 | What are some of the things that you find frustrating about the service? |
| 3 | Is it easy to get the diabetes and kidney health care that you need (locality, cost/affordability, waiting times)? |
| 4 | Based on your experience, what would you say are the strengths of the diabetes and CKD health services that you/your relative received? |
| 5 | What do you find particularly effective about the diabetes and CKD health services? |
| 6 | What impact has attending the diabetes and renal health services made to your/your relative’s health? |
| 7 | Let us move onto your experiences with living with and looking after your/your relative’s diabetes and CKD: In your opinion, what do you/your relatives’ need to look after your/their diabetes and chronic kidney disease well? |
| 7 | What things make it hard for you/your relative to look after your diabetes and chronic kidney disease well? |
| 8 | Moving back to the health services provided for the management of your/your relatives diabetes and CKD. You’ve made some comments about strengths/weaknesses and likes and dislikes about the diabetes and CKD health services, and we would like you to make some recommendations. Imagine you became director of the hospital and you had the power to change things about the diabetes and CKD health services. If we were going to design a “perfect’ health service which would meet all your/your relative’s needs, what would it include?  What would be the key components of such a model? |
| 9 | We want you to help us evaluate these services to help improve the service and the difference it makes to patients. Is there anything that we have missed or that I should’ve asked you that I didn’t think to ask? |
